# Scalable near-real-time Bayesian phylogenetics for outbreaks with Delphy

**DOI:** 10.1038/s41586-026-11012-6

**Published:** 2026-09-16

**Authors:** Patrick Varilly, Mark Schifferli, Katherine Yang, Paul Cronan, Ivan Specht, Tim Burcham, Olivia Glennon, Olivia Jacks, Ellory Laning, Libby Marrs, Kyle Oba, Shannon Yeung, Karlie Wenran Zhao, Edyth Parker, Ifeanyi Omah, Jonathan E. Pekar, Laura Luebbert, Kristian G. Andersen, Daniel J. Park, Stephen F. Schaffner, Bronwyn L. MacInnis, Christian Happi, Jacob E. Lemieux, Al Ozonoff, Michael Mitzenmacher, Ben Fry, Pardis C. Sabeti

**Affiliations:** 1 Broad Institute of Harvard and MIT, Cambridge, MA USA; 2 Fathom Information Design, Boston, MA USA; 3 Institute for Computational and Mathematical Engineering, School of Engineering, Stanford University, Stanford, CA USA; 4 The Institute of Genomics and Global Health, Redeemer’s University, Ede, Nigeria; 5 Institute of Ecology and Evolution, University of Edinburgh, Edinburgh, UK; 6 Department of Parasitology and Entomology, Nnamdi Azikiwe University, Awka, Nigeria; 7 Eric and Wendy Schmidt Center, Broad Institute of Harvard and MIT, Cambridge, MA USA; 8 Department of Organismic and Evolutionary Biology, Faculty of Arts and Sciences, Harvard University, Cambridge, MA USA; 9 FutureHouse, San Francisco, CA USA; 10 Department of Immunology and Microbiology, The Scripps Research Institute, La Jolla, CA USA; 11 Scripps Research Translational Institute, La Jolla, CA USA; 12 Department of Immunology and Infectious Diseases, Harvard T.H. Chan School of Public Health, Harvard University, Boston, MA USA; 13 Massachusetts Consortium on Pathogen Readiness, Harvard Medical School, Harvard University, Boston, MA USA; 14 Department of Biological Sciences, Faculty of Natural Sciences, Redeemer’s University, Ede, Nigeria; 15 Department of Medicine, Massachusetts General Hospital, Harvard Medical School, Boston, MA USA; 16 Department of Pediatrics, Harvard Medical School, Boston, MA USA; 17 Department of Computer Science, School of Engineering and Applied Sciences, Harvard University, Boston, MA USA; 18 Howard Hughes Medical Institute, Chevy Chase, MD USA

**Keywords:** Phylogenomics, Software, Phylogenetics, Epidemiology, Epidemiology

## Abstract

Pathogen genomic analysis is central to tracking, understanding and containing outbreaks^1–13^, but the complexity and cost of state-of-the-art phylogenetic tools limit global access and impact. Here we introduce Delphy, an exact reformulation of Bayesian phylogenetics^14–17^ designed to transform its speed, scalability and accessibility while retaining Bayesian state-of-the-art accuracy. Delphy’s central data structure, an explicit mutation-annotated tree, takes advantage of the high sequence similarity of large-scale epidemic datasets^18–20^ for efficient tree exploration and convergence. By reproducing key analyses from recent major epidemics, including Ebola^1,21^, Zika^2^, SARS-CoV-2 (ref. ^22^), mpox^3,4^ and H5N1 (refs. ^23,24^), we demonstrate state-of-the-art accuracy with up to 2–3 orders of magnitude improvements in speed. Assessing Delphy’s scalability, we show that a simulated dataset of 100,000 sequences can be analysed within a day. We distribute Delphy as a client-side web application that enables local, interactive analysis of raw data on the user’s machine. Delphy automatically identifies key viral lineages and mutations, as well as their emergence and prevalence through time, with quantified uncertainties grounded in Bayesian theory. Delphy establishes Bayesian phylogenetics as a fast, accessible frontline tool for future outbreak response.

## Abstract (web summary)

Delphy makes near-real-time and scalable Bayesian phylogenetics possible for growing viral outbreaks, enabling public health bodies anywhere to analyse and react to their own data with state-of-the-art accuracy and minimal friction.

## Main

Large-scale sequencing of pathogen genomes, now routine using next-generation sequencing, provides considerable clarity into pathogen spread and evolution during outbreaks. However, the resulting massive datasets—illustrated by over 17 million publicly available SARS-CoV-2 genomes^25^—pose an equally substantial challenge to analyse and interpret.

Phylogenetics is a fundamental tool for organizing and interpreting pathogen genome sequences, inferring plausible trees of descent among sampled pathogens. Phylogenetic reconstruction enables diverse analyses, including (1) dating the start of outbreaks^1–3^; (2) distinguishing zoonotic spillovers from human-to-human transmission^1,4^; (3) identifying major lineages^5^; (4) detecting early warning signs of concerning new lineages^13^; (5) revealing community-based and geographically based spread patterns^4,6–8^; and (6) reconstructing detailed transmission networks^9–12^.

Among phylogenetic approaches, Bayesian phylogenetics, as implemented in widely used tools such as BEAST X^14^, BEAST2 (ref. ^15^), MrBayes^15,17^ and RevBayes^16^, is the most flexible and reliable, and underpins outbreak reconstruction and phylodynamics. Phylogenetic reconstruction has inherent uncertainties, which Bayesian methods quantify, simultaneously inferring other latent variables such as geographical spread^4^ or viral population dynamics^4,26^.

Despite its advantages, Bayesian phylogenetics remains technically complex and resource intensive. A handful of specialized research groups can handle this complexity, but it remains inaccessible to most epidemiologists and public health bodies. Bayesian techniques are so computationally expensive that outbreak-tracking infrastructures (such as NextStrain^27^ and Cov2Tree^28^) rely on approximate methods, such as maximum parsimony^18,29^ or maximum likelihood^30,31^. These methods typically generate a single tree topology, then post-process it with ad hoc techniques to infer uncertainties and latent variables^9,27,32,33^.

When applying Bayesian phylogenetics to nearly identical sequences, large computational gains are possible by reformulating calculations around local mutations rather than all mutated sites. This exact reformulation is always possible, but should be especially efficient when mutations are sparse. Outbreak sequences often differ from their closest related sequence at as few as 0 to 2 sites^18–20^, and therefore clearly exhibit such sparsity.

Recent work exploiting low outbreak sequence diversity has substantially improved the performance and scalability of maximum-parsimony^18,29^ and maximum-likelihood^31^ methods, suggesting that similar gains are possible in a Bayesian framework. However, previous attempts had considerable limitations: fixed tree topology, simplified evolution models, inability to handle missing data, no site rate heterogeneity and the absence of an ancestry prior^34–42^. These efforts also missed key efficiencies, such as avoiding operations in inner loops that scale with genome size and leveraging parallelism. Thus far, no Bayesian phylogenetics tool using an explicit mutation representation addresses the real-world genomic epidemiology dataset complexities needed for broad use.

Here we present Delphy, the result of reimagining and rebuilding every portion of the Bayesian phylogenetics pipeline to make it fast, accurate, accessible and scalable for large-scale outbreak data. Our key goals are to (1) enable frontline workers to analyse their own data using state-of-the-art Bayesian methods, without specialized training or resources beyond a laptop; (2) rapidly generate interpretable, publication-quality analysis and figures from raw aligned sequences for public health action; and (3) scale efficiently to accommodate growing genomic datasets without sacrificing accuracy.

## EMATs

**Fig. 1:**
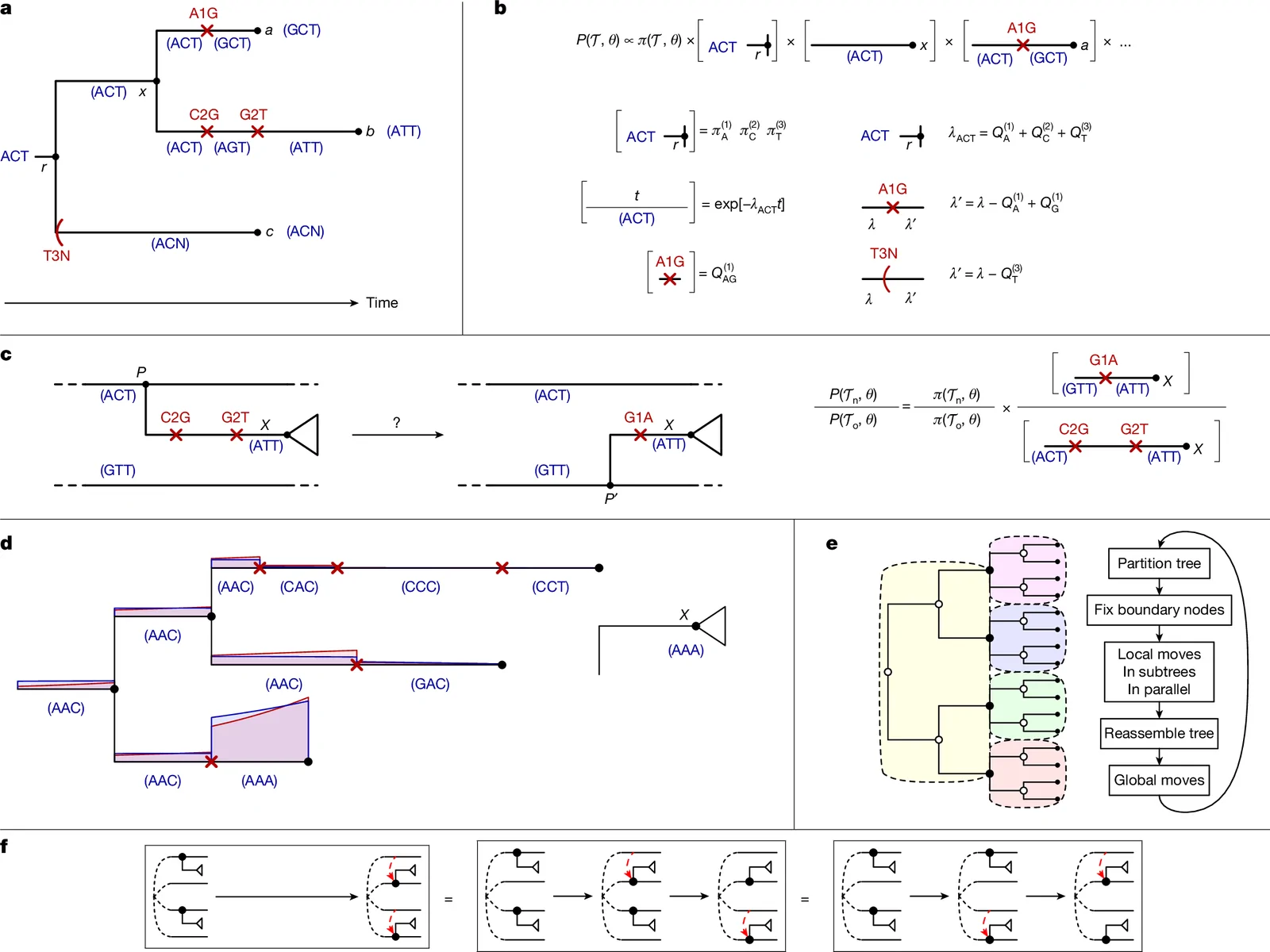
Key elements of Delphy. **a**, Phylogenetic trees are represented by EMATs, timed bifurcating trees with an explicit root sequence and explicit mutation and missation events on branches at explicit times; every point has a specific but implicitly encoded sequence (in parentheses).** b**, The posterior distribution *P*(${\mathcal{T}}$,*θ*) sampled by Delphy is dominated by a genetic prior—a product of three kinds of simple terms that mirror the tree structure; it can be calculated with the simple rules shown, in time proportional to the number of elements in the tree (further details (including definitions of symbols) are provided in the accompanying text and the Methods). **c**, Schematic of an SPR move. When pruning a subtree rooted at *X* from *P* and regrafting it at *P*′, we fix everything except the branch above *X*, so the ratio of posteriors of the new tree ${\mathcal{T}}$_n_ and old tree ${\mathcal{T}}$_o_ is dominated by a simple ratio of small numbers of factors, dependent only on what happens along that branch. **d**, Schematic of an mdSPR move: when evaluating possible regrafting locations for the subtree rooted at *X*, the EMAT tree structure facilitates proposals according to a simple Jukes–Cantor model (red) that closely tracks the real conditional posterior (blue); thus, plausible large-scale rearrangements are proposed and accepted with high probability. **e**, Schematic of the scaling strategy: the proposal and acceptance of local tree rearrangements in an EMAT is independent of distant portions of the tree; a tree can therefore be partitioned into smaller subtrees, each subject to independent, parallel MCMC moves and then reassembled to apply global moves. **f**, Moves can be parallelized because the probability of proceeding from an initial state to a particular final state in parallel is equal to that when moves are interleaved arbitrarily and attempted serially.

Delphy’s computations are organized around explicit mutation-annotated trees (EMATs)—timed phylogenetic trees that explicitly encode the mutations linking an ancestral sequence to dated observed sequences. Each EMAT represents one concrete hypothesis for how those sequences arose, mutation by mutation (Fig. 1a). Tips correspond to observed sequences; the inner nodes correspond to the most-recent common ancestors (MRCAs) of their descendant tips; and branches trace lineages through time. Every point on the tree represents a sequence that existed at a specific time and is ancestral to an observed sequence. EMATs represent the root sequence explicitly and all other sequences implicitly as accumulated mutations from the root. Branches contain explicit, time-stamped mutation and missation events, with missations marking missing data at specific sites in downstream tips (see below). EMATs are inspired by UShER’s mutation-annotated trees, but extend them by (1) assigning time-stamps to each node and mutation; (2) allowing multiple mutations on the same site along a single branch; and (3) explicitly encoding missing data.

Given a set of dated observed sequences, Delphy samples EMATs ${\mathcal{T}}$ and associated model parameters *θ* from a posterior distribution *P*(${\mathcal{T}}$,*θ*). This distribution is proportional to two factors. The first, a prior distribution on (${\mathcal{T}}$,*θ*), represents the probability that randomly drawn sequences are ancestrally related as in ${\mathcal{T}}$ and evolve from a random starting point. The parameters *θ* include the mutation rate, the relative likelihood of different mutations and the viral population size over time. The second factor, the data likelihood given (${\mathcal{T}}$,*θ*), is currently trivial because Delphy neglects sequencing error: it is 1 if the tip sequences in (${\mathcal{T}}$,*θ*) match the observations, and 0 otherwise.

The prior distribution is largely shaped by a genetic prior, which represents the probability that a randomly chosen root sequence evolves along ${\mathcal{T}}$ according to its explicit mutations (Fig. 1b). Evolution is modelled independently at each site *ℓ* as a continuous-time Markov chain with transition rate matrix ${Q}_{{ab}}^{({\ell })}$, and escape rates ${Q}_{a}^{({\ell })}$ = −${Q}_{aa}^{({\ell })}$. The stationary distribution ${{\pi }}_{a}^{({\ell })}$ of the chain at site *ℓ* serves as the prior distribution for the root sequence. In an EMAT, the genetic prior factors into simple terms: one for the root sequence involving ${{\pi }}_{a}^{({\ell })}$, one for each branch involving ${Q}_{a}^{({\ell })}$ and one for each mutation involving ${Q}_{{ab}}^{({\ell })}$. Apart from the genetic prior, the remaining factors of the posterior, denoted by *π*(${\mathcal{T}}$,*θ*), relate to ancestry and parameter priors in standard ways (Methods and Supplementary Information). We avoid calling the genetic prior an augmented likelihood or a complete data likelihood because Delphy’s model includes the full mutational history leading to the observed sequences and therefore, in principle, allows a straightforward coupling of sequence and ancestry to model selection effects. Separating ancestry and genetics into an ancestry prior and augmented or complete likelihood needlessly obscures such a coupling.

The genetic prior functions similarly to the Felsenstein tree likelihood in traditional Bayesian phylogenetics^14,15,43^, making EMAT sampling analogous to sampling tree topologies in that framework. For a fixed topology and tip sequences, integrating the genetic prior over all possible root sequences and mutation histories yields the Felsenstein tree likelihood (Supplementary Information). Whereas traditional methods perform this integration analytically, Delphy folds it into the sampling: it simultaneously explores possible tree topologies, root sequences and mutation histories, of which only a few are typically plausible. Calculating the Felsenstein tree likelihood accounts for all such possibilities, but an overwhelming effort goes to vanishingly rare ones. For small datasets with limited diversity, this effort can be reduced by grouping sites by site patterns, identical state patterns across input sequences of which the contributions need only be calculated once. In Delphy’s genetic prior, site patterns have no role: every site has a fully independent mutational history over an EMAT.

Delphy samples models (${\mathcal{T}}$,*θ*) from the posterior distribution *P*(${\mathcal{T}}$,*θ*) using a standard Markov-chain Monte Carlo (MCMC) algorithm, generating a sequence of correlated samples by repeatedly proposing small changes, or moves, that are probabilistically accepted^44^. The most common acceptance probability follows the Metropolis–Hastings criterion, which uses the ratio of the posterior *P*(${\mathcal{T}}$,*θ*) before and after the proposed change^45,46^. Moves whereby most factors in this ratio cancel out can be proposed and evaluated efficiently.

Delphy’s central move, subtree pruning and regrafting (SPR) (Fig. 1c), is computationally efficient on EMATs. SPR moves propose detaching a subtree rooted at *X* from its attachment point *P* and reattaching it elsewhere at* P*′. In an EMAT, a new mutational history must also be proposed, currently restricted to the *P*′–*X* branch so the posterior ratio involves only a few factors from that branch. As mutations on that branch are overwhelmingly dictated by end-point sequence differences, we use a simplified Jukes–Cantor model for proposals and rely on the Metropolis–Hastings criterion to correct approximation errors (Supplementary Information). When handling missing data, missations on the path from *P* to *P*′ (see below) introduce additional but manageable complexity (Supplementary Information).

Delphy is much faster than traditional tools because it does far less work: EMAT moves involve orders of magnitude fewer operations than analogous moves using the Felsenstein tree likelihood. Calculating Felsenstein tree likelihood ratios may require hundreds of thousands of operations, as partial site likelihoods must be recalculated for every variable site at every node between the attachment points and the root node. By contrast, posterior ratios for EMAT moves require only a handful of local operations, proportional to the number of affected mutations. The trade-off is that EMAT-based moves are less statistically efficient, requiring more moves between uncorrelated samples, but the cost savings per move typically more than compensate.

The locality of Delphy’s moves opens the door to pandemic-scale analyses: the genetic prior, in contrast to the Felsenstein tree likelihood, factorizes over subtrees, so Delphy can parallelize moves across the tree (Fig. 1e,f). For example, Fig. 1c shows how the genetic prior ratio for an SPR move depends only on factors linked to the *P*–*X* and *P*′–*X* branches. If the rest of the posterior is also local, posterior ratios for moves on non-overlapping subtrees are independent of move order. In a maximum-parsimony context, this motivates the parallelization strategy of matOptimize^18^: it concurrently evaluates many potential SPR moves, then applies the largest non-conflicting beneficial subset. In Delphy’s MCMC, we periodically partition the tree, concurrently apply local moves in each partition and then reassemble for global moves (Fig. 1e). This preserves correct sampling because it is equivalent to attempting local moves sequentially in an arbitrary interleaving (Fig. 1f). Allowing any EMAT partitioning to be proposed maintains ergodicity of the underlying Markov chain (a mild approximation must be introduced when coupling to a coalescent prior; Supplementary Information).

Delphy’s EMATs facilitate efficient proposals of large-scale beneficial rearrangements through a process that we call mutation-directed SPR (mdSPR) (Fig. 1d), enhancing convergence without fine-tuning MCMC move parameters. An mdSPR move generalizes the core idea of UShER from maximum-parsimony to a Bayesian context: instead of placing a new sample at the most parsimonious attachment point, mdSPR proposes arbitrary attachment points while biasing towards those requiring fewer mutations, even when far away. As mutations are explicit, Delphy quickly evaluates the minimum mutations required for any proposed *P*′–*X* branch. Under a Jukes–Cantor model without site-rate heterogeneity, where parsimonious histories dominate, one can write a closed-form conditional posterior for* P*′ (blue) that can be sampled to propose *P*′. The Jukes–Cantor-inspired proposal distribution closely tracks the real conditional posterior for *P*′ (red). Thus, the Metropolis–Hastings criterion achieves high acceptance probabilities even when *P*′ is far from *P*, with occasional rejections correcting the bias in the approximate proposal. However, mdSPR’s robustness degrades when many tip dates have large uncertainties. We speculate that the entropy across many uncertain branch lengths overwhelms the constraints from tip sequences, a problem that is probably not unique to Delphy. For now, Delphy is unsuitable for such datasets.

Finally, Delphy introduces *N-*pruning, a missing-data technique that maintains move speed and parallelizability by preserving the genetic prior’s functional form. Naively, an EMAT fully specifies the sequence at each tip, imputing the state of sites with missing data. However, during SPR moves on subtrees with missing data, such imputation would require either global rearrangements or spurious mutations that severely degrade sampling efficiency (Supplementary Information). Delphy circumvents these tensions by not imputing missing sites in such subtrees. As the root node state of such subtrees must evolve into something, integrating over all mutation histories at those sites causes the associated branch and mutation factors to disappear from the genetic prior while preserving its functional form (Fig. 1b (bottom right corner)). This integration is equivalent to Felsenstein pruning on sites and subtrees ending in missing data. Conceptually, if we view an EMAT as a product of site trees, one per site, then *N*-pruning recursively prunes all branches on such site trees that lead to tips with missing data (Supplementary Fig. 11). Missations mark where this pruning occurs. *N*-pruning’s main drawback is additional bookkeeping and complexity in SPR moves (Supplementary Information). A lesser limitation is that partially ambiguous states (such as Y = C or T) must be treated as missing data; such partial ambiguity is rare in our benchmarks.

Delphy introduces many ideas, and its core MCMC implementation comprises around 8,000 new lines of numerically sensitive and data-structure-heavy C++ code. We carefully validated its correctness using the Well-Calibrated Simulation Study framework of ref. ^47^ (Supplementary Information 15). These studies exercised all major features, providing strong evidence that the core MCMC is free of material defects.

## Web-based interface and analyses

Delphy makes Bayesian phylogenetics globally accessible through a fully web-based implementation that eliminates installation or compilation and keeps data with the data generator. In contrast to similar tools, Delphy can run directly in a web browser as a client-side application (https://delphy.bio). The only required input is a multiple-sequence alignment in FASTA or MAPLE^31^ format, with dates, possibly inexact, in the sequence IDs. An optional metadata file can annotate samples with categorical labels (Methods). A TypeScript front-end processes these inputs and communicates with Delphy’s C++ core, compiled into WebAssembly; data never leave the user’s machine. The front-end manages execution and provides an interactive interface to explore results.

**Extended Data Fig. 1:**
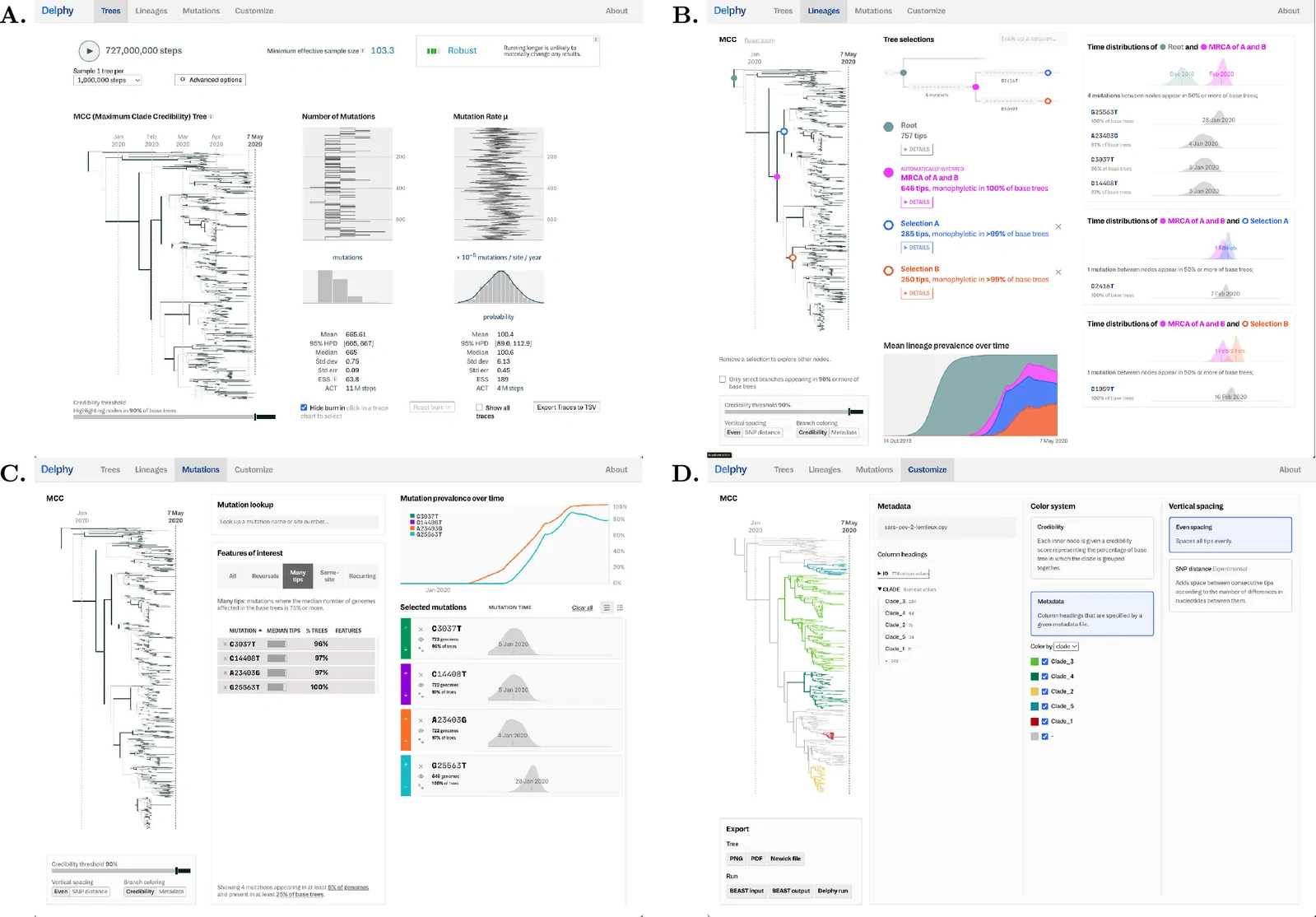
Delphy’s web interface. **A**. The *Trees Panel*, controls the MCMC run and monitors progress. The MCC tree (left) summarizes sample posteriors, highlighting high-confidence nodes. Traces of observables (right) allow monitoring progress. A heuristic suggests a burn-in/production cutover point, which can be overridden (Methods); subsequent analyses use only production samples. Effective Sample Sizes (ESSs) for tracked observables are estimated and summarized in a Minimum ESS (excluding the less reliable ESS estimate for mutation count, which, often taking only a few discrete values, is not well-modelled by a correlated Gaussian random walk) (top). **B**. The *Lineages Panel* is a lineage-centric view of the results. Users hover over the MCC (left) to select up to two nodes (blue and orange) and their common ancestor (pink). Each MCC node represents the most-recent common ancestor (MRCA) of its downstream tips; high-support nodes correspond to distinguishable lineages. Dominant relationships between selected nodes appear in a minimap (top centre). Their time to MRCA (tMRCA) distributions and the identity and time distribution of intervening mutations are shown (right). Using the population model, Delphy estimates a time-varying lineage prevalence histogram (bottom centre; Methods). All distributions can be probed by hovering. **C**. The *Mutations Panel* is a mutation-centric view. Delphy automatically identifies potentially interesting mutations (centre), selecting those reaching high prominence. For each mutation, time distributions (bottom right) and prevalence (top right) are shown. Hovering reveals prevalence uncertainty and the approximate tree location of each mutation. **D**. The *Customize Panel* allows annotating the MCC with categorical metadata such as clades or geographical labels. A naive Fitch parsimony algorithm propagates metadata to interior MCC nodes (coloured tree on the left). Users can export an image or Newick file of the MCC, raw results in BEAST format for post-processing, or in “Delphy” format for reloading.

Delphy’s web interface guides users through the full workflow, from data input to interactive analysis and result export (Extended Data Fig. 1). Users can monitor the MCMC run’s burn-in and convergence, analyse results interactively and export publication-ready summaries and raw outputs. Built-in analyses include standard outputs: the maximum-clade-credibility tree (MCC, calculated at interactive speeds through clade fingerprinting; Supplementary Information), key lineages (high-support MCC nodes), time-to-most-recent-common-ancestor (tMRCA) estimates for the whole dataset and sublineages, and ancestral state reconstruction for metadata. Delphy’s explicit-mutation representation enables additional analyses, including mutation identities and time distributions between any two nodes, as well as experimental visualizations, such as lineage and mutation prevalence curves (Methods).

We designed Delphy to make uncertainty explicit while remaining usable, extensible and interoperable with existing phylogenetic workflows. The interface displays posterior supports, 95% highest-posterior density (HPD) ranges for time and prevalence distributions, and ambiguous mutation placements while keeping these details unobtrusive for occasional users. For analyses not yet in Delphy, such as calculating CCD0 (ref. ^48^) or HIPSTR^49^ trees instead of MCCs, users can export results in BEAST-compatible trees and log files.

## Exact and 2–3 orders of magnitude faster

Delphy’s results should be statistically indistinguishable from those of existing tools for implemented models, and remain so as we add models. In effect, Delphy samples the same tree distribution as these tools once explicit mutation events are projected out, apart from an arbitrarily tuneable discretization error introduced by our treatment of the coalescent prior. To facilitate direct comparisons, Delphy can export XML input and output files for equivalent BEAST X and BEAST 2 runs.

We benchmarked Delphy against BEAST X and BEAST 2, state-of-the-art Bayesian tools, and IQ-Tree 2 + TreeTime, the core of NextStrain’s maximum-likelihood pipeline, using published SARS-CoV-2, Zika, Ebola and H5N1 datasets. These included 757 SARS-CoV-2 sequences from early 2020 (ref. ^22^), 174 Zika sequences from 2015–2016 (ref. ^2^) and 1,610 Ebola sequences from the 2014–2016 West Africa outbreak^1,21^. We focus in this section on the SARS-CoV-2 and Ebola results. Supplementary Figs. 2–5 show Zika and a smaller Ebola dataset. All study datasets are available as demonstrations (https://delphy.bio).

**Fig. 2:**
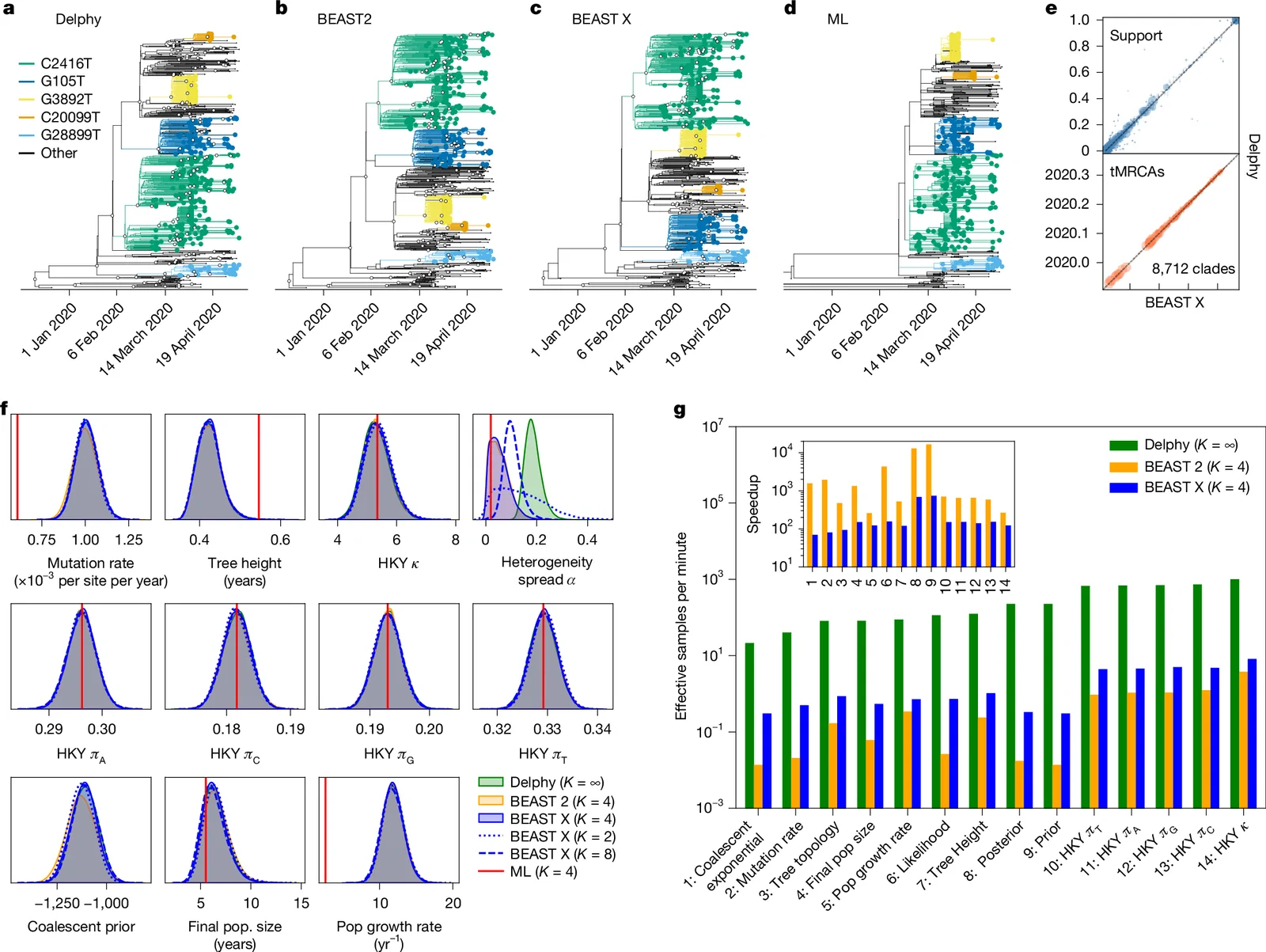
Delphy is consistent with existing Bayesian tools. **a**, MCC tree for 757 SARS-CoV-2 samples from ref. ^22^, as produced by Delphy (the results recapitulate figure 3a of ref. ^22^). Inner nodes with posterior support above 95% are marked with white dots; local rearrangements of low-support nodes are not highly significant and are expected even between identical finite runs. Interior labels are imputed by naive parsimony on the MCC. **b**,**c**, Analogue for BEAST X (**b**) and BEAST 2 (**c**). **d**, Maximum-likelihood (ML) timed tree, as calculated by IQ-Tree 2 and TreeTime. **e**, Clade correlation graphs between Delphy and BEAST X runs. **f**, The distributions of key observables, as produced by Delphy (green) and BEAST 2 (orange), BEAST X (blue) and maximum-likelihood estimate (red); the discrepancy in the heterogeneity spread parameter *α* arises from Delphy’s use of a continuous gamma model versus a *K*-category discrete approximation in the BEAST runs (Methods); the curves for *K* = 2 (dotted line), *K* = 4 (the standard setting, filled blue) and *K* = 8 (dashed line) demonstrate the sensitivity of *α* to *K* and its convergence towards the Delphy result (*K* = ∞). Pop, population. **g**, The runtime efficiency of Delphy versus BEAST X and BEAST 2. The visual style and colouring of diagrams in **a**–**d** were adapted from figure 3a in ref. ^22^ under a CC BY 4.0 licence.

Delphy- and BEAST-inferred MCC trees for the SARS-CoV-2 dataset show excellent agreement (Fig. 2a–c). The MCCs, point summaries of the full posterior distribution, have the same overall structure and timing; vertical rearrangements of low-support inner nodes (lacking white dots) are expected even between independent BEAST runs. The maximum-likelihood tree (Fig. 2d) also captures the essential lineage structure, but infers tMRCAs of major lineages less accurately. Clade correlation graphs^47^ between Delphy and BEAST X runs (Fig. 2e and Methods) show compatible posterior supports and mean tMRCAs; the few outliers reflect differing treatment of site-rate heterogeneity (see below) and rugged tree landscapes^50^ (Supplementary Information 14).

Key parameter distributions, such as mutation rate, also show excellent agreement between Delphy and BEAST (Fig. 2f). The only significant deviation is in the site-rate heterogeneity parameter *α*, reflecting a deliberate model difference: Delphy uses a continuous gamma model, whereas BEAST uses a discrete *K*-category approximation owing to technical limitations. Typical usage has *K* = 4; the results for *K* = 2 and *K *= 8 demonstrate *α*’s sensitivity to *K* and convergence toward the Delphy result (*K* = ∞). A direct comparison of runs with no site-rate heterogeneity shows no significant discrepancies (Supplementary Fig. 1). By contrast, maximum-likelihood methods appear to infer some parameters accurately but not others, particularly the population curve and, by extension, the mutation rate and tree height.

Although Delphy, BEAST 2 and BEAST X produce essentially indistinguishable results for the SARS-CoV-2 dataset, Delphy produces the results 2–3 orders of magnitude faster than BEAST 2 and about 2 orders of magnitude faster than BEAST X (Fig. 2g). Speed is measured as the rate at which each MCMC run produces effectively uncorrelated samples (Methods). Although speed estimates vary by observable, Delphy’s relative speedup over BEAST 2 and BEAST X is robust.

**Extended Data Fig. 2:**
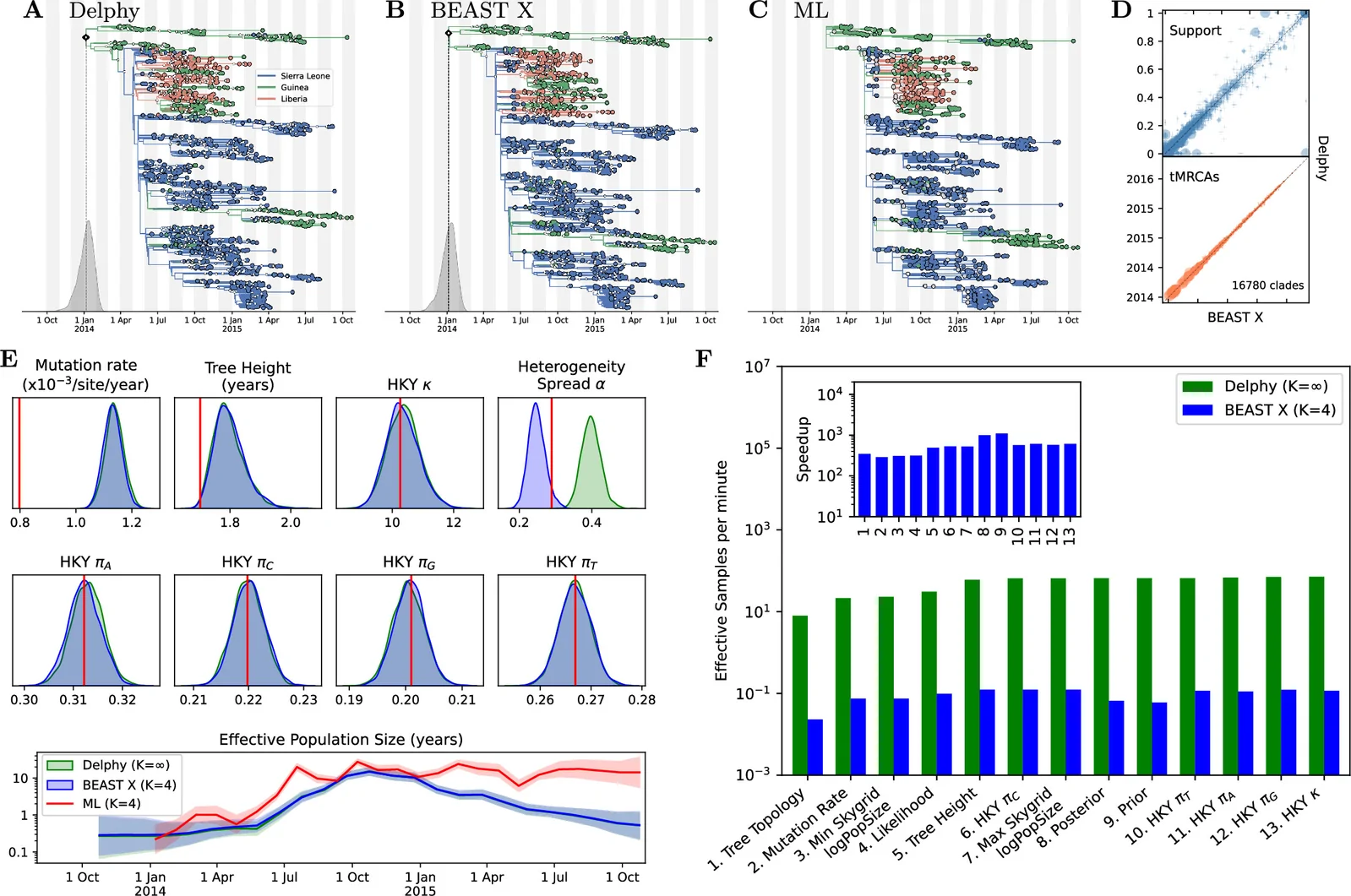
Delphy is consistent with existing Bayesian tools (Ebola example). **a**, MCC tree for 1,610 Ebola sequences spanning 2014–2016 from ref. ^21^. Inner nodes with posterior support above 95% are marked with white dots; local rearrangements of low-support nodes are not highly significant and are expected even between identical finite runs. Phylogeographical reconstruction reflects naive parsimony on the MCC. **b**, Analogue for BEAST X. **c**, Maximum-likelihood timed tree, as calculated by IQ-Tree 2 and TreeTime. **d**, Clade correlation graphs between Delphy and BEAST X runs. **e**, The distributions of key observables (effective population size confidence bands cover 95% HPD). **f**, The runtime efficiency of Delphy versus BEAST X.

For the larger 1,610-sequence Ebola dataset, which spans periods of exponential growth and decline, we implemented a standard Skygrid^51^ flexible population model (Methods and Extended Data Fig. 2). BEAST 2 does not offer this model, so we excluded it from the comparison. Delphy and BEAST X again show excellent agreement in MCCs (Extended Data Fig. 2a,b), clade correlation graphs (Extended Data Fig. 2d) and parameter distributions (Extended Data Fig. 2e), while maximum likelihood captures the essential lineage structure (Extended Data Fig. 2c) but is less reliable for tMRCAs and the population curve. Delphy’s statistical efficiency (Extended Data Fig. 2f) is consistently close to three orders of magnitude higher than that of BEAST X.

Delphy’s advantage over Felsenstein pruning grows with dataset size and sampling density (Discussion). For example, the relative efficiency is even higher for the 3,339-sequence H5N1 dataset described below. Conversely, for the 81-sequence Ebola and 174-sequence Zika datasets, Delphy is only modestly more efficient than BEAST 2 or BEAST X. The Zika dataset has the lowest sampling density among our benchmarks (around 1,200 mutations, averaging about 4 mutations per branch). Although Delphy remains exact there, it slows down, as reflected in a tree topology effective sample size (ESS) per minute per sequence of about 7 times lower than in the other benchmarks.

## A flexible computational core

Delphy shares the same mathematical foundations as other Bayesian methods, enabling support for diverse model variations with the same accuracy. These include diverse evolution models, advanced site-rate heterogeneity models, flexible ancestry priors, including coalescent, birth–death or epidemic models, and phylogeography. For its initial release, Delphy uses a single-partition Hasegawa–Kishino–Yano (HKY) evolutionary model^52^ and a coalescent model with flexible population growth (Supplementary Information). Future updates will relax these restrictions on the basis of user needs.

As a proof of concept for Delphy’s flexibility, its initial public release includes a model for analysing human mpox virus (hMPXV) sequences^3,4^ (Methods). In contrast to most viruses, hMPXV accumulates mutations primarily through APOBEC3 activity in humans rather than replication errors^3^. APOBEC3 targets TC and GA dimers, mutating them to TT and AA dimers, respectively. Delphy formalizes this with a two-partition model closely resembling the post-zoonosis model of refs. ^3,4^ (Methods).

**Fig. 3:**
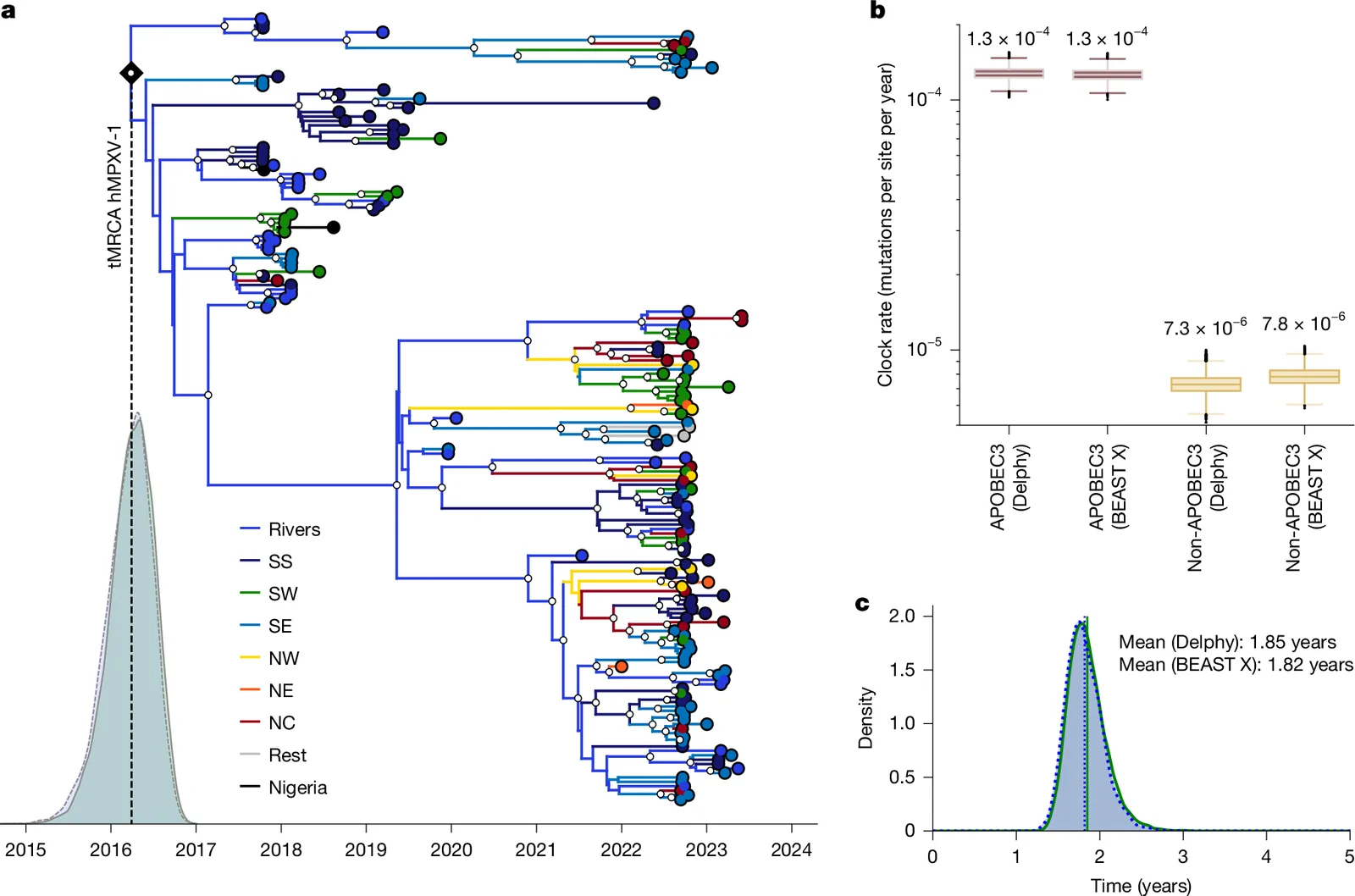
Delphy is flexible. **a**, MCC tree for 177 hMPXV-1 samples from ref. ^4^ as produced by Delphy (compare with figure 3a and extended data figure 1 of ref. ^4^). The tMRCA distributions are for Delphy (green) and BEAST X (blue). Tips and branches are coloured by parsimonious assignment of Nigerian state on the MCC. All states other than Rivers in the South-South (SS) region are grouped by region. The original zoonosis event probably occurred in the South-South region, probably in Rivers state. NC, North-Central; NE, North-East; NW, North-West; SE, South-East; SW, South-West. **b**, Inferred mutation rates due to APOBEC3 and non-APOBEC3 mechanisms. The box plots show the median (centre line) and interquartile range (shaded box), and the whiskers extend 1.5× the interquartile range beyond the box; outliers beyond are shown as dots. **c**, The inferred doubling time under an exponential growth model (Delphy, green; BEAST X, blue).

Delphy’s analysis of hMPXV-1 sequences, descendants of the spillover that led to worldwide mpox infections from 2022, closely aligns with the BEAST results. Figure 3 presents Delphy’s results for 177 hMPXV-1 sequences from ref. ^4^. While we omitted non-hMPXV-1 sequences to avoid modelling the spillover (Methods), Delphy’s agreement with BEAST is excellent. The MCC clearly recovers the clade structure (Fig. 3a). Despite its simplicity, Delphy’s MCC parsimony reproduces the ref. ^4^ central conclusion at regional resolution: the spillover event above the hMPXV-1 root node probably occurred in Nigeria’s South-South region. However, in contrast to their phylogeographic reconstruction (figure 5b of ref. ^4^), Delphy’s simple parsimony cannot establish a finer geographical state-level assignment to Rivers with high certainty.

Small quantitative differences with ref. ^4^ can be attributed to dataset composition. Delphy infers a slightly later tMRCA, a higher non-APOBEC3 mutation rate and a modestly faster doubling time. These differences probably reflect purifying selection in the sparsely sampled pre-spillover portion of the tree, which would suppress the observed non-APOBEC3 mutation rate in ref. ^4^ (Methods). Adapting the BEAST run of ref. ^4^ to include only hMPXV-1 samples, thereby omitting spillover and phylogeographic modelling, yields much closer agreement (Fig. 3). Similar agreement is observed for the earlier dataset from ref. ^3^ (Supplementary Fig. 6).

## Enabling real-time outbreak response

To assess Delphy’s use for real-time public health response, we analysed all complete SARS-CoV-2 sequences submitted to GISAID from 1 January to 28 March 2020 (CDC Epiweeks 2020-02 to 2020-13). To emulate the limited knowledge and urgency of outbreak investigations, we applied minimal processing, trimming sequence ends and removing clear outliers without masking sites later identified as problematic (Methods; results using sequences collected by each CDC Epiweek are provided in Supplementary Fig. 7).

**Extended Data Fig. 3:**
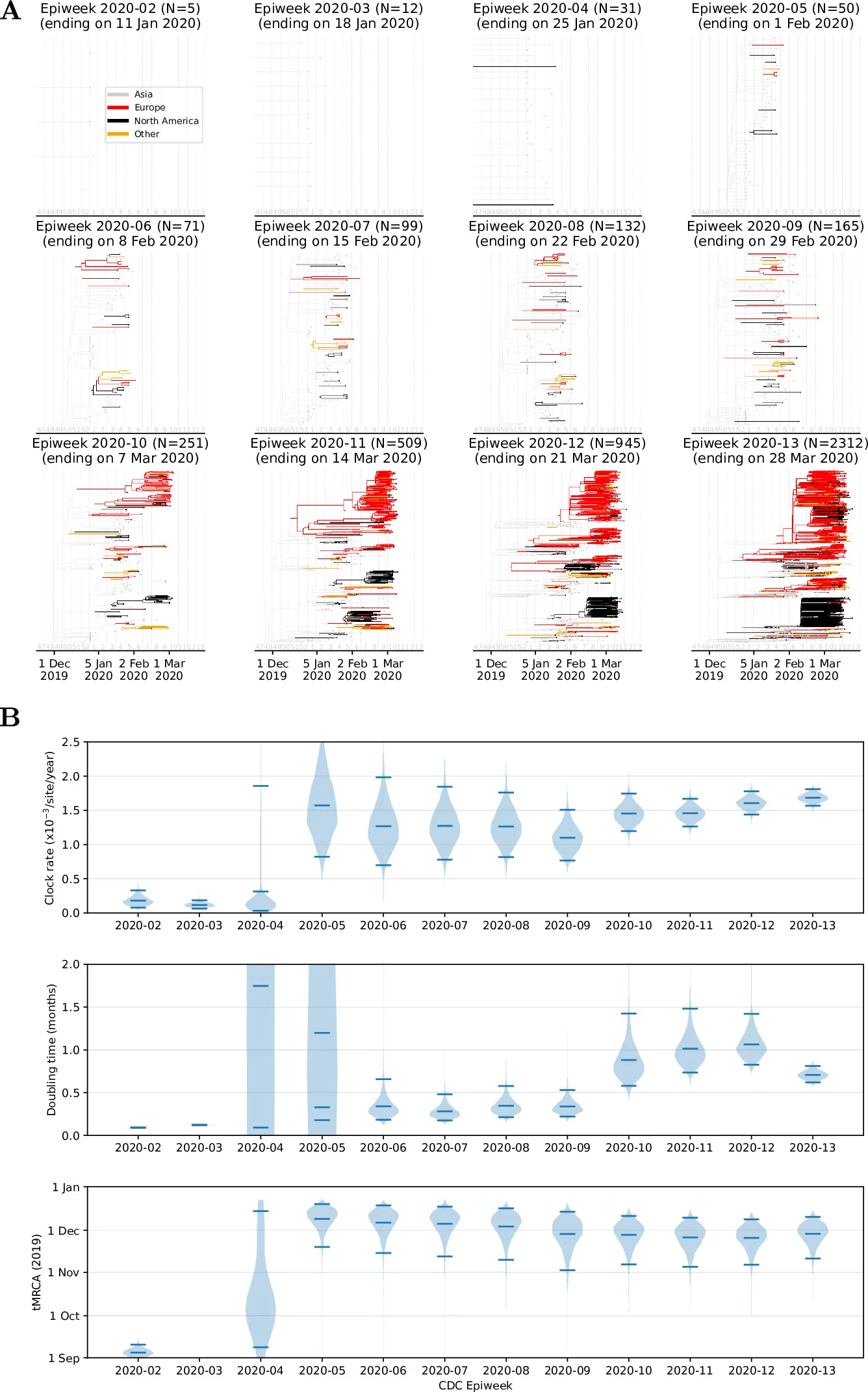
Delphy’s view of the first weeks of the COVID-19 pandemic. **A**. MCC trees derived from the SARS-CoV-2 sequences submitted to GISAID up to 28 Mar 2020 (CDC Epiweek to 2020-13). Branches coloured by continent as derived from naive parsimony. Before CDC week 2020-04 (ending on Sat 25 Jan 2020), the data is clearly insufficient to infer trees reliably. From 2020-05, trees are relatively stable. Many independent introductions to both Europe and North America, as well as persistent local transmission chains, are already plainly visible by the end of CDC week 2020-06 (ending on Sat 8 Feb 2020). **B**. Distributions for mutation rate, doubling times and tMRCAs derived from GISAID data submitted by successive CDC weeks; lines show mean, 2.5% percentile and 97.5% percentile. The mutation rate is overestimated vs. consensus value of around 1.0 × 10^−^^3^ / site / year, possibly owing to no masking of known problematic sites and minimal culling of outliers, as would happen near the early stages of an outbreak (Methods).

We generated inferred trees and associated parameter distributions, including mutation rates, doubling times and tMRCAs, as they would have been available in near real time to a public health responder (Extended Data Fig. 3a,b). Even simple parsimony on the MCC reveals that, by 8 February 2020 (CDC Epiweek 2020-06), many independent introductions from Asia into Europe and North America had occurred, followed by onward local transmission. Despite potential biases from minimal data filtering, the basic parameters of the growing epidemic—including a mutation rate of 1 × 10^−3^ to 2 × 10^−3^ mutations per site per year, a doubling time of 10–20 days, and a tMRCA around early December 2019—would all have been evident by 1 February 2020 (CDC Epiweek 2020-05).

**Fig. 4:**
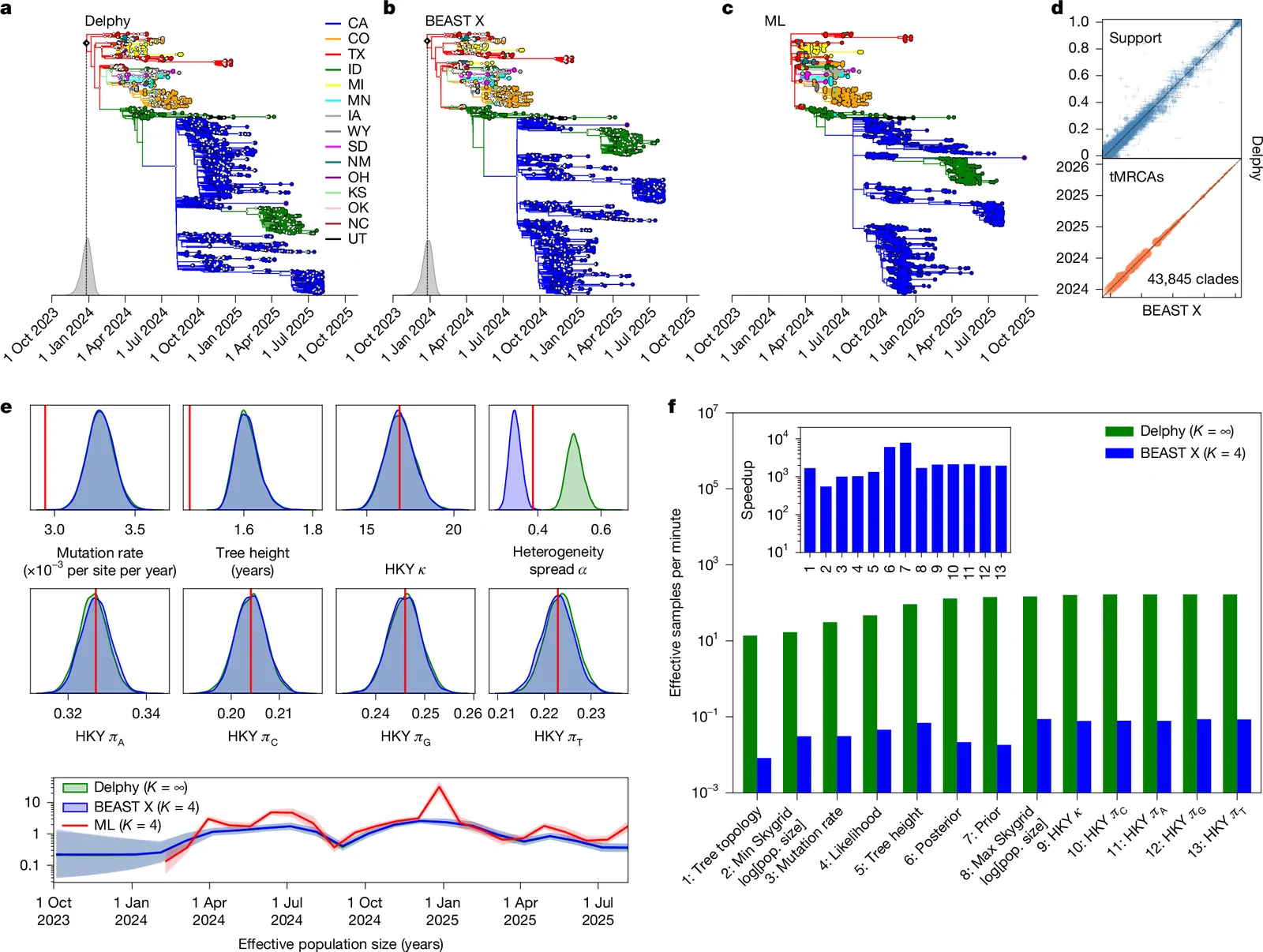
H5N1 influenza outbreak in US cattle in 2024–2025. **a**, MCC tree for 3,339 H5N1 B3.13 sequences in cattle as produced by Delphy. Inner nodes with posterior support above 95% are marked with white dots; local rearrangements of low-support nodes are not highly significant and are expected even between identical finite runs. Phylogeographical reconstruction reflects naive parsimony on the MCC. **b**, Analogue for BEAST X. **c**, Maximum-likelihood timed tree, as calculated by IQ-Tree 2 and TreeTime. TreeTime flagged two tips as outliers, of which the apparent and stated dates are highly inconsistent (red circles in **a**–**c**). **d**, Clade correlation graphs between Delphy and BEAST X runs. **e**, The distributions of key observables (effective population size confidence bands cover 95% HPD). **f**, The runtime efficiency of Delphy versus BEAST X.

We applied Delphy in real-time to the H5N1 influenza outbreak in US cattle, first detected in early 2024 (ref. ^53^). We created a daily updated Delphy analysis of the latest dated H5N1 sequences—as curated by the K.G.A. laboratory with dates and locations from GenBank, available at https://delphy.bio/us-h5n1-latest. Figure 4 shows Delphy’s MCC tree as of early October 2025 (Methods), with states colour-coded by location through naive MCC parsimony. Also shown are the equivalent BEAST X and maximum-likelihood (IQ-Tree 2 + TreeTime) trees, with clade correlation, distribution and efficiency comparisons as in Fig. 2. Delphy accurately recovers the known outbreak dynamics^24,53^: an origin in Texas farms, slow spread to other states and a tMRCA into cattle around 26 December 2023 (95% HPD, 2 December 2023 to 15 January 2024). The overall mutation rate across all segments is 3.3 × 10^−3^ per site per year (95% HPD, 3.1–3.5), consistent with previous influenza estimates^26^. For comparison, Delphy took 42 min to produce these results; BEAST X was interrupted before reaching halfway after 21 days; and IQ-Tree 2 took several hours.

In two independent H5N1 runs, the mutation count trace has a small discontinuity mid-run (from about 4,364 to 4,358 mutations in one run, and around 4,361 to 4,357 in the second). These discontinuities, undetectable in other traces, produce sporadic outliers in clade correlation graphs, presumably due to rearrangements of localized subtrees through improbable intermediates. Similar difficulties arise in maximum parsimony and maximum likelihood, whereby long searches continue to find small improvements at decreasing speed^18^. Regardless, the effect on key public health quantities, such as population curves and tMRCAs, is negligible^50^, and large-scale topological exploration seems to be unaffected (frechetCorrelationESS of 576 and 467).

A substantial portion of recent public H5N1 sequences lacks precise dates or locations—about 18% as of early October 2025, probably covering the preceding 3 months. Incorporating these sequences presents considerable convergence challenges that are probably not unique to Delphy (Supplementary Information 14). While imposing stronger priors on collection dates might help, we instead excluded these sequences from this paper and our daily-updated trees. However, data generators with precise metadata can easily adapt our Delphy runs for private analyses. If precise metadata become publicly available, our runs will automatically incorporate all sequences.

## Pandemic-scale Bayesian phylogenetics

To be useful in a pandemic, Bayesian phylogenetics tools must handle very large datasets accurately and rapidly. To evaluate Delphy’s performance on pandemic-scale data, we generated synthetic trees of increasing size and measured how quickly and accurately Delphy reconstructed them from tip sequences.

**Fig. 5:**
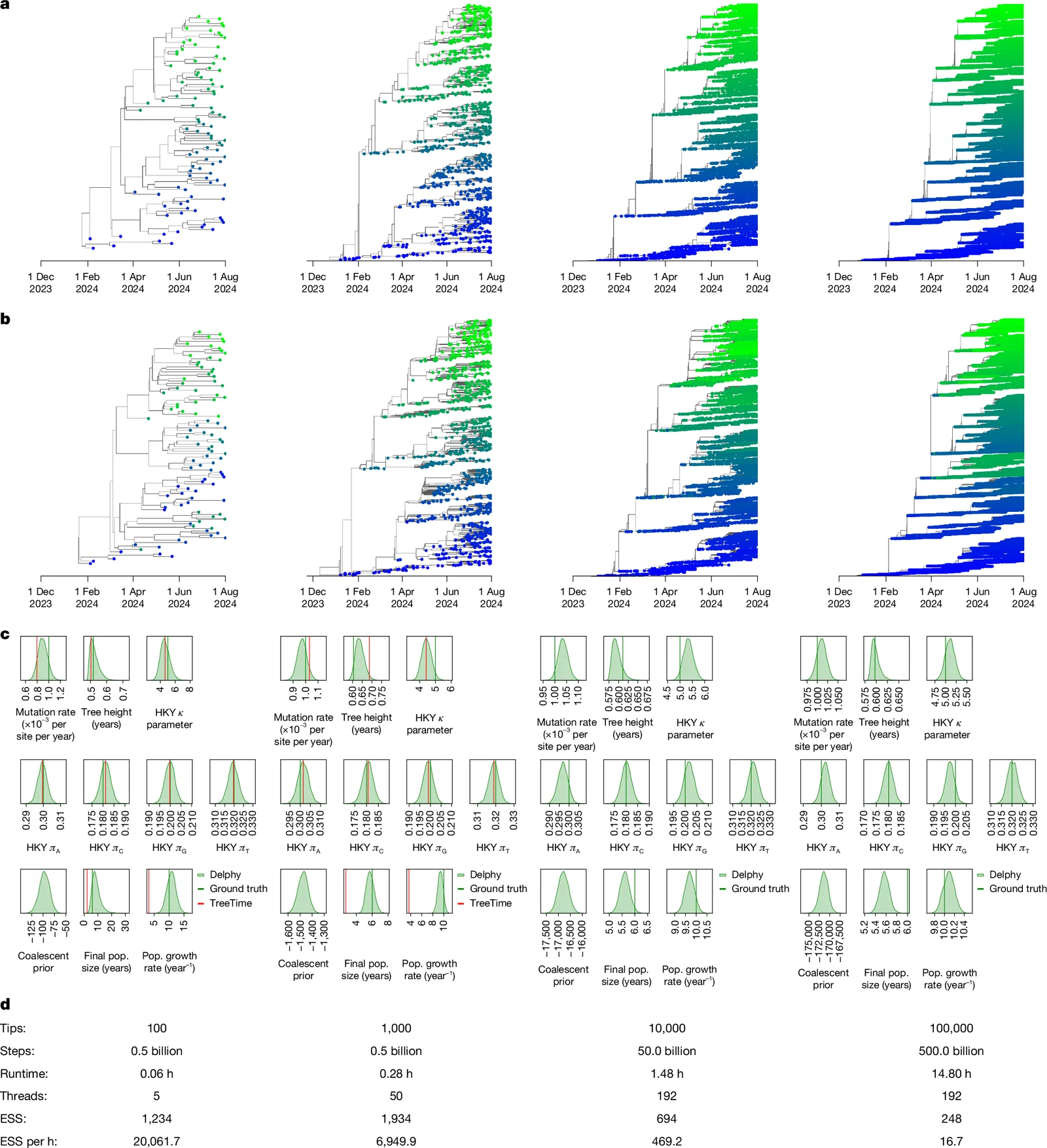
Delphy’s scaling behaviour in the current proof-of-concept implementation. **a**, Simulated phylogenetic trees including 100, 1,000, 10,000 and 100,000 sequences (left to right); tips are coloured blue to green by vertical ordering in the simulated tree (Methods). **b**, MCC trees inferred by Delphy from the simulated tip sequences (corresponding tips in **a** and **b** have the same colour). **c**, Inferred parameter distribution versus known inputs (green) and maximum-likelihood estimates (red, only for 100 and 1,000 tips). **d**, Run times and ESSs (posterior) for each run on a 96-core machine.

We simulated SARS-CoV-2-like outbreaks expanding exponentially over 6 months and containing 100 to 100,000 samples, sampled at a rate proportional to the viral population at the time (Fig. 5a; analogous results in a constant-size population are shown in Supplementary Fig. 8). We constructed their ancestry tree using coalescent simulation and evolved a 30,000-site random root sequence under an HKY model with a mutation rate of 10^−3^ mutations per site per year and a transition–transversion ratio of 5. This process produced *n* = 100 to 100,000 complete, dated sequences with a known true phylogeny.

Delphy’s inferred phylogeny (Fig. 5b) and model parameters (Fig. 5c) closely matched the ground truth. The largest benchmark, with *n* = 100,000 samples, achieved an ESS of 248 in under 15 h on a 96-vCPU Amazon Web Services (AWS) virtual machine. By contrast, the largest Bayesian phylogenetics runs thus far^54^ involved around 40,000 sequences, used fixed topologies with polytomies and took about 3 weeks (personal communication). Thus, even in this early implementation of parallelized MCMC, Delphy processes pandemic-scale data fast enough for real-time public health decision-making, beyond the reach of existing state-of-the-art Bayesian phylogenetic tools.

**Extended Data Fig. 4:**
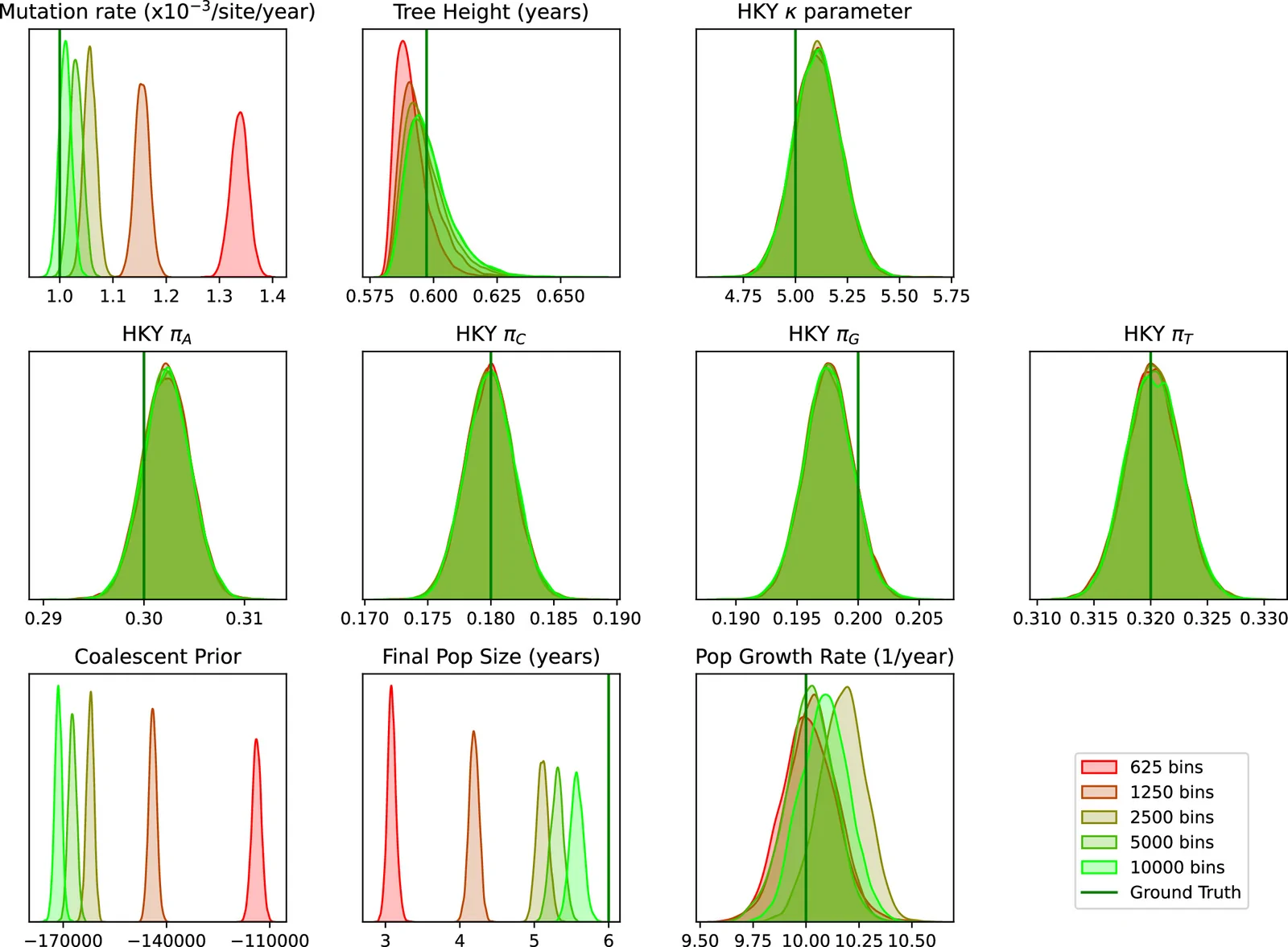
Overall accuracy for N = 100,000 scaling dataset increases as the coalescent prior is discretized more finely. When sampling intensity is so large that the coalescent prior’s k(t) / N(t) factor is comparable to the genome-wide mutation rate, numerical artifacts in the discretized coalescent prior have an appreciable effect. By default, Delphy splits the time range of the tree into about 400 bins to discretize the coalescent prior. Here, we show the effect for the N = 100,000 dataset of using 625, 1,250, 2,500, 5,000 and 10,000 bins. As bin counts increase, the distributions for each observable approach the ground truth values (green vertical bar). In Fig. 5 of the main text, the N = 100,000 dataset used 10,000 bins, while all others used the default 400 bins.

The main challenge in scaling Delphy to hundreds of thousands of sequences or more is numerical inaccuracy in the parallelized coalescent prior when too many branches are active simultaneously. Note the discrepancy between simulated and inferred population size for *n* = 100,000 in Fig. 5c. We have mitigated this discrepancy by increasing the resolution of the underlying numerical discretization, but doing so adds substantial computational cost and reduces ESS per h for large runs (Extended Data Fig. 4). Separately, our parallelization implementation needs further optimization; for example, the 100,000-sample run used less than 25% of the machine’s compute capacity at any given time.

## Discussion

Delphy demonstrates that an exact reformulation in terms of explicit mutations can substantially accelerate Bayesian phylogenetics for public health applications. By structuring every inner-loop step around mutations, Delphy simplifies each operation compared with established Bayesian tools. Yet, owing to their shared Bayesian foundation, Delphy’s results are statistically indistinguishable from those of conventional Bayesian methods, up to an arbitrarily small discretization error in the parallelized coalescent prior. An exact but non-parallelizable coalescent prior, or exact parallelizable ancestry models such as birth–death or epidemic models, would yield absolutely indistinguishable results. Our current implementation can already analyse real outbreak data. However, for thinly sampled data spanning evolutionary timescales, an explicit-mutation approach remains exact but may offer smaller efficiency gains, or even a loss, compared with existing approaches.

Delphy is effective for typical genomic epidemiology datasets largely owing to their low sequence diversity. For example, the 1-million-sequence maximum parsimony tree for SARS-CoV-2 in ref. ^18^ contains about 1 million mutations, averaging just one mutation between each sequence and its nearest neighbour. In such cases, the cost of individual MCMC moves is small (scaling with the number of affected mutations) and the posterior distribution is dominated by nearly parsimonious trees^19,20,55^. While maximum parsimony loses timing information and cannot disambiguate between equally parsimonious trees, Delphy adds sufficient detail about evolutionary dynamics to address both shortcomings. By maintaining a Bayesian framework, rather than simplifying to maximum-likelihood, Delphy preserves accurate phylogenetic uncertainty and enables additional inference, such as phylodynamics, outbreak reconstruction or hypothesis testing. For datasets that are far from parsimonious, locally or globally, Delphy degrades predictably: it remains exact but slows down, rather than silently introducing artifacts.

Delphy’s treatment of missing data may offer a general strategy applicable for other contexts. *N*-pruning transforms a difficult problem with potentially enormous uncertainty into a simpler problem with little uncertainty by avoiding imputation of missing data near tree tips.

Delphy’s mdSPR move (Fig. 1c) enables large-scale rearrangements without fine-tuning; this robustness helps open Bayesian phylogenetics to a much broader audience. mdSPR generalizes UShER’s core step of finding the most parsimonious attachment point for any tip, with three key differences: (1) the global parsimony requirement is relaxed, yielding completely local, scalable moves; (2) continuous rearrangement avoids committing to an early suboptimal tree; and (3) its Bayesian formulation ensures moves can only improve sampling efficiency, not change the sampled distribution. However, mdSPR is not perfect: we suspect that most clade correlation graph outliers reflect sampling difficulties with misplaced tips resulting from rugged tree landscapes^50^ (Supplementary Information 12). Similarly, year-long tip-date uncertainties are problematic for convergence. At present, we recommend corroborating any conclusion that depends on exact sequence placement through multiple independent runs and excluding tips with date uncertainties above 1 year.

Our benchmarks do not show strong evidence that parallelization slows large-scale rearrangements or topology exploration, despite the concern that restricting moves to small subtrees could have this effect. High frechetCorrelationESS values for tree topology support this conclusion (Figs. 2f,k and 5e). We speculate that, for densely sampled datasets, typical trees are dominated by a well-defined ‘backbone’ decorated with more variable offshoots, and that topological moves primarily rearrange these offshoots. A fuller exploration remains for future work.

Delphy demonstrates that Bayesian phylogenetics can be parallelized over the number of sequences, not just genome size, an increasingly important feature as genomic surveillance expands. Our current parallelization implementation is a proof of concept. As EMAT-based phylogenetics is likely not memory bound, and because alternative ancestry models, such as birth–death or epidemiological, may be both more appropriate and simpler to parallelize, systematic optimization should unlock pandemic-scale Bayesian phylogenetics at considerable detail, in the spirit of UShER, matOptimize and MAPLE.

Delphy’s Bayesian foundations can, in principle, support many features and extensions that have accumulated in existing tools^14,15,17^. We have focused on emerging and ongoing viral outbreaks and plan to extend Delphy with other relevant features, including codon effects, more flexible substitution models and non-strict molecular clocks. Investigations requiring the full flexibility of existing tools will remain within their purview for now.

Delphy lowers the barrier to entry for Bayesian phylogenetics. Our web application enables any user to obtain publication-quality Bayesian results in minutes without specialized software or training. Delphy is freely available: its core innovations are formally disclosed and dedicated to the public domain, and the software released under a permissive open-source MIT license. Together, these choices remove barriers to adoption, extension and reuse, supporting broad application of explicit-mutation Bayesian phylogenetics across research, public health and other settings. We hope that this encourages further development and broadens the collective impact of these ideas.

## Methods

For brevity, we summarize the essentials of Delphy’s operation here; full details are provided in the Supplementary Information.

### EMATs

Delphy represents trees internally as a collection of explicitly timed nodes and a reference sequence. Each non-root node points to an earlier parent, and each inner node to two later children. Every instant in the tree has an associated sequence, encoded as successive differences from a reference sequence consisting of *L* states, each in {A,C,G,T}. The root node stores mutations (*a*,*ℓ*,*b*) recording a difference at site *ℓ* between the reference state *a* and the root sequence state *b*; we refer to these mutations as being above the root node. Every other node is annotated with a sequence of mutations from its parent to itself. Each mutation (*a*,*ℓ*,*b*,*t*) records a change in state at time *t* from *a* to *b*. Thus, the sequence at point *x* on the tree is obtained starting with the reference sequence, applying the mutations above the root node to obtain the root sequence, then successively applying in order all mutations on the unique path from the root node to *x*.

Nodes are also annotated with missations, tuples (*a*, *ℓ*) recording that for all tips downstream of this node, but not its parent, the state of site* ℓ* is unknown (missing, ‘N’ in the input); at the parent, the state is *a*. The tree topology, mutational history and tip sequences jointly completely determine all missations. Missations are encoded using two complementary structures: an ordered sequence of disjoint, non-consecutive half-open intervals [*ℓ*_start_,* ℓ*_end_); and a map of sites *ℓ* to states *a* whenever the reference state is not *a*. This representation reflects that missing data typically appears in a few long gaps, and that the site-to-state map is sparse, as the state at site* ℓ* is typically the root state when root-to-tip times are small compared with mutation rates (assuming a reference sequence matching a representative root sequence).

To support uncertain tip dates, each tip has a minimum and maximum time, which coincide when there is no uncertainty.

EMATs have evident consistency requirements for node times, mutation times and identities and missation states (Supplementary Information).

### Posterior distribution

Delphy samples trees ${\mathcal{T}}$ and associated model parameters *θ* using MCMC according to the following (unnormalized) posterior distribution:

1 $$P({\mathcal{T}},\theta )\propto L({\mathcal{T}}\,)\times {\mathcal{G}}({\mathcal{T}}|\theta )\times {\pi }_{\mathrm{anc}}({\mathcal{T}}|\theta )\times {\pi }_{\theta }(\theta )$$

The factors are as follows:

- ${\pi }_{\theta }(\theta )$ is the prior distribution for the model parameters *θ*.
- ${\pi }_{{\rm{a}}{\rm{n}}{\rm{c}}}({\mathcal{T}}\,|\theta )$ is an ancestry prior for the tree’s topology given the model parameters *θ*.
- $G({\mathcal{T}}\,|\theta )$ is a genetic prior for the particular mutational history decorating the EMAT ${\mathcal{T}}$.
- $L({\mathcal{T}}\,)$ is the likelihood of the data given the EMAT ${\mathcal{T}}$; as Delphy currently restricts tip sequences to be either definite (A,C,G,T) or completely missing (N), this likelihood is simply 1 if the EMAT is consistent (see above) or 0 otherwise.

The first two priors are described below. The genetic prior is the probability that a random root sequence evolved with the evolution model parametrized by θ produces the mutational history in EMAT ${\mathcal{T}}$. Explicitly,

2 $${\mathcal{G}}({\mathcal{T}}\,|\theta )=\left\{\prod _{{\ell }\in \xi (r)}{\pi }_{{s}_{r}^{({\ell })}}^{({\ell })}\right\}\times \exp \,\left[-{\int }_{x\in {\mathcal{T}}}\lambda (x){\rm{d}}x\right]\times \prod _{(a,{\ell },b)\in {\mathcal{M}}}{Q}_{ab}^{({\ell })}$$

where:

- ${Q}_{{ab}}^{({\ell })}$ is the rate at which state *a* at site *ℓ* transitions to state *b*, as parametrized by the evolutionary model parameters in *θ*.
- ${Q}_{a}^{({\ell })}=-{Q}_{{aa}}^{({\ell })}$ is the rate at which state *a* at site* ℓ* transitions to any other state.
- ${s}_{x}^{({\ell })}$ is the state of site *ℓ* at point *x* on ${\mathcal{T}}$.
- ξ(x) is the set of sites for which at least one tip below point *x* on ${\mathcal{T}}$ is informative (that is, its state is not missing).
- $\lambda (x)=\sum _{{\ell }\in \xi (x)}{Q}_{{s}_{x}^{({\ell })}}^{({\ell })}$ is the sequence-dependent genome-wide mutation rate at point *x* on ${\mathcal{T}}$ (see the *N*-pruning discussion below).
- ${\int }_{x\in {\mathcal{T}}}f(x){\rm{d}}x$ is an integral of a function f(x) defined at every point on the tree, defined as the sum of time integrals along each branch of ${\mathcal{T}}$.
- ${\pi }_{a}^{({\ell })}$ is the probability that the root sequence has state *a* at site *ℓ*.
- r is the root node of the tree.
- ${\mathcal{M}}$ is the set of all mutations across the tree.

The Supplementary Information further discusses the motivation for these choices, considerations for efficient calculations and its equivalence to the standard tree likelihood based on Felsenstein pruning.

### Parameter and ancestry priors

Delphy’s priors, ${\pi }_{\theta }(\theta )$ and ${\pi }_{{\rm{a}}{\rm{n}}{\rm{c}}}({\mathcal{T}}\,|\theta )$, are currently as follows:

- Tip times are, a priori, uniformly distributed between their minimum and maximum times.
- The transition rate matrices have the form ${Q}_{{ab}}^{({\ell })}=\mu {\nu }^{({\ell })}{q}_{{ab}}$, where *μ* is an overall per-site mutation rate, the quantities {*ν*^(*ℓ*)^} are site-relative rates, and ${q}_{{ab}}$ are the normalized transition rate matrix elements of an HKY evolution model with transition-transversion rate *κ* and stationary state frequencies ${\pi }_{a}$. This form implies a strict molecular clock.
- By default, the mutation rate *μ* has an improper uniform prior; a more general gamma prior can also be applied.
- The site-relative rates {*ν*^(*ℓ*)^} are either fixed to 1 (no site-rate heterogeneity), or have a priori ${\nu }^{({\ell })} \sim \mathrm{Gamma}(\alpha ,\alpha )$, with $\alpha  \sim \mathrm{Expo}(1)$ a priori, where ~ means ‘distributed as’.
- The HKY parameters are chosen such that, a priori, $\{{\pi }_{a}\} \sim \mathrm{Dir}(\mathrm{1,1,1,1})$ and $\log (\kappa ) \sim N(1,{1.25}^{2})$.
- The ancestry prior is one of:

1. A standard coalescent prior with an exponentially growing population curve $N(t)={n}_{0}{{\rm{e}}}^{g(t-{t}_{0})}$, where *t*_0_ is the time of the latest tip. By default, the final effective population size *n*_0_ has an improper 1/*x* prior, while the growth rate *g* has a Laplace prior with mean 0.001 per year and scale 30.701135 per year. More general priors for *n*_0_ and *g* are also available (Supplementary Information).
2. A standard Skygrid flexible population prior^51^, where the log of the population curve is specified parametrically at a fixed number of equally spaced times, and at intermediate times is either piecewise constant but discontinuous (staircase, standard) or piecewise linear and continuous (log-linear, particular to Delphy). A priori, the parametrized log-populations follow a random walk with a diffusion constant that allows the population to change by somewhere between halving and doubling in 1 month. We deviate from the original Skygrid by defaulting to fixing instead of inferring this diffusion constant (equivalently, the precision parameter *τ*), which we find to be both more generally stable and to better encode our intuition of what constitutes reasonable population fluctuations. We also add a penalty for the population curve to assume unrealistically low values (coalescence times below 1 day), which further stabilizes Skygrid for general use. An optional Inverse-Gamma prior can also be applied to the mean population level. Full details are provided in the Supplementary Information.

These choices are suitable for most viral outbreaks, and are currently fixed in Delphy, but their details are not essential. We expect to evolve Delphy to make prior specification more flexible in the future. Most of the above details are the same as the defaults provided by BEAUTi2, and coincide with those used previously^22^. The priors are discussed further in the Supplementary Information.

### MCMC moves

Delphy samples trees and model parameters from the above posterior distribution using MCMC. We distinguish between local moves that affect only a few nodes, and global moves that affect the whole tree.

The following global moves are used (details are provided in the ‘Global moves’ section of the Supplementary Information): Gibbs sampling of mutation rate; delta-exchange moves for stationary frequencies *π*_*a*_ and scale moves for *κ*; scale moves for *α* after integrating out {*ν*^(*ℓ*)^}, followed by Gibbs sampling of {*ν*^(*ℓ*)^}; population parameter moves (exponential model: scale moves on *n*_0_ and random walk on *g*; Skygrid: Gibbs move on overall population size, Hamiltonian Monte Carlo move on log-population sizes and optional Gibbs sampling of *τ*). The relative simplicity of global moves in the explicit-mutation representation, including the marginalization of {*ν*^(*ℓ*)^} for making moves for *α*, was first highlighted in ref. ^37^.

The following local moves are used (details are provided in the ‘Local moves’ section of the Supplementary Information): inner node displacement, branch reform, SPR. For SPR, the regrafting point *P*′ is proposed using an annealed, approximate mdSPR, scanning points up to 1 mutation away from *P* in 99% of cases, the whole tree (partition) in 1% of cases. The mutational history on the *P*′–*X* branch is a Jukes–Cantor history compatible with end-point sequences, implemented to scale with the number of sequence differences, not the genome size. The interaction of SPR moves with *N*-pruning is described in full in the Supplementary Information.

### Missing data: *N*-pruning and missations

Missing data substantially complicates using an explicit-mutation representation. Imputing missing data and inferring full mutational histories can be costly in practice (Supplementary Information 5). *N*-pruning performs Felsenstein pruning only below missations, where the tree likelihood is manifestly 1: the state *a* below a missation (*a*,*ℓ*) evolves to something downstream. This limited Felsenstein pruning preserves the properties of the posterior functional form that Delphy exploits: locality and factorizability; on net, it merely restricts ${\ell }\in \xi (x)$ in the above formulas.

Despite the substantial bookkeeping complications (Supplementary Information 6), one can efficiently update missations on trees as subtrees are pruned and regrafted and mutational histories are changed. Missations enter the proposal and acceptance probabilities of all MCMC moves.

### Parallelizable coalescent

Delphy augments the usual Kingman coalescent ancestry prior to allow for parallelization (full details are provided in Supplementary Information 9). In particular, terms involving *k*(*t*), the number of active lineages at time *t*, which a priori require a global view of the tree not available within a single partition, are replaced by terms involving *k*_*p*_(*t*), the number of active lineages in partition *p* only, and an auxiliary Gaussian coupling field with carefully chosen distribution whose net effect is to recover the Kingman coalescent. For local moves, the Gaussian field is kept fixed, so different *k*_*p*_(*t*)’s can evolve independently. For global moves, when *k*(*t*) is known but static, the coupling field can be Gibbs sampled conditioned on *k*(*t*).

To implement this scheme numerically, the Kingman coalescent integral must be discretized at a user-tuneable resolution, below which *k*(*t*) and *N*(*t*) are approximated as constant. Delphy aims for a discretization with around 400 cells, occasionally changing resolution if the tree height spans too many or too few cells. This default value is often suitable, but can lead to artifacts when there are too many active branches (Extended Data Fig. 4). Note also that there is a subtle interaction between the tree partitioning and correct sampling that must be mitigated in concrete implementations (Supplementary Information 9.1).

While the above scheme is correct and permits parallelization, we expect and encourage better parallelization schemes to be developed.

### Lineage and mutation prevalence curves

Delphy’s interface shows prevalence curves *u*(*t*) for lineages and mutations, equal to the probability that a random member of the population at time *t* is descended from the subtree below a lineage’s founding inner node, or one below a specific mutation. The coalescent model yields a simple differential equation for *u*(*t*), which Delphy solves numerically (full details are provided in Supplementary Information 12). At densely sampled times, where there are many active lineages *k*(*t*), then *u*(*t*) reduces to the fraction of active lineages with a certain property. More generally, *u*(*t*) is an exponentially moving average of that fraction, with decay rate *k*(*t*)*/N*(*t*).

Prevalence curves are calculated for each posterior tree, and the mean and 95% HPD range of each such family of curves is displayed to the user.

### Automatic detection of burn-in cut-off

Delphy implements a simple heuristic for suggesting an MCMC burn-in cut-off. For a given observable, it calculates the mean and s.d. over the second half of the run, finds the latest time that the observable’s fluctuations exceed 5 s.d., then finds the earliest subsequent time that fluctuations fall within 2 s.d. This time is the suggested cut-off for that observable; Delphy takes the maximum suggested cut-off across the log-posterior, mutation rate and total evolutionary time traces. Unless a user overrides this suggestion, the cut-off is continuously updated; typically, it stabilizes once the run is well into production.

This heuristic identifies a point near the end of the initial burn-in, then advances to the earliest subsequent ‘normal’ part of the trace. Empirically, this procedure makes similar choices as a human would. Importantly for Delphy’s accessibility goal, the cut-off suggestion is made automatically: experience with early users showed that requiring manual cut-off selection led to either needless friction or no cut-off at all, biasing the results.

### Delphy input formats

Delphy reads multiple-sequence alignments (MSAs) in FASTA or MAPLE^31^ format. From each description line, a full sequence ID is extracted after the initial ‘>’ up to the end of the line. The full ID consists of fields separated by vertical bars (‘|’): the first field serves as a short ID for the interface and metadata annotation, the last field is a date specification, and other fields are ignored. A date specification can be an exact date (‘2025-01-24’), a month (‘2025-01’), a year (‘2025’) or a date range (‘2025-01-20/2025-01-24’).

Metadata should be a comma-separated value (.csv) or tab-separated value (.tsv) file with a header row. One column should be called “id” or “accession” (case insensitive), with values matching short sequence IDs from the MSA. The remaining columns may have any names and values. Values may be quoted with double-quotes (“), and missing values may be indicated by an empty entry or the values ‘-’, ‘noknown’ or ‘none’ (case insensitive).

Sample MSA and metadata files can be downloaded for the demos on Delphy’s landing page.

### APOBEC3-aware evolution model for mpox

Inspired by previous studies^3,4^, Delphy includes a specialized evolution model suitable for mpox sequences, which can be activated in the web interface under “Advanced Options”. In brief, each site is classified as having or lacking APOBEC3 context: a site with state C or T has APOBEC3 context when preceded by a T, whereas a site with state G or A has APOBEC3 context when followed by an A. We then use a Jukes–Cantor model with rate* μ*, modified in sites with APOBEC3 context so C-to-T and G-to-A mutations occur at a rate *μ* + μ*. This setup retains the essence of the models of refs. ^3,4^ but differs minimally in its details. Full details are provided in the Supplementary Information.

### Benchmarks

All benchmarks are in the GitHub and Zenodo data repositories (Data availability). Each benchmark is organized as a series of numbered scripts. Unless noted, the scripts are self-contained and download external data as needed. The repos also include intermediate and final results files, as well as many of the plots here and in the Supplementary Information (large files are only at Zenodo; and, for GISAID data subject to its data use agreement, we include download instructions but not the actual data). We intend this repository to be executable documentation of every benchmark detail: while all scripts ran correctly at publication, we do not intend to modify them to ensure they continue to run indefinitely.

We used Delphy v.1.1.4 (build 2044, commit a50e378), MAFFT v.7.505 (10 April 2022), BEAST2 v.2.7.7, BEAST X v.10.5.0, Sapling v.0.1.1 (build 2, commit a0b9da1), BEAGLE commit 6480ad3 (Monday, 15 September 2025), IQ-TREE v.2.3.6 and TreeTime v.0.11.4. The sars-cov-2-gisaid-week-by-week and sims benchmarks used Delphy v.1.0 (build 2036, commit 06a7ee4), which lacks a Skygrid population model but is otherwise not materially different. Unless noted, all calculations were run on AWS c7a.2xlarge instances (eight vCPUs), not the web interface (which is 2–3× slower due to WebAssembly). All Delphy runs were performed twice independently, with convergence checked visually using Tracer^56^.

MCCs were calculated using clade fingerprinting (Supplementary Information), implemented in the ‘delphy-mcc’ utility program that is part of Delphy. We verified these match TreeAnnotator2’s output^15^, which hit severe runtime and memory limits on larger benchmarks; by contrast, delphy-mcc processed our 100,000-sequence simulations in minutes. Delphy-mcc applies a 30% burn-in and behaves as if the option ‘--heights ca’ had been given to TreeAnnotator2, so inner node times are the mean tMRCA of the downstream tips over all posterior trees, not just those trees where these tips form a monophyletic clade^57^ (matching the MCC in figure 3a of ref. ^22^).

Sampling speed was assessed by dividing ESSs by the wallclock time. For numerical observables, we used LogAnalyser2 (ref. ^15^) with 30% burn-in (verified to suffice by visual inspection of traces, and higher than necessary to avoid subtleties relating to incomplete filtering of burn-in). For tree topology, we implemented the frechetCorrelationESS measure described previously^58^, which quantifies the rate at which pairwise Robinson–Foulds distances between trees tend to their long-term expected value with increasing separation in the run (using clade fingerprinting, we can calculate frechetCorrelationESS for even the large H5N1 benchmark in seconds; Supplementary Information 11.2). In all cases, ESS values were in the hundreds to thousands; exact values are in the benchmark repository.

Clade correlation graphs between any two runs (Fig. 2e) were computed using a custom program (compare_clades in the data repo), inspired by the analogous CladeSetComparator tool^47^ in BEAST 2 but using clade fingerprints (Supplementary Information). In brief, we first identify all clades appearing in any posterior sample of either run, and filter out any clade with posterior support below 1% in both runs. We then plot for each clade either the posterior support (Support) or the mean clade tMRCA (tMRCAs) in one run versus the other. Error bars show standard errors for posterior support and mean tMRCAs. Dot areas are proportional to the clade size.

#### SARS-CoV-2 data from ref. ^22^ (sars-cov-2-lemieux)

We obtained accession IDs for the 772 samples in figure 3a of ref. ^22^ from the authors (sample_ids.csv), then downloaded the 757 publicly available sequences in GenBank. These were aligned to reference NC_045512.2 (dated to December 2019) using mafft^59^ (--auto–keeplength), then masked the initial 267 and final 230 sites as in ref. ^22^.

Delphy was run twice for 2 billion steps (trace every 100,000 steps, posterior trees every 1,000,000). BEAST X and BEAST 2 were run using Delphy’s ‘equivalent’ XML output without changes for 200 million steps (a rough heuristic: 10 Delphy steps achieve the work of 1 BEAST step). Separate runs were prepared with and without site-rate heterogeneity enabled. Two additional BEAST X runs used *K* = 2 and *K* = 8 discrete gamma categories for site-rate heterogeneity.

An unrooted ML tree was built with IQ-Tree 2 (-m HKY+FO or -m HKY+FO+G4), then rooted and dated with TreeTime, which also estimates population growth rates (--coalescent skyline --n-skyline 2 --stochastic-resolve).

MCCs were plotted using baltic library, with inner node metadata inferred through parsimony (ties resolved arbitrarily).

#### Zika data from ref. ^2^ (zika-metsky-2017)

We extracted the sequences from ref. ^2^ from the BEAST XML file in its supplementary data (SupplementaryData/BEAST input and output/Phylogenetic analyses and model selection/SRD06-strict-exponential.xml). These were aligned to reference KX197192.1, with the initial 107 and final 428 sites trimmed. Runs and analysis otherwise follow the SARS-CoV-2 benchmark above.

#### Ebola data from ref. ^1^ (ebola-gire-2014)

We extracted the sequences from ref. ^1^ from the BEAST XML file in supplementary file 3 (beast/2014_GN.SL_SRD.HKY_strict_ctmc.exp.xml). In this XML file, the raw sequences are partitioned into genic and intergenic regions, scrambling the mapping to reference KJ660346; we manually deduced the inverse mapping to reconstitute an MSA against this reference. Runs and analysis otherwise follow the SARS-CoV-2 benchmark above.

#### Ebola data from ref. ^21^ (ebola-dudas-2017)

We extracted aligned sequences and metadata from ref. ^21^ from the companion GitHub repository (Data/Makona_1610_genomes_2016-06-23.fasta and Data/Makona_1610_metadata_2016-06-23.csv at https://github.com/ebov/space-time.git, commit 9db59a4). Runs and analysis follow the SARS-CoV-2 benchmark above, except with longer runs (Delphy: 10 billion steps; BEAST X: 1 million) and a Skygrid population model having 24 month-long intervals over the 2 years ending at the latest tip (24 October 2015), with a log-space random walk prior having a 6-month halving/doubling time (precision *τ* = 12.3 when effective population sizes are in years).

#### Mpox data from ref. ^3^ (mpox-otoole-2023)

We extracted the sequences from ref. ^3^ from a BEAST XML file in its companion GitHub repository (data/apobec3_2partition.epoch.xml at https://github.com/hmpxv/apobec3, commit c0b4c9b). We removed one non-public GISAID sequence (EPI_ISL_13983888), and two pre-spillover sequences from before 2017 (KJ642617 from 1971 and KJ642615 from 1978), leaving 41 sequences forming the ‘hMPXV-1’ ingroup in figure 3c of ref. ^3^. The XML partitions sites into APOBEC3-context and remaining sites; since every site is marked ‘N’ in at least one partition, the original aligned sequences are trivially reconstituted.

Delphy was run twice for 200 million steps using --v0-mpox-hack, with trace output every 20,000 steps and posterior trees every 200,000 steps. MCCs and ESSs were calculated as described above.

#### Mpox data from ref. ^4^ (mpox-parker-2025)

We extracted the sequences from ref. ^4^ from a BEAST XML file in its companion GitHub repository (BEAST/Mpox_2epoch_combinedDTA.xml.zip at https://github.com/andersen-lab/Mpox_West_Africa, commit 2b481da). We removed three non-public GISAID sequences (EPIISL-13953610, EPIISL-13983888 and EPIISL-15008577), and all pre-spillover/pre-2017 sequences, leaving 177 sequences forming the hMPXV-1 clade in ref. ^4^. As above, the XML partitions sites into APOBEC3-context and remaining, so original sequences are trivially reconstituted.

Delphy was run twice for 1 billion steps using --v0-mpox-hack, with trace output every 100,000 steps and posterior trees every 1,000,000 steps. MCCs and ESSs were calculated as described above.

For BEAST comparisons, we modified the original BEAST X XML (BEAST/Mpox_2epoch_combinedDTA.xml.zip) to match Delphy’s sequences and removed phylogeography, spillover detection and pre/post-spillover partitioning. We shortened the chain to 50 million steps, sufficient for convergence (posterior ESS = 374). The script to modify this input file and the resulting XML are in our data repository (mpox-parker-2025-beast.xml).

#### SARS-CoV-2 data from GISAID submitted/collected by each CDC week (sars-cov-2-gisaid-week-by-week)

We downloaded all SARS-CoV-2 sequences from GISAID collected on or before 31 March 2020 with metadata, filtering out non-human hosts, sequences shorter than 20,000 bases, or with uncertain dates. Each Fig. 5 CDC Epiweek panel includes sequences with the submission date in or before that week (filtered to those submitted in 1 December to 28 March 2020, which is the end of CDC Epiweek 2020-13, and collected from 1 December 2019). For Supplementary Fig. 7, we used collection date instead of submission date (and filtered sequences to those submitted in 1 December 2019 to 31 December 2024, and collected from 1 December 2019). Matching sequences were aligned to NC_045512.2 using mafft, then masked at the initial 268 and final 230 sites (as described previously^22^). No further masking was applied.

Quick initial diagnostic runs revealed clear outlier sequences (for example, inducing a tMRCA to early 2019, lying in an isolated long branch with tens to hundreds of mutations, collection dates far preceding first reported cases in a particular region). After several rounds of iterative refinement by outlier removal, no trees contained obvious outliers. We verified that most offending sequences had been identified as outliers near the beginning of the pandemic, marked as under investigation in GISAID, or appeared in NextStrain or sarscov2phylo exclusion lists. See Supplementary Tables 1 and 2 for the 94 excluded sequences.

Delphy was run on AWS c7a.4xlarge instances (16 vCPUs) for each Epiweek as in the SARS-CoV-2 benchmark, with the following differences: 5 million steps per tip, 10,000 trace and 200 tree samples and at most 1 thread per 100 tips (maximum 32 threads).

#### Simulated trees for scaling assessment (sims)

We prepared two groups of four SARS-CoV-2-like simulated datasets with *N* = 100, 1,000, 10,000 and 100,000 tips. The first group used an exponentially growing population curve, $N(t)={n}_{0}\,{e}^{g(t-{t}_{0})}$ with *n*_0_ = 6 years, *g* = 10 per year and *t*_0_ = 31 July 2024. The second used a constant population *N*(*t*) = *n*_0_ = 2 years. Sample times were drawn from [1 January 2024, 31 July 2024] proportional to *N*(*t*), mimicking a uniform sampling of members of the historical viral population, then linked through a standard coalescent simulation. A random 30,000-site root sequence was sampled with *π* = [0.30, 0.18, 0.20, 0.32] and evolved along the ancestry using the Gillespie algorithm under a site-homogeneous HKY model (mutation rate, 1 × 10^−3^ per site per year, *κ* = 5). Trees were recorded in Newick format, summaries in JSON, and dated tip sequences as FASTA (*N* ≤ 1,000) and MAPLE files. To perform these simulations efficiently, we wrote a tool called sapling (Code availability, Data availability and Supplementary Information 15.2).

Delphy was run twice on a 96-vCPU c7a.24xlarge AWS instance with 5 million steps per tip, 10,000 log and 200 tree samples and at most 1 thread per 20 tips (maximum 192 threads). The *n* = 100,000 run also used around 10,000 cells for discretizing the coalescent prior instead of the default about 400 (comparisons at 625–5,000 cells are shown in Extended Data Fig. 4).

#### H5N1 in cattle dataset (h5n1-andersen-2025)

We cloned the Andersen laboratories avian-influenza repository from GitHub (https://github.com/andersen-lab/avian-influenza) at commit e756a15 (3 October 2025). From 11,541 unique SRRs, we filtered to the 4,080 non-retracted SRRs with a cattle host and B3.13 genotype. For each of H5N1’s 8 segments, we aligned to reference A/cattle/Texas/24-008749-003/2024 (SRR28752635), as found in ‘avian-influenza/reference’, using mafft, then concatenated the segments longest-to-shortest into a single sequence per sample (assuming no appreciable reassortment, which appears valid as of October 2025).

For dating, we identified the 3,339 sequences with GenBank accessions providing day-resolved dates (SRR metadata dates typically only indicate year). We prepared these as input to Delphy; runs with the larger set that includes those with uncertain dates exhibited severe convergence problems and were excluded. For geographical analysis, we used GenBank’s ‘geo_loc_name’, which resolves to a US state in 3,194 of 3,383 accessions.

Delphy was run twice for 20 billion steps (trace every 2 million steps, trees every 20 million). We applied a Skygrid model with 22 month-long intervals ending at the latest tip (5 August 2025), with a log-space random walk prior with 3 month halving/doubling time (precision *τ* = 6.15 when effective population sizes are in years). Coalescent prior discretization was increased to 1,000 cells to reduce discretization error.

### Reporting summary

Further information on research design is available in the Nature Portfolio Reporting Summary linked to this article.

## Online content

Any methods, additional references, Nature Portfolio reporting summaries, source data, extended data, supplementary information, acknowledgements, peer review information; details of author contributions and competing interests; and statements of data and code availability are available at https://doi.org/10.1038/s41586-026-11012-6.

## Supplementary information

Supplementary Information Supplementary Figs. 1–8: benchmark of Delphy on additional datasets and models, including analyses without site-rate heterogeneity, comparisons using collection versus submission dates, and constant-population scaling experiments. Supplementary Tables 1 and 2: excluded GISAID SARS-CoV-2 sequences and the rationale for their exclusion. Supplementary Methods (Sections 2–15): detailed descriptions of the models, MCMC implementation, parallelization, clade fingerprinting, sampling challenges, and calibration studies based on simulations; these sections also include Supplementary Figs. 9–24. Supplementary Tables 4 and 5: summaries of key notation for EMATs, and tree and parameter distributions.

Reporting Summary

Peer Review File

## Data Availability

Data, raw outputs and scripts for generating the figures in this paper are available at Zenodo^60^ (https://doi.org/10.5281/zenodo.17899935). This resource archives the GitHub repository (https://github.com/broadinstitute/delphy-2026-paper-data), and includes large data files that cannot be distributed there owing to GitHub size restrictions.

## Code availability

Delphy is open-source and distributed under an MIT license. The code for its computational core, precompiled x86 and arm64 binaries, and prebuilt Docker images are all available at GitHub (https://github.com/broadinstitute/delphy; permanently archived at Zenodo^61^, https://doi.org/10.5281/zenodo.11922813). Its web interface is hosted at https://delphy.bio, with source code available at GitHub (https://github.com/fathominfo/delphy-web; permanently archived at Zenodo^62^, https://doi.org/10.5281/zenodo.11998924); the code includes instructions for local deployment, which allows the use of historical versions for reproducibility. The ‘Delphy’ format is described at https://github.com/broadinstitute/delphy/blob/main/doc/dphy_file_format.md. Sapling, the special-purpose program that we developed to efficiently simulate very large trees, is available at GitHub (https://github.com/broadinstitute/sapling; permanently archived at Zenodo^63^, https://doi.org/10.5281/zenodo.20717608). In the latest stages, development of Delphy, Sapling and the analysis scripts for this paper were assisted by AI (Claude Code); all AI-generated code was carefully reviewed by the authors. Ready-to-use Google Colab tutorials for downloading and formatting sequencing data from the NCBI Virus database, as well as user-provided data, are available online (https://colab.research.google.com/github/broadinstitute/delphy/blob/main/tutorials/delphy_workflow.ipynb (blank notebook) and https://colab.research.google.com/github/broadinstitute/delphy/blob/main/tutorials/delphy_workflow_example.ipynb (example tutorial)); both notebooks are also part of the Delphy repository. These notebooks streamline the uniform formatting of sequencing data and the associated metadata for further analysis with Delphy, and then run Delphy using a pre-compiled binary. The output files can be visualized on the Delphy web interface.
