# Supplementary material for: Scalable near-real-time Bayesian phylogenetics for outbreaks with Delphy
Source: Nature. 2026 Sep 16;658(8136):747–56. doi: 10.1038/s41586-026-11012-6 (PMC13668698; doi:10.1038/s41586-026-11012-6)
Supplement: Supplementary file 1 [file 41586_2026_11012_MOESM1_ESM.pdf]

nature portfolio

https://doi.org/10.1038/s41586-026-11012-6

Supplementary information

Scalable near-real-time Bayesian phylogenetics for outbreaks with Delphy

In the format provided by the authors and unedited

Supplementary Information for "Scalable near-real-time Bayesian phylogenetics for outbreaks with Delphy"

Patrick Varilly[1,*], Mark Schifferli[2], Katherine Yang[2], Paul Cronan[2], Ivan Specht[1,3], Tim Burcham[2], Olivia Glennon[2], Olivia Jacks[2], Ellory Laning[2], Libby Marrs[2], Kyle Oba[2], Shannon Yeung[2], Karlie Wenran Zhao[2], Edyth Parker[4], Ifeanyi Omah[5,6], Jonathan E. Pekar[5], Laura Luebbert[7,8,9], Kristian G. Andersen[10,11], Daniel J. Park[1], Stephen F. Schaffner[1,12], Bronwyn L. MacInnis[1,12,13], Christian Happi[4,12,14], Jacob E. Lemieux[1,15], Al Ozonoff[1,16], Michael Mitzenmacher[17], Ben Fry[2,19], Pardis C. Sabeti[1,8,12,13,18,19,*]

[1]Broad Institute of Harvard and MIT, Cambridge, MA, USA
[2]Fathom Information Design, Boston, MA, USA
[3]Institute for Computational and Mathematical Engineering, School of Engineering, Stanford University, Stanford, CA, USA
[4]The Institute of Genomics and Global Health, Redeemer's University, Ede, Nigeria
[5]Institute of Ecology and Evolution, University of Edinburgh, Edinburgh, UK
[6]Department of Parasitology and Entomology, Nnamdi Azikiwe University, Awka, Nigeria
[7]Eric and Wendy Schmidt Center, Broad Institute of Harvard and MIT, Cambridge, MA, USA
[8]Department of Organismic and Evolutionary Biology, Faculty of Arts and Sciences, Harvard University, Cambridge, MA, USA
[9]FutureHouse, San Francisco, CA, USA
[10]Department of Immunology and Microbiology, The Scripps Research Institute, La Jolla, CA, USA
[11]Scripps Research Translational Institute, La Jolla, CA, USA
[12]Department of Immunology and Infectious Diseases, Harvard T.H. Chan School of Public Health, Harvard University, Boston, MA, USA
[13]Massachusetts Consortium on Pathogen Readiness, Harvard Medical School, Harvard University, Boston, MA, USA
[14]Department of Biological Sciences, Faculty of Natural Sciences, Redeemer's University, Ede, Nigeria
[15]Department of Medicine, Massachusetts General Hospital, Harvard Medical School, Boston, MA, USA
[16]Department of Pediatrics, Harvard Medical School, Boston, MA, USA
[17]Department of Computer Science, School of Engineering and Applied Sciences, Harvard University, Boston, MA, USA
[18]Howard Hughes Medical Institute, Chevy Chase, MD, USA
[19] These authors jointly supervised this work: Ben Fry, Pardis C. Sabeti.
[*] e-mail: `pvarilly@broadinstitute.org`; `pardis@broadinstitute.org`.

August 28, 2026

1 Supplementary Figures and Tables

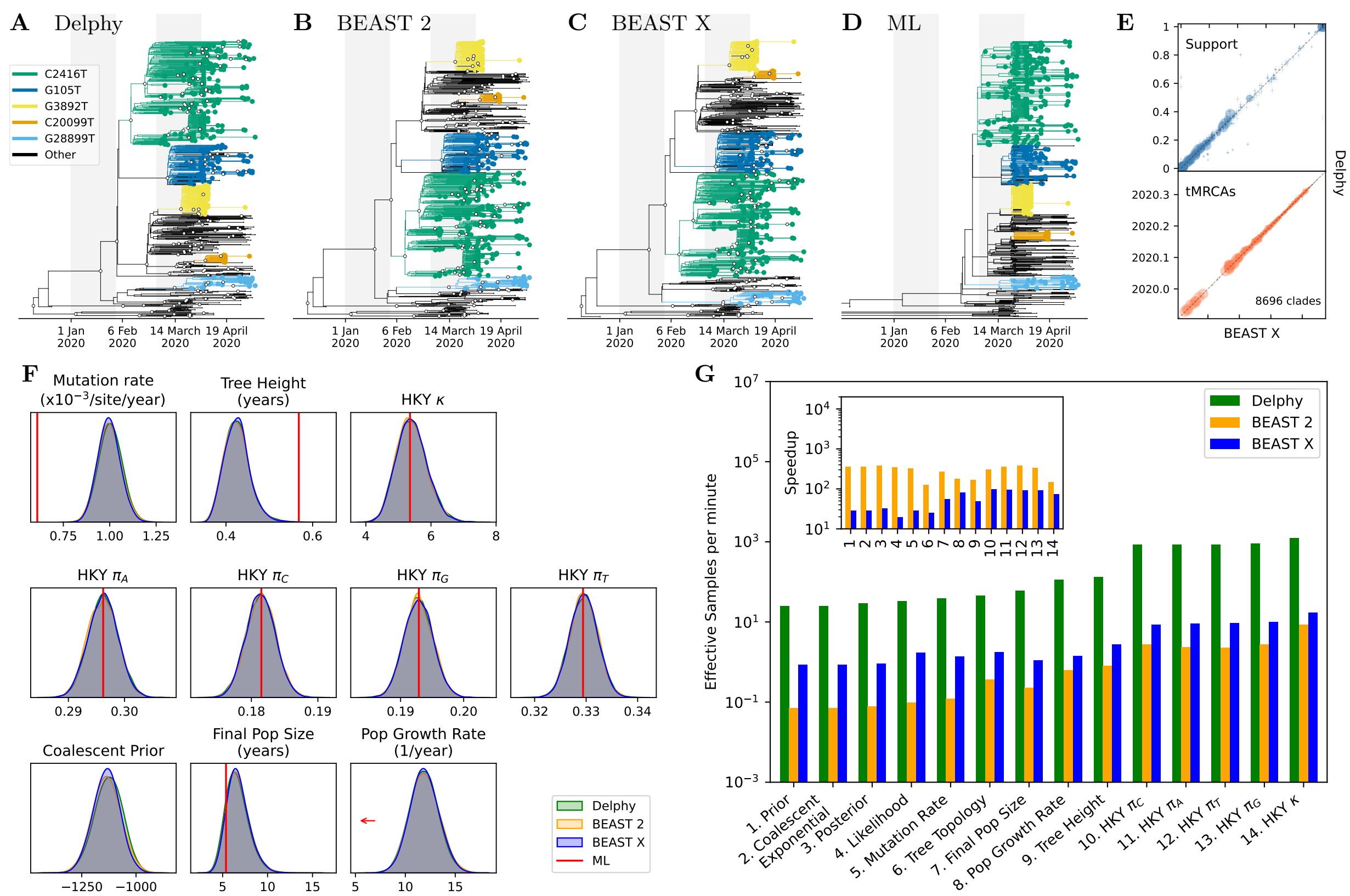

Supplementary Figure 1: Comparison of Delphy, BEAST 2, BEAST X and ML for the SARS-CoV-2 dataset with no site-rate heterogeneity (compare to Figure 2 in the main text). A. Maximum-Clade-Credibility (MCC) as produced by Delphy. **B.** Analog for BEAST 2. **C.** Analog for BEAST X. **D.** Maximum-likelihood timed tree, as calculated by IQ-Tree 2 and TreeTime. **E.** Clade correlation graphs comparing Delphy to BEAST X (comparison to BEAST 2 is similar). **F.** Distributions of key observables, as produced by Delphy (green), BEAST 2 (orange), BEAST X (blue), and maximum-likelihood estimate (red). **G.** Runtime efficiency of Delphy vs BEAST 2 and BEAST X.

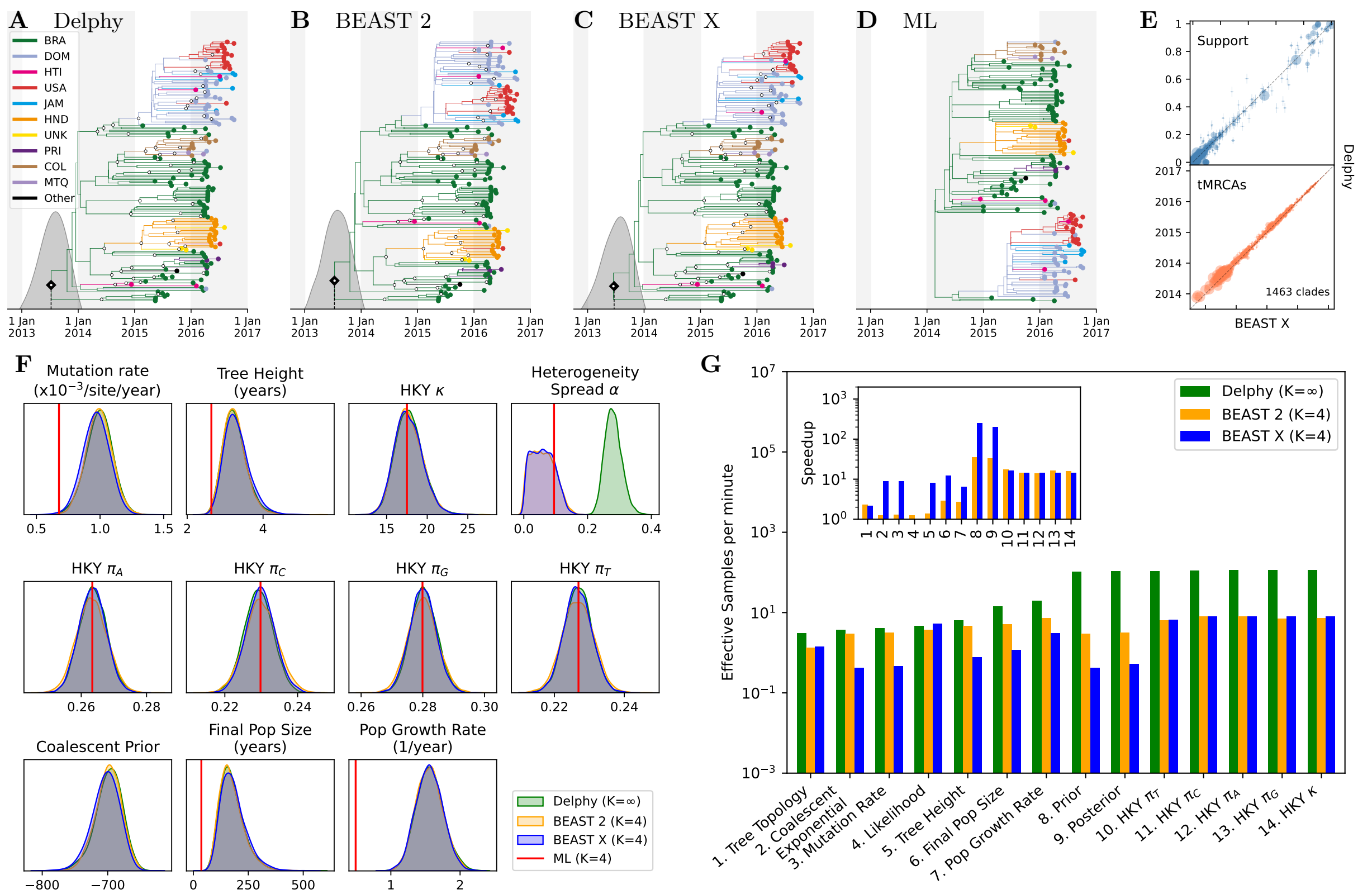

Supplementary Figure 2: Comparison of Delphy, BEAST 2, BEAST X and ML for the Zika dataset with site-rate heterogeneity (compare to Figure 2 in the main text). A. Maximum-Clade-Credibility (MCC) as produced by Delphy. **B.** Analog for BEAST 2. **C.** Analog for BEAST X. **D.** Maximum-likelihood timed tree, as calculated by IQ-Tree 2 and TreeTime. **E.** Clade correlation graphs comparing Delphy to BEAST X (comparison to BEAST 2 is similar). **F.** Distributions of key observables, as produced by Delphy (green), BEAST 2 (orange), BEAST X (blue), and maximum-likelihood estimate (red). **G.** Runtime efficiency of Delphy vs BEAST 2 and BEAST X.

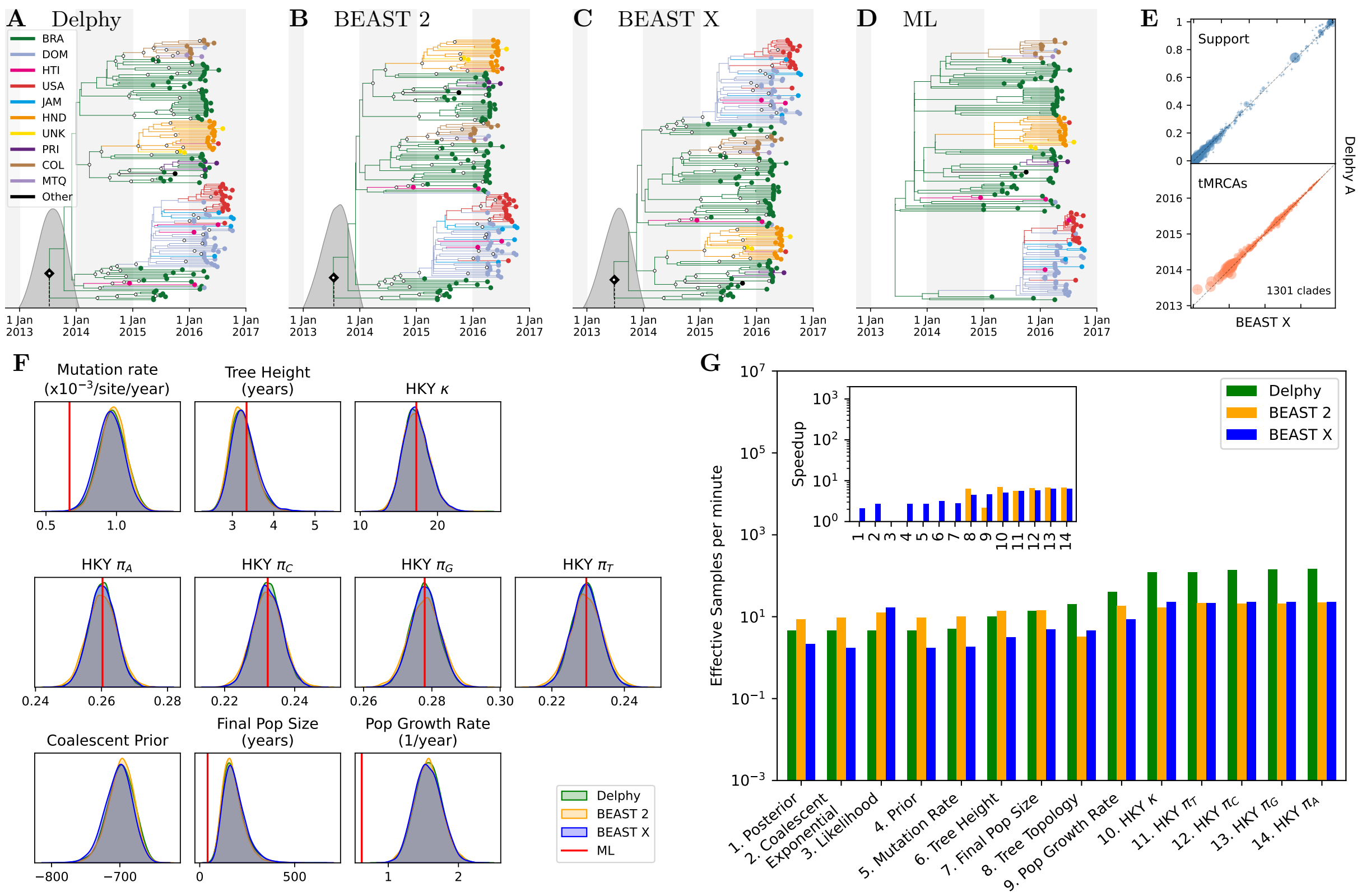

Supplementary Figure 3: Comparison of Delphy, BEAST 2, BEAST X and ML for the Zika dataset without site-rate heterogeneity (compare to Figure 2 in the main text). A. Maximum-Clade-Credibility (MCC) as produced by Delphy. **B.** Analog for BEAST 2. **C.** Analog for BEAST X. **D.** Maximum-likelihood timed tree, as calculated by IQ-Tree 2 and TreeTime. **E.** Clade correlation graphs comparing Delphy to BEAST X (comparison to BEAST 2 is similar). **F.** Distributions of key observables, as produced by Delphy (green), BEAST 2 (orange), BEAST X (blue), and maximum-likelihood estimate (red). **G.** Runtime efficiency of Delphy vs BEAST 2 and BEAST X.

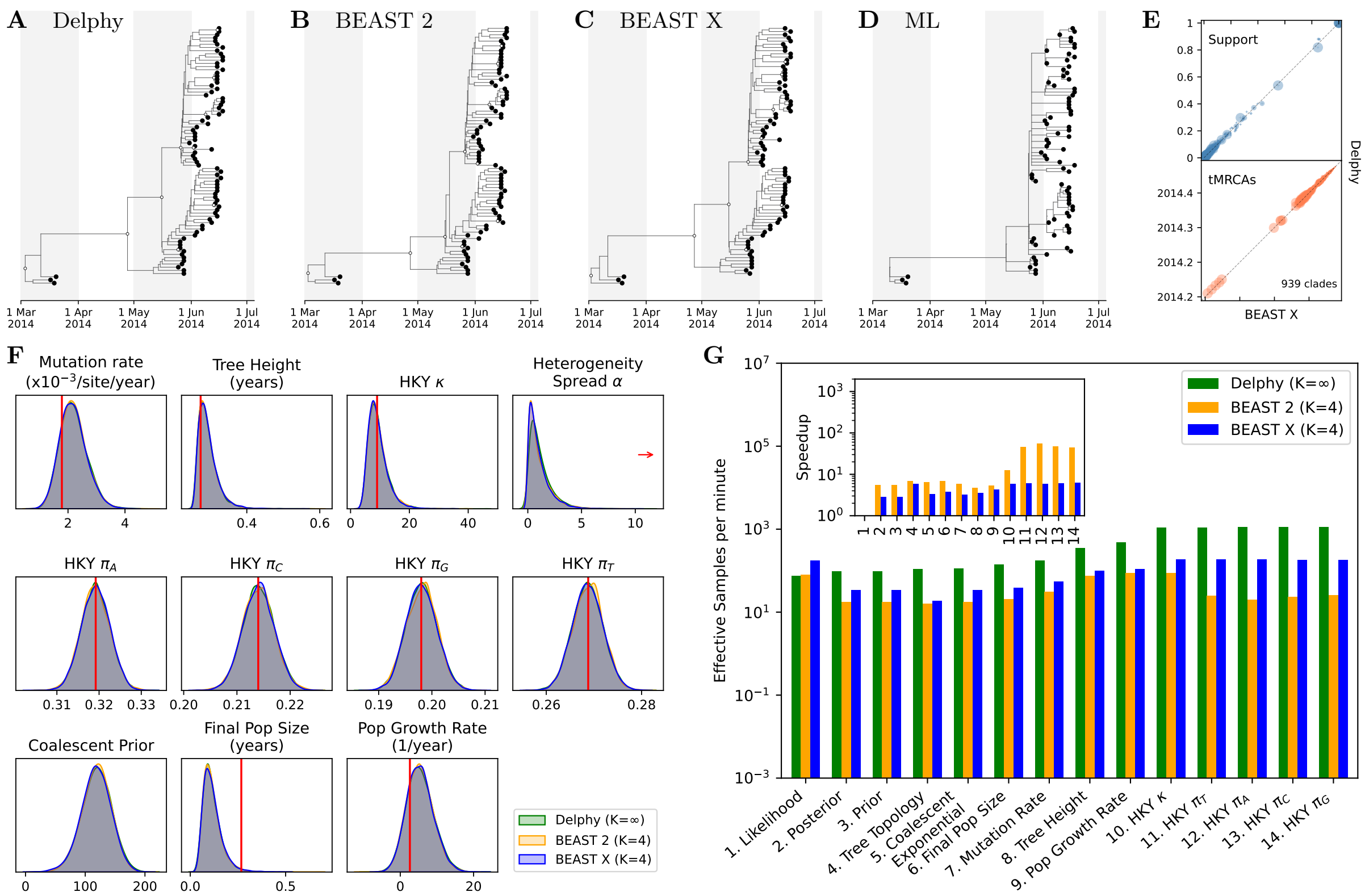

Supplementary Figure 4: Comparison of Delphy, BEAST 2, BEAST X and ML for the small Ebola dataset with site-rate heterogeneity (compare to Figure 2 in the main text). A. Maximum-Clade-Credibility (MCC) as produced by Delphy. **B.** Analog for BEAST 2. **C.** Analog for BEAST X. **D.** Maximum-likelihood timed tree, as calculated by IQ-Tree 2 and TreeTime. **E.** Clade correlation graphs comparing Delphy to BEAST X (comparison to BEAST 2 is similar). **F.** Distributions of key observables, as produced by Delphy (green), BEAST 2 (orange), BEAST X (blue), and maximum-likelihood estimate (red). **G.** Runtime efficiency of Delphy vs BEAST 2 and BEAST X.

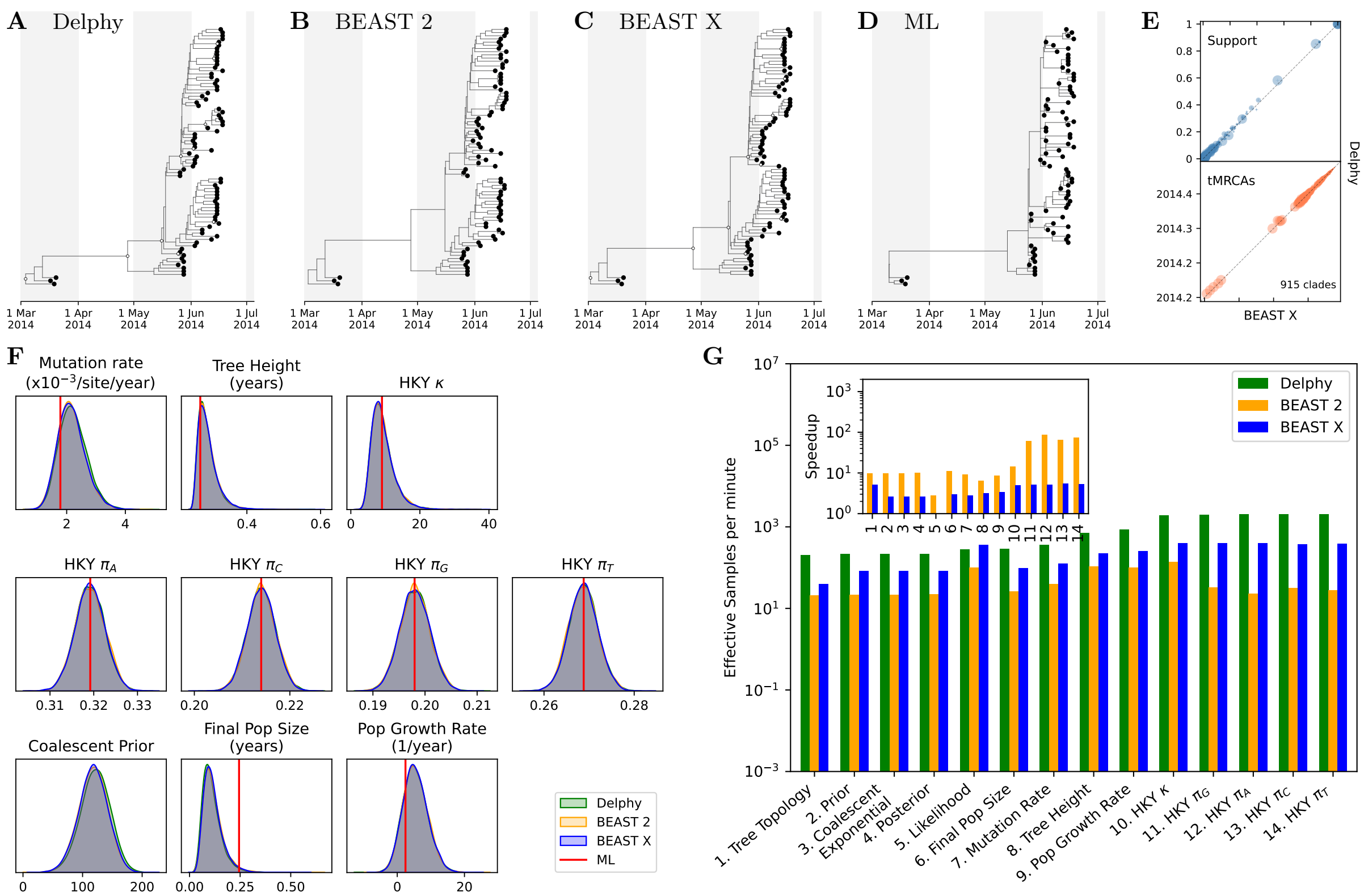

Supplementary Figure 5: Comparison of Delphy, BEAST 2, BEAST X and ML for the small Ebola dataset without site-rate heterogeneity (compare to Figure 2 in the main text). A. Maximum-Clade-Credibility (MCC) as produced by Delphy. **B.** Analog for BEAST 2. **C.** Analog for BEAST X. **D.** Maximum-likelihood timed tree, as calculated by IQ-Tree 2 and TreeTime. **E.** Clade correlation graphs comparing Delphy to BEAST X (comparison to BEAST 2 is similar). **F.** Distributions of key observables, as produced by Delphy (green), BEAST 2 (orange), BEAST X (blue), and maximum-likelihood estimate (red). **G.** Runtime efficiency of Delphy vs BEAST 2 and BEAST X.

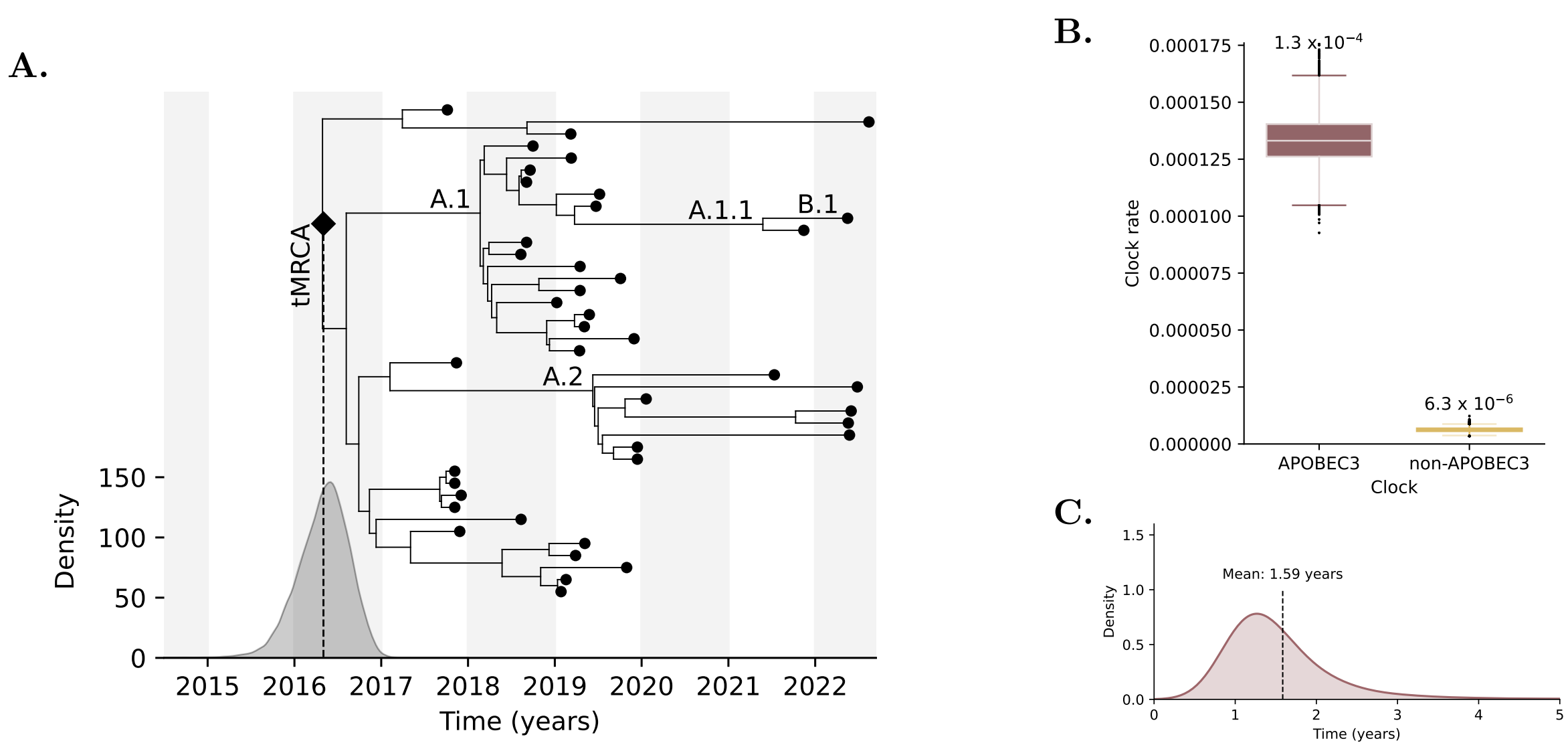

Supplementary Figure 6: Delphy applied to the mpox dataset from O'Toole et al 2023 [29]. **A.** MCC for 41 hMPXV-1 samples from [29] as produced by Delphy (compare to Figure 3C in [29]). **B.** Inferred mutation rates owing to APOBEC3 and non-APOBEC3 mechanisms (compare to Figure S17A of [29], where the APOBEC3 rate is 1.2×10^{-4} per site per year and the non-APOBEC3 rate is 4.2×10^{-6} per site per year). **C.** Inferred doubling time under exponential growth model (compare to Figure S17B of [29]], with a reported mean of ~ 1.5 years).

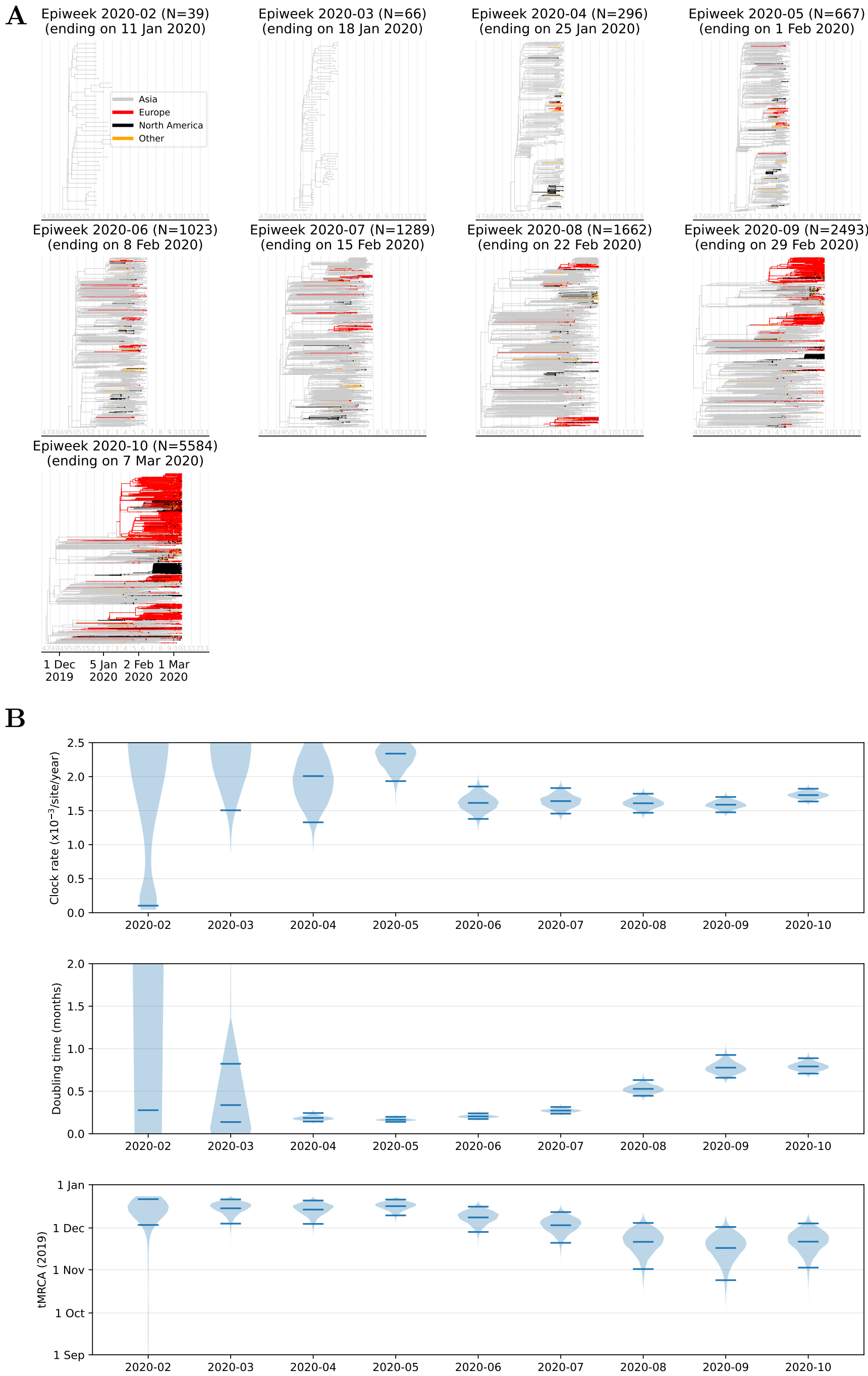

Supplementary Figure 7: Delphy's view of the first weeks of the COVID-19 pandemic. Analogous to Extended Data Figure 3 in the main text, but including all sequences *collected* by the end of each CDC epiweek, not just submitted. Ends at CDC epiweek 202010 since the number of collected sequences explodes beyond that point. We suspect that sequences collected early on but only sequenced much later are of systematically lower quality, as reflected in qualitatively poorer trees and parameter distributions.

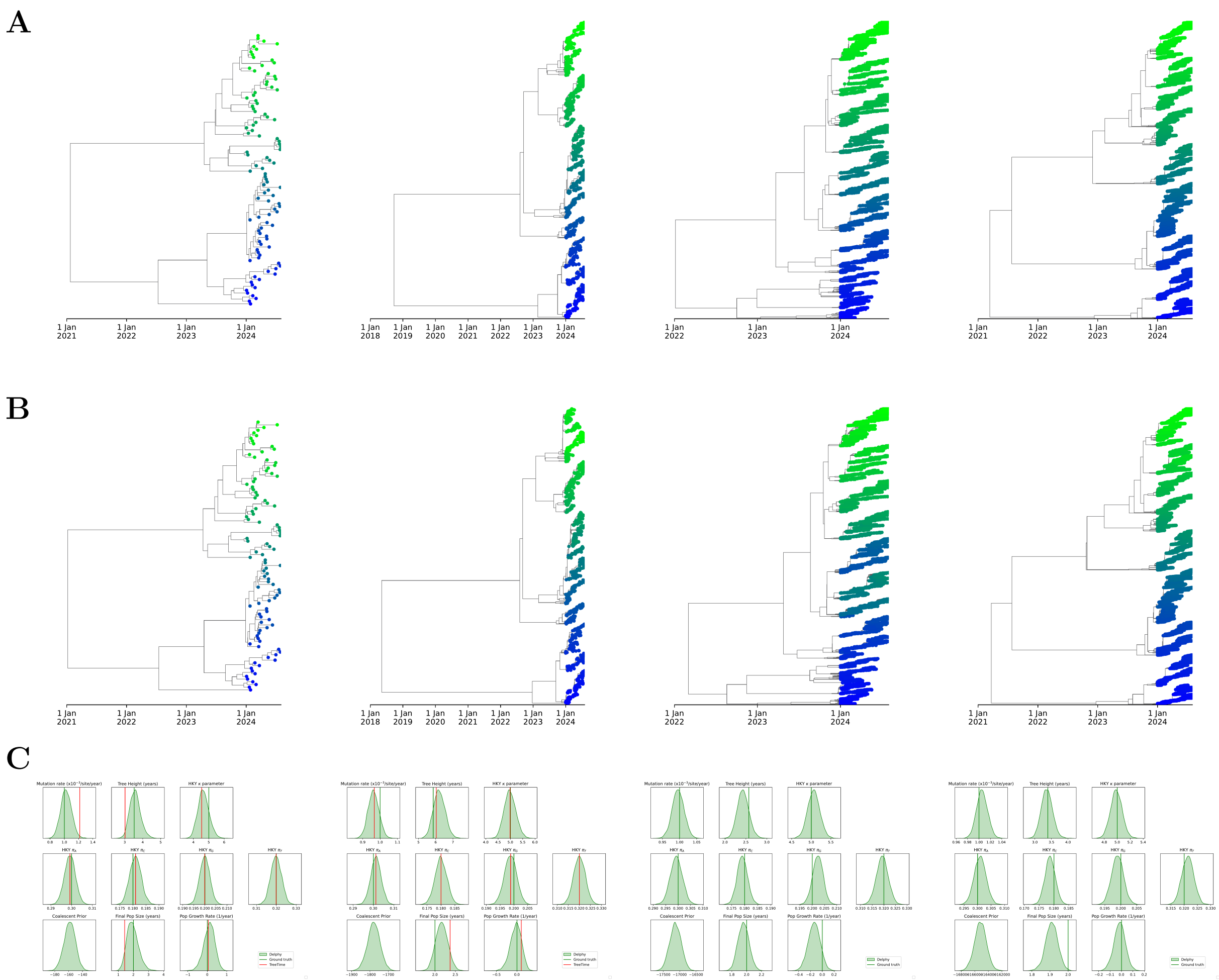

D

Tips:	100	1000	10,000	100,000
Steps	0.5 bn	5.0 bn	50.0 bn	500.0 bn
Runtime	0.09 h	0.35 h	1.50 h	13.42 h
Threads	5	50	192	192
ESS	634	780	1365	380
ESS / hr	6860.5	2211.9	909.0	28.3

Supplementary Figure 8: Delphy's scaling behavior, simulations with constant-size viral populations. Analogous to Figure 6 in the main text, but with a constant effective viral population size of 2 years. As in Figure 6 of the main text, the $N = 100\,000$ dataset used $10\,000$ bins to discretize the coalescent prior as in Eq. (57), while all others used the default 400 bins.

Accession ID	Name	First anomalous run	Name in Nextstrain	Date excluded in Nextstrain	Notes
EPI_ISL_406592	hCoV-19/Guangdong/SZTH-001/2020	Sub. by CDC-202005	Shenzhen/SZTH-001/2020	2020-02-17	1,2,5,6
EPI_ISL_408483	hCoV-19/Shanghai/IVDC-SH-001/2020	Sub. by CDC-202007	Shanghai/IVDC-SH-001/2020	2020-02-09	2,5,6,7
EPI_ISL_408487	hCoV-19/Henan/IVDC-HeN-002/2020	Sub. by CDC-202007	Henan/IVDC-HeN-002/2020	2020-02-09	2,5,6,7
EPI_ISL_412900	hCoV-19/Wuhan/HBCDC-HB-04/2019	Sub. by CDC-202009	Wuhan/HBCDC-HB-04/2019	2020-06-08	2,5,6,7
EPI_ISL_406595	hCoV-19/Guangdong/SZTH-004/2020	Sub. by CDC-202010	Shenzhen/SZTH-004/2020	2020-02-17	1,2,5,6
EPI_ISL_416030	hCoV-19/Saudi Arabia/CV1249/2020	Sub. by CDC-202011	—	—	7
EPI_ISL_414935	hCoV-19/Shandong/LY002-2/2020	Sub. by CDC-202011	—	—	5,7
EPI_ISL_527871	hCoV-19/Yunnan/KMS1/2020	Sub. by CDC-202011	Yunnan/KMS1/2020	2021-01-09	6,7
EPI_ISL_414934	hCoV-19/Shandong/LY001-2/2020	Sub. by CDC-202011	Shandong/LY001-2/2020	2021-04-03	5,6,7
EPI_ISL_415593	hCoV-19/USA/WA-UW65/2020	Sub. by CDC-202011	USA/WA-UW65/2020	2020-06-08	5,6,7
EPI_ISL_417919	hCoV-19/Malaysia/186197/2020	Sub. by CDC-202012	Malaysia/186197/2020	2020-06-08	5,7
EPI_ISL_417413	hCoV-19/Turkey/6224-Ankara1034/2020	Sub. by CDC-202012	Turkey/6224-Ankara1034/2020	2020-06-08	5,7
EPI_ISL_8311702	hCoV-19/Lebanon/LAU-R107/2020	Col. by CDC-202003	—	—	8
EPI_ISL_8311703	hCoV-19/Lebanon/LAU-R126/2020	Col. by CDC-202003	Lebanon/LAU-R126/2020	2023-06-09	6,8
EPI_ISL_8311704	hCoV-19/Lebanon/LAU-R128/2020	Col. by CDC-202003	Lebanon/LAU-R128/2020	2022-09-18	6,8
EPI_ISL_8311705	hCoV-19/Lebanon/LAU-R130/2020	Col. by CDC-202003	Lebanon/LAU-R130/2020	2022-09-18	6,8
EPI_ISL_8311706	hCoV-19/Lebanon/LAU-R135/2020	Col. by CDC-202003	Lebanon/LAU-R135/2020	2022-08-23	6,8
EPI_ISL_8311707	hCoV-19/Lebanon/LAU-R137/2020	Col. by CDC-202003	Lebanon/LAU-R137/2020	2022-09-18	6,8
EPI_ISL_8311708	hCoV-19/Lebanon/LAU-R150/2020	Col. by CDC-202003	Lebanon/LAU-R150/2020	2022-06-21	6,8
EPI_ISL_8311709	hCoV-19/Lebanon/LAU-R158/2020	Col. by CDC-202003	Lebanon/LAU-R158/2020	2022-09-18	6,8
EPI_ISL_8311711	hCoV-19/Lebanon/LAU-R178/2020	Col. by CDC-202003	Lebanon/LAU-R178/2020	2023-06-09	6,8
EPI_ISL_8311729	hCoV-19/Lebanon/LAU-R289/2020	Col. by CDC-202003	Lebanon/LAU-R289/2020	2022-09-18	6,8
EPI_ISL_8311730	hCoV-19/Lebanon/LAU-R293/2020	Col. by CDC-202003	Lebanon/LAU-R293/2020	2022-09-18	6,8
EPI_ISL_8311731	hCoV-19/Lebanon/LAU-R294/2020	Col. by CDC-202003	Lebanon/LAU-R294/2020	2022-10-24	6,8
EPI_ISL_8311733	hCoV-19/Lebanon/LAU-R328/2020	Col. by CDC-202003	Lebanon/LAU-R328/2020	2022-10-06	6,8
EPI_ISL_8311734	hCoV-19/Lebanon/LAU-R330/2020	Col. by CDC-202003	Lebanon/LAU-R330/2020	2022-09-18	6,8
EPI_ISL_8311741	hCoV-19/Lebanon/LAU-R350/2020	Col. by CDC-202003	Lebanon/LAU-R350/2020	2023-06-17	6,8
EPI_ISL_8311755	hCoV-19/Lebanon/LAU-R84/2020	Col. by CDC-202003	Lebanon/LAU-R84/2020	2022-09-18	6,8
EPI_ISL_8311756	hCoV-19/Lebanon/LAU-R89/2020	Col. by CDC-202003	Lebanon/LAU-R89/2020	2022-09-18	6,8
EPI_ISL_8311758	hCoV-19/Lebanon/LAU-R97/2020	Col. by CDC-202003	Lebanon/LAU-R97/2020	2022-09-18	6,8
EPI_ISL_8311766	hCoV-19/Lebanon/LAU-R101/2020	Col. by CDC-202003	—	—	7,8
EPI_ISL_8311767	hCoV-19/Lebanon/LAU-R146/2020	Col. by CDC-202003	—	—	7,8
EPI_ISL_8311771	hCoV-19/Lebanon/LAU-R104/2020	Col. by CDC-202003	Lebanon/LAU-R104/2020	2022-09-18	6,7,8
EPI_ISL_8317204	hCoV-19/Lebanon/LAU-R99/2020	Col. by CDC-202003	Lebanon/LAU-R99/2020	2023-06-18	6,8
EPI_ISL_8317205	hCoV-19/Lebanon/LAU-R95/2020	Col. by CDC-202003	Lebanon/LAU-R95/2020	2022-06-03	6,7,8
EPI_ISL_8317209	hCoV-19/Lebanon/LAU-R295/2020	Col. by CDC-202003	—	—	7,8
EPI_ISL_8317210	hCoV-19/Lebanon/LAU-R351/2020	Col. by CDC-202003	Lebanon/LAU-R351/2020	2023-06-23	6,7,8
EPI_ISL_2671842	hCoV-19/Japan/20200409-129/2020	Col. by CDC-202003	Japan/20200409-129/2020	2021-06-25	6,7
EPI_ISL_2716627	hCoV-19/Sierra Leone/SL10/2020	Col. by CDC-202003	SierraLeone/SL10/2020	2021-06-01	6,7
EPI_ISL_2716636	hCoV-19/Sierra Leone/SL21/2020	Col. by CDC-202003	SierraLeone/SL21/2020	2021-06-01	6,7
EPI_ISL_4405694	hCoV-19/Argentina/PAIS-A1026/2020	Col. by CDC-202003	Argentina/PAIS-A1026/2020	2021-10-01	6
EPI_ISL_7955525	hCoV-19/Argentina/PAIS-C0160/2020	Col. by CDC-202003	Argentina/PAIS-C0160/2020	2023-06-23	6,7
EPI_ISL_11575530	hCoV-19/USA/CO_c8ftxs_0117/2020	Col. by CDC-202003	USA/CO_c8ftxs_20200117/2020	2023-06-18	6
EPI_ISL_14307752	hCoV-19/USA/MT-UMGC-02796/2020	Col. by CDC-202003	—	—	—
EPI_ISL_3804266	hCoV-19/Niger/16250A/2020	Col. by CDC-202003	—	—	—

1 Virological post, "Temporal signal and the evolutionary rate of 2019 n-CoV using 47 genomes collected by Feb 01 2020", from 3 Feb 2020 (https://virological.org/t/temporal-signal-and-the-evolutionary-rate-of-2019-n-cov-using-47-genomes-collected-by-feb-01-2020/379).

2 Bal et al, "Molecular characterization of SARS-CoV-2 in the first COVID-19 cluster in France reveals an amino acid deletion in nsp2 (Asp268del)", 28 Mar 2020 (https://doi.org/10.1016/j.cmi.2020.03.020).

3 Wang et al, "The establishment of reference sequence for SARS-CoV-2 and variation analysis", 13 Mar 2020 (https://doi.org/10.1002/jmv.25762).

4 Lv et al, "Detection of Phenotype-Related Mutations of COVID-19 via the Whole Genomic Data", 8 Jan 2021 (https://doi.org/10.1109/TCBB.2021.3049836).

5 sarscov2phylo exclusion list, available at https://raw.githubusercontent.com/roblanf/sarscov2phylo/master/excluded_sequences.tsv.

6 Nextstrain exclusion list, available at https://raw.githubusercontent.com/nextstrain/ncov/master/defaults/exclude.txt. Quoted dates reflect first commit where accession appears.

7 Marked as "Under Investigation" in GISAID.

8 Cluster of submissions from Lebanon likely incorrectly annotated with collection date 2020-01-10.

Supplementary Table 1: Excluded GISAID accession IDs for SARS-CoV-2 week-by-week vignette (1 of 2). After short trial runs for each data subset (submitted/collected by end of CDC epiweek N), we visually identified a handful of sequences as clear outliers and excluded them from the analysis. Most of these were also excluded from analyses at the time, are marked "Under Investigation" by GISAID, or are present in the exclusion lists of Nextstrain and/or `sarscov2phylo`.

Accession ID	Name	First anomalous run	Name in Nextstrain	Date excluded in Nextstrain	Notes
EPI_ISL_2835566	hCoV-19/USA/CA-SEARCH-103149/2020	Col. by CDC-202004	USA/CA-SEARCH-103149/2020	2021-06-09	6
EPI_ISL_17116757	hCoV-19/USA/WV-WV064576/2020	Col. by CDC-202004	USA/WV064576/2020	2021-09-01	6
EPI_ISL_17116755	hCoV-19/USA/WV-WV064569/2020	Col. by CDC-202004	USA/WV064569/2020	2021-09-01	6,7
EPI_ISL_19045047	hCoV-19/USA/CA-SEARCH-139716/2020	Col. by CDC-202004	—	—	7
EPI_ISL_19037038	hCoV-19/Ghana/CRI-01/2020	Col. by CDC-202004	—	—	7
EPI_ISL_19037039	hCoV-19/Ghana/CRI-02/2020	Col. by CDC-202004	—	—	7
EPI_ISL_19037040	hCoV-19/Ghana/CRI-03/2020	Col. by CDC-202004	—	—	7
EPI_ISL_19037041	hCoV-19/Ghana/CRI-04/2020	Col. by CDC-202004	—	—	7
EPI_ISL_19037042	hCoV-19/Ghana/CRI-05/2020	Col. by CDC-202004	—	—	7
EPI_ISL_19037043	hCoV-19/Ghana/CRI-06/2020	Col. by CDC-202004	—	—	7
EPI_ISL_19037044	hCoV-19/Ghana/CRI-08/2020	Col. by CDC-202004	—	—	7
EPI_ISL_19037045	hCoV-19/Ghana/CRI-09/2020	Col. by CDC-202004	—	—	7
EPI_ISL_19037046	hCoV-19/Ghana/CRI-10/2020	Col. by CDC-202004	—	—	7
EPI_ISL_19037047	hCoV-19/Ghana/CRI-11/2020	Col. by CDC-202004	—	—	7
EPI_ISL_19037048	hCoV-19/Ghana/CRI-13/2020	Col. by CDC-202004	—	—	7
EPI_ISL_19037049	hCoV-19/Ghana/CRI-14/2020	Col. by CDC-202004	—	—	7
EPI_ISL_19037050	hCoV-19/Ghana/CRI-15/2020	Col. by CDC-202004	—	—	7
EPI_ISL_19037051	hCoV-19/Ghana/CRI-16/2020	Col. by CDC-202004	—	—	7
EPI_ISL_17121378	hCoV-19/USA/OK-PHL-0022663/2020	Col. by CDC-202005	USA/OK-PHL-0022663/2020	2023-06-18	6,7
EPI_ISL_2426018	hCoV-19/Norway/7651/2020	Col. by CDC-202005	Norway/7651/2020	2021-06-07	6
EPI_ISL_3364539	hCoV-19/USA/NY-GEO-0231/2020	Col. by CDC-202005	—	—	7
EPI_ISL_17846484	hCoV-19/USA/un-KDHE-2352575/2020	Col. by CDC-202006	—	—	7
EPI_ISL_1603195	hCoV-19/Spain/CL-COV00781/2020	Col. by CDC-202006	Spain/CL-COV00781/2020	2021-04-19	6,7
EPI_ISL_4899903	hCoV-19/Morocco/INH-108/2020	Col. by CDC-202006	Morocco/INH-108/2020	2021-10-13	6
EPI_ISL_4899911	hCoV-19/Morocco/INH-109/2020	Col. by CDC-202006	Morocco/INH-109/2020	2021-10-13	6
EPI_ISL_4899898	hCoV-19/Morocco/INH-107/2020	Col. by CDC-202006	Morocco/INH-107/2020	2021-10-13	6
EPI_ISL_4899870	hCoV-19/Morocco/INH-103/2020	Col. by CDC-202006	Morocco/INH-103/2020	2022-09-18	6
EPI_ISL_4899881	hCoV-19/Morocco/INH-104/2020	Col. by CDC-202006	Morocco/INH-104/2020	2021-10-19	6
EPI_ISL_4899863	hCoV-19/Morocco/INH-101/2020	Col. by CDC-202006	Morocco/INH-101/2020	2021-10-13	6
EPI_ISL_4899888	hCoV-19/Morocco/INH-105/2020	Col. by CDC-202006	Morocco/INH-105/2020	2021-10-14	6
EPI_ISL_4899917	hCoV-19/Morocco/INH-MN908947/2020	Col. by CDC-202006	Morocco/INH-MN908947/2020	2022-10-06	6
EPI_ISL_4899892	hCoV-19/Morocco/INH-106/2020	Col. by CDC-202006	—	—	—
EPI_ISL_1263332	hCoV-19/Belgium/UZA-UA-CV0615326772/2020	Col. by CDC-202006	Belgium/UZA-UA-CV0615326772/2020	2021-03-18	6
EPI_ISL_1265909	hCoV-19/USA/OH-ODH-SC1040172/2020	Col. by CDC-202007	USA/OH-ODH-SC1040172/2020	2021-03-19	6,7
EPI_ISL_1014733	hCoV-19/Spain/MD-IBV-99018532/2020	Col. by CDC-202008	Spain/MD-IBV-99018532/2020	2021-02-18	6
EPI_ISL_1311841	hCoV-19/Netherlands/ZH-EMC-2080/2020	Col. by CDC-202008	Netherlands/ZH-EMC-2080/2020	2021-03-24	6
EPI_ISL_1311840	hCoV-19/Netherlands/ZH-EMC-2079/2020	Col. by CDC-202008	Netherlands/ZH-EMC-2079/2020	2021-03-24	6
EPI_ISL_10980369	hCoV-19/Zambia/MH23_107_4328/2020	Col. by CDC-202008	—	—	—
EPI_ISL_1136974	hCoV-19/USA/NV-NSPHL-338695/2020	Col. by CDC-202008	USA/NV-NSPHL-338695/2020	2021-03-04	6
EPI_ISL_9879582	hCoV-19/Mongolia/UB-109429/2020	Col. by CDC-202008	Mongolia/UB-109429/2020	2022-04-30	6,7
EPI_ISL_1167830	hCoV-19/Chile/AR-265171/2020	Col. by CDC-202008	Chile/AR-265171/2020	2021-03-08	6
EPI_ISL_7946128	hCoV-19/USA/CA-SEARCH-58338/2020	Col. by CDC-202008	—	—	—
EPI_ISL_7946211	hCoV-19/USA/CA-SEARCH-58362/2020	Col. by CDC-202008	—	—	—
EPI_ISL_7946167	hCoV-19/USA/CA-SEARCH-58348/2020	Col. by CDC-202008	—	—	
EPI_ISL_10980370	hCoV-19/Zambia/MH21_105_5506/2020	Col. by CDC-202009	—	—	7
EPI_ISL_417446	hCoV-19/Italy/LOM-UniMI02/2020	Col. by CDC-202009	Italy/UniMI02/2020	2020-06-08	6,7
EPI_ISL_2758215	hCoV-19/India/un-IRSHA-CD210871/2020	Col. by CDC-202010	India/un-IRSHA-CD210871/2020	2021-06-05	6,7
EPI_ISL_2758214	hCoV-19/India/un-IRSHA-CD210927/2020	Col. by CDC-202010	India/un-IRSHA-CD210927/2020	2021-06-05	6,7
EPI_ISL_2758213	hCoV-19/India/un-IRSHA-CD210929/2020	Col. by CDC-202010	India/un-IRSHA-CD210929/2020	2021-06-05	6,7

1 Virological post, "Temporal signal and the evolutionary rate of 2019 n-CoV using 47 genomes collected by Feb 01 2020", from 3 Feb 2020 (https://virological.org/t/temporal-signal-and-the-evolutionary-rate-of-2019-n-cov-using-47-genomes-collected-by-feb-01-2020/379).

2 Bal et al, "Molecular characterization of SARS-CoV-2 in the first COVID-19 cluster in France reveals an amino acid deletion in nsp2 (Asp268del)", 28 Mar 2020 (https://doi.org/10.1016/j.cmi.2020.03.020).

3 Wang et al, "The establishment of reference sequence for SARS-CoV-2 and variation analysis", 13 Mar 2020 (https://doi.org/10.1002/jmv.25762).

4 Lv et al, "Detection of Phenotype-Related Mutations of COVID-19 via the Whole Genomic Data", 8 Jan 2021 (https://doi.org/10.1109/TCBB.2021.3049836).

5 sarscov2phylo exclusion list, available at https://raw.githubusercontent.com/roblanf/sarscov2phylo/master/excluded_sequences.tsv.

6 Nextstrain exclusion list, available at https://raw.githubusercontent.com/nextstrain/ncov/master/defaults/exclude.txt. Quoted dates reflect first commit where accession appears.

7 Marked as "Under Investigation" in GISAID.

8 Cluster of submissions from Lebanon likely incorrectly annotated with collection date 2020-01-10.

Supplementary Table 2: Excluded GISAID accession IDs for SARS-CoV-2 week-by-week vignette (2 of 2). Continued from previous page.

2 Explicit Mutation-Annotated Trees

Inspired by the Mutation-Annotated Trees (MATs) introduced by UShER [36] and matOptimize [42] to build parsimonious phylogenetic trees, and building on ideas first presented by Nielsen [28], we introduce *Explicit Mutation-Annotated Trees (EMATs)* (see Figure 9). Informally, an EMAT is a timed bifurcating tree with timed decorations on its branches where the sequence changes (*mutations*) or becomes ambiguous (*missations*) at a specific site, together with a reference sequence at a point above the root node. Each leaf (tip) represents a dated sample with a known sequence, although that sequence may have missing data and/or gaps. As usual, all sequences are assumed to be aligned onto an L-site reference, and the sequences specify for each site ℓ ranging from 1 to L one of: (a) the state (A, C, G, T) at that site; or (b) the fact that that site is in an alignment gap ('-') or was not (reliably) sequenced ('N'). As usual, we do not differentiate between gaps and missing data. We also do not distinguish ambiguous base calls (e.g., Y = C or T) and missing sites (N); in practice, this is not a significant limitation. Each inner node represents the most recent common ancestor of its children. Every other point in the tree represents some species along the lineage that connects a node to its parent. As a result, every point in an EMAT has a specific sequence at a subset of the sites in the genome. Mutations and missations encode these sequences and the relevant subsets of sites efficiently, analogously to how mutations in UShER's MATs encode the sequence of nodes in a parsimony tree. EMATs extend MATs by assigning times to both nodes and mutations (so in principle, there may be multiple mutations at the same site along a single branch). Further, missations permit treating missing data efficiently without imputing it; an EMAT with missations represents an appropriately weighted collection of corresponding EMATs where those missations have been replaced by all possible downstream mutations (see Section 5).

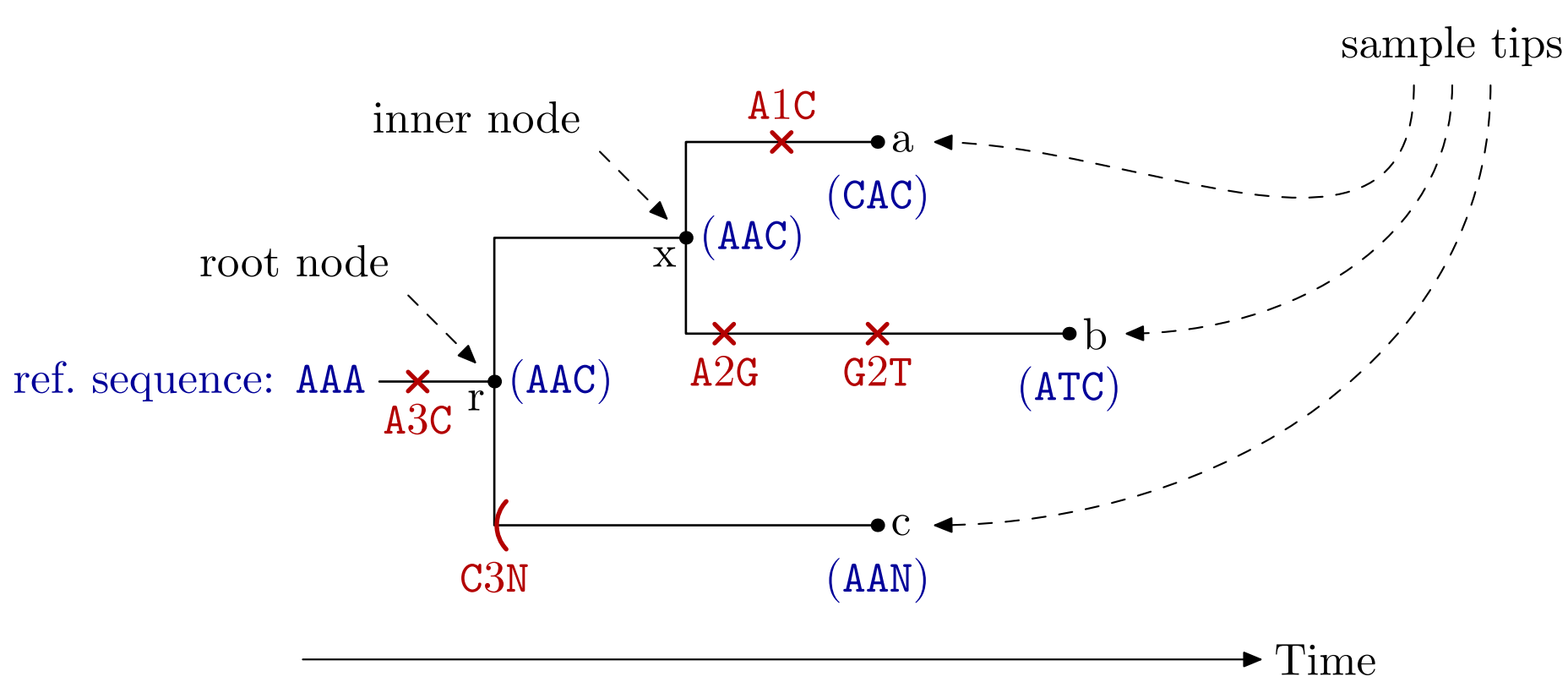

Supplementary Figure 9: An Explicit Mutation-Annotated Tree (EMAT). The nodes a, b and c are dated tips, whose sequences were determined experimentally. The node x is an inner node, the most recent common ancestor of a and b. The root node r is the most recent common ancestor of all the tips. An EMAT explicitly encodes the reference sequence above the root node, the mutation events (red crosses) and the missations (red parentheses). From this representation, the sequence of the species represented at any point in the tree is determined implicitly (shown in parentheses in blue below every node).

Formally, an EMAT $\mathcal{T}$ for N dated input sequences is a timed binary tree with N tips (leaves) and $N-1$ inner nodes. A node is indexed by i ranging from 1 to $2N-1$. For notational convenience, we list tips first, so tip i corresponds to input sequence i. The ancestor of node i is denoted by $\varphi(i)$. The root node, with index r, has no ancestor. However, it is frequently useful to refer to the point *above the root node*, which we denote by $\perp$. Formally, we set $\varphi(r) = \perp$. Each node has a time t_i that grows towards the future. Times must be consistent with the tree topology, so $t_{\varphi(i)} < t_i$ for all i. Tip times correspond to the collection times of the associated samples. Usually, t_i is fixed by the data, but we also allow it to range uniformly along a date range to implement "tip date sampling".

Every node except the root node is the endpoint of a branch from $\varphi(i)$ to i, which we call *branch* i. A point x on a tree is uniquely specified by the branch i that it is on and a time t in the interval $(t_{\varphi(i)}, t_i]$. When we need to give explicit coordinates for a point on the tree, we use the notation $[[i, t]]$; when referring to the point at the node i itself, we often simply use i in place of $[[i, t_i]]$. We also use the notation x^- and x^+ when referring to the point immediately preceding or succeeding x, e.g., if there is a mutation at x and we need to distinguish the sequence before and after the mutation. We denote by τ_i the length of branch i, given by $\tau_i = t_i - t_{\varphi(i)}$. The set of all points on branch i is denoted by B_i. For completeness, we define the root branch B_r to include the single point at the root node, with coordinates $[[r, t_r]]$. The set of all points on the tree is $\mathcal{T} := \cup_i B_i$. We distinguish this set from the tree $\mathcal{T}$ itself only when context does not make the distinction clear.

Every branch i is decorated with a set of mutations $\mathcal{M}_i$. The set of all mutations on the tree is $\mathcal{M} := \cup_i \mathcal{M}_i$. A mutation is a tuple (i, t, a, ℓ, b), which specifies a change in the state of site ℓ from a to b at $[[i, t]]$. In the discussions below, we omit some members of this tuple when they are clear from context or they are irrelevant. In diagrams, we represent mutations as crosses at $[[i, t]]$ labeled $a\ell b$, e.g., `A123C`.

The sequence at x is denoted by the states $s_x^{(\ell)}$ at every site ℓ. The sequence above the root node, denoted by $s_\perp^{(\ell)}$, is an (arbitrary) reference sequence, possibly different from the sequence at the root node. We can reconstruct $s_x^{(\ell)}$ by successively applying to $s_\perp^{(\ell)}$ the mutations on the path from $\perp$ to x in order of encounter. Note that $\mathcal{M}_r$, the mutations on the *root branch* are special: they simply detail the differences between the reference sequence and the sequence at the root node (i.e., the "root sequence"), and their times are meaningless. Separating the reference and root sequences simplifies the implementation of tree moves that change the root node, as described below. While we represent $s_\perp^{(\ell)}$ explicitly, we only reconstruct portions of $s_x^{(\ell)}$ as above when needed.

Each branch is also decorated at its start with "missations" Φ_i, in analogy with mutations. The set of all missations on the tree is $\Phi := \cup_i \Phi_i$. A missation is a tuple (i, a, ℓ) that signals that every species below the starting point of branch i, including all downstream tips, are missing data at site ℓ, whereas in the species immediately ancestral to the missation, the state of site ℓ is a. As with mutations, we omit below some members of this tuple when they are clear from context. In diagrams, we represent missations as a parenthesis ("(") labeled $a\ell$`N`, e.g., `A123N`.

Given an arbitrary point x on the tree, we define $\xi(x)$ as the set of sites for which at least one tip downstream of x is informative. We use $\xi(x)$ to implement our strategy for dealing with missing data, which we call "`N`-pruning" (Section 5). Missations efficiently encode the value of $\xi(x)$ in terms of deltas just before the start of each branch. To calculate $\xi(x)$, we start with the set $\{1, \ldots, L\}$ and successively remove any sites ℓ for which there is a missation at ℓ on the path from $\perp$ to x. Note that missations on the root branch signal sites for which none of the tips are informative. By construction, for a given set of sequences and tree topology, there is a unique way to decorate the tree with missations: $\xi(x)$ is defined in terms of x and its downstream tips, and the missations at branch i simply encode differences between $\xi(\varphi(i)^-)$ and $\xi(\varphi(i)^+)$ immediately above and below the starting point of branch i. This property is essential for correct sampling: each possible way of imputing all the missing data is thus associated with exactly one and only one EMAT. Inspired by MAPLE [20], we encode missations as a sorted list of disjoint contiguous ranges of missing sites (since such groupings are very common in real sequencing data) and the ancestral state is encoded only in terms of its difference from the reference sequence. This representation remains compact even in the face of long sequences with many large gaps (e.g., as is typical in mpox sequences).

In light of the success of UShER, it should be clear that the above representation allows for a very efficient encoding of the sequences in a genomic epidemiology dataset, including the portions of the sequences that are missing. As detailed below, it also allows for very efficient calculations and Monte Carlo moves.

To conclude this section, we introduce notation to integrate over branches and trees. We define the integral over the points B_i of a branch i in terms of the time coordinate, as follows:

$$\int_{x \in B_i} f(x)\, dx := \int_{t_{\varphi(i)}}^{t_i} f([[i, t]])\, dt. \tag{1}$$

Similarly, we define an integral over the tree by summing over its branches:

$$\int_{x \in \mathcal{T}} f(x)\, dx := \sum_i \int_{x \in B_i} f(x)\, dx. \tag{2}$$

Integrals over subtrees, including paths between points on a tree, are defined similarly.

As a trivial example, the length of branch i can be calculated as follows:

$$\tau_i = \int_{x \in B_i} dx = t_i - t_{\varphi i}.$$

Analogously, the total branch length T of the tree can be calculated as:

$$T = \int_{x \in \mathcal{T}} dx.$$

Supplementary Table 4 at the end summarizes the key EMAT notation introduced above.

3 Posterior distribution

The posterior distribution that we sample is, up to normalization, as follows:

$$\mathrm{P}(\mathcal{T}, \boldsymbol{\theta}) \propto \mathrm{L}(\mathcal{T}) \cdot \mathcal{G}(\mathcal{T}|\boldsymbol{\theta}) \cdot \pi_{\mathrm{anc}}(\mathcal{T}|\boldsymbol{\theta}) \cdot \pi_{\boldsymbol{\theta}}(\boldsymbol{\theta}). \tag{3}$$

The four factors are:

- A *parameter prior* $\pi_{\boldsymbol{\theta}}(\boldsymbol{\theta})$ that is a product of priors on associated inference parameters $\boldsymbol{\theta}$, such as the mutation rate μ.
- An *ancestry prior* $\pi_{\mathrm{anc}}(\mathcal{T}|\boldsymbol{\theta})$ on the genealogy of tree $\mathcal{T}$. We have implemented the standard neutral coalescent prior (adapted to be parallelizable as described in Section 9), but other priors are possible (e.g., a Yule pure-birth model, or a sampling-through-time birth-death model [34]).
- A *genetic prior* $\mathcal{G}(\mathcal{T}|\boldsymbol{\theta})$ for the exact root sequence and mutations layered onto the genealogy of $\mathcal{T}$. In the traditional Bayesian framework, this term forms the bulk of the *tree likelihood*, calculated via Felsenstein pruning [9]. Here, the *model* instead describes the exact genetic sequences at every point of the tree, so the terms of the usual tree likelihood appear in a prior.
- The *likelihood* $\mathrm{L}(\mathcal{T})$ of the *data* given the tree $\mathcal{T}$.

We discuss each of these four factors in more detail below. Supplementary Table 5 at the end summarizes the key notation for distributions introduced in this section.

3.1 Likelihood

Given that $\mathcal{T}$ fully specifies the sequences of the tips except where there is missing data, the likelihood $L(\mathcal{T})$ is either 1 if $\mathcal{T}$ agrees with the data, or 0 otherwise. In a more elaborate treatment, the likelihood would be a product of weight vectors at every tip and site, to model, say, sequencing errors; we leave that extension for future work. By choosing our starting tree and MCMC moves to always preserve the compatibility of the model with the experimental data, we avoid ever having to calculate the likelihood explicitly.

3.2 Genetic prior

As usual, we assume that each site ℓ evolves independently according to a continuous-time Markov chain with transition rate matrix $Q_{ab}^{(\ell)}$. For convenience, we denote by $Q_a^{(\ell)}$ the negative of the diagonal elements, so that

$$Q_a^{(\ell)} := -Q_{aa}^{(\ell)} = \sum_{b \neq a} Q_{ab}^{(\ell)}. \tag{4}$$

The second equality reflects that $Q_{ab}^{(\ell)}$ is a stochastic matrix. We denote by $\pi_a^{(\ell)}$ the stationary distribution of this Markov chain at site ℓ, which satisfies

$$\sum_a \pi_a^{(\ell)} Q_{ab}^{(\ell)} = 0. \tag{5}$$

The effect of the CTMC process over a finite time t is captured by the *evolution matrix* $P_{ab}^{(\ell)}$, defined as the probability that a trajectory for site ℓ of length t that starts with state a ends in state b, and given by the following matrix exponential:

$$\boldsymbol{P}^{(\ell)}(t) = \exp(t\boldsymbol{Q}^{(\ell)}). \tag{6}$$

The *total mutation rate at* x, denoted by $\lambda(x)$, is the rate at which mutations happen at point x on the tree on any site in $\xi(x)$. It is given by

$$\lambda(x) := \sum_{\ell \in \xi(x)} Q_{s_x^{(\ell)}}^{(\ell)}. \tag{7}$$

As discussed in Section 5, the restriction to sites in $\xi(x)$ arises from allowing all possible genetic histories below any given missation.

A central result of this paper, which extends Nielsen's original formulation [28], is that the genetic prior $\mathcal{G}(\mathcal{T}|\boldsymbol{\theta})$ has the following form:

$$\mathcal{G}(\mathcal{T}|\boldsymbol{\theta}) = \left\{ \prod_{\ell \in \xi(r)} \pi_{s_r^{(\ell)}}^{(\ell)} \right\} \cdot \exp\left[-\int_{x \in \mathcal{T}} \lambda(x)\, dx \right] \cdot \prod_{(a,\ell,b) \in \mathcal{M}} Q_{ab}^{(\ell)}. \tag{8}$$

This is simply the probability of the particular realizations of the jump chains for the continuous-time Markov processes that model evolution at each site. The first factor is the prior on the root sequence. From there, following every branch in turn, the probability of observing a particular mutational history on that branch is a product of waiting time distributions to each mutation, the probability of choosing the specific mutation observed, and finally a survival probability that there are no further mutations under the end of the branch. Concretely, consider a branch i spanning points $[[i, t_{\varphi(i)}]]$ and $[[i, t_i]]$ on the tree, having an ordered sequence of mutations $\{(i, t'_1, a_1, \ell_1, b_1), \ldots, (i, t'_m, a_m, \ell_m, b_m)\}$. The sequence at a point $[[i, t]]$ has a sequence $\{s_{[[i,t]]}^{(\ell)}\}$, which gives rise to a genome-level mutation at $[[i, t]]$ of $\lambda([[i, t]])$ as above. The probability of a particular jump chain then consists of a product of the following factors:

- The probability that the first mutation occurs a time τ_1:

$$\lambda([[i, t'_1]]^-) \cdot \exp\left[-\lambda([[i, t'_1]]^-) \cdot (t'_1 - t_{\varphi(i)}) \right].$$

- The probability that the mutation happens on site ℓ_1:

$$\frac{Q_{a_1}^{(\ell)}}{\lambda([[i, t'_1]]^-)}.$$

  where, as in Eq. (7), we have $\lambda(x) = \sum_{\ell \in \xi(x)} Q_{s_x^{(\ell)}}^{(\ell)}$.

- The probability that the mutation is specifically from a_1 to b_1:

$$\frac{Q^{(\ell)}_{a_1 b_1}}{Q^{(\ell)}_{a_1}},$$

where, as in Eq. (4), we have $Q^{(\ell)}_a = -Q^{(\ell)}_{aa} = \sum_{b \neq a} Q^{(\ell)}_{ab}$.

- Analogous factors for mutations 2 through m.
- A final survival probability for there being no mutations between mutation m and the end of the branch:

$$\exp\left[-\lambda([[i, t_i]]) \cdot (t_i - t'_m)\right].$$

This product of factors explicitly traces out an alternating sequence of (a) waiting for the next event (of any kind), followed by (b) the probability of a particular event being chosen from among all possible events. Notice that all denominators cancel preceding factors. Hence, using the compact notation introduced above for integrals over branches and trees, the product of all of the above factors for branch i can thus be compactly expressed as:

$$\exp\left[-\int_{x \in \mathcal{B}_i} \lambda(x)\, dx\right] \cdot \prod_{(a,\ell,b) \in \mathcal{M}_i} Q^{(\ell)}_{ab}.$$

Multiplying all such factors over all branches in the tree finally yield Eq. (8).

In contrast to the above genetic prior, the traditional tree likelihood $\mathcal{L}_{\text{tree}}(\mathcal{T}|\boldsymbol{\theta})$, as calculated by Felsenstein pruning and used in BEAST and other similar Bayesian phylogenetics tools, encodes the probability of observing particular states at every node, then sums over all possible values of those states. The equivalence of these two approaches is intuitive, and is indeed a standard result of continuous-time Markov processes. However, for readers unfamiliar with this result, a demonstration of the equivalence using the present notation is given in Section 4. A consequence of the equivalence of these two approaches is that the results from a Delphy run are statistically indistinguishable from an *equivalent* BEAST run.

The crucial property of Eq. (8) is that it is a straight product of simple factors, and the overwhelming majority of those factors cancel out when taking ratios of genetic priors of trees that only differ in a localized region. This observation implies that: (a) local MCMC moves on EMATs can be implemented very efficiently, in time proportional to the number of mutations and missation blocks in nearby branches, not the number of variable sites in the entire dataset (a fact not exploited in Nielsen's original formulation); and (b) local moves acting on different parts of the tree are completely decoupled, and may thus be attempted in parallel. Clearly the EMAT representation trades off some statistical efficiency for substantial mathematical simplifications. However, particularly in genomic epidemiology datasets, where N sample sequences can generally be placed into a parsimony tree involving only $\sim N$ mutations [16, 42], this is a very favorable trade-off.

To establish intuition for the behavior of $\mathcal{G}(\mathcal{T}|\boldsymbol{\theta})$, we consider trees with no missing data and specialize to a Jukes-Cantor model of evolution with no site rate heterogeneity, whereby all possible mutations occur at the same rate $\mu/3$ at every site. In that scenario, the above expression takes the following trivial form:

$$\mathcal{G}(\mathcal{T}|\boldsymbol{\theta}) = \left(\frac{1}{4}\right)^L \cdot e^{-\mu L T} \cdot \left(\frac{\mu}{3}\right)^M, \qquad \text{(Jukes-Cantor model, no site-rate heterogeneity)}, \tag{9}$$

where T is the total branch length of the tree, M is the total number of mutations on it, and, as above, L is the total number of sites. We often use the intuition built on the Jukes-Cantor model to make good MCMC proposals, but then rely on the general expression for $\mathcal{G}(\mathcal{T}|\boldsymbol{\theta})$ in the Metropolis-Hastings ratio to obtain correct samples for the actual evolution model we use.

For some calculations, it is advantageous to organize the calculation of the genetic prior $\mathcal{G}(\mathcal{T}|\boldsymbol{\theta})$ in terms of the contributions made by each branch of $\mathcal{T}$. We thus define the *branch genetic prior* $\mathcal{G}_i(\mathcal{T}|\boldsymbol{\theta})$ for branches i other than the root branch as follows:

$$\mathcal{G}_i(\mathcal{T}|\boldsymbol{\theta}) := \exp\left[-\int_{x \in B_i} \lambda(x)\, dx\right] \cdot \prod_{(a,\ell,b) \in \mathcal{M}_i} Q^{(\ell)}_{ab}, \qquad (i \neq r). \tag{10}$$

An important simplification follows if we define the branch genetic prior of the root branch, $\mathcal{G}_r(\mathcal{T}|\boldsymbol{\theta})$, as follows:

$$\mathcal{G}_r(\mathcal{T}|\boldsymbol{\theta}) = \prod_{\ell \in \xi(r)} \pi^{(\ell)}_{s^{(\ell)}_\perp} \cdot \prod_{(a,\ell,b) \in \mathcal{M}_r} (\pi^{(\ell)}_b / \pi^{(\ell)}_a). \tag{11}$$

With this definition, most subsequent manipulations need not distinguish between the root and nonroot branches. For example, the complete genetic prior is given simply by:

$$\mathcal{G}(\mathcal{T}|\boldsymbol{\theta}) = \prod_i \mathcal{G}_i(\mathcal{T}|\boldsymbol{\theta}).$$

In the definition of $\mathcal{G}_r(\mathcal{T}|\boldsymbol{\theta})$, we have also factored out the contribution of the reference sequence for the sites $\xi(r)$ that are missing in every input sequence; the leftover factors then arise from mutations above the root node. By arranging for most MCMC moves to leave $s^{(\ell)}_\perp$ unchanged ($\xi(r)$ is fixed throughout the run), only the factors arising from mutations above the root node survive when taking ratios of branch genetic priors for the root branch.

We define path and subtree genetic priors in the obvious way as products of the relevant branch genetic priors.

Just as it is sometimes convenient to split the calculation of the genetic prior according to the contributions made by each branch, it is also sometimes convenient to split it according to the contributions made by each site. Thus, we define the *site genetic prior* $\mathcal{G}^{(\ell)}(\mathcal{T}|\boldsymbol{\theta})$ as the factors of $\mathcal{G}(\mathcal{T}|\boldsymbol{\theta})$ that relate to site ℓ. Explicitly,

$$\mathcal{G}^{(\ell)}(\mathcal{T}|\boldsymbol{\theta}) = \begin{cases} \pi^{(\ell)}_{s^{(\ell)}_r} \cdot \exp\left[-\int_{x\in\mathcal{T}} \lambda^{(\ell)}(x)\,dx\right] \cdot \prod_{(a,b)\in\mathcal{M}^{(\ell)}} Q^{(\ell)}_{ab}, & \ell \in \xi(r); \\ 1, & \text{otherwise.} \end{cases} \tag{12}$$

Here, $\lambda^{(\ell)}(x)$ is either the single term $Q^{(\ell)}_{s^{(\ell)}_x}$ if $\ell \in \xi(x)$, or 0 otherwise; and $\mathcal{M}^{(\ell)}$ is the subset of mutations in $\mathcal{M}$ occurring at site ℓ. By construction,

$$\mathcal{G}(\mathcal{T}|\boldsymbol{\theta}) = \prod_\ell \mathcal{G}^{(\ell)}(\mathcal{T}|\boldsymbol{\theta}).$$

Clearly, the site genetic prior is closely related to the traditional site likelihood. This relation is detailed in Section 4.

Finally, we can decompose $\mathcal{G}(\mathcal{T}|\boldsymbol{\theta})$ yet more finely into a product of *site-branch genetic priors* $G^{(\ell)}_i(\mathcal{T}|\boldsymbol{\theta})$ as follows:

$$\mathcal{G}^{(\ell)}_i(\mathcal{T}|\boldsymbol{\theta}) = \begin{cases} \pi^{(\ell)}_{s^{(\ell)}_r}, & i = r \text{ and } \ell \in \xi(r); \\ \exp\left[-\int_{x\in\mathcal{B}_i} \lambda^{(\ell)}(x)\,dx\right] \cdot \prod_{(a,b)\in\mathcal{M}^{(\ell)}_i} Q^{(\ell)}_{ab}, & i \neq r \text{ and } \ell \in \xi(r); \\ 1, & \text{otherwise.} \end{cases} \tag{13}$$

As above, $\mathcal{M}^{(\ell)}_i$ is the subset of mutations in $\mathcal{M}$ occurring at site ℓ on branch i.

Finally, we remark that calculating the integral $\int_{x\in B_i} \lambda(x)\,dx$, and thus $\mathcal{G}(\mathcal{T}|\boldsymbol{\theta})$, is considerably simplified if we keep track of the value of $\lambda(x)$ at every node i, which we denote by λ_i. First, we note that we always store the mutations in $\mathcal{M}_i$ sorted by ascending time. Label their identities by the tuples $(a_j, \ell_j, b_j, t_{i,j})$ for $1 \le j \le |\mathcal{M}_i|$ in the obvious way, and set $t_{i,0} = t_{\varphi(i)}$ and $t_{i,|\mathcal{M}_i|+1} = t_i$. Then compute the quantities $\lambda_{i,j}$, which are the values of $\lambda(x)$ between mutations j and $j+1$, with $0 \le j \le |\mathcal{M}_i|$. These obey the following recursion relations:

$$\lambda_{i,|\mathcal{M}_i|} = \lambda_i, \tag{14}$$

$$\lambda_{i,j-1} = \lambda_{i,j} - Q^{(\ell_j)}_{b_j} + Q^{(\ell_j)}_{a_j}, \qquad (1 \le j \le |\mathcal{M}_i|). \tag{15}$$

With these quantities, we obtain:

$$\int_{x\in B_i} \lambda(x)\,dx = \sum_{j=0}^{|\mathcal{M}_i|} \lambda_{i,j}(t_{i,j+1} - t_{i,j}).$$

To calculate λ_i for all nodes, note that there is a simple recurrence relation between the total mutation rate at a node i and that at its ancestor:

$$\lambda_i = \lambda_{\varphi(i)} + \sum_{(a,\ell,b)\in\mathcal{M}_i} (Q_b^{(\ell)} - Q_a^{(\ell)}) - \sum_{(a,\ell)\in\Phi_i} Q_a^{(\ell)}. \tag{16}$$

Since $\lambda_\perp = \sum_\ell Q^{(\ell)}_{s^{(\ell)}_\perp}$, it follows that having calculated $\lambda_\perp$, the subsequent calculation of λ_i for every node i can be done in time linear with the size of the tree and the number of mutations and missations on its branches. Hence, $\mathcal{G}(\mathcal{T}|\boldsymbol{\theta})$ can also be calculated in linear time, and changes to it arising from changes to a subtree can be calculated in time linear with the number of mutations and missations in that subtree.

3.3 Ancestry prior

Any ancestry prior suitable for Bayesian phylogenetics can be used in our framework. For concreteness, we have implemented the traditional Kingman coalescent prior [14, 15]. A significant complication in parallelizing the coalescent prior is that it directly couples all the nodes at the end of branches that cross a given time t. We discuss the details of the ancestry prior and an equivalent formulation that is parallelizable in Section 9.

3.4 Parameter priors

The genetic prior is defined in terms of the site transition rate matrices $Q^{(\ell)}_{ab}$. Our framework accommodates all common choices for these, e.g., multiple site partitions with linked or unlinked mutation rates, with or without site rate heterogeneity, with more (GTR) or fewer (HKY) free exchangeabilites, with some proportion of invariant sites, etc. With minor modifications, more elaborate schemes can also be accommodated, e.g., uncorrelated relaxed molecular clocks. Similarly, the ancestral prior is defined in terms of the population curve $N(t)$, and all models in common use can be applied, from a fixed flat population, to parametrized exponential growth, to more flexible schemes such as a Skygrid prior [26].

For concreteness, however, we have thus far only implemented an evolution model consisting of a single-partition HKY model with optional site-rate heterogeneity and no invariant sites, and a either a population model representing exponential growth with tunable hyperparameter priors whose defaults mimic the model used in [18], or a non-parametric Skygrid population model[1]. We leave it to future work to implement more flexible specifications of evolution and population models, along with their respective MCMC moves.

In detail, the concrete evolution model we have implemented is:

$$Q^{(\ell)}_{ab} = \mu\nu^{(\ell)} q_{ab}, \tag{17}$$

where μ is the overall mutation rate, $\nu^{(\ell)}$ is a site-relative rate, and q_{ab} is the usual reduced transition rate matrix for the HKY model with stationary base frequencies π_a and transition-transversion ratio κ, given by:

$$q_{ab} = r_{ab}\pi_b/R, \quad (a \neq b) \qquad \text{with } r_{ab} := \begin{pmatrix} 0 & 1 & \kappa & 1 \\ 1 & 0 & 1 & \kappa \\ \kappa & 1 & 0 & 1 \\ 1 & \kappa & 1 & 0 \end{pmatrix} \text{ and } R := \sum_{ab} \pi_a r_{ab} \pi_b. \tag{18}$$

By default, the hyperparameters μ, $\nu^{(\ell)}$, π_a and κ have the same priors as those produced by default when configuring a BEAST 2 run with BEAUti 2 for an HKY model and a strict molecular clock, which are those used in [18]. However, to allow for proper priors in the context of the Well-Calibrated Simulation Studies (WCSSs) of Section 15, we implemented more flexible priors, carefully chosen for conjugacy:

[1] We have also successfully run a two-partition model in the context of analyzing mpox sequences, where some sites are susceptible to fast APOBEC-mediated mutations [29] while other sites exhibit only mutations from polymerase errors (unpublished).

- Mutation rate μ: by default, a uniform, improper prior,

$$\pi_\mu(\mu) = 1, \qquad \text{(default)}. \tag{19}$$

In general, π_μ is a Gamma(α_μ, β_μ) prior with user-specified shape α_μ and rate β_μ (equivalently, user-specified mean and standard deviation when $\beta_\mu > 0$):

$$\pi_\mu(\mu) \propto \mu^{\alpha_\mu - 1} e^{-\beta_\mu \mu}, \qquad \text{(general form)}. \tag{20}$$

By default, $\alpha_\mu = 1$ and $\beta_\mu = 0$. This prior is proper whenever $\beta_\mu > 0$.

- Site relative mutation rates: We either set $\nu^{(\ell)} = 1$ for all ℓ (no site rate heterogeneity), or we choose $\nu^{(\ell)}$ to be Gamma distributed with mean 1 and shape parameter α:

$$\pi_\nu(\{\nu^{(\ell)}\}|\alpha) = \prod_\ell \frac{\alpha^\alpha}{\Gamma(\alpha)} \left[\nu^{(\ell)}\right]^{\alpha-1} e^{-\alpha\nu^{(\ell)}}. \tag{21}$$

We emphasize that in our framework, all the $\nu^{(\ell)}$ parameters are explicit, independent, and continuous, not restricted in the usual way to discrete categories [41]. The site rate heterogeneity parameter α is an additional latent parameter, itself associated with a prior distribution $\pi_\alpha(\alpha)$, which we take to be exponential with mean $\mu_\alpha = 1$:

$$\pi_\alpha(\alpha) = \frac{1}{\mu_\alpha} e^{-\alpha/\mu_\alpha}. \tag{22}$$

- Evolution model parameters: the stationary base frequencies have a Dir$(1, 1, 1, 1)$ prior, while κ has a log-normal prior with mean $\mu_{\log\kappa} = 1.0$ and standard deviation $\sigma_{\log\kappa} = 1.25$:

$$\pi_Q(\kappa, \{\pi_a\}) = 1 \cdot \frac{1}{\log\kappa \cdot \sqrt{2\pi\sigma^2_{\log\kappa}}} e^{-(\log\kappa - \mu_{\log\kappa})^2/2\sigma^2_{\log\kappa}}. \tag{23}$$

- Population model parameters: a user can choose between a pure exponential growth model or a non-parametric Skygrid model. Different priors apply in each case:
  - For an **exponential growth** population model, the precise population curve $N(t)$ is given by

    $$N(t) := n_0 \cdot e^{g(t-t_0)}, \tag{24}$$

    where t_0 is the time of the latest tip. As with the evolution model, by default we use the same priors for n_0 and g that are output by default by BEAUti 2 when preparing a BEAST 2 run with a "Coalescent Exponential Population", namely:
    * An improper $1/x$-prior on the effective population size n_0 at time t_0,

      $$\pi_{n_0}(n_0) = 1/n_0, \qquad \text{(default)}.$$

      In general, π_{n_0} is an Inv-Gamma$(\alpha_{n_0}, \beta_{n_0})$ prior (i.e., $1/n_0 \sim$ Gamma$(\alpha_{n_0}, \beta_{n_0})$) with user-specified shape α_{n_0} and rate β_{n_0} (equivalently, a user-specified mean and standard deviation when $\alpha_{n_0} > 2$ and $\beta_{n_0} > 0$):

      $$\pi_{n_0}(n_0) \propto n_0^{-\alpha_{n_0}-1} e^{-\beta_{n_0}/n_0}, \qquad \text{(general form)}.$$

      By default, $\alpha_{n_0} = 0$ and $\beta_{n_0} = 0$. This prior is proper whenever $\alpha_{n_0} > 2$ and $\beta_{n_0} > 0$.

* A Laplace prior on the growth rate g, with configurable mean μ_g and scale s_g. Additionally, we allow imposing a hard lower bound $g_{\min}$ and a hard upper bound $g_{\max}$. By default, $\mu_g = 0.001\,\mathrm{yr}^{-1}$, $s_g = 30.701135\,\mathrm{yr}^{-1}$, $g_{\min} = -\infty$ and $g_{\max} = +\infty$:

$$\pi_g(g) \propto \frac{1}{s_g} \exp\left(-\frac{|g-\mu_g|}{s_g}\right) \cdot [g_{\min} < g < g_{\max}].$$

  Here, $[X]$ is the Iverson bracket, which is 1 when condition X is true and 0 otherwise. Note that π_g reduces to an exponential distribution with mean s_g when $\mu_g = g_{\min} = 0$.

– For a **Skygrid** population model, we implement the flexible nonparametric method in [11]. Following their notation, a user picks the number M of "Skygrid intervals" and a cutoff time x_0. Fixing the (maximum) time of the latest tip at x_M, this determines a set of $M+1$ "knot" times $\{x_0, x_1, \ldots, x_M\}$ given by

$$x_k := x_0 + k\frac{x_M - x_0}{M}. \tag{25}$$

A user can also instead specify the values of x_0, x_M and M directly, with the $M-1$ intermediate times spaced evenly between x_0 and x_M. (Note that, for coherence with the rest of Delphy, time increases towards the future, unlike in [11], where it advances towards the past.) The value of the population curve is specified parametrically at these knots by a set of $M+1$ parameters $\{\gamma_0, \gamma_1, \ldots, \gamma_M\}$ as follows:

$$\log N(x_k) = \gamma_k. \tag{26}$$

To complete specifying the full population curve, we interpolate the values between knots, before the first knot and after the last knot. In the original Skygrid formulation, which we denote by "staircase" (and matches what is implemented in BEAST X), the value of $N(t)$ is constant between two knots and equal to the value at the closest future knot, as follows:

$$\log N(t) = \begin{cases} \gamma_0, & t \le x_0; \\ \gamma_k, & x_{k-1} < t \le x_k; \\ \gamma_M, & x_M < t; \end{cases} \qquad \text{(standard "staircase" Skygrid).} \tag{27}$$

We have also implemented a variant where the interpolation is continuous and piecewise linear, which we call "log-linear", as follows:

$$\log N(t) = \begin{cases} \gamma_0, & t \le x_0; \\ (1-c)\gamma_{k-1} + c\gamma_k, & x_{k-1} < t \le x_k; \quad \text{where } t = (1-c)x_{k-1} + c x_k \\ \gamma_M, & x_M < t; \end{cases} \qquad \text{("log-linear" Skygrid).} \tag{28}$$

Empirically, we have found the log-linear variant to be more robust: lacking discontinuities, it is far less susceptible to getting the MCMC into the metastable states described below.

The following priors are in place when using a Skygrid model:

* A Gaussian Markov Random Field prior for the log-population at the knots. Concretely, as in [11], we impose the following 1D random walk prior on the population curve:

$$\pi_\gamma = \exp\left[-\frac{\tau}{2}\sum_{k=1}^{M}(\gamma_k - \gamma_{k-1})^2\right].$$

  Having found the precision parameter τ difficult to interpret, we instead parametrize this prior with a diffusion constant D as follows:

$$\pi_\gamma(\{\gamma_k\}) = \exp\left[-\sum_{k=1}^{M}\frac{(\gamma_k - \gamma_{k-1})^2}{4D\Delta t}\right],$$

where $\Delta t := (x_M - x_0)/M$. In this formulation, it is easy to specify population curves with different resolutions but similar a priori behavior, by fixing D. Concretely, the a priori distribution of the difference between γ_k and γ_{k+n} is exactly $\mathcal{N}(0, 2Dn\Delta t)$, and for intermediate times T, we have approximately

$$\log N(t+T) - \log N(t) \sim \mathcal{N}(0, 2DT), \qquad \text{(a priori; approximate unless } T = n\Delta t\text{)}.$$

Though we have not exploited this possibility, a parametrization in terms of a diffusion constant naturally allows for nonuniform spacing between knots. We further make the parametrization more intuitive by specifying the typical timescale T_2 over which the population fluctuates a priori by a factor of 2, i.e., at which the standard deviation of the log-population change is $\log 2$. The following formulas evidently relate D, T_2 and τ:

$$T_2 = \frac{\log^2 2}{2D} \quad \Leftrightarrow \quad D = \frac{\log^2 2}{2T_2} \quad \Leftrightarrow \quad \tau = \frac{T_2/\Delta t}{\log^2 2}. \tag{29}$$

We have found it both more stable and more physically meaningful to fix T_2 to some user-specified value that captures "reasonable" timescales over which viral populations can change, instead of imposing a very soft prior on τ and trying to infer τ from the data, as is usually done (in our experience, an inferred τ is nearly unconstrained by typical datasets; see Supplementary Figure 22 for an example). Of course, given enough data, it's possible to infer population curves that evolve much more quickly or slowly than this a priori timescale. By default, we fix T_2 to 1 month, but we also allow inference of τ subject to the standard Gamma prior parametrized by α_τ and β_τ (by default both equal to 0.001):

$$\pi_\tau(\tau) = \tau^{\alpha_\tau - 1} e^{-\beta_\tau \tau}, \qquad \text{(when } \tau \text{ is not fixed)}.$$

∗ An Inverse-Gamma prior on the overall population level. In the standard Skygrid prior, the overall population level is unconstrained, so the prior is improper; this additional prior enables use cases that do require proper priors (e.g., the WCSSs in Section 15). Concretely, we define derived quantities $\bar{\gamma}$ and $\bar{N}$ as follows:

$$\bar{\gamma} := \frac{1}{M+1} \sum_{k=0}^{M} \gamma_k,$$
$$\bar{N} := \exp(\bar{\gamma}).$$

Physically, $\bar{N}$ is the geometric mean of the population sizes at the knots of the Skygrid population curve $N(t)$. We impose on $\bar{N}$ an Inv-Gamma$(\alpha_{\bar{N}}, \beta_{\bar{N}})$ prior (i.e., $1/\bar{N} \sim \text{Gamma}(\alpha_{\bar{N}}, \beta_{\bar{N}})$) with user-specified shape $\alpha_{\bar{N}}$ and rate $\beta_{\bar{N}}$ (equivalently, a user-specified mean and standard deviation when $\alpha_{n_0} > 2$ and $\beta_{n_0} > 0$):

$$\pi_{\bar{N}}(\bar{N}) \propto \bar{N}^{-\alpha_{\bar{N}} - 1} e^{-\beta_{\bar{N}}/\bar{N}}.$$

By default, $\alpha_{\bar{N}} = 0$ and $\beta_{\bar{N}} = 0$, resulting in a $1/\bar{N}$ prior on $\bar{N}$, which is equivalent to the standard improper uniform prior on $\bar{\gamma}$ (the factor $1/\bar{N}$ arises from the Jacobian in transforming the sampling coordinates $\{\gamma_k\}$ to $\{1/\bar{N}; \gamma_k - \bar{\gamma} | k < M\}$; see Section 7.3). This prior is proper whenever $\alpha_{\bar{N}} > 2$ and $\beta_{\bar{N}} > 0$.

∗ A "low-population barrier" prior. During development, we occasionally observed a failure mode where in one Skygrid interval, the population curve would be driven to a very low value, followed by a much higher value in the next interval. This causes all active branches at the boundary between these intervals to coalesce just before the boundary. Once the tree and population curve enter this configuration, the run tends to get stuck: the high number of coalescences in the first interval reinforces the low population there, while the

absence of coalescences in the second intervals reinforces the high population there. The main counterweight to this behaviour is a high value of τ, but when τ is inferred, the large difference in populations between these two consecutive intervals favors a low value of τ. In turn, this low value of τ allows other pairs of intervals to enter the problematic low-population/high-population configuration. It also triggers numerical instabilities in our HMC sampler (described below). These are the main reason we prefer to fix τ a priori to match our prior beliefs about the fluctuations in viral population curves.

Despite fixing τ, some of our runs still got stuck as described above. Thus, we also found it helpful to introduce a second defense against abnormally low populations in any one interval, which we call the "low-population barrier". Recall that the effective population size is implicitly multiplied by a generation time to form a coalescence timescale (the typical timescale it takes any pair of lineages to coalesce). Recognizing that viral collection dates are rarely more precise than 1 day, and that it's unlikely that the coalescence timescale is below 1 day in practice (implying that all currently circulating viral particles are descended from a single viral particle that was present merely ~ 2 days previously), we strongly penalize such low populations with the following prior:

$$\pi_{\text{low}}(\{\gamma_k\}) = \sum_{k=0}^{M} \begin{cases} 0, & \gamma_k \geq \gamma_{\min}; \\ (\gamma_{\min} - \gamma_k)^2/\sigma_B^2, & \gamma_k < \gamma_{\min}. \end{cases}$$

By default, $\gamma_{\min} = \log(1 \text{ day})$ and $\sigma_B = -\log(0.7)$, representing a penalty of 1 nat for the log-population dropping 30 % below γ_b, of 4 nats for dropping 30 % below that again, and so on. These particular choices are somewhat arbitrary, and we note that in the production parts of a typical MCMC run, one never observes any γ_k approach $\gamma_{\min}$. The above barrier simply prevents the MCMC run from getting stuck in an implausible metastable state from which it's hard to recover, particularly during the burn-in phase. We also note that when running direct comparisons with BEAST X, we disabled this low-population barrier to ensure that both Delphy and BEAST X were sampling from equivalent posteriors.

Empirically, we observe that by using the "log-linear" Skygrid interpolation, fixing τ to a physically reasonable value, and applying the low-population barrier, Delphy's MCMC sampler never gets stuck in any of our real-world viral datasets. This robustness obviates the need for any parameter fine-tuning from a user's perspective. For very advanced users, all of the above choices can be overridden.

With the concrete choices above, the overall prior on latent parameters is simply the product of the above priors:

$$\pi_{\boldsymbol{\theta}}(\boldsymbol{\theta}) = \pi_\mu(\mu) \cdot \left\{ \begin{array}{c} \pi_\nu(\{\nu^{(\ell)}\}|\alpha) \cdot \pi_\alpha(\alpha) \\ 1 \end{array} \right\} \cdot \pi_Q(\kappa, \{\pi_a\}) \cdot \left\{ \begin{array}{l} \pi_{n_0}(n_0) \cdot \pi_g(g) \\ \pi_\gamma(\{\gamma_k\}) \cdot \pi_{\bar{N}}(\bar{N}) \cdot \left\{ \begin{array}{c} \pi_\tau(\tau) \\ 1 \end{array} \right\} \cdot \left\{ \begin{array}{c} \pi_{\text{low}}(\{\gamma_k\}) \\ 1 \end{array} \right\} \end{array} \right\}. \quad (30)$$

4 Genetic prior vs. tree likelihood

In this section, we explicitly establish that integrating the genetic prior (Equation (8)) over all possible EMATs with fixed topology yields the traditional tree likelihood, as computed via Felsenstein pruning [9]. Hence, what distinguishes Delphy from the traditional Bayesian methods based on Felsenstein pruning is that it samples over explicit topologies and genetic histories using a simple posterior, instead of integrating out the genetic histories analytically and then sampling only over topologies using the resulting more computationally demanding posterior. Otherwise, the methods are statistically equivalent. A reader comfortable with this standard result can safely skip this section.

Throughout this section, we assume that no data is missing. Section 5 details how implicit tracking of some ambiguous sites minimally alters the genetic prior.

First, we make precise the notion of "integrating over all genetic histories". Consider the evolution of single site over a single lineage and a fixed period of time. Define a site genetic history $\mathcal{M}$ as a set of $K \geq 0$ mutations, specified as tuples (t_k, u_k, v_k) where the state at the site changes from u_k to v_k at time t_k. Such a history satisfies three coherency conditions: (a) $u_k \neq v_k$ for all k; (b) $v_k = u_{k+1}$ for $0 \leq k < K$; and (c) $t_1 < \cdots < t_K$. Let $\mathcal{H}_{ab}(p,q)$ be the set of site genetic histories that further have starting state a at time p and an ending state b at time q. Formally, this means that each such site genetic history satisfies the following additional conditions: (a) if $K > 0$, then $u_1 = a$ and $v_K = b$; otherwise, if $K = 0$, then $\mathcal{H}_{ab}(p,q)$ is nonempty only when $a = b$, in which case, the only genetic history it contains is the empty set; (b) $p < t_1$ and $t_K < q$. We define an integral over all such site genetic histories as follows:

$$\int_{\mathcal{H}_{ab}(p,q)} d\mathcal{M}\, f(\mathcal{M}) := \delta_{ab} f(\emptyset) + \sum_{K=1}^{\infty} \sideset{}{'}\sum_{\{u_k,v_k\}} \int_{p<t_1<\cdots<t_K<q} dt_1 \cdots dt_K\, f(\mathcal{M}).$$

The primed sum is over all possible assignments of states to u_k and v_k that produce a valid site genetic history in $\mathcal{H}_{ab}(p,q)$.

We define $R^{(\ell)}_{ab}(p,q)$ as the integral over all site genetic histories in $\mathcal{H}_{ab}(p,q)$ of the kinds of factors that appear in the site genetic prior $\mathcal{G}^{(\ell)}(\mathcal{T}|\boldsymbol{\theta})$:

$$R^{(\ell)}_{ab}(p,q) := \int_{\mathcal{H}_{ab}(p,q)} d\mathcal{M}\, \exp\left[-\int_p^q dt\, Q^{(\ell)}_{s^{(\ell)}(t)}\right] \cdot \prod_{(a,b)\in\mathcal{M}} Q^{(\ell)}_{ab} \tag{31}$$

Here, $s^{(\ell)}(t)$ is the state of site ℓ at time t according to the genetic history $\mathcal{M}$. That is, first $s^{(\ell)}(p) = a$, then $s^{(\ell)}(t)$ assumes the various values of v_k as t crosses t_k, until finally, $s^{(\ell)}(q) = b$.

We show below that the above integral over all genetic histories is closely related to the traditional evolution matrix $\boldsymbol{P}^{(\ell)}(t)$. The element $P^{(\ell)}_{ab}(t)$ of this matrix is the probability that a state a at site ℓ evolves to a state b over a time t along a single lineage. This matrix is defined by the forward Kolmogorov equation of the Markov process and a suitable initial condition:

$$\frac{dP^{(\ell)}_{ab}(t)}{dt} = \sum_c P^{(\ell)}_{ac}(t) Q^{(\ell)}_{cb}; \tag{32}$$

$$P^{(\ell)}_{ab}(0) = \delta_{ab}. \tag{33}$$

For time-independent $Q^{(\ell)}_{ab}$, one obtains the usual result, $\boldsymbol{P}^{(\ell)}(t) = \exp(\boldsymbol{Q}^{(\ell)} t)$. We note in passing that our treatment below is almost unchanged for time-dependent $Q^{(\ell)}_{ab}$, and may thus provide a tractable way of modeling time-inhomogeneous evolution in a Bayesian phylogenetics context.

We can now state the key result of this section, namely, that the above integral over all site genetic histories evaluates to an evolution matrix element:

$$R^{(\ell)}_{ab}(p,q) = P^{(\ell)}_{ab}(q-p). \tag{34}$$

To show this, we rewrite the definition of $R^{(\ell)}_{ab}(p,q)$ in Equation (31) by first separating the term where there are no mutations from p to q, then writing the remainder in terms of the time t' of the last mutation and the site genetic history preceding that mutation. For later convenience, we also replace $Q^{(\ell)}_b$ by $-Q^{(\ell)}_{bb}$:

$$R^{(\ell)}_{ab}(p,q) := \delta_{ab} e^{Q^{(\ell)}_{bb}(q-p)} + \int_p^q dt' \sum_{c\neq b} R^{(\ell)}_{ac}(p,t') Q^{(\ell)}_{cb} e^{Q^{(\ell)}_{bb}(q-t')}.$$

We then calculate the derivative of $R^{(\ell)}_{ab}(p, q)$ with respect to q:

$$\begin{aligned}
\frac{dR^{(\ell)}_{ab}(p,q)}{dq} &= \delta_{ab} e^{Q^{(\ell)}_{bb}(q-p)} Q^{(\ell)}_{bb} \\
&\qquad + \int_p^q dt' \sum_{c\neq b} R^{(\ell)}_{ac}(p,t') Q^{(\ell)}_{cb} e^{Q^{(\ell)}_{bb}(q-t')} Q^{(\ell)}_{bb} \\
&\qquad + \sum_{c\neq b} R^{(\ell)}_{ac}(p,q) Q^{(\ell)}_{cb} \\
&= R^{(\ell)}_{ab}(p,q) Q^{(\ell)}_{bb} + \sum_{c\neq b} R^{(\ell)}_{ac}(p,q) Q^{(\ell)}_{cb} \\
&= \sum_{c} R^{(\ell)}_{ac}(p,q) Q^{(\ell)}_{cb}.
\end{aligned}$$

We also observe that when $p = q$, we have:

$$R^{(\ell)}_{ab}(p,p) = \delta_{ab}.$$

Since $R^{(\ell)}_{ab}(p, q)$ satisfies the same differential equation and initial conditions as $P^{(\ell)}_{ab}(t)$, we conclude they are equivalent (Equation (34)).

Next, we generalize the concept of site genetic histories to encompass mutations at any site along a single branch j. For convenience, let $i = \varphi(j)$. Thus, branch j starts at node i, with time t_i, and ends at node j, with time t_j. Define a branch genetic history $\mathcal{M}_j$ on branch j as a set of $K \geq 0$ mutations, specified as tuples (j, t', a', ℓ, b'), where $t_i < t' < t_j$ and $1 \leq \ell \leq L$. Each tuple records that at time t', the state of site ℓ on branch j changes from a' to b'. A branch genetic history can be partitioned into individual site genetic histories, each of which must satisfy the coherency conditions (a)–(c) given at the beginning of this section. Denote by $\mathcal{H}_j$ the set of branch histories over branch j, compatible with fixed sequences $\{s^{(\ell)}_i\}$ and $\{s^{(\ell)}_j\}$ at its endpoints. Thus, an arbitrary member of $\mathcal{H}_j$ consists of the union of L sets, one for each site ℓ, derived each from $\mathcal{H}_{s^{(\ell)}_i s^{(\ell)}_j}(t_i, t_j)$. We define an integral over all branch genetic histories in $\mathcal{H}_j$ as follows:

$$\int_{\mathcal{H}_j} d\mathcal{M}_j\, f(\mathcal{M}_j) := \left[\prod_{\ell} \int_{\mathcal{H}_{s^{(\ell)}_i s^{(\ell)}_j}(t_i,t_j)} d\mathcal{M}^{(\ell)}\right] f\Big(\bigcup_{\ell} g^{(\ell)}_j[\mathcal{M}^{(\ell)}]\Big). \tag{35}$$

The function $g^{(\ell)}_j[\mathcal{M}^{(\ell)}]$ maps each tuple (t_k, u_k, v_k) in $\mathcal{M}^{(\ell)}$ to (j, t_k, u_k, ℓ, v_k).

With the above definitions in place, we note that the product of all the $R^{(\ell)}_{ab}(p, q)$ factors associated with a given branch j can be simply expressed as an integral over branch genetic histories in $\mathcal{H}_j$, where mutations can occur over any site. Explicitly, it follows from Equations (31) and (35) that

$$\prod_{\ell} R^{(\ell)}_{s^{(\ell)}_i s^{(\ell)}_j}(t_i, t_j) = \int_{\mathcal{H}_j} d\mathcal{M}_j\, \exp\left[-\int_{t_i}^{t_j} dt \sum_{\ell} Q^{(\ell)}_{s^{(\ell)}(t)}\right] \cdot \prod_{(a,\ell,b)\in\mathcal{M}_j} Q^{(\ell)}_{ab}.$$

Using Equation (34) to express the left-hand side in terms of evolution matrix elements, and then using Equation (7) for the total mutation rate $\lambda(x)$ at a point x on the tree (when all sites are explicitly tracked everywhere), we obtain the equivalent relation

$$\prod_{\ell} P^{(\ell)}_{s^{(\ell)}_i s^{(\ell)}_j}(t_j - t_i) = \int_{\mathcal{H}_j} d\mathcal{M}_j\, \exp\left[-\int_{x\in\mathcal{B}_j} \lambda(x)\, dx\right] \cdot \prod_{(a,\ell,b)\in\mathcal{M}_j} Q^{(\ell)}_{ab} = \int_{\mathcal{H}_j} d\mathcal{M}_j\, \mathcal{G}_j(\mathcal{T}|\boldsymbol{\theta}).$$

At the end, we've noted that the integrand above is just the branch genetic prior $\mathcal{G}_j(\mathcal{T}|\boldsymbol{\theta})$ of Equation (10).

We now further generalize genetic histories to cover the entire tree. Define a tree genetic history $\mathcal{M}_{\mathcal{T}}$ as a union of branch genetic histories, one for each branch except the root branch. A tree genetic history must satisfy the obvious coherency condition that the ending state of the branch genetic history of branch $\varphi(i)$ is the starting state of branch i. Denote by $\mathcal{H}_{\mathcal{T}}$ the set of tree genetic histories compatible with fixed sequences $\{s_i^{(\ell)}\}$ at every node i, including the root node. We define an integral over all tree genetic histories in $\mathcal{H}_{\mathcal{T}}$ as follows:

$$\int_{\mathcal{H}_{\mathcal{T}}} d\mathcal{M}_{\mathcal{T}}\, f(\mathcal{M}_{\mathcal{T}}) := \left[\prod_{j\neq r} \int_{\mathcal{H}_j} d\mathcal{M}_j\right] f\Big(\bigcup_j \mathcal{M}_j\Big). \tag{36}$$

With the above definition, we can finally relate the full genetic prior to a product of evolution matrix elements and base frequencies π_a:

$$\int_{\mathcal{H}_{\mathcal{T}}} d\mathcal{M}_{\mathcal{T}}\, \mathcal{G}(\mathcal{T}|\boldsymbol{\theta}) = \prod_\ell \left[\pi^{(\ell)}_{s_r^{(\ell)}} \cdot \prod_{j\neq r} P^{(\ell)}_{s_i^{(\ell)}\, s_j^{(\ell)}}(t_j - t_i)\right]. \tag{37}$$

To complete the link between this work and Bayesian methods based on Felsenstein pruning, we first recast in our notation the traditional tree likelihood as computed via Felsenstein pruning [9]:

$$L_{\text{tree}}(\mathcal{T}_{\text{FT}}|\boldsymbol{\theta}) = \prod_\ell \left\{ \sum_{\substack{\{s_i^{(\ell)}\}\\ \text{fixed } \ell\\ \text{inner } i}} \left[\pi_{s_r^{(\ell)}} \cdot \prod_{j\neq r} P^{(\ell)}_{s_{\varphi(j)}^{(\ell)}\, s_j^{(\ell)}}(t_j - t_i)\right]\right\}. \tag{38}$$

The tree $\mathcal{T}_{\text{FT}}$ appearing on the left-hand side is the tree $\mathcal{T}$ stripped of its mutational history, i.e., the object that is usually tracked when using Felsenstein pruning. The outer product on the right-hand side is over all sites ℓ, so the factor in braces is the site likelihood of site ℓ. The inner sum expands into $N-1$ nested sums over the possible values of all states $s_i^{(\ell)}$ of all inner nodes i, for a fixed ℓ (the values of $s_i^{(\ell)}$ for tips i are simply the input sequences). The factor $\pi_{s_r^{(\ell)}}$ is the stationary base frequency of the state at site ℓ of the root node. The first innermost product is over all nonroot branches j and its factors are the matrix elements of the evolution matrix $P_{ab}^{(\ell)}(t)$.

By distributing the first product over the sum in Eq. (38), we obtain

$$L_{\text{tree}}(\mathcal{T}_{\text{FT}}|\boldsymbol{\theta}) = \sum_{\substack{\{s_i^{(\ell)}\}\\ \text{all } \ell\\ \text{inner } i}} \left\{\prod_\ell \left[\pi_{s_r^{(\ell)}} \cdot \prod_{j\neq r} P^{(\ell)}_{s_{A_j}^{(\ell)}\, s_j^{(\ell)}}(t_j - t_i)\right]\right\}. \tag{39}$$

Note that the sum in Eq. (38) expands into $N-1$ simpler nested sums, whereas that in Eq. (39) above expands into $(N-1)\times L$ simpler nested sums.

Substituting Equation (37) into the above, we immediately obtain the desired equivalence between the traditional tree likelihood on the one hand, and integrals over genetic histories of the product of the genetic prior and the likelihood on the other hand:

$$L_{\text{tree}}(\mathcal{T}_{\text{FT}}|\boldsymbol{\theta}) = \sum_{\substack{\{s_i^{(\ell)}\}\\ \text{all } \ell\\ \text{inner } i}} \int_{\mathcal{H}_{\mathcal{T}}} d\mathcal{M}_{\mathcal{T}}\, \mathcal{G}(\mathcal{T}|\boldsymbol{\theta}) \tag{40}$$

Similar considerations lead to the following link between the traditional site likelihood and integrals over genetic histories of the site genetic prior and the site likelihood:

$$L^{(\ell)}_{\text{tree}}(\mathcal{T}_{\text{FT}}|\boldsymbol{\theta}) = \sum_{\substack{\{s_i^{(\ell)}\}\\ \text{fixed } \ell\\ \text{inner } i}} \int_{\mathcal{H}^{(\ell)}_{\mathcal{T}}} d\mathcal{M}^{(\ell)}_{\mathcal{T}}\, \mathcal{G}^{(\ell)}(\mathcal{T}|\boldsymbol{\theta}).$$

The above derivations can be generalized straightforwardly in various ways, e.g., to account for site weight vectors at the tips, non-strict clocks, etc.

5 Dealing with missing data: `N`-pruning

In Section 4, we established that if we track all sites explicitly everywhere, then integrating the product of the genetic prior and the likelihood over all genetic histories yields the traditional tree likelihood. Throughout this paper, we argue that it's enormously beneficial to perform this integration statistically instead of analytically. However, in the presence of ambiguous tip sequences, a mixed strategy, which we call "`N`-pruning", retains almost all the benefits of the explicit approach while treating sequence ambiguity efficiently.

To illustrate the basic idea, consider the portion of a 1-site tree depicted in Figure 10(a). It includes 2 tips, A and B, their common ancestor, C, and another ancestor further removed, D. For clarity, we omit site superscripts in this 1-site example. Tip A is an `N` while tip B is a `T`. The traditional partial site likelihood at D is:

$$L_d = \sum_{a,c} P_{dc}(\tau_C) P_{ca}(\tau_A) P_{c\mathtt{T}}(\tau_B).$$

Note that we sum over all possible states a of tip A, but the state of tip B is fixed to `T`. The summation over a is simple to perform explicitly:

$$\begin{aligned} L_d &= \sum_{a,c} P_{dc}(\tau_C) P_{ca}(\tau_A) P_{c\mathtt{T}}(\tau_B), \\ &= \sum_{c} P_{dc}(\tau_C) P_{c\mathtt{T}}(\tau_B). \end{aligned}$$

This follows from $\sum_a P_{ca}(\tau_A) = 1$, i.e., the state c definitely evolves to *something* after a time τ_A. We now apply the Markov property of the evolution process, expressed as $\sum_c P_{dc}(\tau_C) P_{c\mathtt{T}}(\tau_B) = P_{d\mathtt{T}}(\tau_C + \tau_B)$, to evaluate the sum over c:

$$L_d = P_{d\mathtt{T}}(\tau_C + \tau_B).$$

Finally, observe that this is just the traditional partial site likelihood at D of the tree depicted in Figure 10(b), which is the result of "pruning" the branch that ended in an `N` from the original tree.

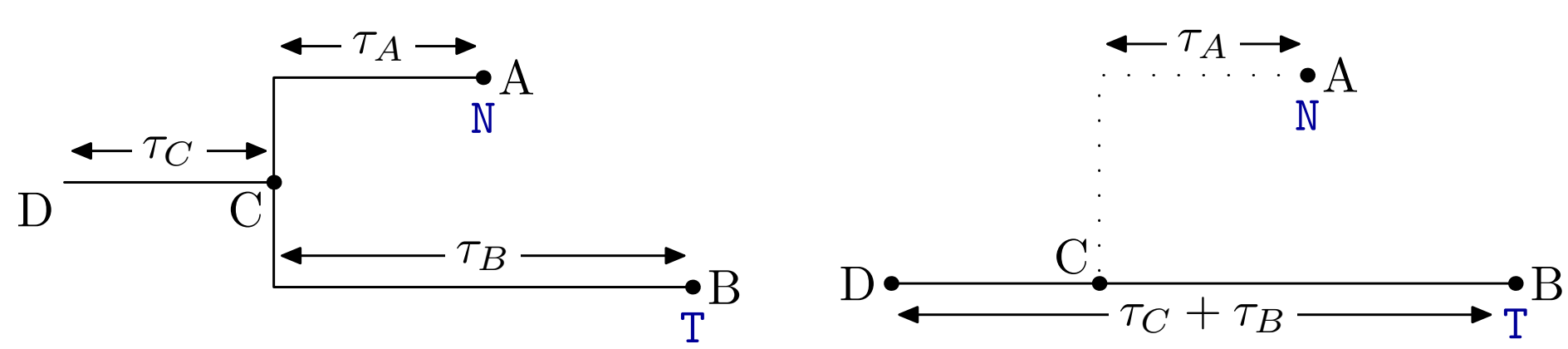

(a) Before (b) After

Supplementary Figure 10: `N`-pruning: Integrating over all possible histories of branch C–A is equivalent to pruning it off.

Clearly, the above pruning process can be repeated recursively to show that the site likelihood of a tree with ambiguous tips is equivalent to the site likelihood of the same tree with all subtrees ending in `N` tips pruned off. By the arguments of Section 4, this is equivalent to restricting the products, sums and integrals in Equation (8) according to $\xi(x)$. Thus, missations are revealed to be the pruning points at particular sites for each of the site trees that make up $\mathcal{T}$. The equivalence is sketched out in Figure 11.

In a very real sense, an EMAT with missations stands for all trees resulting from realizing all possible genetic histories below each missation. In particular, the space of all possible EMATs without missations is thus partitioned by EMATs with missations into sets, according first to topology, then to genetic histories

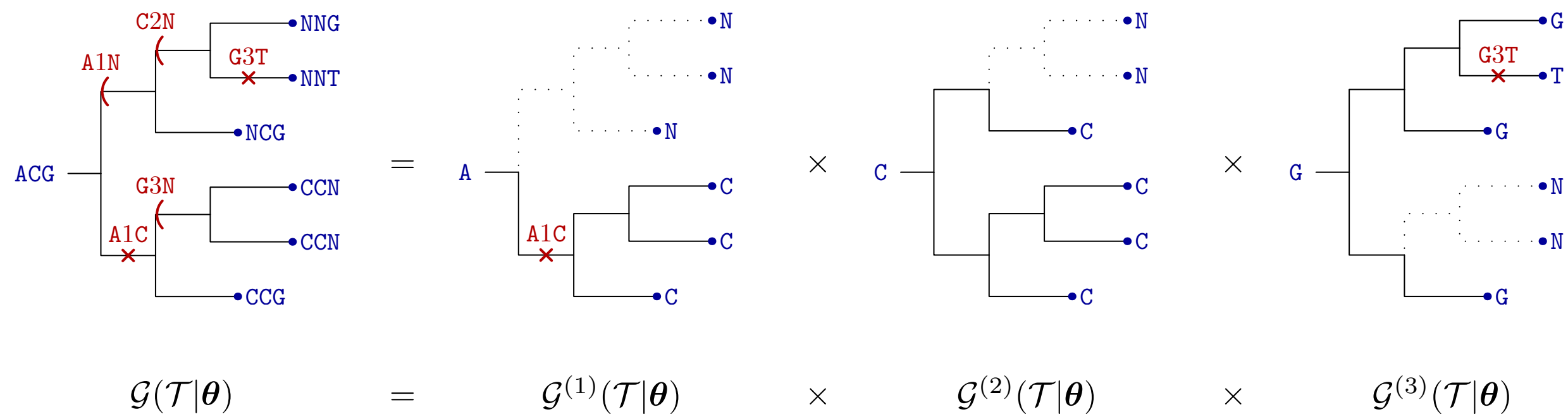

Supplementary Figure 11: N-pruning: A tree with "missations" decomposes into site trees, each of which has had its subtrees ending in N-tips pruned off. The genetic prior for the whole tree equals the product of site genetic priors for the pruned site trees.

above the missations. The posterior probability of an EMAT with missations is then sum of the posterior probabilities of the contained EMATs without missations. In order for this scheme to work, it is essential that any one EMAT without missations be "contained" in exactly one EMAT with missations, as indeed they are.

Empirically, we have found that very local MCMC moves are effective for EMATs with explicitly tracked sites everywhere when the tips are densely sampled, as is the case for SARS-CoV-2 and will likely be the case when understanding outbreaks and/or future pandemics. This is compatible with the observation that most trees linking such densely sampled datasets are nearly parsimonious [13, 16]. Applying N-pruning to all tips with missing data (converting partially ambiguous sites into totally ambiguous ones where necessary) effectively reduces the problem of sampling trees with missing data to that of sampling smaller trees without missing data, so very local MCMC moves are also effective there.

The alternative to implicit tracking of sites with data is to explicitly realize and modify genetic histories that lead up to tips with ambiguous state. Implicit tracking allows us to encode large-scale rearrangements with small structural changes. For example, in Figure 12, by far the most likely state of site 1 in all tips below X is A. An SPR move (Section 6.1) that detaches the subtree rooted at X from P and reattaches it at P' changes the most likely state at site 1 everywhere downstream of X to C, while only requiring very local rearrangements of the EMAT. In explicit tracking, local moves that do not perform this large-scale rearrangement are unlikely to be accepted because they introduce spurious additional mutations. We empirically verified that implementing only such local moves, e.g., an SPR move on tips that allows for the tip sequence to change where it is ambiguous, did not result in efficient sampling. While it is possible to improve the local moves we present in the following section to rearrange mutations in large clusters of the tree (perhaps inspired by the set of branches that an equivalent move in a parsimony tree would affect [42]), we have not yet explored this possibility fully. Certainly, N-pruning allows us to sidestep this issue for models where sites evolve independently and for datasets where sites are either fully determined by experiment or are completely ambiguous. This seems to cover the most common use cases we have seen for Bayesian phylogenetics. Nevertheless, a different approach would need to be developed to handle tip sites that are only partially ambiguous (i.e., for arbitrary weight vectors that model sequencer error), or to handle ambiguity when sites do not evolve independently (e.g., to correctly account for context-dependent APOBEC- or ADAR-induced mutations).

One final complication with missing data is that often a sequence will have large numbers of consecutive sites that are missing, forming "gaps". To take one concrete if arbitrary example, GenBank sequence OP612676, a nearly 200kb mpox sequence, has more than a dozen gaps each over 100 bases long, including one that is 5653 bases long. It's therefore essential to have an efficient representation of consecutive missations, different from a naive list with one entry per site. Further, efficient MCMC moves require knowledge of the state of a site preceding a missation (to implement the last term of the recurrence relation for λ_i in Equation (16)). Fortunately, for outbreak-like datasets, the probability that a random site differs in state

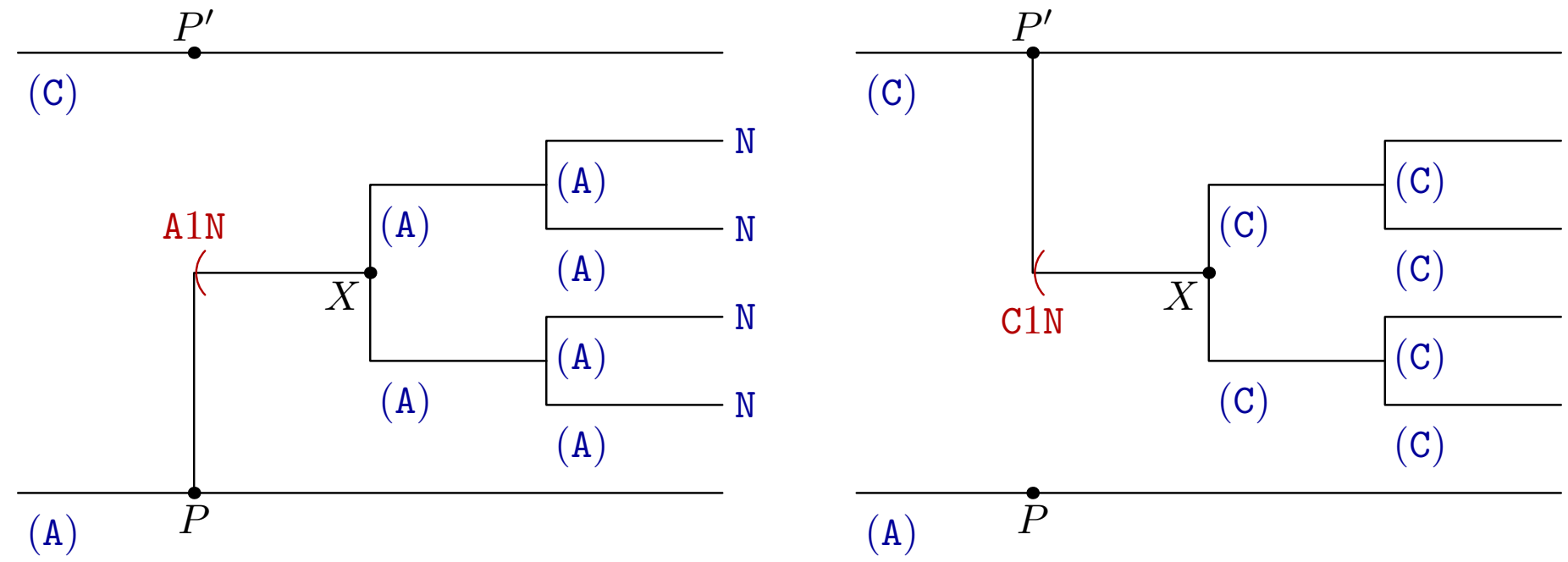

(a) Before (b) After

Supplementary Figure 12: N-pruning permits large-scale rearrangements with only local changes: By changing the single missation at the beginning of branch X from A2N to C2N, the implied sequences downstream of X all change from A to C.

at a point x on the tree from its state at the root node is relatively small (for example, after 4 years of SARS-CoV-2 evolution, a typical sequence differs from the Wuhan reference sequence NC_045512.2 at only 150 or so of its 30 000 sites). Since high-quality sequences rarely have more than a few percent of sites with missing data, the probability that a random *missing* site has a state different from the reference state is even smaller. Hence, inspired by MAPLE's representation for missing data [20], we represent Φ_i, the missations at the start of branch i, as a set of disjoint sorted intervals of sites that have no informative tips downstream of $\varphi(i)$, coupled with a sparse map from site ℓ to state $s_i^{(\ell)}$ for sites where this state differs from the reference state $s_\perp^{(\ell)}$. This representation admits efficient union and intersection operations, which we need to implement MCMC moves below, and the sparse map rarely contains more than a handful of entries. We further keep track of the cumulative sum of the terms $Q^{(\ell)}_{s_\perp^{(\ell)}}$, which allows us to implement the recurrence relation of Equation (16) even in the presence of multi-thousand base gaps.

We note briefly that the N-pruning scheme is not restricted to Bayesian phylogenetics. It may thus be possible to adapt parsimony and maximum likelihood approaches to use N-pruning with resulting efficiency improvements. Such an exploration is outside the scope of this paper.

Undoubtedly, N-pruning makes implementing MCMC moves on EMATs more complicated. We spell out all the details below.

6 Local MCMC moves

Here, we consider *local MCMC moves*, that is, moves that rearrange the tree $\mathcal{T}$ while keeping all the associated inference parameters $\boldsymbol{\theta}$ fixed. Hence, for clarity, we suppress the dependence on $\boldsymbol{\theta}$ on all quantities throughout this section.

A local move starts with an old configuration o of the tree and proposes a new configuration n with probability $\alpha(\mathsf{o} \to \mathsf{n})$. As usual, the move is accepted with a probability $P_{\text{acc}}(\mathsf{o} \to \mathsf{n})$ according to the Metropolis-Hastings criterion [12, 25],

$$P_{\text{acc}}(\mathsf{o} \to \mathsf{n}) = \min\left[1, \frac{P(\mathsf{n})}{P(\mathsf{o})} \frac{\alpha(\mathsf{n} \to \mathsf{o})}{\alpha(\mathsf{o} \to \mathsf{n})}\right]. \tag{41}$$

Here, $P(\mathcal{T})$ is the posterior of configuration $\mathcal{T}$, as given in Equation (3). Since the values of the parameters $\boldsymbol{\theta}$ are unchanged in local moves, the ratio of the posterior involves only ratios of the genetic and ancestral priors:

$$\frac{P(\mathsf{n})}{P(\mathsf{o})} = \frac{\mathcal{G}(\mathsf{n})}{\mathcal{G}(\mathsf{o})} \frac{\pi_{\text{anc}}(\mathsf{n})}{\pi_{\text{anc}}(\mathsf{o})}.$$

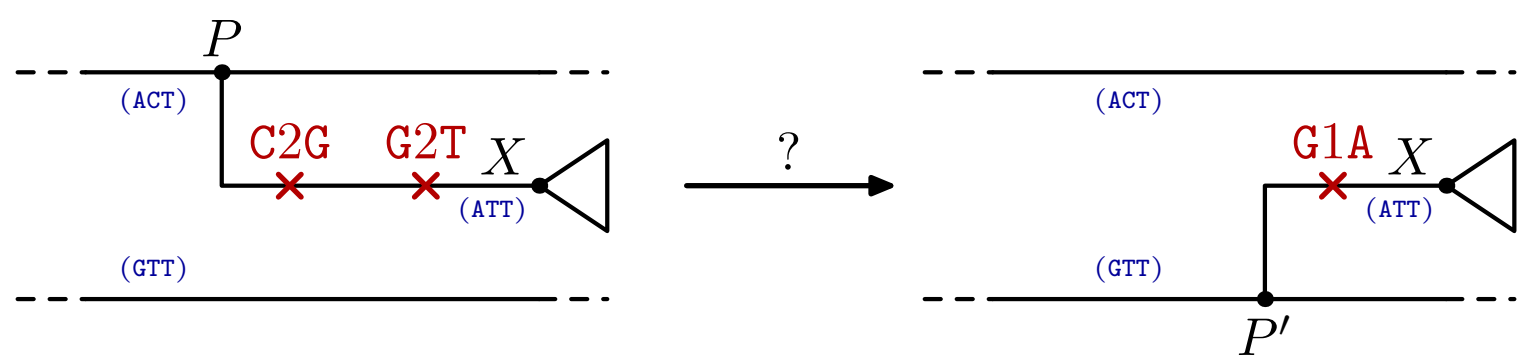

Supplementary Figure 13: A generic SPR move: a subtree rooted at X is attached to the rest of the tree at point P. An SPR move proposes detaching this subtree and reattaching it at a different point P'. The move includes a proposal for a new mutational history, at the very least covering the P'–X branch.

The ratio of ancestral priors is easily calculated for all local moves, whereas calculating the ratios of proposal probabilities and genetic priors involves many subtleties. We describe the latter two calculations in detail below for all the local MCMC moves that we have implemented.

6.1 Subtree Pruning and Regrafting (SPR) moves

Delphy implements a subtree pruning and regrafting move that is conceptually related to Nielsen's original SPR move [28]. The main differences are: (a) the choice of grafting point is made general, with some specific choices detailed below; (b) the mutation proposal is adapted for efficiency for large genomes with few mutations per branch; (c) the move is generalized to arbitrary evolution models (not necessarily time-reversible); and (d) the move efficiently handles tip sequences with missing data.

We first describe the move when all data is present and the root node is not changing, then show how these restrictions are relaxed in the subsequent sections.

A general SPR move is depicted in Figure 13. A subtree rooted at X, grafted at P in the old configuration o, is to be regrafted at another point P' in the new configuration n. The proposal, picked with probability $\alpha(\mathsf{o} \to \mathsf{n})$, consists of choosing a new grafting point P' with probability $\alpha_{\text{graft}}(\mathsf{o} \to \mathsf{n})$, followed by a new mutational history for the tree compatible with the tip sequences, picked with probability $\alpha_{\text{mut}}(\mathsf{o} \to \mathsf{n})$. We take $\alpha_{\text{graft}}(\mathsf{o} \to \mathsf{n})$ as given and focus on the second step here.

At one extreme, we could consider proposing a new mutational history for the entire tree via stochastic mapping [27]. The probability $\alpha_{\text{mut}}(\mathsf{o} \to \mathsf{n})$ is then proportional to $\mathcal{G}(\mathsf{n})$, and by construction, the normalization constant is the usual Felsenstein pruning tree likelihood $\mathcal{L}_{\text{tree}}(\mathsf{n})$ (see Section 4). This approach yields the following acceptance probability:

$$p_{\text{acc}} = \min\left[1, \frac{\mathcal{L}_{\text{tree}}(\mathsf{n})\pi_{\text{anc}}(\mathsf{n})}{\mathcal{L}_{\text{tree}}(\mathsf{o})\pi_{\text{anc}}(\mathsf{o})} \bigg/ \frac{\alpha_{\text{graft}}(\mathsf{o} \to \mathsf{n})}{\alpha_{\text{graft}}(\mathsf{n} \to \mathsf{o})}\right], \qquad \text{(full stochastic mapping).}$$

This is nothing but the standard acceptance probability for SPR moves when using Felsenstein pruning, as implemented in BEAST and other similar tools. In the explicit mutation representation, we thus see that these moves correspond to performing stochastic mapping over the entire tree after every single move, which hints at why this operation is relatively expensive.

At the other extreme, we could consider proposing a new mutational history which is identical to the old one except for the the branch connecting X to P or P'. In such a scheme, the sequence at X, P and P' are the same in the old and new configurations. By walking in the old configuration along the path from X to P and then to P', we can enumerate the $M(\mathsf{n})$ differences in the sequences of X and P' in time proportional to the number of mutations along that path, which we expect to be very small. An "optimistic" parsimony-inspired scheme for proposing a new mutational history from P' to X would be to omit any mutations in sites where the sequences of X and P' agree, and propose a single mutation where the sequences disagree, distributed uniformly over a time $t_X - t_{P'}$. Such a scheme has:

$$\alpha_{\text{mut}}(\mathsf{o} \to \mathsf{n}) = \left(\frac{1}{t_X - t_{P'}}\right)^{M(\mathsf{n})}, \qquad \text{(optimistic scheme).} \tag{42}$$

The optimistic scheme works and is efficient as long as the mutational history of the P–X branch in the old configuration has at most one mutation per site. In particular, note that the cost of the proposal scales as the number of mutations along the X–P–P' path, which is typically of order unity, not the genome size, which can easily exceed several thousands for real-world datasets. However, if the P–X branch in the old configuration has 2 or more mutations on the same site, then the proposal probability of the reverse move is zero, and the SPR move is always rejected. Hence, the fatal flaw of the optimistic scheme is that, in the absence of more sophisticated moves, the MCMC lacks a mechanism for modifying the tree around a branch with multiple mutations on the same site.

One way to improve the optimistic scheme is to apply stochastic mapping along the single P' to X branch given its fixed endpoints, using the site-specific evolution model for each site (this is the essence of Nielsen's original SPR move [28]). In that case, the probability of generating the specific configuration for site ℓ is proportional to $\mathcal{G}_X^{(\ell)}(\mathsf{n})$, with a normalization constant that can be calculated explicitly in terms of evolution matrix elements:

$$\alpha_{\text{mut}}(\mathsf{o} \to \mathsf{n}) = \frac{\prod_\ell \mathcal{G}_X^{(\ell)}(\mathsf{n})}{\prod_\ell P^{(\ell)}_{s_{P'}^{(\ell)} s_X^{(\ell)}}(t_X - t_{P'})}, \qquad \text{(exact stochastic mapping on } P'\text{–}X \text{ branch)}.$$

In this scheme, the ratios of site-branch genetic priors $G_X^{(\ell)}(\mathsf{n})/G_X^{(\ell)}(\mathsf{o})$ exactly cancel the ratio of genetic priors $\mathcal{G}(\mathsf{n})/\mathcal{G}(\mathsf{o})$. However, since each site ℓ in principle has a different transition rate matrix $Q_{ab}^{(\ell)}$, the stochastic mapping needs to be performed site by site, so the cost of the proposal scales as the genome size. Moreover, the state at P' must be reconstructed at each site, which needs a walk up to the root node of the tree. These are both serious flaws.

A simple adaptation tames the high cost of the above scheme. First, note that whenever $\mu(t_X - t_{P'}) \ll 1$, the parsimonious solution of the optimistic scheme dominates regardless of the details of the evolution model. All that is really required is a scheme that exhibits a similar dominance but that also allows, in principle, for any mutational history to be proposed. To this end, we use a simple Jukes-Cantor model with a fictitious mutation rate $\tilde{\mu} = \lambda(X)/L$ and no site-rate heterogeneity. Let $P_=^{JC}(t)$ and $P_{\neq}^{JC}(t)$ be the evolution matrix elements of this model when the endpoints respectively do or do not match, given by the well-known formulas

$$P_=^{JC}(t) = (1/4)(1 + 3e^{-(4/3)\tilde{\mu}t}) \approx 1 - \tilde{\mu}t;$$
$$P_{\neq}^{JC}(t) = (1/4)(1 - e^{-(4/3)\tilde{\mu}t}) \approx (\tilde{\mu}/3)t.$$

The approximations hold in the limit of $\tilde{\mu}t \ll 1$. Since we use the same model for every site, and multiple mutations in sites with equal states at the endpoints of a short branch are very rare, we can perform the stochastic mapping using this model roughly in time proportional to $M(\mathsf{n})$ (see below). The final proposal probability is given in terms of $M(\mathsf{n})$, the actual number $\mathcal{M}(\mathsf{n})$ of mutations proposed, and the above evolution matrix elements, as follows:

$$\alpha_{\text{mut}}(\mathsf{o} \to \mathsf{n}) = \frac{e^{-\tilde{\mu}L(t_X - t_{P'})}(\tilde{\mu}/3)^{\mathcal{M}(\mathsf{n})}}{[P_{\neq}^{JC}(t_X - t_{P'})]^{M(\mathsf{n})}[P_=^{JC}(t_X - t_{P'})]^{L-M(\mathsf{n})}},$$
$$\text{(approximate stochastic mapping on } P'\text{–}X \text{ branch)}.$$

Note that in the limit where $\tilde{\mu}(t_X - t_{P'}) \ll 1$ and $\mathcal{M}(\mathsf{n}) = M(\mathsf{n})$, the above proposal probability reduces to that of the optimistic scheme (Eq. (42)), which is independent of $\tilde{\mu}$.

6.2 Efficient Jukes-Cantor stochastic mapping in short branches

We first consider the simpler problem of performing a stochastic mapping on a branch of length τ with a Jukes-Cantor model having mutation rate $\tilde{\mu}$, and where the starting and ending state at all L sites is `A`. For sufficiently small $\tilde{\mu}\tau$, we expect this procedure to result in no mutations at all with overwhelming likelihood, so we arrange the algorithm to produce that result efficiently when necessary. At the end of this section,

we discuss the refinements needed to implement stochastic mapping when a few sites have different starting and ending states, and the other sites' equal starting and ending states are not all `A`.

Conceptually, proposing L independent trajectories from `A` to `A` over a time τ proceeds via L repetitions of a variation of Nielsen's rejection sampling algorithm [27]. A trajectory is first proposed as follows: the number of mutations on the trajectory is drawn from a Poisson distribution with mean $\tilde{\mu}\tau$, with times distributed uniformly from 0 to τ, and with identities chosen at random (subject to the end state of one mutation matching the start state of the next mutation). The trajectory is accepted if the state at time τ is `A`, otherwise it is rejected and the algorithm begins anew. The course of the algorithm is generally as follows. If zero mutations are proposed, which happens with probability $p_0 = e^{-\tilde{\mu}\tau}$, the trajectory is empty and is immediately accepted. If one mutation is proposed, which happens with probability $p_1 = \tilde{\mu}\tau e^{-\tilde{\mu}\tau}$, the trajectory necessarily ends in a state other than `A` and is rejected. Otherwise, the acceptance or rejection of the trajectory depends on the details of the proposed mutation identities.

Executing the above procedure directly is expensive and wasteful. Overwhelmingly, the above procedure simply proposes and rejects a one-mutation trajectory k times, with $k = 0$ almost always, and then proposes a zero-mutation trajectory, which is accepted. This observation suggests that we *simulate* the above procedure by deciding up front whether or not it ends in an acceptance of an empty trajectory before any trajectory with 2 or more mutations is proposed. The probability that no trajectory with 2 or more mutations is proposed before a zero-mutation proposal is accepted is

$$p_* = \sum_{k=0}^{\infty} p_1^k p_0 = \frac{p_0}{1 - p_1}$$

Hence, we can directly produce an empty trajectory for a site with probability p_*. With probability $1 - p_*$, we instead directly make a proposal with 2 or more mutations: if it ends in `A`, we accept it; otherwise, we repeat this procedure. Finally, instead of doing this independently for each site, we can draw a number of sites from $\mathrm{Geom}(1 - p_*)$ distribution to determine for how many sites we trivially propose an empty trajectory before we have to resort to more detailed sampling. The final algorithm is presented in Algorithm 1, which we note must be implemented carefully to avoid numerical issues with small p_* and large numbers drawn from $\mathrm{Geom}(1 - p_*)$.

For a typical genomic epidemiology tree of a SARS-CoV-2-like virus, we have $\tilde{\mu} \approx 10^{-3}\,\mathrm{yr}^{-1}$, $L \approx 30\,000$ and $\tau \approx 2\,\mathrm{weeks}$, whereby $1 - p_* \approx 7 \times 10^{-10}$. The probability that the initial draw from $\mathrm{Geom}(1 - p_*)$ doesn't immediately skip over all L sites is $1 - (1 - p_*)^L \approx 2 \times 10^{-5}$. Even for a much longer branch with $\tau = 1\,\mathrm{year}$, this probability only rises to around 1.5 %. Hence, overwhelmingly, this procedure immediately produces an empty mutational trajectory for all L sites after drawing a single random number.

Two further refinements are needed to apply the above ideas to complete the proposal of the mutational history between P' and X. First, we need to treat differently the $M(\mathsf{n})$ sites where the states at P' and X disagree. Since these are few in number, and their discovery unearths the actual states of that site at P' and X, we apply a small variation on Nielsen's rejection sampling scheme directly, site by site: we repeatedly choose a Poisson-distributed number of mutations n, subject to $n \geq 1$, produce random mutation identities and times as above, and accept the proposal only if the start and end states agree with the actual states at P' and X. When we then use Algorithm 1 to effect a stochastic mapping of the remaining sites, if we stop at a site ℓ that where P' and X differ, we immediately skip over this site; this is equivalent to first executing stochastic mapping over all L sites, then filtering out the mutations on the $M(\mathsf{n})$ sites where the states at P' and X differ, which itself is equivalent to performing the detailed stochastic mapping only on the $L - M(\mathsf{n})$ sites where starting and ending states are equal. Finally, in the unlikely case that this stochastic mapping does introduce mutations in sites where P' and X agree, we must discover the actual start and end state of the site, which is unlikely to be `A`, by looking for the nearest mutation at that site on the path from P' to the root node of the tree; if no such mutation exists, we read off the state from the reference sequence. With the start and end states known, we then cyclically rotate the states on the proposed mutational history until the start and end states match the known state. For example, if the start and end states of site 1 are `G` and the proposed mutational history was `A1G`, followed by `G1T`, followed by `T1A`, the cyclic rotation modifies the proposal to `G1A`, followed by `A1C`, followed by `C1G`.

Algorithm 1 Efficient stochastic mapping for a branch of length τ using a Jukes-Cantor model with mutation rate $\tilde{\mu}$, with starting and ending states `A` at all L sites.

```
p_0 ← e^{-μ̃τ}
p_1 ← μ̃τ e^{-μ̃τ}
p_* ← p_0/(1 − p_1)
ℓ ← 0
while ℓ < L do
    Δ ~ Geom(1 − p_*)
    ℓ ← ℓ + Δ
    if ℓ < L then
        n ~ KPois(μ̃τ, 2)                ▷ KPois(λ, k) is the restriction of Pois(λ) to at least k events.
        s_0 ← A
        for i ← 1 to n do
            s_i ~ DUnif({A, C, G, T} \ {s_{i−1}})
        end for
        if s_n = A then                  ▷ Acceptance of non-empty mutational history
            t_1 ≤ ... ≤ t_n ~ Unif(0, τ)
            Output mutations s_0ℓs_1 at t_1, ..., s_{n−1}ℓs_n at t_n
            ℓ ← ℓ + 1
        else
            (Rejection sampling starts anew at site ℓ, not ℓ + 1).
        end if
    end if
end while
```

6.3 SPR moves involving root node changes but no missing data

An SPR move can cause the root node of the tree to change if P is the root node of the old configuration o and/or if P' is the root node of the new configuration n. We focus here on the latter scenario, as the treatment of the former one is completely analogous. In this section, we are still restricted to sample sequences with no missing data.

Since an EMAT does not explicitly represent the mutational history of the lineage ancestral to the root node, the SPR proposal needs to include these details. Concretely, let S be the sibling of X in the old configuration o, and let S' be the sibling of X in the new configuration n, as depicted in Figure 14. Delphy's SPR move in this case does not consider the sequence at P' to be fixed; instead, the mutational history along the entire X–P'–S' path is proposed afresh, subject to the sequences at X and S' being fixed. To do this, we exploit the time-reversibility of the Jukes-Cantor model to propose a single trajectory of length $\tau' = (t_X - t_{P'}) + (t_{S'} - t_{P'})$ with the end sequences of X and S'. Although we're exploiting the time-reversibility of the Jukes-Cantor model, note that the actual evolution model used in the posterior distribution need not be time-reversible. The proposal probability given by:

$$\alpha_{\text{mut}}(\mathsf{o} \to \mathsf{n}) = \frac{e^{-\tilde{\mu}L\tau'}(\tilde{\mu}/3)^{\mathcal{M}(\mathsf{n})}}{[P^{JC}_{\neq}(\tau')]^{M(\mathsf{n})}[P^{JC}_{=}(\tau')]^{L-M(\mathsf{n})}}, \qquad \text{(when } P' \text{ is the root node of the new configuration).}$$

Note that the ratio of genetic priors in the acceptance probability (Eq. (41)) includes a ratio of root priors between the old and new configurations (see below for details). This ratio can be evaluated efficiently in time proportional to the number of mutations proposed along the P' to S' branch in the new configuration and/or the P to S branch in the old configuration.

A useful viewpoint for the remainder of this section is to view the root node of an EMAT as missing data *upstream* at every site, as opposed to downstream at some sites towards the tips, so that the sequence at P' is not highly constrained by the three branches that impinge on it. The way the above scheme deals with

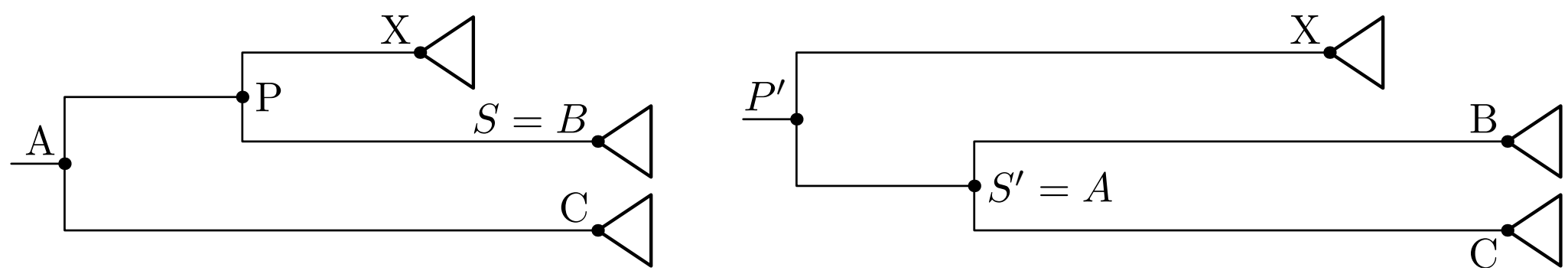

(a) Before (b) After

Supplementary Figure 14: An SPR move that changes the root node. Whereas the mutational history along the A–B path can remain fixed, a new mutational history must be proposed for the entire X–P'–S' path.

such missing data is by extending the path over which mutations are proposed from X–P' to X–P'–S' for all sites, and stopping at S' because the sequence at S' *is* strongly constrained by those of the two children of X and S'. This viewpoint is expanded in the sections below on how Delphy deals with missing data.

6.4 SPR moves involving missing data but no root node changes

Consider the situations in Figures 15(a) and (b) involving a dataset with $L = 100$ sites. The subtree rooted at X is detached at P from the rest of the tree, with the entire subtree, its mutational history and the sequence at X held fixed in sites where at least one downstream tip is informative. In the original configuration, the branch leading to X is decorated with missations on sites 10–20, while the one leading to X's sibling S is decorated with missations on sites 30–40. These missations imply that at least one of the tips downstream of X *is* informative for sites 30–40, and analogously, at least one of the tips downstream of S is informative for sites 10–20 (otherwise, the corresponding missations would appear further upstream). When X is detached, the points immediately upstream of P no longer have downstream tips that are informative for sites 30–40. It follows that the corresponding missations will be forced to appear further upstream. Any mutations at sites 30–40 on the branch above P, such as the `G33T`, must not appear on the tree after X is detached. This process continues recursively towards the root node: while in the original configuration, P's sibling has missations on sites 35–45, the subset of those missations on sites 35–40 are forced to appear further upstream, removing the `C37G` mutation. This example captures the essential complication of implementing SPR moves in the presence of missing data. In a very concrete way, parts of the path from X all the way to the root node are "linked" to the attachment of X at P. SPR moves in the presence of missing data must consider these paths and the mutations on them in their proposal and evaluation of their Metropolis-Hastings factor.

To discuss the move in detail, we first introduce helpful notation. Let $\phi_i := \varphi^i(X)$ be the ith ancestor of X, with $i = 0, \ldots, I$. It follows that ϕ_0 is X and ϕ_1 is P; as for I, it is chosen such that ϕ_I is the root node. In this section, we assume X is not a child of the root node, so that I is at least 2; we relax that restriction in the next section. For i positive, denote by "branch i" the branch from ϕ_i to ϕ_{i-1}. Likewise, denote by "path i" the path from ϕ_i to X, which has length T_i given by $T_i = t_X - t_{\phi_i}$. Next, denote by S_i the child of ϕ_i that is not on path i. Denote by $\mathcal{N}_i$ the missations on the branch from ϕ_i to S_i.

We first identify the sets of sites $\tilde{\mathcal{L}}_i$ along branch i that are "linked" to the attachment of X at P. We say that these sites are "warm" on branch i. Clearly

$$\tilde{\mathcal{L}}_i = \tilde{\mathcal{L}}_{i-1} \cap \mathcal{N}_{i-1}, \qquad (2 \le i \le I+1).$$

The base case of this recurrence is $\tilde{\mathcal{L}}_1 = \xi(X)$, i.e., the set of sites present at X. We discuss the meaning of $\tilde{\mathcal{L}}_{I+1}$ below, but for now assume that it is empty. For example, in the scenario of Figure 15(a), but with no missations on the lower branch from the root node, we have $\tilde{\mathcal{L}}_1 = [1,9] \cup [21,100]$, $\tilde{\mathcal{L}}_2 = [30,40]$, $\tilde{\mathcal{L}}_3 = [35,40]$ and $\tilde{\mathcal{L}}_4 = \emptyset$. We re-examine the full scenario with $\tilde{\mathcal{L}}_4 \neq \emptyset$ at the end of this section. In general, calculating $\tilde{\mathcal{L}}_1$ involves an undesirable walk from X all the way to the root node to enumerate all the sites for which there is a missation on path I. We can almost always avoid this calculation by noting that if $\ell \in \mathcal{N}_1$, it is necessarily present along the branch P–X, and is thus a member of $\tilde{\mathcal{L}}_1$. Hence, $\tilde{\mathcal{L}}_2 = \mathcal{N}_1$, and

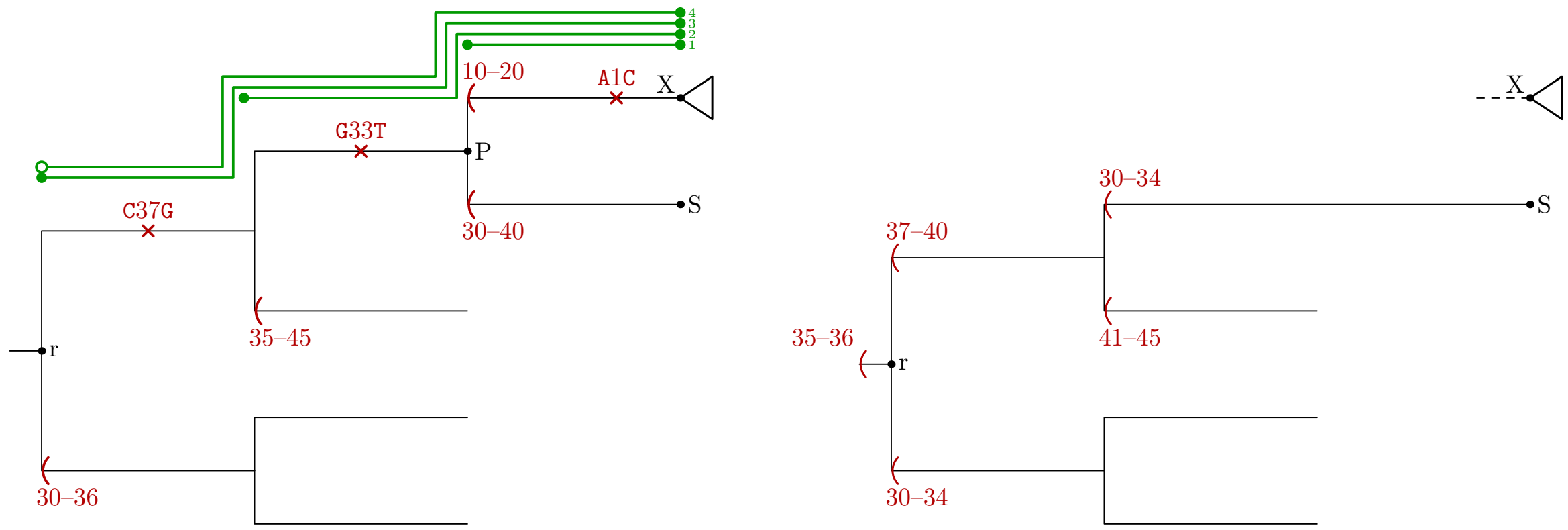

(a) Before pruning X (b) After pruning X

Supplementary Figure 15: The pruning step of an SPR move in the presence of missations. The paths labeled 1 through 4 all need to be treated differently. Paths 3 and 4 are distinguished by whether the state at the root is unchanged (sites 37–40) or resampled (sites 25–26). See text for details.

in general,

$$\tilde{\mathcal{L}}_i = \mathcal{N}_{i-1} \cap \cdots \cap \mathcal{N}_1, \qquad (2 \leq i \leq I+1).$$

These subsets, represented as sorted lists of intervals, can be calculated efficiently in time proportional to the number of such intervals because missations are themselves represented as sorted lists of intervals. We'll see below that we usually only need to know the size of $\tilde{\mathcal{L}}_1$, not its exact contents. We also note that very often, the above recurrence often reaches a point where $\mathcal{L}_i = \emptyset$ for small i, so we often don't need to walk more than a few branches upstream of X in the calculations below (in the extreme case, when there are no missations on the branch from P to S, we have $\mathcal{L}_2 = \emptyset$ already, and the entire scheme here reduces to that presented in Section 6.1).

We further define a set of "hot" sites $\mathcal{L}_i$ on path i as follows:

$$\mathcal{L}_i := \tilde{\mathcal{L}}_i \setminus \tilde{\mathcal{L}}_{i+1}, \qquad (1 \leq i \leq I).$$

For example, in Figure 15(a) but without the missations on the lower branch from the root node, we have $\mathcal{L}_1 = [1, 9] \cup [21, 29] \cup [41, 100]$, $\mathcal{L}_2 = [30, 34]$ and $\mathcal{L}_3 = [35, 40]$. The sets $\mathcal{L}_1, \ldots, \mathcal{L}_I$ partition the set of sites $\tilde{\mathcal{L}}_1$ that are present at X such that a site ℓ is in $\mathcal{L}_i$ if it is warm on path i but not on path $i+1$. When viewed in terms of the pruned site-trees (Figure 11), path i corresponds to the branch upstream of X for the site-ℓ pruned tree for which ℓ is hot in path i.

The SPR move proposal is as follows. For every path i, we hold the sequences and mutational histories of sites $\mathcal{L}_i$ fixed everywhere except in the interior of path i. We then use the scheme described in Section 6.2 to propose new mutational histories along the entirety of path i, with length T_i, subject to fixed end states, with fake mutation rate $\tilde{\mu} = \lambda_X / |\xi(X)|$. The set of sites that are hot in path i and have different end states is small and easy to construct while calculating $\mathcal{L}_i$. For the remaining sites, we expect the probability of even a single non-parsimonious mutation to appear to be very small, so we simply run Algorithm 1 over all L sites, then filter the result to include only sites in $\mathcal{L}_i$; in the exceedingly unlikely case that the result is not empty, we walk up the tree to deduce the actual state of site i at ϕ_i, as in Section 6.2. The resulting proposal probability is

$$\alpha_{\text{mut}}(\mathsf{o} \to \mathsf{n}) = \prod_{i=1}^{I} \frac{e^{-\tilde{\mu}|\mathcal{L}_i|T_i} (\tilde{\mu}/3)^{\mathcal{M}_i(\mathsf{n})}}{[P^{JC}_{\neq}(T_i)]^{M_i(\mathsf{n})} [P^{JC}_{=}(T_i)]^{|\mathcal{L}_i| - M_i(\mathsf{n})}},$$

(approximate stochastic mapping on hot sites in paths above X).

In analogy to the situation without missing data, we have that $|\mathcal{L}_i|$ is the number of hot sites on path i, T_i is the path's length, $\mathcal{M}_i(\mathsf{n})$ is the actual number of proposed mutations on hot sites of path i and $M_i(\mathsf{n})$ is

the number of hot sites on path i whose start and end state differ. As hinted above, this formula requires us to know $|\mathcal{L}_1| = |\xi(X)|$, i.e., the number of sites present at node X, but rarely its exact membership, which is expensive to calculate (the exception occurs when a hot site on path 1 accumulates mutations when the start and end states are equal, which we must filter to exclude sites that are missing at X). We keep track $|\xi(X)|$ for all nodes X separately, and update it progressively during all tree topology changes.

To calculate the ratio of genetic priors in the new and old configurations, we actually calculate a ratio of ratios of the genetic priors between each configuration with X detached or attached (the situations in Figures 15(a) and (b)). Denote by $\mathcal{G}'(\mathsf{n})$ (respectively, $\mathcal{G}'(\mathsf{o})$) this latter ratio for the new (respectively, old) configuration. Since only the hot sites along each path i change when detaching X, the calculation resembles the analogous calculation when there is no missing data. In particular,

$$\mathcal{G}' = \prod_{i=1}^{I} \left[e^{-\int_{x \in \text{ path } i} \lambda_i(x)\, dx} \prod_{(a,\ell,b)\in\mathcal{M}_i} Q_{ab}^{(\ell)} \right].$$

Here, $\lambda_i(x) = \sum_{i\in\mathcal{L}_i} Q^{(\ell)}_{s_i^{(\ell)}(x)}$, that is, the contribution to $\lambda(x)$ from the hot sites of path i, while $\mathcal{M}_i$ are the mutations on hot sites along path i. Calculating this quantity given $\mathcal{L}_i$, represented as a sorted list of intervals, is efficient because, as noted earlier, we keeping a cumulative sum of all terms $Q^{(\ell)}_{s_\perp^{(\ell)}}$ for the reference sequence, and the reference sequence rarely changes. The value of $\lambda_i(\phi_i)$ if the sequence at ϕ_i were the reference sequence follows straightforwardly. To adjust it to the actual sequence at ϕ_i, we note that all sites which are hot on path i are also in the missations $\mathcal{N}_i$, and those missations record state differences with the reference sequence (of which there are usually few) explicitly.

For completeness, the acceptance probability of the SPR move in the presence of missing data is

$$p_{\text{acc}} = \min\left[1, \frac{\mathcal{G}'(\mathsf{n})}{\mathcal{G}'(\mathsf{o})} \Big/ \frac{\alpha_{\text{mut}}(\mathsf{o}\to\mathsf{n})\alpha_{\text{graft}}(\mathsf{o}\to\mathsf{n})}{\alpha_{\text{mut}}(\mathsf{n}\to\mathsf{o})\alpha_{\text{graft}}(\mathsf{n}\to\mathsf{o})}\right].$$

One final complication arises when there are sites ℓ for which only the tips downstream of X are informative. These are precisely the members of $\mathcal{L}_{I+1}$, which we assumed empty above. One possibility would be to *define* $\mathcal{L}_{I+1} := \emptyset$, which leads to proposals where the state of the root node at these sites is fixed. If we proceeded thus, and the current states of a site ℓ at the root node and at X were different, then the SPR move would have no mechanism of proposing a mutation-free path for site ℓ, which would change the state of site ℓ at the root node to its state at X; this is unfortunate because in the near-parsimonious limit, that is overwhelmingly the most likely configuration. So, instead, we treat sites in $\tilde{\mathcal{L}}_{I+1}$ specially. First, we declare them to be hot by definition, that is:

$$\mathcal{L}_{I+1} := \tilde{\mathcal{L}}_{I+1}.$$

Then, the SPR mutational history proposal for a site $\ell \in \mathcal{L}_{I+1}$ consists of a simple open-ended trajectory with end state $s_X^{(\ell)}$ (in Figure 15(a), this is indicated by the open circle at the end of path 4). We construct this trajectory backwards, starting at time t_X and repeatedly drawing exponentially distributed times between successive mutations at a rate $\tilde{\mu}$ until the next mutation time precedes the root node time. For each mutation time, we pick a corresponding site ℓ uniformly over all sites L, and discard it unless the site is in $\mathcal{L}_{I+1}$. On the rare occasions when one mutation in $\mathcal{L}_{I+1}$ is proposed, the reference sequence, the mutations on branch X and the missations $\mathcal{N}_1$ are used to reconstruct $s_X^{(\ell)}$, and then a mutation identity is chosen uniformly at random. The state at ℓ is recorded and the algorithm proceeds further until the next proposed mutation occurs at a time $t < t_r$. The proposal probability gets an additional term, as follows:

$$\alpha'_{\text{mut}}(\mathsf{o}\to\mathsf{n}) = \alpha_{\text{mut}}(\mathsf{o}\to\mathsf{n}) \cdot e^{-\tilde{\mu}|\mathcal{L}_{I+1}|T_I} (\tilde{\mu}/3)^{\mathcal{M}_{I+1}(\mathsf{n})}.$$

The time of the open-ended trajectory is T_I (there is no T_{I+1}) and there is no denominator in the extra factor because the trajectory is open-ended. Conversely, the genetic prior ratio $\mathcal{G}'$ is modified to reflect the change in root node state for each $\ell \in \mathcal{L}_{I+1}$ to that at X once X is detached:

$$\mathcal{G}'' = \mathcal{G}' \cdot e^{-\int_{x\in\text{path } I} \lambda_{I+1}(x)\, dx} \prod_{(a,\ell,b)\in\mathcal{M}_{I+1}} Q_{ab}^{(\ell)} \cdot (\pi_a^{(\ell)}/\pi_b^{(\ell)}).$$

We close this section by noting that the book-keeping burden of updating missations and the differences between their "from" states and the reference sequence as trees are pruned and regrafted elsewhere is nontrivial but tractable. Since the details of such book-keeping are not particularly illuminating, we omit them here.

6.5 SPR moves involving root node changes and missing data

Consider the situation in Figure 16. Here, a subtree rooted at X is directly attached to the root node of the tree. Detaching X also removes the branch from the root node to its sibling S. Doing so is complicated by the presence of missations on either the P–X or the P–S branch. However, unlike in the previous section, the missations upstream of the X, P and S nodes suffice to build the sets of sites with no informative tips downstream of these three nodes. Hence, we can implement a variant of the scheme of the previous section in terms of three paths: a path S–P–X with fixed endpoints, and two open-ended paths P–X and P–S.

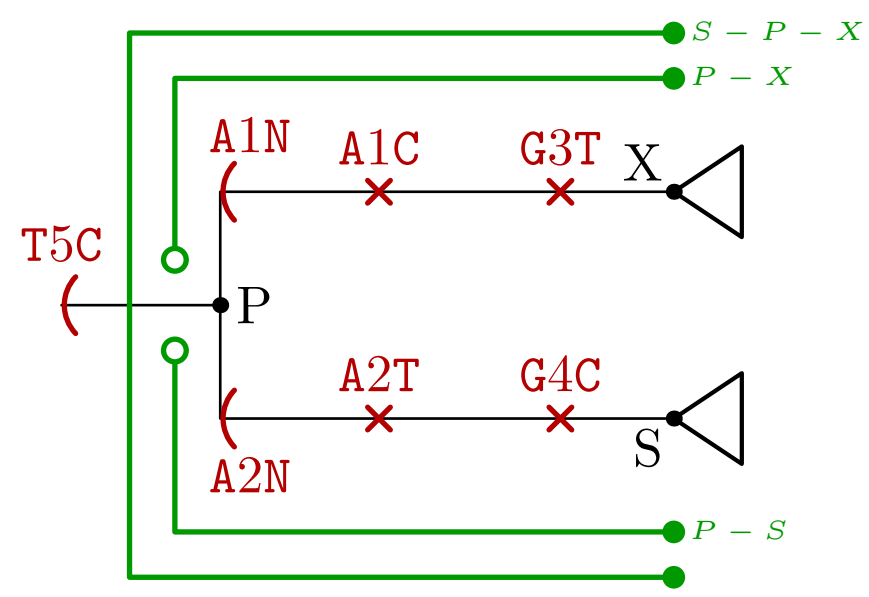

Supplementary Figure 16: The situation preceding the pruning step of an SPR move when the pruned subtree is a direct child of the root node and there are missations around the root node. The paths labeled $S - P - X$, $P - X$ and $P - S$ all need to be treated differently. See text for details.

Denote by $\mathcal{N}_X$, $\mathcal{N}_P$ and $\mathcal{N}_S$ the missations on the branches ending at nodes X, P and S, respectively. Denote by $\mathcal{P}_i$ the set of sites present at node i, i.e., for which there is at least one informative tip downstream of i. Clearly, we have

$$\begin{aligned} \mathcal{P}_P &= \{1, \ldots, L\} \setminus \mathcal{N}_P; \\ \mathcal{P}_X &= \mathcal{P}_P \setminus \mathcal{N}_X; \\ \mathcal{P}_S &= \mathcal{P}_S \setminus \mathcal{N}_S. \end{aligned}$$

Each of the sites present at P is next classified as a hot site in one of the paths S–P–X, P–X and P–S. Concretely,

$$\begin{aligned} \mathcal{L}_{S-P-X} &= \mathcal{P}_P \setminus (\mathcal{N}_X \cup \mathcal{N}_S); \\ \mathcal{L}_{P-X} &= \mathcal{N}_S; \\ \mathcal{L}_{P-S} &= \mathcal{N}_X. \end{aligned}$$

The SPR mutation history proposal treats each of these three paths separately, as described below.

For the S–P–X path, we again use a variation of the scheme described in Section 6.2 to propose mutations for the sites in $\mathcal{L}_{S-P-X}$ according to a Jukes-Cantor model with mutation rate $\tilde{\mu}$ over a time $T_{S-P-X} = (t_X - t_P) + (t_S - t_P)$, with fixed endpoints. By walking over the mutations along the path S–P–X (which are typically few in number), we identify all sites whose starting and ending states differ, as well as what those states are. For the remaining sites, if any mutations are proposed, we can reconstruct the state at X by looking directly at the reference sequence and the mutations above P. Since a Jukes-Cantor model is time-reversible and has all root prior frequencies of $1/4$, no complications arise if the proposed root node states differ from those at X. Mutations with times in the range $[0, t_S - t_P)$ are mapped with reversed

identities to the range t_S to t_P on the P–S branch, while those with times in the range $[t_S - t_P, T_{S-P-X})$ are mapped with their proposed identities to the range t_P to t_X on the P–X branch. The part of the proposal probability due to the S–P–X path is given by

$$\alpha_{\text{mut, } S\text{–}P\text{–}X}(\mathsf{o} \to \mathsf{n}) = \frac{e^{-\tilde{\mu}|\mathcal{L}_{S-P-X}|T_{S-P-X}} (\tilde{\mu}/3)^{\mathcal{M}_{S-P-X}(\mathsf{n})}}{[P^{JC}_{\neq}(T_{S-P-X})]^{M_{S-P-X}(\mathsf{n})} [P^{JC}_{=}(T_{S-P-X})]^{|\mathcal{L}_{S-P-X}|-M_{S-P-X}(\mathsf{n})}},$$
$$\text{(approximate stochastic mapping on hot sites of the } S\text{–}P\text{–}X \text{ path).}$$

As above, $M_{S-P-X}(\mathsf{n})$ is the actual number of mutations proposed, while $\mathcal{M}_{S-P-X}(\mathsf{n})$ is the number of sites in $\mathcal{L}_{S-P-X}$ where the states at X and S differ. The ratio of genetic priors before and after detaching X is as in the previous section, with a slight change to reflect that the root sequence after detachment is that at S:

$$\mathcal{G}'_{S-P-X} = e^{-\int_{x \in S-P-X} \lambda_{S-P-X}(x)\, dx} \prod_{(a,\ell,b) \in \mathcal{M}_{S-P-X}} Q^{(\ell)}_{ab} \prod_{(a,\ell,b) \in \mathcal{M}_{S-P-X} \text{ on } P\text{–}S \text{ branch}} (\pi^{(\ell)}_a / \pi^{(\ell)}_b).$$

In analogy to the open-ended paths of the previous section, the mutational history proposal for the P–X path is an open-ended proposal for sites in $\mathcal{L}_{P-X}$ ending in the current state at X. As above, this leads to a proposal probability of

$$\alpha_{\text{mut, } P\text{–}X}(\mathsf{o} \to \mathsf{n}) = e^{-\tilde{\mu}|\mathcal{L}_{P-X}|T_{P-X}} (\tilde{\mu}/3)^{\mathcal{M}_{P-X}(\mathsf{n})},$$
$$\text{(approximate stochastic mapping on hot sites of the open-ended } P\text{–}X \text{ path).}$$

The corresponding genetic ratio reflects that the root node state after detachment for these sites is set to the state at X:

$$\mathcal{G}'_{P-X} = e^{-\int_{x \in P-X} \lambda_{P-X}(x)\, dx} \prod_{(a,\ell,b) \in \mathcal{M}_{P-X}} Q^{(\ell)}_{ab} \cdot (\pi^{(\ell)}_a / \pi^{(\ell)}_b).$$

The treatment of the P–S path is exactly analogous.

The final proposal probability for the mutations is given by

$$\alpha_{\text{mut}}(\mathsf{o} \to \mathsf{n}) = \alpha_{\text{mut, } S\text{–}P\text{–}X}(\mathsf{o} \to \mathsf{n}) \cdot \alpha_{\text{mut, } P\text{–}X}(\mathsf{o} \to \mathsf{n}) \cdot \alpha_{\text{mut, } P\text{–}S}(\mathsf{o} \to \mathsf{n}).$$

Similarly, the final ratio of genetic priors before and after grafting is given by

$$\mathcal{G}'(\mathsf{n}) = \mathcal{G}'_{S-P-X}(\mathsf{n}) \cdot \mathcal{G}'_{P-X}(\mathsf{n}) \cdot \mathcal{G}'_{P-S}(\mathsf{n}).$$

6.6 Concrete SPR moves

In the current version of Delphy, we have implemented two of BEAST's SPR moves, subtree slide and Wilson-Balding, as well as a more sophisticated one we call "mutation-directed SPR" (mdSPR). For completeness, we briefly describe the first two to present explicit expressions for $\alpha_{\text{graft}}(\mathsf{o} \to \mathsf{n})$. We then describe mdSPR in detail.

For subtree slide, we pick X as a random non-root node. We then find a point P' at a time δ downstream or upstream of X's parent P, where $\delta \sim \mathcal{N}(0, \Delta^2)$ and $\Delta = \lambda_X^{-1}/2$ (this choice makes it possible for the path from P to P' to sometimes have one mutation, but rarely more). When δ is negative, the choice of P' is unambiguous. Conversely, in the reverse move and when δ is positive, there are K points on the tree to choose from that are descended from P, active at time $t_P + \delta$ and exist before t_X. If $K = 0$, we reject the move. Otherwise, we choose one of these uniformly at random. With the above considerations, the ratio of proposal probabilities is:

$$\frac{\alpha_{\text{graft}}(\mathsf{n} \to \mathsf{o})}{\alpha_{\text{graft}}(\mathsf{o} \to \mathsf{n})} = \begin{cases} K, & \delta > 0; \\ 1/K, & \delta \leq 0, \end{cases} \qquad \text{[Subtree slide].}$$

For the Wilson-Balding move, we pick X randomly such that neither X nor P is the root node. We then pick what will be X's sibling after the move, S', randomly, subject to the following condition. Let $G = \varphi(P)$ and $G' = \varphi(S')$. First, the path G'–S' must have no overlap with the path G–S (including the endpoints). Second, we need $t_{G'} > t_X$, as otherwise we cannot attach X to the branch S' with a positive-length branch. If either of these conditions is not met, we reject the move. Otherwise, we pick the new attachment time $t_{P'} \sim U(t_{G'}, \min[t_X, t_{S'}])$. With the above considerations, the ratio of proposal probabilities is:

$$\frac{\alpha_{\text{graft}}(\mathsf{n} \to \mathsf{o})}{\alpha_{\text{graft}}(\mathsf{o} \to \mathsf{n})} = \frac{\min[t_X, t_{S'}] - t_{G'}}{\min[t_X, t_S] - t_G}, \qquad \text{[Wilson-Balding]}.$$

6.6.1 Mutation-directed SPR moves (mdSPR)

In Figure 17, the possible grafting points for the SPR move that prunes the subtree rooted at X are classified into regions along each branch, demarcated from each other by an intervening mutations; all points in a single region are in the same branch and have the same sequence. It is easy to efficiently calculate the number of sequence differences between the sequence at X and every point in each region, by a simple local walk of the tree: these numbers are written in red inside parentheses. These annotations clearly suggest an effective way to propose a new grafting point for X: overwhelmingly propose points where those differences are minimized. Indeed, the Wilson and Balding's original move implemented a crude rendering of this idea in the context of microsatellites [39]. Zhang, Huelsenbeck and Ronquist's pSPR moves [43], implemented in MrBayes, similarly make use of a parallel parsimony tree to guide move proposals. And matOptimize, which pursues strict parsimony improvements, would *always* pick one of the points with minimal sequence difference, with ties broken arbitrarily.

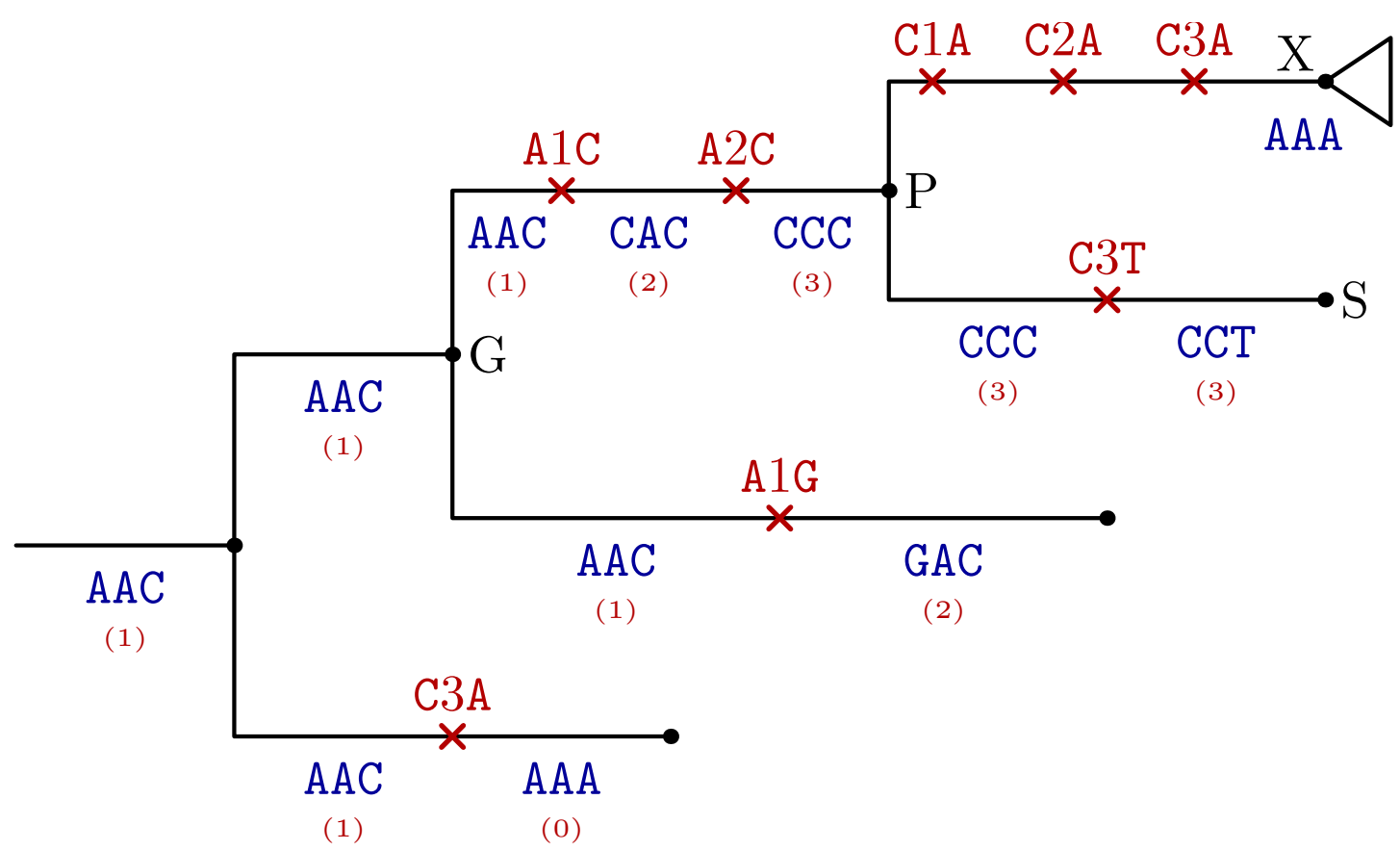

Supplementary Figure 17: Setup for an SPR move. A subtree rooted at X will be detached from the remaining tree, where it is currently grafted at P. The sequence at X is `AAA`, while the sequence at P is `CCC`. Each branch is divided by its mutations into distinct regions, here annotated with their sequence (blue) and the number of differences between that sequence and the sequence at X (red, in parentheses).

Our concrete proposal below, which we call "mutation-directed SPR moves" (mdSPR), exploits the EMAT representation that Delphy already uses as the guide to making SPR proposals. The guide is the "best" one in the sense that a pure mdSPR move reduces to Gibbs sampling when the evolution model is Jukes-Cantor, the mutation rate is low enough that all branches are nearly parsimonious, there is no site-rate heterogeneity, and there is no missing data. In the more general scenario, mdSPR generalizes in a disciplined way the intuition that small changes to the tree are likely to result in a plausible new tree, but that sometimes large-scale changes are essential to correctly sample all plausible trees, not just a subset of closely related ones. While a long enough sequence of local SPR moves are in principle sufficient to turn

any tree topology into any other, mdSPR moves ensure that large-scale rearrangement are possible without going through extremely improbable intermediate states. As we describe below, mdSPR moves provide a clear rationale for deciding what constitutes a local move, what balance to strike between local and global moves, how to choose global rearrangements that are likely to result in posterior increases.

To proceed, we restate the observation in Eq. (9) that the posterior of Eq. (3) simplifies drastically when specialized to a Jukes-Cantor substitution model with no site rate heterogeneity and there is no missing data:

$$\mathcal{G}_{JC}(\mathcal{T}) \propto e^{-\mu LT} \cdot \left(\frac{\mu}{3}\right)^M. \tag{43}$$

As before, T is the total branch length and M is the total number of mutations. The first factor of Eq. (9), equal to $(1/4)^L$, has been subsumed into the constant of proportionality.

Denote by $N(x)$ the number of differences between the sequences at X and an arbitrary point x on the tree. The above picture suggests that, to a good approximation, the posterior ratio $\mathcal{P}(\mathsf{n})/\mathcal{P}(\mathsf{o})$, is given by

$$\frac{\mathcal{P}(\mathsf{n})}{\mathcal{P}(\mathsf{o})} \approx \frac{\exp[-\mu L(t_X - t_{P'})] \cdot [\mu/3]^{N(P')}}{\exp[-\mu L(t_X - t_P)] \cdot [\mu/3]^{N(P)}}.$$

Unlike above, here, $L = |\xi(X)|$ is the number of sites with informative tips downstream of X and μ is an effective site mutation rate, given by $\mu = \lambda(X)/L$. The approximation would be exact only if the substitution model were indeed Jukes-Cantor, there were indeed no missing data, and there were exact cancellation in the ancestry priors. Nevertheless, intuitively, the approximation captures the dominant factors in the posterior ratio. We can thus use it to make a proposal, and thus obtain an MCMC move with near-unity acceptance probability; the deviations from unity exactly correct for the approximate nature of the proposal. As with the proposal for the mutational history along a branch, this proposal using a Jukes-Cantor model and near-parsimonious intuition to make a good proposal, while relying on the Metropolis-Hastings criterion to obtain correct results in the general case.

Concretely, we adopt the following form for the grafting point proposal probability:

$$\alpha_{\text{graft}}(\mathsf{o} \to \mathsf{n}) \propto \exp[-\mu L(t_X - t_{P'})] \cdot [\mu/3]^{N(P')} \cdot (t_X - t_{P'})^{N(P')}. \tag{44}$$

The additional factors of $(t_X - t_{P'})^{N(P')}$ approximately cancel the corresponding factors in $\alpha_{\text{mut}}(\mathsf{o} \to \mathsf{n})$ that account for choosing concrete times for all the relevant mutations (cf., Eq. (42)). The above equation provides an unnormalized probability density for every possible regrafting point P' in terms of easily calculable quantities (see Figure 18). In particular, the quantity $N(P')$ can be computed trivially in an EMAT through a depth-first traversal of the tree starting at P (see annotations in Figure 17). It is this property of EMATs that greatly facilitates the calculation of $N(x)$ everywhere in the tree that motivates our naming this technique *mutation-directed* SPR moves: in effect, the explicit mutations on the tree serve as an excellent and efficient guide to choosing regrafting points well, even over large distances.

To be more concrete, we divide the tree left over after pruning X into K regions as follows. For branch i, let $t_1, \ldots, t_{|\mathcal{M}_i|}$ be the times of the mutations on it; let t_0 be the time of the start of the branch (or $-\infty$ if i is the root node) and let $t_{|\mathcal{M}_i|+1}$ be the time of the end of the branch. Then record the region of branch i spanning the time (t_j, t_{j+1}) for each j between 0 and $|\mathcal{M}_i|$ (inclusive). Finally, remove all regions that are entirely to the future of t_X; those regions that span t_X are also truncated at t_X. All points x in each region have the same value of $N(x)$, which we also record while constructing the regions. In the end, a region k thus consists of a tuple (i_k, t_k, t'_k, N_k), respectively the branch index, the region's start and end times, and the number of sequence differences between any point in the region and X. We call such a set of annotated regions an "SPR study". With this information, we define a weight w_k for each region as follows:

$$w_k = \int_{t_k}^{t'_k} dt\, \exp[-\mu L(t_X - t)][\mu(t_X - t)/3]^{N_k}. \tag{45}$$

To pick a new regrafting point, pick a region k in proportion to the weights $\{w_k\}$. Then pick a point between t_k and $t_{k'}$ in proportion to

$$p(t|k) \propto \exp[-\mu L(t_X - t)][\mu(t_x - t)/3]^{N_k}. \tag{46}$$

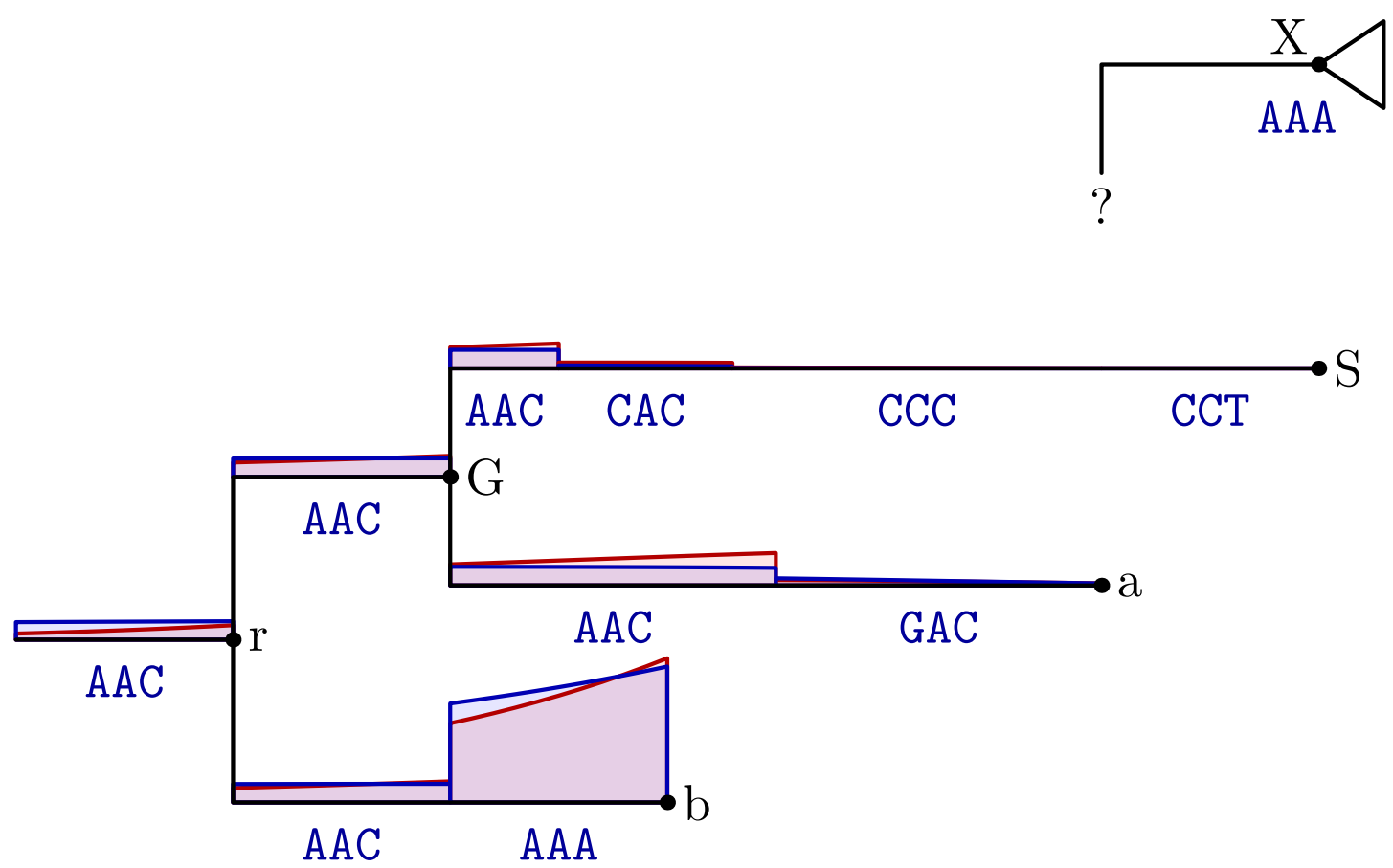

Supplementary Figure 18: Cartoon of grafting site probabilities calculated by mutation-directed SPR move (red) vs. distribution implied by the posterior (blue). In this example, the real posterior has an HKY substitution model with $\kappa = 5.25$, $\pi = [0.29, 0.18, 0.19, 0.33]$, which is representative of the SARS-CoV-2 data in [18]. Note that probabilities are broadly similar. The parsimonious region with sequence AAA, where attaching X necessitates no mutations on the P'–X branch, is clearly the most likely choice, but all other regions are possible, and their combined weight need not be negligible.

All of the above calculations can be implemented in terms of incomplete Gamma functions and their inverses, which are widely available (e.g., in `boost::math` for C++ and in `scipy.special` in Python).

The above procedure describes the "pure" version of mdSPR. In practice, we apply the following changes that make the move more efficient to execute:

- When walking the tree to enumerate regions and calculate $N(x)$, we skip over mutations on sites $\ell \notin \xi(X)$, since they would not require additional mutations along the P'–X branch owing to `N`-pruning.
- Particularly after the burn-in period of the MCMC, it is wasteful to consider possible new grafting points over the entire tree, since X is presumably grafted near a locally optimal spot. Thus, most of the time, we limit the walk on the tree to regions for which $N_k \le 1$; when the walk would cross a mutation that would make $N_k = 2$, we truncate the walk and begin to backtrack. Nevertheless, about 1 % of the time, we do perform complete explorations of the tree, to account for nodes X which could be comfortably grafted in multiple, separated areas of the tree, and to always allow the MCMC some chance of escaping local minima.
- We observed that sometimes mdSPR would make good proposals too eagerly compared to what was implied by the actual evolution model, and which would then be rejected according to the Metropolis-Hastings criterion. For example, proposals near the beginning of a run where the P–X branch had dozens of mutations and the P'–X branch had 0 or 1 would be spuriously rejected! To mitigate this issue, we actually use a dampened p.d.f. for the grafting point:

$$\alpha_{\text{graft}}(\mathtt{o} \to \mathtt{n}) \propto \Big[\exp[-\mu L(t_X - t_{P'})] \cdot [\mu/3]^{N(P')} \cdot (t_X - t_{P'})^{N(P')}\Big]^f .$$

 The annealing factor f, so-called because it plays an analogous role to a temperature factor in simulated annealing, leads to mdSPR making the most promising grafting proposals less eagerly than implied by a Jukes-Cantor model, which then leaves some room for the Metropolis-Hastings factor to make corrections without driving the acceptance probability to 0 (at the cost of occasionally making and rejecting grafting proposals that are less promising). Empirically, we found that $f = 0.8$ is sufficient to keep mdSPR effective while accepting most of the beneficial proposals in the burn-in phase. This value

is hard-coded and not tunable by end users: it nonetheless appears to work well over a wide range of datasets.

- Finally, since incomplete Gamma functions and their inverses are expensive to calculate, we actually approximate the integrand in the definition of w_K by its value at the midpoint time $t_* = (t'_k + t_k)/2$, so that

$$w_k \approx (t'_k - t_k) \left[\exp[-\mu L(t_X - t_*)][\mu(t_X - t_*)/3]^{N_k}\right]^f .$$

  Correspondingly, in those regions, we approximate $p(t|k)$ by a uniform distribution between t_k and t'_k. We have not made any attempt to detect regions for which this approximation would be inappropriate, such as long branches or branches that span the maximum of the integrand. However, the approximation cannot be applied for the region above the root node (where $t_k = -\infty$), so there, we do use the full expression and pay the cost of using incomplete Gamma functions.

As noted in the main text, we have empirically observed that MCMC runs for genomic epidemiology datasets (real and simulated), which previously ran into convergence problems and/or for which we needed to hand-tune step sizes in the subtree slide move, all converge reliably when mdSPR moves are used, even though mdSPR exposes no tunable parameters. We thus believe that mdSPR plays a foundational role in making our MCMC robust without requiring any parameter tuning.

6.7 Other local moves

Besides SPR moves, we have implemented two other cheap move types to rapidly and efficiently sample a tree's mutational history and branch lengths while keeping its topology fixed:

1. *Inner node displacement moves*: We first pick a non-tip node X and note its allowable range of times $(t_{\min}, t_{\max})$, subject to preserving the tree topology and the times of all mutations on the branches impinging on it. If X is not the root node, we pick a new time directly from the exponential distribution $\exp[+\lambda_X t]$ with $t_{\min} < t < t_{\max}$. This choice of t_X exactly cancels the ratio of genetic priors in the acceptance probability, so only the ratio of coalescent priors must be calculated, which is usually close to 1. If, however, X is the root node, we instead add to the old time a Gaussian-distributed displacement with mean 0 and standard deviation $\lambda_X^{-1}/2$ (as in the subtree slide move). For non-root nodes, this move efficiently approximates Gibbs sampling of branch lengths conditioned on the topology and mutational history not changing. In this sense, it is reminiscent of the Thorney Beast extension to BEAST, which has been used [31] for Bayesian inference of very large trees with à prior fixed topologies (up to polytomies).

2. *Branch reform moves*: We first pick a random branch i other than the root branch. If i is also not a child of the root node, we pick new times for the mutations on branch i uniformly between $t_{\varphi(i)}$ and t_i (if there are multiple mutations on the same site, we sort the proposed times and preserve the relative order of the existing mutations). Because mutations only change $\lambda(x)$ very slightly over the length of the branch, this proposal is close to a Gibbs sampling of the mutation times, and so is almost always accepted. When i is a child of the root node, however, we instead execute an SPR move with $P' = P$, as described above; this allows mutations on one child branch of the root node to easily migrate to the other branch, with a concomitant root sequence change.

Throughout the development of Delphy, we experimented with many other possibilities for local MCMC moves. These included: (a) "junction reform moves", which would resample the mutational history of all three branches impinging on a node simultaneously while keeping the rest of tree fixed; (b) narrow and wide exchange moves, analogous to those implemented in BEAST; and (c) "cluster" moves, wherein an SPR move proposes changing the mutational history of a connected cluster of branches, of which the P'–X is but one, and amounts to a local ancestral state reconstruction. All of these were technically more complex to implement than the battery of moves described in this local MCMC moves section (particularly cluster moves), and yet we did not notice an appreciable effect on MCMC convergence from disabling them. In the

interests of simplicity, robustness and ease of evolving Delphy in the future, we dropped all support for these other kinds of moves. In agreement with the experience of matOptimize, it seems that simple SPR moves, particularly when mutation-directed, suffice to explore tree space for the densely sampled datasets we are targeting.

7 Global MCMC moves

We now focus on *global* MCMC moves, where we keep the topology and genetic history of the tree fixed but vary the associated parameters $\boldsymbol{\theta}$. As noted by Lartillot as early as 2006 [17], an explicit-mutation representation lends itself to surprisingly cheap global moves, often perfect Gibbs samplers. In contrast, when using an implicit representation, global moves involve a full recalculation of the tree likelihood, and so are among the most expensive moves in the MCMC. Here, we summarize the global moves used by Delphy, which essentially recap Lartillot's earlier proposals.

7.1 Mutation rate μ

The conditional posterior distribution for μ follows from Equation (3) up to normalization:

$$P_\mu(\mu) \propto e^{-\mu L \tilde{T}} \cdot \mu^M \cdot \pi_\mu(\mu). \tag{47}$$

Here, M is the total number of mutations and $\tilde{T}$ is an effective total branch length of the tree, defined as

$$\tilde{T} = \frac{\int_{x \in \mathcal{T}} \lambda(x)\, dx}{\mu L}.$$

The right-hand side is independent of μ, as follows from expanding the definitions of $\lambda(x)$ and $Q^{(\ell)}_{ab}$. We note that maintaining an updating the values of M and $\tilde{T}$ is straightforward, and both can be calculated efficiently from scratch in time linear with the size of the tree.

An MCMC move that proposes a change from μ to μ' with probability $\alpha(\mu \to \mu')$ has acceptance probability

$$P_{\text{acc}}(\mu \to \mu') = \min\left[1, e^{-(\mu'-\mu)L\tilde{T}} \cdot \left(\frac{\mu'}{\mu}\right)^M \cdot \frac{\pi_\mu(\mu')}{\pi_\mu(\mu)} \cdot \frac{\alpha(\mu' \to \mu)}{\alpha(\mu \to \mu')}\right].$$

For a general prior $\pi_\mu(\mu)$, this acceptance probability can be applied to moves that perturb μ, e.g., scaling moves. Because such a move is very cheap, it's beneficial to attempt many such moves in quick succession without changing the tree topology, in effect approximating a Gibbs sampler for μ.

In Delphy, a desire for conjugacy has motivated our choice for $\pi_\mu(\mu)$ to be a Gamma(α_μ, β_μ) distribution (which includes the standard choices of an improper uniform prior and a $1/x$ prior if we allow $\alpha_\mu = 0$ and/or $\beta_\mu = 0$). This choice results in a conditional posterior in Equation (47) is a Gamma distribution, so we can go further and directly and cheaply Gibbs sample μ:

$$\mu \sim \text{Gamma}(M + \alpha_\mu, L\tilde{T} + \beta_\mu).$$

For the concrete choices of a uniform prior and a $1/x$ prior, we have

$$\mu \sim \begin{cases} \text{Gamma}(M+1, L\tilde{T}), & \text{for } \pi_\mu(\mu) = 1, \text{ i.e., } \alpha_\mu = 1 \text{ and } \beta_\mu = 0; \\ \text{Gamma}(M, L\tilde{T}), & \text{for } \pi_\mu(\mu) = 1/\mu, \text{ i.e., } \alpha_\mu = 0 \text{ and } \beta_\mu = 0. \end{cases}$$

The above procedure also makes it clear that the conditional average of μ is close to $M/(L\tilde{T})$ (exactly so for a $1/x$ prior), a physically pleasing result.

In our initial implementation of Delphy, we have not implemented a move analogous to BEAST's up-down operator that can simultaneously scale the mutation rate and all the branch lengths while keeping their

product roughly unchanged. While doing so would speed convergence for trees where tips are concentrated in a small time window at the end of the tree [8], we are targeting use cases where tips occur throughout a large fraction of the range of the tree, where such a simultaneous scaling move would have low acceptance probability.

7.2 Evolution model parameters $\{\pi_a\}$, $\{q_a\}$ and $\{q_{ab}\}$

As for the mutation rate μ, we can construct a joint conditional posterior distribution for the variables that specify the evolution model parameters, $\{\pi_a\}$, $\{q_a\}$ and $\{q_{ab}\}$. For the HKY model we use, while $\{\pi_a\}$ is specified directly, the other parameters are derived from the variable κ; the procedure below, however, is easily adapted to any other model of evolution. Thus, the joint conditional posterior distribution for κ and $\{\pi_a\}$ that follows from Equation (3) is, up to normalization:

$$P_Q(\kappa, \{\pi_a\}) \propto \prod_a \pi_a^{R_a} \cdot e^{-\mu L \sum_a q_a \tilde{T}_a} \cdot \prod_{a,b} (q_{ab})^{M_{ab}} \cdot \pi_Q(\kappa, \{\pi_a\}). \tag{48}$$

Here, R_a is the number of sites with state a in the root sequence, M_{ab} is the number of a-to-b mutations in the tree, and $\tilde{T}_a$ is an effective total branch length in state a, defined as

$$\tilde{T}_a = \frac{1}{q_a \mu L} \int_{x \in \mathcal{T}} \sum_{\ell \in \xi(x)} [s^{(\ell)}(x) = a] Q_a^{(\ell)}.$$

As above, $\tilde{T}_a$ is actually independent of the values of μ and q_a. The above expression is valid when the same substitution model applies at all sites, modulated only by the site rates $\nu^{(\ell)}$, but is easy to generalize to multiple partitions. As noted before, these values can be calculated efficiently from scratch and maintained up to date after local MCMC moves.

As with the mutation rate, the above conditional posterior suggests very cheap moves for making small trial changes to κ and $\{\pi_a\}$, for example, by scaling and delta-exchange, respectively. A long sequence of such moves approximates a Gibbs sampler for κ and $\{\pi_a\}$, and can be implemented efficiently if there are no intervening topological or mutational changes to the tree. Concretely, we make and potentially accept the following proposal of moves 10 times in a row:

- Delta-exchange on $\{\pi_a\}$: pick a random pair of distinct states a and b and a displacement $\sim \mathrm{Unif}(0, 0.01)$; then propose $\pi_a^{(\mathrm{new})} = \pi_a^{(\mathrm{old})} + \delta$ and $\pi_b^{(\mathrm{new})} = \pi_b^{(\mathrm{old})} - \delta$. Rejecti immediately if either proposed value is outside $[0, 1]$.
- Scale moves on κ: multiply κ by a random factor $s \sim \mathrm{Unif}(3/4, 4/3)$, with appropriate Hastings ratio $1/s$.

Unlike with the mutation rate, the above conditional posterior does not immediately suggest a direct Gibbs sampler for any common choice of prior $\pi_Q(\kappa, \{\pi_a\})$. However, Nicola de Maio has pointed out to us that an UNREST evolution model [40] may be suitable for densely sampled datasets. In this model, each transition rate is inferred independently under a Gamma-distributed prior, and so the conditional posterior for all rates is a product of independent Gamma distributions; similarly, the parameters $\pi_a^{(\ell)}$ stop playing the role of the stationary distribution of the evolution model, and instead become free parameters with Dirichlet priors, so that their conditional posterior is a Dirichlet-multinomial distribution. These changes imply efficient Gibbs samplers for all the rates $Q_{ab}^{(\ell)}$ and $\pi_a^{(\ell)}$ [17]. We leave the exploration of this possibility for future work.

7.3 Population model $N(t)$

When using an exponentially-growing population model, the conditional posterior distribution for the parameters n_0 and g that specify the population curve $N(t) = n_0 e^{gt}$ depends only on the coalescent prior and

parameter priors, as follows:

$$P_N(n_0, g) \propto \pi_{\text{anc}}(\mathcal{T}|\boldsymbol{\theta})\pi_N(n_0, g).$$

The coalescent prior depends on the tree topology only through (a) the function $k(t)$ that counts the number of active branches at time t; and (b) the times $\{t_i\}$ of all the inner nodes. By using a fine-grained piecewise-constant approximation to $k(t)$, as described in Section 9, we can efficiently evaluate ratios of the above conditional posterior for old and new values of n_0 and g. Hence, we can execute a long sequence of consecutive MCMC moves that perturb n_0 and g so as to approximate a Gibbs sampler for both. Concretely, we run a batch of 100 moves interleaving scaling moves on n_0 with a factor $\sim \text{Unif}(3/4, 4/3)$ and random walks on g with steps $\Delta g \sim \text{Unif}(-1/\text{year}, +1/\text{year}$.

When using a Skygrid model, on the other hand, proposing new values of $\{\gamma_k\}$ with high acceptance probabilities is difficult. Following Ref. [1], we do not implement the original block-update move (i.e., Laplace approximation) described in Ref. [11], but a Hamiltonian Monte Carlo move. Unlike in [1], we only include the variables $\{\gamma_k\}$ and not τ, which we usually fix instead (see discussion in Section 3.4). As in Ref. [1], we regard the $\{\gamma_k\}$ variables as positions in a dynamical system, with associated masses $\{m_k\}$ and momenta $\{p_k\}$. As is standard, these are then evolved according to a Hamiltonian $\mathcal{H}(\{\gamma_k\}, \{p_k\})$ given by

$$\begin{aligned}
\mathcal{H}(\{\gamma_k\}, \{p_k\}) &:= U(\{\gamma_k\}) + K(\{p_k\}), \\
U(\{\gamma_k\}) &:= \log[\pi_{\text{anc}}(\mathcal{T}|\boldsymbol{\theta})\pi_\gamma(\{\gamma_k\})], \\
K(\{p_k\}) &:= \sum_{k=0}^{M} \frac{p_k^2}{2m_k}.
\end{aligned}$$

If evolved without integration error, the reversible trajectory traces out values of $\{\gamma_k\}$ and $\{p_k\}$ that are as probable as the original values under a posterior that has been extended by a factor of $\exp[-K(\{p_k\})]$, and so an MCMC proposal would have acceptance ratio of 1 while evolving all the $\{\gamma_k\}$ in non-trivial ways. Any concrete numerical integrator that only approximately conserves $\mathcal{H}$ has an acceptance ratio related to the (small) numerical error incurred. As usual, the actual values of the masses $\{m_k\}$ do not affect the correctness of the proposal, but may affect its effectiveness.

We deviate from Ref. [1] in two ways. First, we use a position Verlet instead of a velocity Verlet integrator [35] to evolve the dynamical system, since that requires one fewer force evaluation and is about as accurate:

$$\gamma_k(t + dt/2) = \gamma_k(t) + (dt/2)(p_k(t)/m_k), \tag{49}$$

$$p_k(t + dt) = p_k(t) + dt\, f_k(t + dt/2), \tag{50}$$

$$\gamma_k(t + dt) = \gamma_k(t + dt/2) + (dt/2)(p_k(t + dt)/m_k). \tag{51}$$

As usual, $f_k(\{\gamma_k\}) = -\partial U/\partial\gamma_k$, and we employ the obvious shorthand $f_k(t) = f_k(\{\gamma_k(t)\})$. While the calculation of f_k is tedious, it is in principle a straightforward application of the chain rule through the various definitions; in particular, we note that $\pi_{\text{anc}}(\mathcal{T}|\boldsymbol{\theta})$ is discretized into several hundred cells with constant population (see Eq. (57) below), which makes the force calculation tractable even for the "log-linear" Skygrid.

Second, we use a heuristic to choose different masses for each knot k. Considering momentarily only on the GMRF and coalescent priors, by Taylor expanding U around its minimum and assuming that $\gamma_{k-1} \approx \gamma_k \approx \gamma_{k+1}$, we find that

$$\frac{\partial^2 U}{\partial \gamma_k^2} \approx -(\underbrace{\tau[k > 0] + \tau[k < M] + c_k}_{m_k}),$$

where, as in Ref. [11],

$$c_k = \sum_{i \text{ inner}} [\gamma_{k-1} < t_i \leq \gamma_k].$$

Here, $[X]$ is the Iverson bracket, which is 1 when condition X is true and 0 otherwise. The quantity c_k counts the number of coalescences occurring in the interval k between x_{k-1} and x_k (with suitably adjustments at

$k = 0$ and $k = M$). The above result suggests that near equilibrium, the values of γ_k will oscillate roughly harmonically with angular frequency 1 for all k if we set m_k to the quantity in parenthesis above. By choosing the masses in this way, all natural frequencies of the dynamical system will be roughly equal, so we avoid being forced to use a very small timestep to accomodate the fastest natural mode of the system. We emphasize that the above result is a rough heuristic only, but we have found it effective in our HMC implementation. We also note that we also attempted to use normal mode amplitudes as coordinates for HMC, for which we can find masses that make all normal modes oscillate at *exactly* the same frequency when $U = -\log \pi_\gamma(\{\gamma_k\})$. In practice, however, that approach performs poorly, suggesting that fluctuations in $\{\gamma_k\}$ are dominated by the constraints of the coalescent except in intervals where there are few or no active lineages.

Finally, to implement HMC proposals, we must choose a timestep and a trajectory length. We use the Randomized HMC heuristic [5] of choosing the trajectory length from an exponential distribution, with mean of $2\pi/4$, i.e., a quarter of the oscillation period of a harmonic oscillator with angular frequency 1, assuming the above heuristic for $\{m_k\}$ is effective in synchronising most motions to that angular frequency. Then we divide this length into 25 equal-time steps, high enough to avoid serious integration errors, but a choice not otherwise optimized. Randomized HMC adds robustness by avoiding trajectories that always perform complete cycles in $\{\gamma_k\}$ and by probabilistically allowing for very short timesteps around values of $\{\gamma_k\}$ for which integration error would otherwise be significant and always result in rejection. With the above choices, we have observed our HMC sampler to be strongly robust without introducing any need for a user to fine-tune any parameters.

On top of HMC moves, we also implement a simple but effective Gibbs move on the overall scale of the population curve. Observe that we can decompose the various $\{\gamma_k\}$ into a mean $\bar{\gamma}$ and deviations $\{\gamma'_k\}$ from this mean:

$$\bar{\gamma} := \frac{1}{M+1} \sum_{k=0}^{M} \gamma_k, \tag{52}$$

$$\gamma'_k := \gamma_k - \bar{\gamma}. \tag{53}$$

By construction, $\pi_\gamma(\{\gamma_k\})$ is independent of $\bar{\gamma}$. Moreover, if we fix all $\{\gamma'_k\}$, then the dependence of π_{anc} on $\bar{\gamma}$ is particularly simple. Concretely, we begin by changing coordinates from $\{\gamma_k\}$ to $\{\bar{I}, \gamma'_k | k < M\}$ (we've excluded γ'_M since it's not independent of the other M values $\{\gamma'_k | k < M\}$). Here, $\bar{I}$ acts as a "mean intensity", defined by:

$$\bar{I} := \exp[-\bar{\gamma}]. \tag{54}$$

Factoring $\bar{I}$ from $N(t)$ yields a reduced population curve $\tilde{N}(t)$ defined as follows:

$$\tilde{N}(t) = N(t) \cdot \bar{I}.$$

Expressing the coalescent prior (Eq. (56) below) in terms of these new coordinates yields

$$\pi_{\text{anc}}(\{\gamma_k\}) = \exp\left[-\int \binom{k(t)}{2} \frac{dt}{N(t)}\right] \cdot \prod_{\text{inner nodes } j} \frac{1}{N(t_j)},$$

$$\implies \pi_{\text{anc}}(\{\bar{I}, \gamma'_k | k < M\}) = \exp[-B\bar{I}] \cdot \bar{I}^{N_{\text{inner}}} \cdot (\text{const.}),$$

where the quantity B is given by

$$B := \int \binom{k(t)}{2} \frac{dt}{\tilde{N}(t)}.$$

Note that π_{anc} is not a probability density for $\{\gamma_k\}$, but a probability density over tree topologies conditioned on a choice of $\{\gamma_k\}$, i.e., a particular population curve. Hence, there is no Jacobian factor introduced here in changing to $\{\bar{I}, \gamma'_k | k < M\}$ coordinates. Recall that we additionally impose an Inv-Gamma$(\alpha_{\bar{N}}, \beta_{\bar{N}})$ prior on $\bar{N}$, or equivalently, a Gamma$(\alpha_{\bar{N}}, \beta_{\bar{N}})$ prior on $\bar{I}$:

$$\pi_{\bar{N}}(\bar{I}) \propto \bar{I}^{\alpha_{\bar{N}}-1} e^{-\beta_{\bar{N}} \bar{I}}, \qquad (\text{in } \bar{I} \text{ coordinates}).$$

It follows that the posterior conditioned on constant $\{\gamma'_k\}$ is

$$P(\bar{I}) = \bar{I}^{N_{\text{inner}}+\alpha_{\bar{N}}-1} e^{-(B+\beta_{\bar{N}})\bar{I}} \cdot (\text{const.}),$$
$$\implies \bar{I} \sim \text{Gamma}(N_{\text{inner}} + \alpha_{\bar{N}}, B + \beta_{\bar{N}}).$$

That immediately provides a Gibbs sampler for $\bar{I}$ (and motivates our choice of an Inverse-Gamma prior for $\bar{N}$). The interpretation of this result is physically appealing: the reduced population curve $\tilde{N}(t)$ acts to dilate or compress relative time in the phylogeny, such that in units of $dt/\tilde{N}(t)$, the effective population is effectively constant. The above Gamma distribution is then the standard result for the population size distribution for a model with constant population size n_0 under an Inverse-Gamma prior for n_0.

We note in passing that a similar argument allows for a Gibbs move on n_0 in a model with an exponentially growing population, provided that we use an Inverse-Gamma prior for n_0. While that motivates our choice above for this particular prior for n_0, Delphy does not yet implement such a Gibbs move.

7.4 Site-rate heterogeneity parameters, α and $\{\nu^{(\ell)}\}$

In the standard approach to modeling site-rate heterogeneity, the Gamma distribution for the site relative rates $\{\nu^{(\ell)}\}$ is approximated by a discrete distribution with K categories because it is not possible to analytically integrate the tree likelihood for all choices of $\{\nu^{(\ell)}\}$. Instead, the integration is approximated by K evaluations of the tree likelihood at evenly distributed quantiles of $\nu^{(\ell)}$, with a concomitant K-fold increase in cost to every MCMC move that changes the tree likelihood. In contrast, in our explicit approach, we do not attempt to integrate these degrees of freedom analytically, but instead let the integration occur implicitly via sampling. In other words, both α and $\{\nu^{(\ell)}\}$ are explicit, continuous parameters of the inference, and individual MCMC moves are not appreciably more expensive when including vs excluding site-rate heterogeneity effects. We note that recent versions of MAPLE have also implemented an equivalent idea in the context of maximum likelihood phylogenetics [21].

It is tempting to evaluate conditional posterior distributions for α and $\{\nu^{(\ell)}\}$ separately, and then implement separate moves on each. The relevant joint conditional posterior distribution again follows from Equation (3) up to normalization:

$$P_\nu(\alpha, \{\nu^{(\ell)}\}) \propto \pi_\alpha(\alpha) \cdot \prod_\ell e^{-\mu\nu^{(\ell)}\tilde{T}^{(\ell)}} \cdot \left[\nu^{(\ell)}\right]^{M^{(\ell)}} \cdot \frac{\alpha^\alpha}{\Gamma(\alpha)} \left[\nu^{(\ell)}\right]^{\alpha-1} e^{-\alpha\nu^{(\ell)}}. \tag{55}$$

Hence, the individual conditional posterior distributions are:

$$P_\alpha(\alpha) \propto \pi_\alpha(\alpha) \cdot \prod_\ell \frac{\alpha^\alpha}{\Gamma(\alpha)} \left[\nu^{(\ell)}\right]^{\alpha-1} e^{-\alpha\nu^{(\ell)}},$$
$$P_{\nu^{(\ell)}}(\nu^{(\ell)}) \propto e^{-\mu\nu^{(\ell)}\tilde{T}^{(\ell)}} \cdot \left[\nu^{(\ell)}\right]^{M^{(\ell)}} \cdot \left[\nu^{(\ell)}\right]^{\alpha-1} e^{-\alpha\nu^{(\ell)}}.$$

Here, $M^{(\ell)}$ is the number of mutations in site ℓ and $\tilde{T}^{(\ell)}$ is an effective total branch length for site ℓ, given by

$$\tilde{T}^{(\ell)} := \frac{1}{\mu} \int_{x\in\mathcal{T}} [\ell \in \xi(x)] Q_a^{(\ell)}\, dx.$$

It is impractical to update the vector $\tilde{T}^{(\ell)}$ after every local MCMC move. However, this vector can be efficiently calculated in time proportional to the sum of the tree size and the number of mutations and missations as follows: (a) accumulate the total branch length of every subtree rooted at inner node i, as if there were no missing data, by a post-order traversal of the tree; (b) calculate an initial value of $\tilde{T}^{(\ell)}$ for each site using the reference sequence and the total branch length at the root node, as if there were no mutations or missations on the tree; then (c) walk the tree and adjust $\tilde{T}^{(\ell)}$ for each observed mutation and missation at site ℓ.

Observe that, like the prior $\pi_\nu(\nu^{(\ell)})$, the conditional posterior for $\nu^{(\ell)}$ given above is the distribution $\text{Gamma}(\alpha + M^{(\ell)}, \alpha + \mu\tilde{T}^{(\ell)})$. The tree topology and mutational history of site ℓ simply change the

prior's Gamma parameters in a way that compares the number of mutations on this site expected in the absence of site-rate heterogeneity ($\mu\tilde{T}^{(\ell)}$) with the actual number ($M^{(\ell)}$), and α modulates the extent to which discrepancies are ascribed to real differences in rates vs stochastic fluctuations. Moreover, because the posterior distribution for $\nu^{(\ell)}$ is a Gamma distribution, we can draw new conditional samples of $\{\nu^{(\ell)}\}$ efficiently. For α, though, we are again reduced to making trial changes, e.g., by scaling, and accepting or rejecting them in the usual way.

In practice, the above scheme of separately sampling α and $\{\nu^{(\ell)}\}$ is not effective. The values of α and $\{\nu^{(\ell)}\}$ are very correlated, and independent moves along each of them do not effectively explore the joint parameter space. However, one can marginalize the $\{\nu^{(\ell)}\}$ in Equation (55) to get an effective conditional posterior distribution for α:

$$\tilde{P}_\alpha(\alpha) \propto \pi_\alpha(\alpha) \cdot \prod_\ell \frac{\Gamma(\alpha + M^{(\ell)})}{\Gamma(\alpha)} \frac{\alpha^\alpha}{(\alpha + \mu\tilde{T}^{(\ell)})^{\alpha+M^{(\ell)}}}$$

Although the function $\tilde{P}_\alpha(\alpha)$ is expensive to calculate, it is relatively smooth, so repeated trial changes to α will have high acceptance probabilities and quickly approximate a Gibbs sampler of $\tilde{P}_\alpha(\alpha)$. After thus obtaining a new value of α, we directly sample all L new values of $\nu^{(\ell)}$ as described above.

In concrete terms, we first perform 10 consecutive scaling proposals for α with a scaling factor $\sim \text{Unif}(0.9, 1/0.9)$, appropriate Hastings ratio, and effective posterior $\tilde{P}(\alpha)$. Then, using the value of α at the end of thes 10 proposals, we sample each $\nu^{(\ell)}$ independently from $\text{Gamma}(\alpha + M^{(\ell)}, \alpha + \mu\tilde{T}^{(\ell)})$.

8 Scaling strategy

In matOptimize, the search for a parsimonious tree is parallelized by observing that the effect of an SPR moves is quite localized, so two SPR moves in different portions of the tree can first be evaluated separately, and their combined effect deduced from those separate evaluations. In other words, the net effect of two non-conflicting SPR moves is independent of the order in which those moves are performed. The MCMC SPR moves on EMATs described above have an analogous property: if we partition a tree into non-overlapping subtrees and independently perform SPR moves in each subtree in parallel, the net result is equivalent to performing those moves serially by interleaving the subtree moves in any way. This idea is depicted schematically in Figure 19.

9 Parallelizable coalescent prior

As is common in Bayesian phylogenetics of viruses, our ancestry prior $\pi_{\text{anc}}(\mathcal{T}|\boldsymbol{\theta})$ is the Kingman coalescent [14, 15], given by

$$\pi_{\text{anc}}(\mathcal{T}|\boldsymbol{\theta}) := \exp\left[-\int \binom{k(t)}{2} \frac{dt}{N(t)}\right] \cdot \prod_{\text{inner nodes } j} \frac{1}{N(t_j)}, \tag{56}$$

where $k(t)$ is the number of active branches at time t and $N(t)$ is the product of the effective population size and the generation time.

A significant complication in parallelizing the coalescent prior is that it directly couples all the nodes at the end of branches that cross a given time t. Hence, at first glance, the use of a coalescent prior seems in conflict with the scaling strategy described above. In this section, we describe in detail one scheme that resolves that conflict through the introduction of an augmented coalescent prior. We emphasize that this scheme is not the only possible scheme, nor is it necessarily the best one. Better schemes may yet improve the sampling.

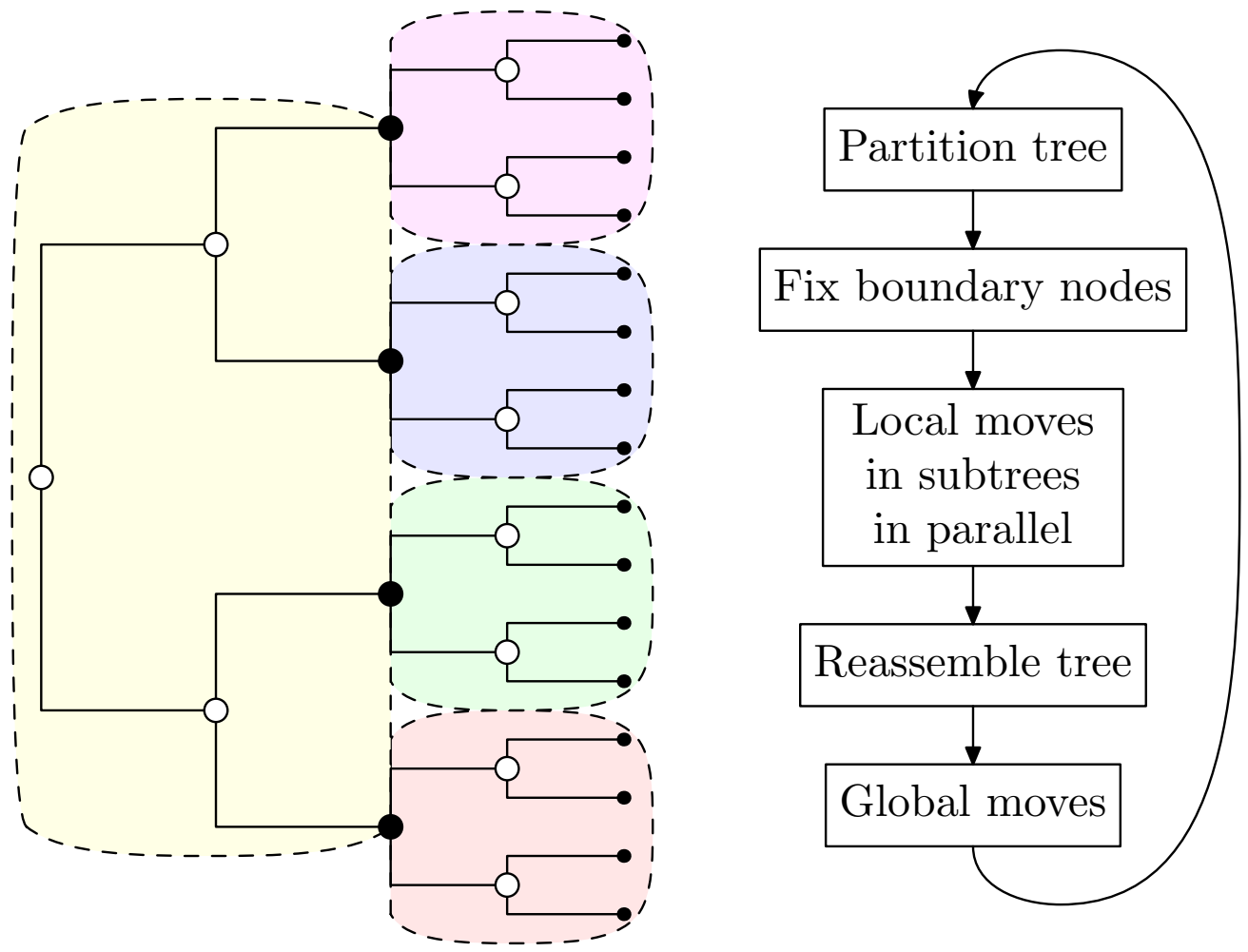

Supplementary Figure 19: Schematic of Delphy's scaling strategy. In each cycle, the sequence and times of the boundary nodes (filled) is fixed, while the sequence, times and topology of the remaining nodes (unfilled) can change. Within each shaded subtree, local MCMC moves are confined to that subtree. To ensure ergodicity, the fixed boundary nodes on successive cycles do not overlap.

First, instead of evaluating Eq (56) piecewise directly, we start by discretizing it into finite time segments "cells" of width Δ over the range of times spanned by the tree:

$$\pi_{\text{anc}}(\mathcal{T}|\boldsymbol{\theta}) \approx \bar{\pi}_{\text{anc}}(\mathcal{T}|\boldsymbol{\theta}) := \exp\left[-\sum_c \frac{\bar{k}_c(\bar{k}_c - 1)}{2}\frac{\Delta}{\bar{N}_c}\right] \cdot \prod_{\text{inner nodes } j} \frac{1}{N(t_j)}. \tag{57}$$

Here and below, a barred variable, say $\bar{k}_c$, stands for the mean value of a corresponding function of time, say $k(t)$, over the time interval spanned by cell c, which need not be an integer. In our current implementation, we partition the range over which $k(t) > 0$ into roughly 400 intervals, periodically changing Δ if the range of $k(t)$ changes drastically. In practice, Δ is readjusted a handful of times during the burn-in period as the tree height converges, but remains stable throughout the production period of the MCMC run. Note that this discretization already results in substantial efficiency improvements, because the cost to displacing an inner node scales with the change in time, not with the density of coalescence events in other parts of the tree around the node's time.

Because the coalescent prior introduces only modest gradients into the posterior, the crudeness of the above approximation does not seem to impact results appreciably. One notable exception is in very large datasets, such as the simulated 100 000-sample outbreak, where the $k(t)^2$ dependence of the integrand and the typically high values of $k(t)$ require a much higher number of cells to mitigate the discretization errors introduced by the above approximation. It remains to be seen whether more sophisticated approximations to the integral in Eq. (56) are also amenable to the parallelization scheme described below.

Second, we split the tree $\mathcal{T}$ into P partitions. It follows that we can decompose the number of active lineages $\bar{k}_c$ into a sum over the numbers of active lineages $\bar{k}_{p,c}$ in partition p, $\bar{k}_c = \sum_p \bar{k}_{p,c}$. Rewriting Eq. (57) in terms of partitions yields

$$\bar{\pi}_{\text{anc}}(\mathcal{T}|\boldsymbol{\theta}) = \prod_p \left\{ \prod_c \exp\left[-\Delta\left(\frac{\bar{k}_{p,c}\cdot[\sum_q \bar{k}_{q,c}]}{2\bar{N}_c} - \frac{\bar{k}_{p,c}}{2\bar{N}_c}\right)\right] \cdot \prod_{j\in p} \frac{1}{N(t_j)} \right\}. \tag{58}$$

This form emphasizes the coupling between partitions p and q owing to the quadratic term in the exponential.

An analogy with electrostatics can be drawn at this point: the potential energy of a set of particles can be calculated either as the sum of pairwise direct Coulomb interactions between all pairs of particles,

or by a sum of local interactions between charges and an induced local potential. In other words, we can replace direct interaction at a distance between charges with a local interaction mediated by a potential that transmits information through space. This picture suggests introducing for each cell c a set of P augmenting Gaussian variables $\tilde{k}_{p,c}$ that couple linearly to the $\{\bar{k}_{p,c}\}$ according to the following *augmented coalescent prior*:

$$\tilde{\pi}_{\text{anc}}(\mathcal{T}|\boldsymbol{\theta},\{\tilde{k}_{p,c}\}) := \prod_p \left\{ \prod_c \mathcal{C}_{p,c} \exp\left[-\Delta \left(\frac{(\tilde{k}_{p,c}-\mu_{p,c})^2}{2\sigma^2_{p,c}} + \frac{\tilde{k}_c \cdot \bar{k}_{p,c}}{\bar{N}_c} - \frac{\bar{k}_{p,c}}{2\bar{N}_c} \right) \right] \cdot \prod_{j\in p} \frac{1}{2N(t_j)} \right\}. \tag{59}$$

Above, we've introduced the sum $\tilde{k}_c$ analogous to $\bar{k}_c$, defined as $\tilde{k}_c := \sum_p \tilde{k}_{p,c}$. The mean $\mu_{p,c}$ and variance $\sigma^2_{p,c}$ of the free variable $\tilde{k}_{p,c}$, as well as the normalization constants $\mathcal{C}_{p,c}$ are not yet specified. We make the following choices:

$$\mu_{p,c} = \bar{k}_{p,c}, \qquad \sum_p \sigma^2_{p,c} = \bar{N}_c, \qquad \mathcal{C}_{p,c} = \left(2\pi\sigma^2_{p,c}/\Delta\right)^{-1/2}. \tag{60}$$

The second equation does not fully specify $\sigma_{p,c}$; for concreteness, we choose $\sigma^2_{p,c} = \bar{N}_c/P_c$, where P_c is the number of partitions with active branches in cell c, i.e., where $k_{p,c} > 0$. We claim that the above choices lead to the augmented coalescent prior reducing to Eq. (58) once the augmenting variables $\tilde{k}_{p,c}$ are integrated out, that is,

$$\int \left\{ \prod_{p,c} d\tilde{k}_{p,c} \right\} \tilde{\pi}_{\text{anc}}(\mathcal{T}|\boldsymbol{\theta},\{\tilde{k}_{p,c}\}) = \bar{\pi}_{\text{anc}}(\mathcal{T}|\boldsymbol{\theta}). \tag{61}$$

The calculation is tedious but straightforward:

$$\begin{aligned}
&\int \left\{ \prod_{p,c} d\tilde{k}_{p,c} \right\} \tilde{\pi}_{\text{anc}}(\mathcal{T}|\boldsymbol{\theta},\{\tilde{k}_{p,c}\}) &(62)\\
&= \prod_{p,c} \int d\tilde{k}_{p,c}\, \mathcal{C}_{p,c} \exp\left[-\Delta \left(\frac{(\tilde{k}_{p,c}-\mu_{p,c})^2}{2\sigma^2_{p,c}} + \frac{\tilde{k}_{p,c} \cdot \bar{k}_c}{\bar{N}_c} - \frac{\bar{k}_{p,c}}{2\bar{N}_c} \right) \right] \cdot \prod_j \frac{1}{2N(t_j)}, &(63)\\
&= \prod_{p,c} \int d\tilde{k}_{p,c}\, \mathcal{C}_{p,c} \exp\left[-\Delta \left(\frac{\tilde{k}^2_{p,c}}{2\sigma^2_{p,c}} + \frac{(\tilde{k}_{p,c}+\mu_{p,c}) \cdot \bar{k}_c}{\bar{N}_c} - \frac{\bar{k}_{p,c}}{2\bar{N}_c} \right) \right] \cdot \prod_j \frac{1}{2N(t_j)}, &(64)\\
&= \prod_{p,c} \exp\left[\frac{(\Delta \bar{k}_c/\bar{N}_c)^2}{2\Delta/\sigma^2_{p,c}} \right] \cdot \exp\left[-\Delta \left(\frac{\mu_{p,c} \cdot \bar{k}_c}{\bar{N}_c} - \frac{\bar{k}_{p,c}}{2\bar{N}_c} \right) \right] \cdot \prod_j \frac{1}{2N(t_j)}, &(65)\\
&= \prod_{p,c} \exp\left[-\Delta \left(-\frac{\bar{k}^2_c \sigma^2_{p,c}}{2\bar{N}^2_c} + \frac{\mu_{p,c} \cdot \bar{k}_c}{\bar{N}_c} - \frac{\bar{k}_{p,c}}{2\bar{N}_c} \right) \right] \cdot \prod_j \frac{1}{2N(t_j)}, &(66)\\
&= \prod_{c} \exp\left[-\Delta \left(-\frac{\bar{k}^2_c}{2\bar{N}_c} + \frac{\bar{k}_c \cdot \bar{k}_c}{\bar{N}_c} - \frac{\bar{k}_c}{2\bar{N}_c} \right) \right] \cdot \prod_j \frac{1}{2N(t_j)}, &(67)\\
&= \exp\left[-\sum_c \binom{\bar{k}_c}{2} \frac{\Delta}{\bar{N}_c} \right] \cdot \prod_j \frac{1}{2N(t_j)}, &(68)\\
&= \bar{\pi}_{\text{anc}}(\mathcal{T}|\boldsymbol{\theta}). &(69)
\end{aligned}$$

The first equation follows from the relation $\sum_p \tilde{k}_c \cdot \bar{k}_{p,c} = \sum_{p,p'} \tilde{k}_{p,c} \cdot \bar{k}_{p',c} = \sum_p \tilde{k}_{p,c} \cdot \bar{k}_c$. The second equation follows from a change of variables $\tilde{k}'_{p,c} = \tilde{k}_{p,c} - \mu_{p,c}$. The third equation follows from the well-known Gaussian integral $\int dx\, \exp[-(ax^2+bx+c)] = \sqrt{\pi/a}\exp[b^2/4a-c]$. The fourth equation simply regroups terms, while the fifth simultaneously substitutes $\mu_{p,c}$ and $\sigma^2_{p,c}$ with the values in Eq. (60), turns the product over p of

exponentials into an exponential of sums over p, and evaluates them. The equality with $\bar{\pi}_{\mathrm{anc}}(\mathcal{T}|\boldsymbol{\theta})$ then follows straightforwardly.

The above transformation paves the way for parallelizing the coalescent prior. First, notice that for fixed $\{\bar{k}_{p,c}\}$, the variables $\{\tilde{k}_{p,c}\}$ can be Gibbs-sampled, because they are independent Gaussians. Concretely, the dependence of $\tilde{\pi}_{\mathrm{anc}}$ on a particular $\tilde{k}_{p,c}$ conditional on all other variables being fixed is:

$$\tilde{\pi}_{\mathrm{anc}}(\mathcal{T}|\boldsymbol{\theta}, \{\tilde{k}_{p,c}\}) \sim \exp\left[-\Delta\left(\frac{(\tilde{k}_{p,c} - \bar{k}_{p,c})^2}{2\sigma^2_{p,c}} + \frac{\tilde{k}_{p,c}\cdot\bar{k}_c}{\bar{N}_c}\right)\right], \tag{70}$$

$$\sim \exp\left[-\frac{\left(\tilde{k}_{p,c} - \left\{\bar{k}_{p,c} - \frac{\bar{k}_c\sigma^2_{p,c}}{\bar{N}_c}\right\}\right)^2}{2\sigma^2_{p,c}/\Delta}\right], \tag{71}$$

$$\tag{72}$$

and so

$$\tilde{k}_{p,c} \sim \mathcal{N}\left(\bar{k}_{p,c} - \frac{\bar{k}_c\sigma^2_{p,c}}{\bar{N}_c}, \sigma^2_{p,c}/\Delta\right), \qquad \text{for fixed } \{\bar{k}_{p,c}\}. \tag{73}$$

Second, for fixed $\{\tilde{k}_{p,c}\}$, Eq. (59) is a product of independent terms, one for each partition p. Hence, we can make independent local moves in each partition p with only knowledge of $\tilde{k}_{p,c}$, $\tilde{k}_c$ and $\bar{N}_c$.

The above considerations lead to the following parallelization scheme:

1. Cut up the tree $\mathcal{T}$ into P partitions.

2. For every cell c, calculate $\bar{k}_{p,c}$ for every partition p and then calculate their sum $\bar{k}_c$.

3. For every cell c, sample random Gaussian fields $\tilde{k}_{p,c}$ for every partition p as per Eq. (73), then calculate their sum $\tilde{k}_c$.

4. Distribute the P partitions over P parallel workers. Worker p receives the subtree in partition p, and the full set of values for $\tilde{k}_{p,c}$ and $\tilde{k}_c$.

5. Apply local MCMC moves within each worker using the augmented coalescent prior of Equation (59). By the above arguments, each worker has enough information to do this correctly and independently.

6. Reassemble the modified subtrees from each of the workers into a global tree.

7. Apply global MCMC moves, such as changes in mutation rates or evolution model parameters. This is done with the discretized coalescent prior given by Eq. (57).

Intuitively, at the beginning of each cycle, a central process has a global view of the tree. It takes a fuzzy "snapshot" of this global view, in the form of $\{\tilde{k}_{p,c}\}$ and $\tilde{k}_c$. Then, each worker adjusts the various $\{\bar{k}_{p,c}\}$ in accordance with that earlier snapshot, not in accordance to the instantaneous $\bar{k}_c$, which they cannot access. Periodically, the tree is reassembled to be repartitioned and resnapshotted. Statistically, this process ends up transmitting the global knowledge needed to impose the coalescent prior to every worker.

A subtle disadvantage of the partitioning scheme presented in this section is that the partitioning enters the posterior distribution indirectly, so that factors arising from the partitioning in principle enter the acceptance probability of all MCMC moves. We describe the problem in more detail below, and present our strategy for mitigating it almost completely.

9.1 Partitioning a tree into P partitions

The key mechanism for parallelization in Delphy is to partition the tree $\mathcal{T}$ into roughly P partitions, and then perform local moves in all partitions in parallel. The basic scheme is inspired by JUNIPER [33], and is itself based on the work of Borndörfer, Elijazyfer and Schwartz [4]. It identifies a series of nodes, which we call *cut points*, where one subtree ends and another begins. Each subtree $\mathcal{T}_p$ is rooted at either the root node of $\mathcal{T}$ or one of the cut points, and extends as far as the closest descendant cut points or the tips of the original tree, whichever is closest. A simple heuristic tries to make the subtrees of roughly equal size, and we add an element of randomness to ensure that any particular point on the tree has a decent chance of being inside a subtree instead of at a boundary.

In concrete terms, we choose the cut points with the following algorithm. We perform a post-order traversal of the tree, descending from a node to its children in random order. In the process, we keep track of the number of nodes m yet to be visited (initially $2N-1$) and the number of partitions p yet to be formed (initially P). Throughout, we also construct the number of descendants n_i of each visited node i; if i is a tip, then $n_i = 1$, otherwise $n_i = 1 + \sum_j n_j$, where the sum runs over the children of i. If at any point n_i is at least $\lambda = \max(10, m/(p+1))$, then node i is considered a viable cut point. If the number of nodes remaining to visit is also at least λ, and a random number $u \sim U(0,1)$ satisfies $u < 0.5$ (i.e., a fair coin toss), then node i becomes an actual cut point. We record it, then set $n_i = 0$ before continuing the walk. Finally, we always record the root node as a cut point. The various thresholds and randomizations ensure that no one partition is too large or too small; that if an overlay small or large partition is formed by chance, then the target size for the remaining partitions is adjusted accordingly; and that different executions of the algorithm can yield different partitionings of the same tree.

As long as partitionings happen regularly and they only enter in the proposal probabilities, such as by limiting the possible regrafting points P' of an SPR proposal to the same partition that contains the pruned subtree, the exact partitioning details do not generally affect the results of the MCMC. However, as mentioned above, our scheme for the coalescent prior goes beyond that: the details of the partitioning appear directly in the posterior distribution. To be concrete, suppose that the probability of the above algorithm producing a partition Υ given a tree $\mathcal{T}$ and parameters $\boldsymbol{\theta}$ is $P(\Upsilon|\mathcal{T},\boldsymbol{\theta})$. We can conceive of the parallelized MCMC as sampling from an augmented state space consisting of trees $\mathcal{T}$, parameters $\boldsymbol{\theta}$, partitions Υ and the partition-dependent variables $\{\tilde{k}_{p,c}\}$. The posterior distribution sampled by this MCMC is actually

$$P_{\|}(\mathcal{T},\boldsymbol{\theta},\Upsilon,\{\tilde{k}_{p,c}\}) \propto P(\{\tilde{k}_{p,c}\}|\Upsilon,\mathcal{T})P(\Upsilon|\mathcal{T})P(\mathcal{T},\boldsymbol{\theta}).$$

Here, the last factor is the posterior distribution that we actually want to sample (Eq. (3)), while

$$P(\{\tilde{k}_{p,c}\}|\Upsilon,\mathcal{T}) = \frac{\tilde{\pi}_{\text{anc}}(\mathcal{T}|\boldsymbol{\theta},\{\tilde{k}_{p,c}\})}{\bar{\pi}_{\text{anc}}(\mathcal{T}|\boldsymbol{\theta})}.$$

As detailed in the previous section, $P(\{\tilde{k}_{p,c}\}|\Upsilon,\mathcal{T})$ is such that the entire posterior can be decomposed into independent factors for each partition, while its integral over all possible values of $\{\tilde{k}_{p,c}\}$ is exactly 1 (Eq. (61)).

The key flaw in $P_{\|}(\mathcal{T},\boldsymbol{\theta},\Upsilon,\{\tilde{k}_{p,c}\})$ is that, conditioned on $\{\tilde{k}_{p,c}\}$ being fixed, the factor $P(\Upsilon|\mathcal{T})$ depends on $\mathcal{T}$, so moves that propose a change in the topology of a subtree, say from $\mathcal{T}_{\mathsf{o}}$ to $\mathcal{T}_{\mathsf{n}}$, require a factor of $P(\Upsilon|\mathcal{T}_{\mathsf{n}})/P(\Upsilon|\mathcal{T}_{\mathsf{o}})$, whose neglect leads to incorrect sampling. We have verified empirically in a simple toy setting of a 2-tip tree whose MRCA is restricted to one of a finite number of positions, and a simplified partitioning scheme that admits an exact expression for $P(\Upsilon|\mathcal{T})$, that: (a) failing to account for $P(\Upsilon|\mathcal{T})$ does lead to incorrect sampling; and (b) adding the missing ratios of this probability to the Metropolis-Hastings criterion restores correct sampling.

One correct but inefficient solution to the above problem is to pick a partitioning scheme where $P(\Upsilon|\mathcal{T})$ is a constant, so that the "missing" correction factor in the Metropolis-Hastings criterion is just 1. One such scheme would be to choose P cutpoints uniformly at random from the $N-1$ inner nodes of the tree, subject to one of these being the root node. However, most inner nodes are close to a tip; for example, in the limit where all the tips occur at the same time and the tree is a complete binary tree, then around half

of the inner nodes are the parent of two tips. Hence, this simple scheme tends to produce $P-1$ partitions with a handful of nodes, and a single enormous partition with the remainder of the tree, which renders parallelization utterly ineffective.

Given the above considerations, we instead introduce the following controlled approximation. Periodically, we choose a set of D fixed partitionings (currently, 10), which we call "stencils". Throughout the MCMC, we sample partitionings uniformly among these D stencils, so that Metropolis-Hastings correction owing to $P(\Upsilon|\mathcal{T})$ is again 1. After sufficiently many MCMC steps, the samples drawn from $P_\|$, when projected to remove Υ and $\{\tilde{k}_{p,c}\}$, are indistinguishable from those drawn from $P(\mathcal{T}|\boldsymbol{\theta})$ (indeed, this is the essential property of the augmented coalescent prior). At that point, it we pick a new set of D stencils, and start this cycle again.

In detail, the final approximate scheme we have implemented in Delphy is as follows:

1. Use any algorithm, such as the one at the beginning of this section, to produce D distinct stencils. Each stencil consists of the node identifiers of all the cutpoints, so any topology of $\mathcal{T}$ can be partitioned according to a particular stencil.

2. Proceed with the MCMC as described at the end of Section 9, whereby "cut up the tree $\mathcal{T}$ into P partitions" means "sample one of the D stencils uniformly and use it to partition $\mathcal{T}$".

3. After many rounds of the previous step (currently, 100), go back to the Step 1 to produce a fresh new set of D stencils.

We have verified in our toy 2-tip system that such uniform cycling over fixed stencils, with infrequent periodic redrawing of the stencils, recovers correct MCMC sampling, even in the face of complex distributions for $P(\Upsilon|\mathcal{T})$. The excellent agreement of Delphy's results with the BEAST benchmark results also suggests that the scheme works in the general setting of complex trees. We note that as one of the above cycles progresses, and inner nodes in the tree move, the stencils used to partition the tree become increasingly ill-fitting, and would eventually degrade to the random-cut-points scheme of the previous paragraph if they are not refreshed. Refreshing every 100 iterations of Step 2 seems to be a reasonable compromise between complete correctness and efficiency.

10 Overall schedule of MCMC moves

Having described all local and global moves, as well as our parallelization scheme, we briefly sketch out how these are assembled in an overall move schedule. Most of these choices are arbitrary; many alternative choices would also be correct, but may impact the efficiency of sampling. Our concrete choices below are inspired by the BEAST run in [18]:

- Inside an outer loop, repeatedly create $D = 10$ partition stencils, then run the following steps 100 times (see previous section):
  - Inside a middle loop: (1) run a fixed schedule of global moves; (2) partition the tree into subtrees according to one of the D stencils chosen at random; (3) run a fixed number of independent local moves in each subtree in parallel; (4) reassemble the tree from the modified subtrees. Concretely:
    - Run the following global moves:
      - A Gibbs sample of μ, as described in Section 7.1.
      - A series of 10 delta-exchange moves to the HKY stationary base frequencies $\{\pi_a\}$ interleaved with 10 scaling moves of the HKY κ parameter, as described in Section 7.2. This approximates a Gibbs sample of the evolution model parameters.
      - When site-rate heterogeneity is enabled: a series of 10 scaling moves of the α parameter after marginalizing the individual relative rates $\{\nu^{(\ell)}\}$, followed by Gibbs sampling of these rates, as described in Section 7.4. This approximate a Gibbs sample of the site-rate heterogeneity parameters.

- · For an exponentially growing population model, a series of 50 scaling moves on the population size n_0 interleaved with 50 uniform delta moves on the population growth rate g. This approximate a Gibbs sample of the population model parameters.
- · For a Skygrid population model, an optional Gibbs move for the precision τ (if inferred, not the default), followed by a Gibbs move on the overall population level, followed by a single HMC move on the population curve parameters $\{\gamma_k\}$.

∗ We partition the tree into around P subtrees using one of the D stencils. We then calculate the number of active branches $\{k_{p,c}\}$ in each partition and cell, and use them to Gibbs sample values for $\{\tilde{k}_{p,c}\}$ of the augmented coalescent prior, as described in Section 9, as well as their sums $\{\tilde{k}_p\}$.

∗ We then dispatch an inner loop to as many independent workers as there are partitions. Worker p receives a copy of subtree p, the values of $\{\tilde{k}_{p,c}$ for its p, as well as the sum $\tilde{k}_c$, and copies of all the global model parameters: μ, $\{\nu^{(\ell)}\}$, $\{\pi_a\}$, $\{q_a\}$, $\{q_{ab}\}$, n_0 and g. Each worker then independently executes W_p steps in an inner loop, where W_p is a large multiple of the number of nodes in subtree p (by default, 50). The intent is to locally equilibrate the subtree conditional on the global parameters and the fixed times and sequences of the boundary nodes between it and its ancestral and descendant subtrees. Each local move is chosen at random from the following weighted list:

- · Inner node displacement move (weight: 15.0).
- · Branch reform move (weight: 15.0)
- · Subtree slide move (weight: 1).
- · Mutation-directed SPR moves (weight: 1; around 1 % of these explore the entire subtree of worker p, while the rest explore only the vicinity of P up to 2 mutations away; see Section 6.6.1).

∗ The partitions are always reassembled so that at the end of each iteration of the middle loop, where we can sample a complete configuration of the tree and the associated global parameters. We also use this moment to change the reference sequence to match the current root sequence, which requires an expensive walk of the tree to adjust all missations; while not required, this step avoids trapping the MCMC in a state where the root and reference sequences differ a lot, which would make root-node-changing moves unduly expensive.

We note that our current implementation of the parallelization scheme is merely a proof-of-concept to show that parallelization is possible. There is a lot of needless copying and recalculation when partitioning the tree, distributing the subtrees to workers and then reassembling the full tree from the modified subtrees. Further, while the above scheme in principle supports distributing the work over a cluster of networked machines, our implementation only distributes work among the CPU cores of a single machine. We expect that engineering improvements in both of these areas will unlock scaling to much larger datasets than we have yet demonstrated here.

11 Fast MCC trees, approximate incremental MCC trees and fast Robinson-Foulds distance calculations

Delphy's web interface rederives an MCC every time a new posterior tree is sampled, so it needs to do this quickly. In contrast, BEAST2's TreeAnnotator takes tens of seconds to deduce the MCC of the SARS-CoV-2 results from Lemieux et al [18]. Here, we describe how we derive such MCCs in milliseconds instead, using a technique inspired by hash trees (also known as Merkle trees) [6, 23, 24].

First, recall how MCCs are usually derived. For each posterior tree, we enumerate all its *clades*, i.e., the sets of tips descended from each node. Any particular clade is present in a fraction of the posterior trees, called the clade's *posterior support*. The MCC is defined as a tree with: (a) the topology of the posterior

tree with highest product of posterior support over all its nodes; and (b) node times that summarize those of the corresponding nodes in the posterior trees. Most commonly, these latter times are the average (or median) of the times of the inner node for the same clade in each posterior tree where it is present.

What most slows down TreeAnnotator's MCC calculation is its representation of clades as explicit sets of tip indices. This leads to high memory consumption and high runtimes as explicit sets are built, inserted and retrieved from hash tables, and compared to each other. Instead, we introduce a probabilistic approach below that has proven much more effective in practice.

In Delphy's MCC calculation, we represent the clade of a node i as a fixed-size C-bit fingerprint F_i. We assign uniformly random fingerprints to tips: the probability $p_1(F_i)$ of any one particular choice is exactly $1/2^C$. The fingerprint of a clade that is the union of two smaller and disjoint clades is calculated as the exclusive-or (XOR) of those two clade's fingerprints. Hence, these fingerprints can be calculated very efficiently and in time linear in the tree size for a single posterior tree, via a post-order traversal of the tree. When this procedure completes, the fingerprint for an arbitrary inner node is the XOR of the fingerprints of all its descendant tips:

$$F_i = \bigotimes_{\text{tips } j \text{ below } i} F_j.$$

Since the tip fingerprints are uniformly random, then so are the fingerprints of the inner nodes (marginally): each leaf fingerprint added to the clade at an inner node acts as a one-time pad that perfectly scrambles the fingerprint of that clade. More generally, the fingerprints of any pair of distinct clades A and B are pairwise independent. Concretely, this means that the joint distribution of fingerprints $p_2(F_A, F_B)$ is just the product of the marginal distributions for each fingerprint, $p_1(F_A)$ and $p_1(F_B)$: whatever the fingerprint F_A of A is, the addition or removal of even a single tip from A to reach B introduces an XOR of a uniformly random fingerprint into F_B, making F_B uniformly random too even when conditioned on F_A. In particular, this implies that the probability that the fingerprints of any pair of distinct clades A and B are equal is exactly $1/2^C$. Although pairwise independence does not extend to higher orders (e.g., consider a clade C consisting of the symmetric difference of A and B, whose fingerprint F_C is determined given F_A and F_B, by $F_C = F_A \otimes F_B$), pairwise independence is sufficient for bounding collision probabilities, as we do below.

The calculation of an MCC of M posterior trees, each with N tips, involves at most D distinct clades, where

$$D = N + 1 + M(N-2) \approx MN.$$

Of these, N are the trivial singleton clades, one is the root clade, and in principle, all the $N-2$ nonroot inner nodes of all M trees could represent different clades. The fingerprints of these up to D clades are, as remarked above, pairwise independent and uniformly random C-bit strings. Owing to the union bound, the probability p of a collision, i.e., that any pair of fingerprints is equal, is bounded by above by the sum over all possible pairs that a single pair is equal (though it may be lower owing to nonindependence of fingerprints for triplets, etc.). That is,

$$p \leq \binom{D}{2} \frac{1}{2^C} \leq \frac{D^2}{2 \cdot 2^C}.$$

We can thus reduce the probability of a fingerprint collision to below an arbitrary level p by using a sufficiently large value of C, as follows:

$$C \geq \log_2 \left[\frac{D^2}{2p} \right].$$

Concretely, using $C = 64$ bits for 1000 posterior tree samples with 1000 inner nodes, yields $p < 2.8 \times 10^{-8}$. For pandemic scales, e.g., 1000 trees with 10 M inner nodes, one would need $C = 86$ to achieve $p < 10^{-6}$. The natural choice of $C = 128$ bits would lead to $p < 1.5 \times 10^{-19}$. Hence, we see no practical danger in using 64-bit fingerprints for near-real-time analysis of smaller datasets and 128-bit fingerprints for pandemic-scale datasets.

In practice, we build an MCC in four passes. First, we build a map of clade fingerprints to lists of tuples "(posterior tree sample index, node index)". Second, we calculate the log-credibility of each clade i as $\log(M_i/M)$, where M_i counts how many posterior trees contain clade i. Third, we compute for each

posterior tree the sum of the log-clade-credibilities of each inner node, and record the tree with the highest sum. This tree defines the topology of the MCC. Finally, we iterate over the nodes of the MCC, and for each one, we iterate over the corresponding nodes in the posterior trees to calculate the mean (or median) node time of each MCC node. The node $\phi_m(i)$ in a posterior tree m corresponding to a given node i in the MCC is the MRCA in the posterior tree m of the nodes corresponding to the child nodes of i in the MCC, i.e., $MRCA\{\phi_m(j)|j \text{ child of } i \text{ in MCC}\}$. Hence, node correspondences for a single posterior tree can be calculated efficiently in $\mathcal{O}(N)$ time (in aggregate, assuming all inner node times are unique, the MRCA calculations walk over every branch in the posterior tree exactly once). If all corresponding nodes are included when calculating the mean (median) time, we mimic the behavior of TreeAnnotator's `--heights ca` option; if only exact matches are included, where the clade fingerprint of $\phi_m(i)$ and i are equal, we obtain the more standard MCC. Neglecting the weak $\log N$ dependence of C, which we have argued above is immaterial for all practical datasets, this four-pass algorithm runs in time $\mathcal{O}(MN)$, i.e., linear in the size of the results of the MCMC.

We note that the above fingerprinting techniques equally benefit similar algorithms for calculating other kinds of recently introduced summary trees, such as CCD0 [3] and HIPSTR [2]. It also benefits Robinson-Foulds distance calculations, as detailed below.

11.1 Incremental calculation of MCCs

For continuously visualizing the MCC for an ongoing MCMC run, the above algorithm can still be very expensive, repeats large amounts of work, and slows down as the inference progresses. Concretely, continuously recalculating the MCC as M grows results in an overall unacceptable $\mathcal{O}(M^2N)$ cost. While this has so far proven acceptable for interactive use in the Delphy web interface, we outline a proposal below for incremental MCC calculation that may prove useful for larger datasets and/or longer runs. We initially tested this proposal and verified its effectiveness, but since from-scratch recalculation of MCCs after every sample proved sufficient in the Delphy web interface, we have not implemented this idea in production.

We observe that the MCC derived from a sequence of posterior tree samples ($\mathcal{T}_1, \ldots, \mathcal{T}_M$) is usually very similar to that derived from a sequence extended with one more sample, $\mathcal{T}_{M+1}$. Most of the time, the topology is unchanged, while the node times make subtle adjustments. But occasionally, the identity of the posterior tree sample with highest clade credibility changes, in which case the tree topology changes drastically.

We exploit the above insight by adapting "beam search" to the problem of incrementally building MCCs. Concretely, when a new posterior tree sample is added, we can calculate its inner node clade fingerprints (incremental pass 1) and update the map of clade fingerprints to pointers to posterior tree nodes (incremental pass 2) in $\mathcal{O}(N)$ time. Then, instead of recalculating the clade credibility of all the $M + 1$ posterior tree samples in $\mathcal{O}(MN)$ time, we simply recalculate it for a "beam" of K such trees in $\mathcal{O}(KN)$ time (incremental pass 3), which closely tracks the K trees with highest clade credibilities. We also update the clade credibility of a random posterior tree sample, and if it is higher than the lowest-clade-credibility tree in the beam, it replaces that tree in the beam. We then pick the highest-clade-credibility tree in the beam for the MCC topology. In the likely case that the MCC topology did not change when adding $\mathcal{T}_{M+1}$, then the node times can be incrementally updated in $\mathcal{O}(N)$ time; otherwise, we recalculate them from scratch in $\mathcal{O}(MN)$ time. Overall, then, adding a single posterior tree sample to an incremental MCC takes $\mathcal{O}(N)$ time, and the cost of continuously recalculating the MCC throughout a run is close to $\mathcal{O}(MKN)$ time.

In essence, we obtain an M/K speed-up by trading away some accuracy: the MCC topology might not always correspond to that of the highest-clade-credibility posterior tree sample. This trade-off seems acceptable for the real-time updating of the MCC of an ongoing MCMC run, though it's essential to recalculate the MCC correctly from scratch at the end of a run for further analysis. The parameter K controls the lag between changes in the real MCC topology and that of this incremental MCC. Empirically, we have found that $K = 5$ works sufficiently well to continuously visualize a reasonably accurate MCC during the course of an MCMC run.

11.2 Fast calculation of `frechetCorrelationESS` and Robinson-Foulds distances

To assess tree topology convergence, we implemented the `frechetCorrelationESS` measure described in [19], which quantifies the rate at which pairwise Robinson-Foulds distances between trees tend to their long-term expected value with increasing separation in the run. Our actual implementation simplifies the treatment of finite data sizes in Eq (9) of [19] to arrive at this formula for the normalized autocorrelation function:

$$\rho(t) = 1 - \frac{1}{2}\frac{\mathbb{E}[\Delta^2(t)]}{\mathrm{Var}[\Delta]}.$$

Here, $\mathbb{E}[\Delta^2(t)]$ is the expected value of the squared-distance between two trees separated by t steps, and $\mathrm{Var}[\Delta]$ is the average squared-distance between two randomly sampled trees. While this formula is written in terms of only pairwise distances, for 1-dimensional quantities, it reduces to the standard formula for normalized auto-correlation written in terms of fluctuations from the mean. For the above, we also discarded the first 30% of the runs as burn-in. ESS is derived from $\rho(t)$ as described in [19].

Underlying $\mathbb{E}[\Delta^2(t)]$ and $\mathrm{Var}[\Delta]$ above is a dense "RF distance matrix", with entry ij the Robison-Foulds (RF) distance [32] between trees i and j in the production portion of an MCMC run. As a reminder, the RF distance characterizes the difference in topology between two trees. It first projects each tree onto a set of "splits", the pairs of tip sets on etiher side of every branch. The distance between two trees is then the number of splits that are present in one tree but not the other.

Clade fingerprinting provides an efficient device for calculating the RF distance matrix of a run. Notably, there is a 1-1 correspondence between splits and clades if (as we do) the point $\perp$ above the root node is regarded as a tip for the purpose of RF distance calculation (implying that otherwise topologically identical trees that are rooted differently have a nonzero RF distance). In particular, for a given branch, the "split" can first be identified with the set of tips that does not contain $\perp$; this tip set can then be identified with the clade defined at the end of the branch that is furthest from $\perp$; and finally that clade can be uniquely identified with its fingerprint with high probability.

In the `calc-tree-ess` program included in our benchmark, we calculate the full RF distance matrix as follows:

- Calculate all clade fingerprints as above.
- Store a sorted list of clade fingerprints for every tree (excluding the fingerprints of the tips and the root node, which are common to all trees).
- For every pair of trees, merge these sorted clade fingerprint lists to count all splits that are unique to one tree or another.

In practice, even though `calc-tree-ess` is single-threaded, it only takes about 20 seconds on a laptop (Intel Core Ultra 7 165H at 5.0 GHz) to calculate all entries in the 700×700 RF distance matrix for all post-burn-in trees in one of our H5N1 runs (each tree having 3,339 tips and thus 3,337 nontrivial splits).

12 Lineage and mutation prevalence curves

Delphy's interface allows a user to mark one or more of a tree's inner nodes, indexed by A, each of which can be regarded as the founding virion of a particular lineage; the root node is always implicitly marked in this way. It then calculates "lineage prevalence curves" $u_A(t)$ for all marked nodes. These are the probability distribution of the closest marked ancestor of a random member of the total viral population at time t. Here, we describe how that calculation is performed.

First, consider a single posterior tree. Let $k_A(t)$ be the number of active lineages at time t whose closest marked ancestor is A. In the coalescent model, if we draw a random member of the total viral population at time t, the probability that it coalesces with one of these $k_A(t)$ active lineages between times $t - dt$ and t is

given by $[k_A(t)/N(t)]\,dt$. Hence, the probability that it does not coalesce at all is given by $1 - [k(t)/N(t)]\,dt$, where $k(t) = \sum_A k_A(t)$. Thus, $u_A(t)$ obeys the following differential equation:

$$\frac{du_A(t)}{dt} = \left[\frac{k_A(t)}{N(t)}\right] - \left[\frac{k(t)}{N(t)}\right] u_A(t) \tag{74}$$

$$= \left[\frac{k(t)}{N(t)}\right] \left(\frac{k_A(t)}{k(t)} - u_A(t)\right). \tag{75}$$

The initial condition that $u_A(t) = 0$ for all A and all t preceding the root node time completely determines $u_A(t)$. The solution to this differential equation is an exponentially moving average of $[k_A(t)/k(t)]$ with decay rate $[k(t)/N(t)]$, as follows:

$$u_A(t) = \int_{-\infty}^{t} dt'\, \frac{k_A(t')}{k(t')} \cdot \frac{k(t')}{N(t')} \exp\left(-\int_{t'}^{t} dt''\, \frac{k(t'')}{N(t'')}\right). \tag{76}$$

Delphy solves the above differential equation numerically, but the analytical solution makes the meaning of $u_A(t)$ clearer. When $k(t)$ is large relative to $N(t)$ and the rate at which these two quantities vary is low, the tree can be thought of as densely sampled at time t: since the rate of coalescence is high but the number of active lineages is not varying quickly, most active lineages are immediately upstream of a tip. In that regime, $u_A(t)$ closely approximates $k_A(t)/k(t)$, which is essentially the lineage prevalence of A that would be estimated by direct case counts. However, outside that regime, $u_A(t)$ integrates all the available information from the population model and the rest of the tree to smooth out such direct case count estimates.

When a user selects an inner node X on the MCC in Delphy's Lineages panel, we interpret that as the smallest clade that contains the particular set S of tips downstream of X in the MCC. Correspondingly, in each posterior tree, that action marks the MRCA of the tips S, which is well-defined for all posterior trees. When a single inner node in the tree is marked multiple times, ties are broken arbitrarily. The corresponding lineage prevalence curves are calculated for each posterior tree, and the mean and 95% HPD range of each such family of curves is displayed to the user.

A completely analogous procedure is used to calculate the "mutation prevalence curves" in the Mutations panel. For a selected mutation (a, ℓ, b), we calculate the probability $u(t)$ that a random member of the total viral population at time t has as its closest ancestor in the tree a virion where site ℓ has state b. As above, $u(t)$ is calculated for all posterior trees, and the mean and 95% HPD at each value of t is displayed to the user.

13 APOBEC3-aware evolution model for mpox

Inspired by [29, 30], we have added an option in Delphy to use a specialized evolution model suitable for mpox sequences, which can be activated in the web interface under "Advanced Options". We first classify each site as having APOBEC3 context according to the sequence of the first sample in the input file (with missing sites replaced by the consensus across all input sequences). A site has APOBEC3 context if it and the preceding site form a `TC` or a `TT` dimer, or if it and its subsequent site form a `GA` or `AA` dimer. For sites without APOBEC3 context, we use a transition rate matrix for a Jukes-Cantor evolutionary model with mutation rate μ and no site-rate heterogeneity:

$$Q_0 = \mu \begin{pmatrix} -1 & 1/3 & 1/3 & 1/3 \\ 1/3 & -1 & 1/3 & 1/3 \\ 1/3 & 1/3 & -1 & 1/3 \\ 1/3 & 1/3 & 1/3 & -1 \end{pmatrix}.$$

In sites with APOBEC3 context, we add transitions from `C` $\to$ `T` and `G` $\to$ `A` at a rate μ^* as follows:

$$Q_1 = \mu \begin{pmatrix} -1 & 1/3 & 1/3 & 1/3 \\ 1/3 & -1 & 1/3 & 1/3 \\ 1/3 & 1/3 & -1 & 1/3 \\ 1/3 & 1/3 & 1/3 & -1 \end{pmatrix} + \mu^* \begin{pmatrix} 0 & 0 & 0 & 0 \\ 0 & -2 & 0 & +2 \\ +2 & 0 & -2 & 0 \\ 0 & 0 & 0 & 0 \end{pmatrix}.$$

The factor of 2 is chosen to match the conventions used in the BEAST runs to define the APOBEC3 rate (the effective rate at which transitions would be observed in a sequence of APOBEC3 sites that are 50% `C/G` and 50% `T/A`).

The above setup retains the essence of that of the BEAST runs in [29, 30] but differs minimally in its details. By deciding on the APOBEC context using the first sequence, we avoid having to previously estimate an ML tree and extract its root sequence (the assumption is that the APOBEC3 context of the vast majority sites is stable throughout the entire tree), and allows us to handle arbitrary new datasets, possibly aligned to a different reference, without complications. By preserving the polymerase mutation mechanism in sites with APOBEC3 context (i.e., the first term in the definition of Q_1), we avoid having to identify only the sites that have not yet mutated (i.e., only the `TC` and `GA` sites) and avoid technical difficulties with completely irreversible transition matrices. For simplicity, we also use a simple Jukes-Cantor model with no site rate heterogeneity instead of a more sophisticated GTR model with 4 gamma rate categories. We model the viral population as growing strictly exponentially, as observed in [29, 30]. Finally, by design, we have not implemented a transition between a pre-spillover and post-spillover portion of the tree, as our interest is in public health response to a growing outbreak that is known to be spreading through humans.

14 Sampling difficulties evidenced by clade correlation graphs

In the main text, we claim that outliers in the clade correlation graphs are the result of sampling difficulties involving a few misplaced tips. In this section, we justify this claim and show evidence that while BEAST X and BEAST 2 have fewer such sampling difficulties, they are not immune to them. We believe these sampling difficulties reflect the fundamental ruggedness of tree space explored in [10]. While Delphy's mdSPR move is usually very effective at exploring tree space without specific user tuning, future enhancements will be required to alleviate the specific sampling difficulties demonstrated here and in the next section (e.g., by extending the region of the tree whose mutational history is reconfigured to more than just the branch above the pruned subtree's root X).

Supplementary Figure 20 shows six clade correlation graphs in addition to those already shown in the main text and in earlier sections of the SI, all for runs without site rate heterogeneity (to make Delphy and BEAST runs directly comparable). Each column corresponds to the labeled dataset. The top row (panels A, B, and C) shows clade correlation graphs between separate but identically configured Delphy runs. The bottom rows show similar graphs either between identically configured BEAST X runs (panels D and E), or similarly a BEAST X run against an identically configured BEAST 2 run (panel F).

The Delphy clade correlation graphs for the large Ebola dataset (panel A) shows that while the vast majority of clades have compatible support between the runs, there are a few significant outliers. We analyze this comparison in detail below. By contrast, the comparison of the two analogous BEAST X runs (panel D) has no evidence of sampling difficulties. The picture is more subtle for the larger H5N1 dataset: here, both Delphy (panel B) and BEAST X (panel E) exhibit sampling difficulties to a comparable extent. Finally, the graphs for the moderately sized SARS-CoV-2 dataset show little evidence of incompatibility in clade support between identical Delphy runs (panel C), though some small sampling difficulties are evident in the handful of outliers. As a baseline, we show an analogous comparison between a BEAST X run and an identically configured BEAST 2 run (panel F), which shows similar levels of compatibility and outliers. We emphasize that panels E and F are constructed entirely with established tools that have been battle-tested for decades, so the discrepancies are unlikely to reflect bugs in implementation.

To further understand the outliers in the Delphy runs for the large Ebola dataset (panel A), we identified all clades with at least 20 tips whose support in both runs differed by more than 10 %. We first sorted them by size and clustered them (cluster boundaries whenever a size gap of more than 3 tips appears in this list). Then, we separated the members of each size cluster into connected components, two clades being connected if they shared at least 50 % of the smaller clade's tips in common. This procedure unmistakably separates the suspicious clades into small clusters whose tip membership differs only by a handful of tips[2]. In Table 3,

[2]See `ebola-dudas-2017/32_suspicious_clades.py` and `ebola-dudas-2017/analysis/suspicious_clades_delphy_a_vs_b_0p10_report.tsv` in the data repo.

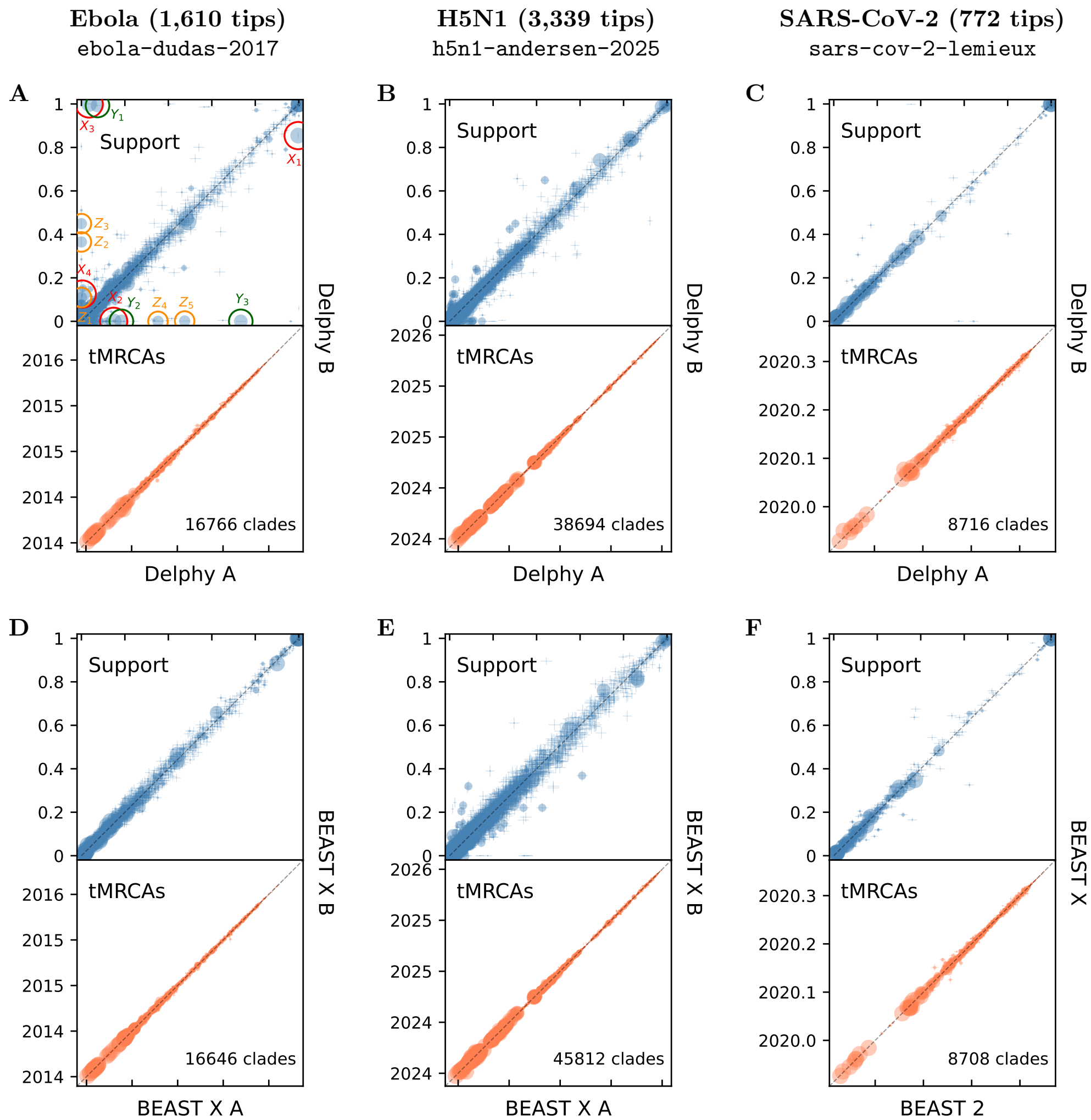

Supplementary Figure 20: Clade correlation graphs evidencing sampling difficulties. Each column corresponds to the labeled data sets. Panels in the top row compare identically configured Delphy runs. Panels in the bottom row similar compare BEAST X with BEAST X or BEAST 2 runs. All runs have site-rate heterogeneity disabled. **A.** Delphy on Ebola dataset (see text and Table 3 for discussion of hiighlighted clades). **B.** Delphy on H5N1 dataset. **C.** Delphy on SARS-CoV-2 dataset. **D.** BEAST X on large Ebola dataset. **E.** BEAST X on H5N1 dataset. **F.** BEAST 2 vs. BEAST X on the SARS-CoV-2 dataset.

<table>
<tr><th>Cluster</th><th>Common tips</th><th>Mean tMRCA spread</th><th>Clade</th><th>Support Run A</th><th>Support Run B</th><th>Unique additional tips</th></tr>
<tr><td rowspan="4">X</td><td rowspan="4">1463</td><td rowspan="4">2014-04-17 – 2014-05-03</td><td>X_1</td><td>100%</td><td>85%</td><td>EBOV-EM_000500-KR817076-GIN-Macenta|2014-09-13
EBOV-EM_080223-KR817241-LBR-?|2014-07-04</td></tr>
<tr><td>X_2</td><td>15%</td><td>0%</td><td>EBOV-EM_000500-KR817076-GIN-Macenta|2014-09-13</td></tr>
<tr><td>X_3</td><td>4%</td><td>100%</td><td>EBOV-EM_080223-KR817241-LBR-?|2014-07-04</td></tr>
<tr><td>X_4</td><td>0%</td><td>13%</td><td>—</td></tr>
<tr><td rowspan="3">Y</td><td rowspan="3">1098</td><td rowspan="3">2014-05-14 – 2014-05-15</td><td>Y_1</td><td>7%</td><td>99%</td><td>EBOV-1100--GIN-Conakry|2014-09-23
EBOV-1283--GIN-Coyah|2014-10-04</td></tr>
<tr><td>Y_2</td><td>18%</td><td>0%</td><td>EBOV-1283--GIN-Coyah|2014-10-04</td></tr>
<tr><td>Y_3</td><td>73%</td><td>0%</td><td>—</td></tr>
<tr><td rowspan="5">Z</td><td rowspan="5">752</td><td rowspan="5">2014-05-31 – 2014-06-08</td><td>Z_1</td><td>0%</td><td>11%</td><td>EBOV-1100--GIN-Conakry|2014-09-23
EBOV-1283--GIN-Coyah|2014-10-04
EBOV-G3855.2-KR105203-SLE-Kenema|2014-06-17</td></tr>
<tr><td>Z_2</td><td>0%</td><td>37%</td><td>EBOV-1100--GIN-Conakry|2014-09-23
EBOV-1283--GIN-Coyah|2014-10-04
EBOV-G4200.1-KR105217-SLE-Kenema|2014-07-08</td></tr>
<tr><td>Z_3</td><td>0%</td><td>45%</td><td>EBOV-1100--GIN-Conakry|2014-09-23
EBOV-1283--GIN-Coyah|2014-10-04</td></tr>
<tr><td>Z_4</td><td>35%</td><td>0%</td><td>EBOV-G4200.1-KR105217-SLE-Kenema|2014-07-08</td></tr>
<tr><td>Z_5</td><td>48%</td><td>0%</td><td>—</td></tr>
</table>

Supplementary Table 3: Main outliers in Supplementary Figure 20A result from errant misplaced tips. In each of these three biggest clusters, every clade consists of a large common core with 1–3 additional clade-specific tips. Different supports in runs A and B result from misplaced tips that MCMC appears unable to reposition, e.g., the `Conarky` tip in cluster Y is almost never attached to the core of cluster Y in run A, but almost always is in run B.

we break down the three largest such clusters, labeled X, Y, and Z. In panel A, we've highlighted the clades inside these three clusters (e.g., the largest cluster, X, consists of clades $X_1, \ldots, X_4$). The simplest explanation for this clustering is that a small number of "errant" tips (typically 1–2) are misplaced into a clade in one run but not the other, and the MCMC moves appear unable to reposition the errant tips in the appropriate location on the tree. This explanation is compatible with the detailed studies in [10], and suggest that more sophisticated MCMC moves may be needed to better explore the fine detail of this dataset's phylogeny.

We emphasize that the sampling difficulties discussed in this section reflect an intrinsic difficulty in exploring the nooks and cranies of tree space, but that higher-level observables seem to be insensitive to these difficulties (a similar observation was made [10]). In particular, the mean tMRCAs of all clades are essentially insensitive to these difficulties in every case we have examined, as are the distributions of global observables such as the mutation rates and population curves. So is the frechetCorrelationESS measure of [19], which is specifically designed to detect topological convergence slowdowns. At a practical level, we would recommend that for one to reliably draw any conclusion from a Delphy run that depends on the exact placement of a handful of tips, the conclusion should be reproduced in several independent runs.

15 Well-Calibrated Simulation Studies (WCSSs)

Delphy contains large amounts of new code implementing existing and new ideas; it does not have the years of battle testing that more established tools like BEAST benefit from. To increase the confidence in the correctness of our implementation, we pursued a series of Well-Calibrated Simulation Studies (WCSSs), as described in [22]. Briefly, a WCSS is a sensitive diagnostic of whether an MCMC implementation correctly samples the posterior that it claims to sample. When all tests of a WCSS pass, one obtains strong evidence (though not proof) that the MCMC implementation is correct; conversely, failing WCSS tests do provide strong evidence of an implementation error.

15.1 Overview

Here, we briefly summarize the main idea behind a WCSS, but refer the reader to [22] for full details. A WCSS for a generic Bayesian inference code involving "Model" and "Data" starts with the observation that it is generally straightforward to implement an indepedent sampler that correctly draws a "true" sample from a joint distribution consisting of known (proper) priors for the "Model" and a likelihood for the "Data" given the "Model". If one then masks the "Model" and uses MCMC to infer a posterior distribution of the "Model" given the "Data", then the "true" sample should be *statistically indistinguishable* from a sample drawn from the posterior distribution. This is a direct consequence of Bayes' theorem, as expressed by the following equalities:

$$\overbrace{\underbrace{P(\text{Data}|\text{Model})}_{\text{Likelihood}}\,\underbrace{P(\text{Model})}_{\text{Prior}}}^{\text{Sampling Time}} = \underbrace{P(\text{Model},\text{Data})}_{\text{Joint}} = \overbrace{\underbrace{P(\text{Model}|\text{Data})}_{\text{Posterior}}\,\underbrace{P(\text{Data})}_{\text{Evidence}}}^{\text{Inference Time}}.$$

The particular WCSS scheme described in [22] involves performing N *replicates* of the process of first drawing a "true" sample, then executing an inference run on that sample's Data to obtain a set of associated posterior samples. These N replicates' results are subjected to three kinds of tests of whether the true sample appear to be drawn from the associated posterior distribution:

1. *Coverage Validation*: Pick out any specific region of the posterior distribution accounting for a fraction x of its probability mass. If the MCMC implementation is correct, then the true sample should be in this target region with probability x. In particular, we can test whether the true sample is in the posterior 95% HPD in about 95% of the replicates, with binomial statistics for the actual number of replicates covered by the 95% HPD.

2. *Rank Uniformity Validation (RUV)*: When projecting the true and posterior samples along any one-dimensional coordinate x, the rank of the true sample's x-value along the sorted list of posterior x-values should be uniformly distributed. This is most easily understood by thinking of drawing the true sample's x using inverse-transform sampling on the eCDF implied by the posterior samples. Uniformity can be visualized in two complementary ways: (a) a "rank histogram", showing the rank of the true sample in each replicate; and (b) an "rank eCDF", showing the proportion of replicates for which the true sample's rank is below a fraction p. If the MCMC implementation is correct, then up to statistical noise, the rank histogram should be uniform and the rank eCDF should trace out a diagonal line from $(0, 0)$ to $(1, 1)$. In both cases, the expected size of the statistical noise follows simple binomial distributions. With K bins, the counts in each rank histogram bin should follow a $\text{Bin}(N, N/K)$ distribution. Similarly, the number of replicates where the rank is below p should follow a $\text{Bin}(N, p)$ distribution, so the rank eCDF at p should be in the interval $p \pm 1.96\sqrt{p(1-p)/N}$ about 95% of the time.

3. *Clade Coverage Validation*: After calculating the posterior supports of every clade observed in the posterior samples, we expect a clade with posterior support x to appear in the true sample with probability x. Since clades in a posterior sample are not independent, it is not straightforward to

estimate the statistics for a finite number of replicates. Instead, we settle for a simpler visual diagnostic: a histogram of clade posterior support vs. fraction of true samples with that clade, aggregated over all clades and replicates. If the MCMC implementation is correct, this histogram should roughly trace out a "triangle" in the lower-right portion of the plot; hence the informal name "triangle graph".

15.2 Sampling

For these studies, we extended Sapling, the custom sampler developed for the large-scale simulations in Fig. 6 of the main text, to support sampling the most complex models and datasets that Delphy can handle[3]. Most model variations are straightforward to sample from, so we omit the details. Only one nontrivial model variantion is described below in detail: how to efficiently sample mutation histories in the presence of site-rate heterogeneity and a general evolution model.

In common with other phylogenetic simulators, Sapling is structured in two phases. In Phase 1, it selects tip times (either exact times specified by a user, or by sampling in proportion to the population curve over a finite time interval) and runs a standard coalescent simulation under a user-specified population model to obtain a tree topology. Then, in Phase 2, it uses a user-specified evolution model to generate a random root sequence, and uses a Gillespie algorithm to sample mutations to that sequence along the branches of the tree topology (similarly to phastSim [7]). Specifically, when focused at a point x on the tree, Sapling first calculates the overall mutation rate there as $\lambda(x) = \sum_{\ell=1}^{L} Q^{(\ell)}_{s_x^{(\ell)}}$, then samples a waiting time to the next mutation time τ from an exponential distribution with rate $\lambda(x)$. If the remaining time on this branch is longer than τ, Sapling advances x by a time τ, inserts a mutation there, updates λ, then restarts the algorithm just after x; it also recurses into child branches to execute an in-order traversal of the whole tree topology. The main difficulty in this process is in choosing at what site to place each mutation: absent rate heterogeneity, this is simply a uniform integer between 1 and L, but in the general case, site ℓ must be picked with probability $p^{(\ell)} := Q^{(\ell)}_{s_x^{(\ell)}}/\lambda(x)$. Doing so by calculating cumulative sums of $p^{(\ell)}$ at every mutation is prohibitively expensive, and phastSim's solution to this problem, genome search trees, are nontrivial to implement. Instead, Sapling implements the efficient algorithm described below

If the site probabilities $p^{(\ell)}$ had no sequence dependence, and were thus everywhere the same, we could simply preprocess them to use the alias method [37, 38], which yields a sample ℓ from this fixed discrete distribution in $\mathcal{O}(1)$ time using at most two PRNG invocations. Since $p^{(\ell)} = Q^{(\ell)}_{s_x^{(\ell)}}/\lambda(x) = \mu\nu^{(\ell)} q_{s_x^{(\ell)}}/\lambda(x)$ (see Equation (17)), the major source of variation is typically $\nu^{(\ell)}$ when site-rate heterogeneity is enabled; by contrast, the relative escape rates q_a are close to 1 for typical evolution models. Given these observations, we combine the alias method with thinning to obtain an efficient sampler. Concretely, let $q_{\max} := \max_a q_a$ be the maximum escape rate. We apply the Gillespie algorithm with a maximum mutation rate $\lambda_{\max} := \mu \cdot (\sum_{\ell'} \nu^{(\ell')}) \cdot q_{\max}$. When we reach the stage of inserting a mutation, we pick a site ℓ with probability $\nu^{(\ell)}/\sum_{\ell'} \nu^{(\ell')}$ using the alias method and its associated preprocessed tables. Suppose the state at site ℓ is a. Then we draw a uniform number between 0 and $q_{\max}$; if it is above q_a, we *reject* this mutation proposal and restart the Gillespie loop at this point: this is the *thinning* step. If we instead accept the mutation proposal, we pick a target state $b \neq a$ with probability q_{ab}/q_a, as usual. In this way, the rate at which we insert mutations at site ℓ from a to b is:

$$\left\{\mu \cdot \left(\sum_{\ell'} \nu^{(\ell')}\right) \cdot q_{\max}\right\} \cdot \left\{\nu^{(\ell)} / \sum_{\ell'} \nu^{(\ell')}\right\} \cdot \{q_a/q_{\max}\} \cdot \{q_{ab}/q_a\} = \mu\nu^{(\ell)} q_{ab} = Q^{(\ell)}_{ab},$$

as required.

[3] In these WCSSs, we do not explore the APOBEC-specific evolution model for mpox used in Fig. 4 of the main text, so we have not added support for it in Sapling.

15.3 Results

We carried out three comprehensive WCSSs (A, B, and C), to demonstrate Delphy's correctness in various configurations, and fourth one (D) that demonstrates the effect of shortcomings in our existing moveset. These studies, along with 12 other preliminary ones used to build up the necessary data pipelines and sampling flexibility, are available in the data accompanying this paper under the `wcss` folder.

All studies here have:

- $N = 200$ replicates.
- 500 tips with dates sampled uniformly over the year 2025.
- A simulated genome size of $L = 30,000$ bases.
- An HKY evolution model with $\{\pi_a\} \sim \text{Dir}(1,\ 1,\ 1,\ 1)$ and $\log \kappa \sim \mathcal{N}(1,\ 1.25^2)$.

Runs were long enough that most achieved an ESS above 200 for all observables after discarding the first 30 % of the run as burn-in. In all studies, we masked out any runs where the ESS of any observable was below 150 (see discussion below); we report the fraction of such discarded runs in each study. All runs were executed on Hetzner `cpx62` instances (cloud-based compute servers with 16 vCPUs and 32 GiB of RAM each) using either Delphy 1.3.1 or Delphy 1.3.2. All ESSs, means and 95 % HPDs were calculated using BEAST 2's `loganalyser` tool. Clade coverage was calculated with a small, custom program `calc_clade_coverage` (available in the data repo) that uses clade fingerprints, as described in the previous section.

Study A (`wcss/21_final_high_mutation`) is the simplest WCSS in this section (Figure 21). Here, we have $\mu \sim \text{Gamma}(1,\ 1 \times 10^{-3}$ mutations / site / year) (i.e., an exponential distribution), a constant population (i.e., fixed $g = 0$) with fixed size $n_0 = 1$ year, no site-rate heterogeneity, no missing data and no tip-date uncertainty. The replicates in this study deliberately have a wide distribution in their total number of mutations, from almost 0 to 5000–7000. Hence, while many replicates resemble the densely sampled "genomic epidemiology" datasets that Delphy is targeted to (i.e., with about 1-2 mutations per tree branch, totalling 1000–2000 mutations), many also have long branches with large numbers of mutations (particularly in the long branches near the root typical of constant-population coalescent simulations). We filtered out 9 of the 200 replicates (4.5%) for having ESS below 150 in at least one observable.

Figure 21A shows that all inferred observables have the expected 95% HPD coverage, while Figure 21B shows that clade coverages also behave as expected. The detailed rank histogram, rank eCDFs and true vs. inferred value scatter plots in Figure 21C have the expected behaviour for a WCSS applied to a correct MCMC implementation. Hence, Figure 21 provides strong evidence both that the core MCMC mechanics of Delphy are correctly implemented, and that Delphy degrades in speed but not correctness when it is applied to datasets that are far from our target of densely sampled "genomic epidemiology" datasets.

Study B (`wcss/22c_skygrid_no_year_uncertainty`) is the most comprehensive of our WCSSs, designed to exercise almost all of Delphy's features at once while focusing on Delphy's preferred "densely sampled" regime to favor quick convergence (Figure 22). Each replicate uses a mutation rate is drawn from a Gamma distribution with mean 10^{-3} and standard deviation 10^{-4} mutations/site/year. They use a Skygrid population model with 5 knots evenly spaced between 2025-01-01 and 2026-01-01, using log-linear interpolation. The mean population size $\bar{N}$ is drawn from an Inverse-Gamma distribution with a mean of 0.25 and a standard deviation of 0.05 years, and with a GMRF precision τ drawn from a Gamma distribution with mean 10^{-3} and standard deviation 10^{-4}. The actual log-populations $\{\gamma_k\}$ are sampled by executing a random walk with the chosen precision, then shifting them all by an appropiate offset to obtain the sampled mean population size $\bar{N}$. For this study, the Skygrid low-population barrier is disabled, and we have chosen $\bar{N}$ and τ to make it highly improbable that the population ever drops below 1 day. Site-rate heterogeneity is enabled, with site relative rates $\{\nu^{(\ell)}\}$ drawn from a $\text{Gamma}(\alpha,\ \alpha)$ distribution, with $\alpha \sim \text{Expo}(1.0)$. A random ~ 5 % of the bases in each sequence is masked as missing data as follows: a random number of gaps ($\sim$ Pois(3.0)) with random lengths ($\sim$ Pois(500.0)) are introduced uniformly throughout the genome (allowing for overlaps), and a similar random number of isolated, uniformly distributed individual sites (also $\sim$ Pois(3.0)) is also marked as missing. Finally, for a random 15 % of tips, the tip date is made uncertain

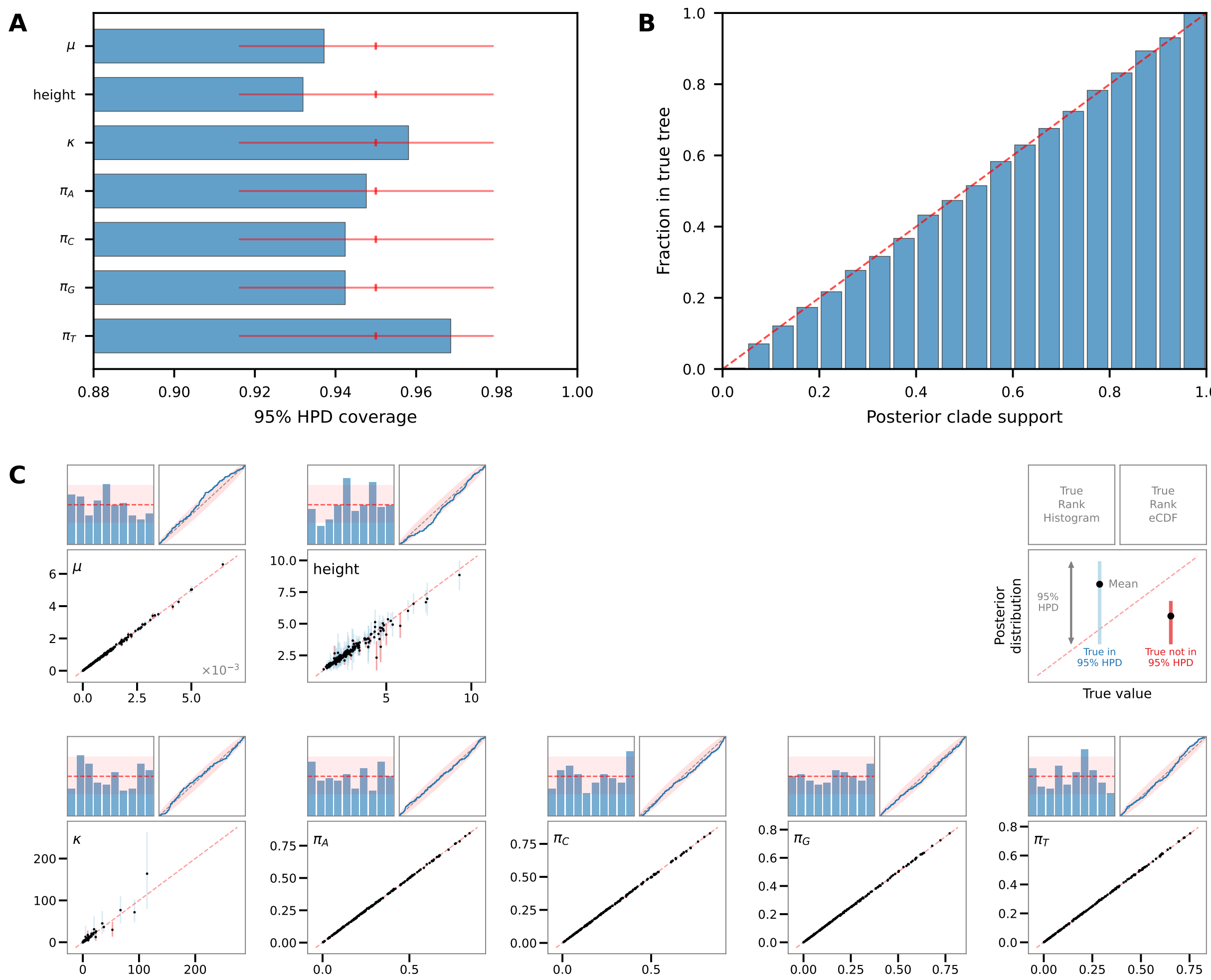

Supplementary Figure 21: WCSS Study A. Simple high-mutation WCSS: mutation rate $\mu \sim \text{Expo}(10^{-3}\,\text{mutations/site/year})$, constant effective population size $n_0 = 1.0\,\text{year}$, no site-rate heterogeneity, no missing data, no tip-date sampling. **A.** Coverage Validation: for each inferred observable, the fraction of replicates whose true value is covered by the 95% HPD of the inferred posterior distribution (red bars = 95% CI under correct sampling). **B.** Clade Coverage: each bin groups all clades across all posterior samples and replicates having the indicated posterior support, and bin heights are the fraction of respective true trees containing that clade (dashed diagonal = expected behaviour under correct sampling). **C.** Rank Uniformity Validation: for each observable, rank histogram shows the counts of replicates which the given true sample's rank among the associated posterior samples (red shading: per-bin 95% CI under correct sampling; x axis ranges from 0 to 1 and y axis starts at 0, with red dashed line at N/K with $K = 10$ bins); rank eCDF shows proportion of replicates whose true sample's rank among associated posterior samples is below p (red shading: 95% CI under correct sampling; both axes range from 0 to 1); scatter plot shows true value (x) vs inferred posterior distribution (y) for each replicate (dot = mean, bar = 95% HPD, blue bar = true value covered by 95% HPD, red bar = true value not covered).

up to the date's month (e.g., a tip date of 2025-07-12 is masked to simply 2025-07). All these priors and conditions are inspired by the general properties of genomic epidemiology studies datasets known to us at the time of writing. After inference, we filtered out 21 of the 200 replicates (10.5%) for having ESS below 150 in at least one observable.

The results in Figure 22 provide strong evidence that all of Delphy's key features are backed by a correct MCMC implementation. In particular, for the parameter and data configurations represented in the study, the inference clearly recovers the mutation rate, the tree height, all evolution model parameters and the underlying population curve parameters. For tip dates with month-long uncertainties, the data is mostly but not completely uninformative of the precise day. However, the 95 % HPDs correctly cover $\sim 95\,\%$ of the true dates (note that since tip dates within a single replicate are not fully independent, the simple binomial CIs for this coverage are more a guide to the eye than a strict expectation). We also note that the Skygrid precision τ is almost unconstrained by the data (correct coverage follows entirely from the prior distribution), which we present as evidence to justify Delphy's choice of using a fixed τ by default, instead of inferring τ as in [11]. Note also that the data is only weakly informative of the site-rate heterogeneity scale α, which agrees with the intuition that since trees in the "dense sampling" region are almost parsimonious, their mutation structure is mostly fixed by the data, and at the tree scales considered in this study, repeated mutations at the same site are rare enough that evidence for site rate heterogeneity is generally weak. Thus, we suggest that not much is lost by running Delphy with site-rate heterogeneity disabled.

Study C (`wcss/23b_skygrid_no_year_uncertainty`) complements Study B by modeling population curves as pure exponentials (Figure 23). It is identical to Study B except for the population model: here, we draw a final population size n_0 (at 2026-01-01) from an Inverse-Gamma distribution with mean 2.5 and standard deviation 0.5 years, and a growth rate g from a Laplace distribution with mean 2.0 and scale 0.2 years before truncation through rejection sampling at $g_{\min} = 0.5\,/\text{year}$. Site-rate heterogeneity, missing data and tip-date uncertainty were as above. After inference, we filtered out 18 of the 200 replicates (9.0%) for having ESS below 150 in at least one observable.

Figure 23 provides further strong evidence that our MCMC implementation is also correct for exponentially growing populations. We also note that, as in Study B, in the parameter and data configurations represented in the study, the inference clearly recovers the final population size and overall growth rate.

Finally, Study D (`wcss/22b_final_skygrid_lower_n0`) presents a varation of Study B where 5% of tip dates have all but their year masked (e.g., a tip date of 2025-07-12 is masked to simply 2025), and presents a cautionary tale of how sampling difficulties with errant misplaced tips manifest in a WCSS. Figure 24 shows that the 95% HPD coverage of the root height and of the site-rate heterogeneity scale α are far outside the expectations for a correct MCMC implementation. On closer inspection, the scatter plot for the inferred tip dates of tips with year-long uncertainties shows that in a handful of replicates, one or more tips are significantly misplaced: their true dates are near the end of the year 2025, but they are confidently placed far before their true date, most of them right at the beginning of 2025 (note, however, that most such tip dates are correctly inferred, and that the data is strongly informative for them). We've verified that it is predominantly in replicates with such misplaced tips that the inferred root height is overestimated and so the 95 % HPD fails to cover the true value. We attribute this failure to a shortcoming in our moveset: we have one move to Gibbs sample a tip date given fixed tree topology and mutational history, and our workhorse mdSPR move for changing tree topology given, among other things, fixed tip dates. For a tip that is misplaced near the beginning of its date range to then be placed elsewhere on the tree at a much later date, the MCMC has to traverse an unlikely intermediate state where the tip is still misplaced but the tip date is pushed farther into the future than the prospective better attachment point, which involves stretching the original tip's branch far beyond what the mutational history on it implies is likely. One potential solution to this problem would be to extend our mdSPR to simultaneously change the attachment point and the tip date of tips with very uncertain dates; we leave such an extension for future work. Finally, we note that this WCSS has one more hint that there are serious convergence problems: after inference, we had to filter out 39 of the 200 replicates (19.5%) for having ESS below 150 in at least one observable. Presumably, many of the filtered runs exhibit similar convergence problems in their year-uncertain tip dates, but the runs exhibit enough variability to ascertain that they have a low ESS; it is only the runs where the tip dates are

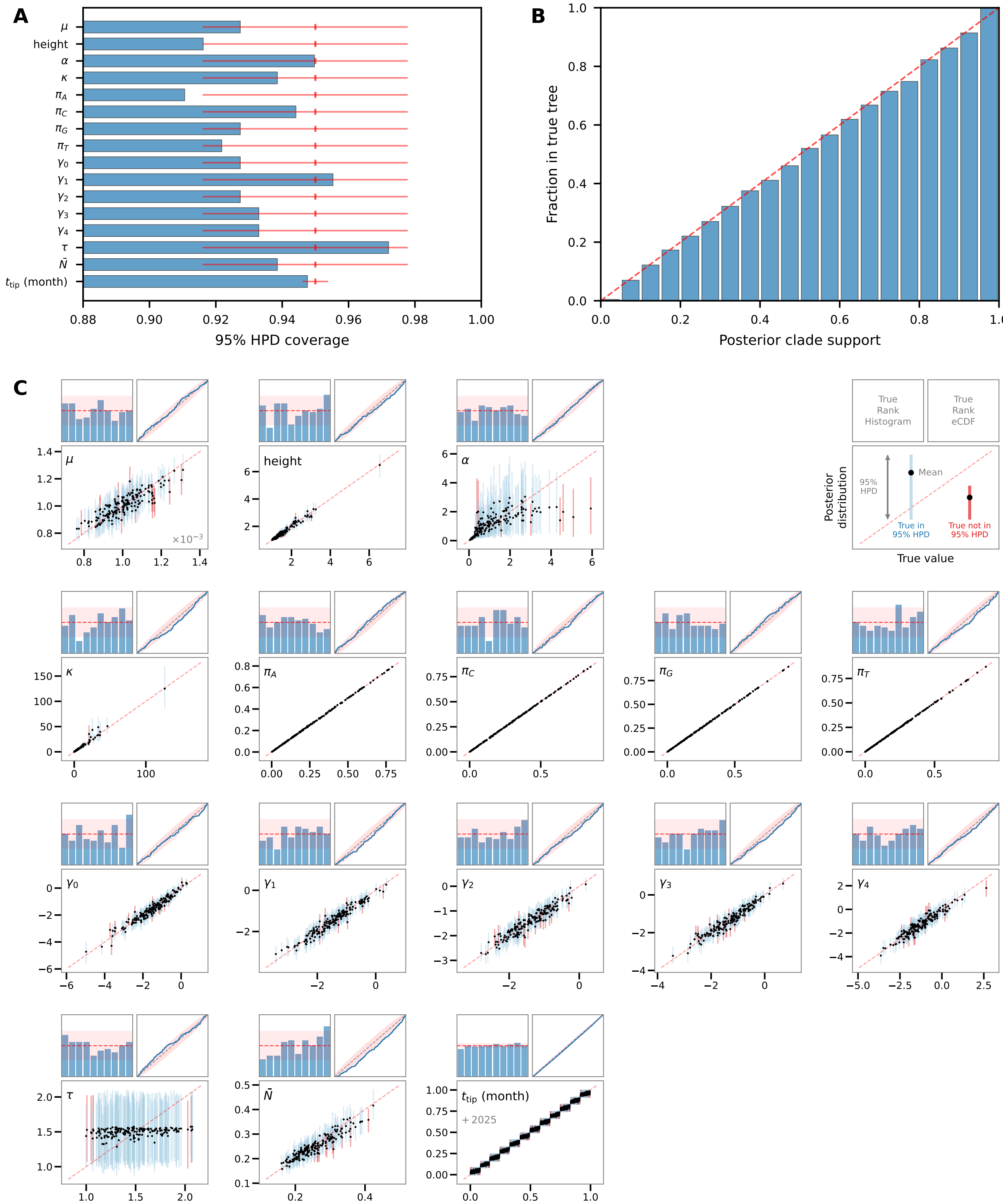

Supplementary Figure 22: WCSS Study B. Comprehensive Skygrid WCSS: mutation rate $\mu \sim \text{Gamma}(\mu = 10^{-3},\ \sigma = 10^{-4}\,\text{mutations/site/year})$; Skygrid population model with 5 knots evenly spaced from 2025-01-01 to 2026-01-01, mean population $\bar{N} \sim \text{Inv-Gamma}(\mu = 0.25,\ \sigma = 0.05\,\text{years})$ and precision $\tau \sim \text{Gamma}(\mu = 10^{-3},\ \sigma = 10^{-4})$; site-rate heterogeneity with $\alpha \sim \text{Expo}(1.0)$; $\sim 5\%$ of bases marked as missing; and $\sim 15\%$ of tips having month-uncertain dates. See Figure 21 for panel descriptions

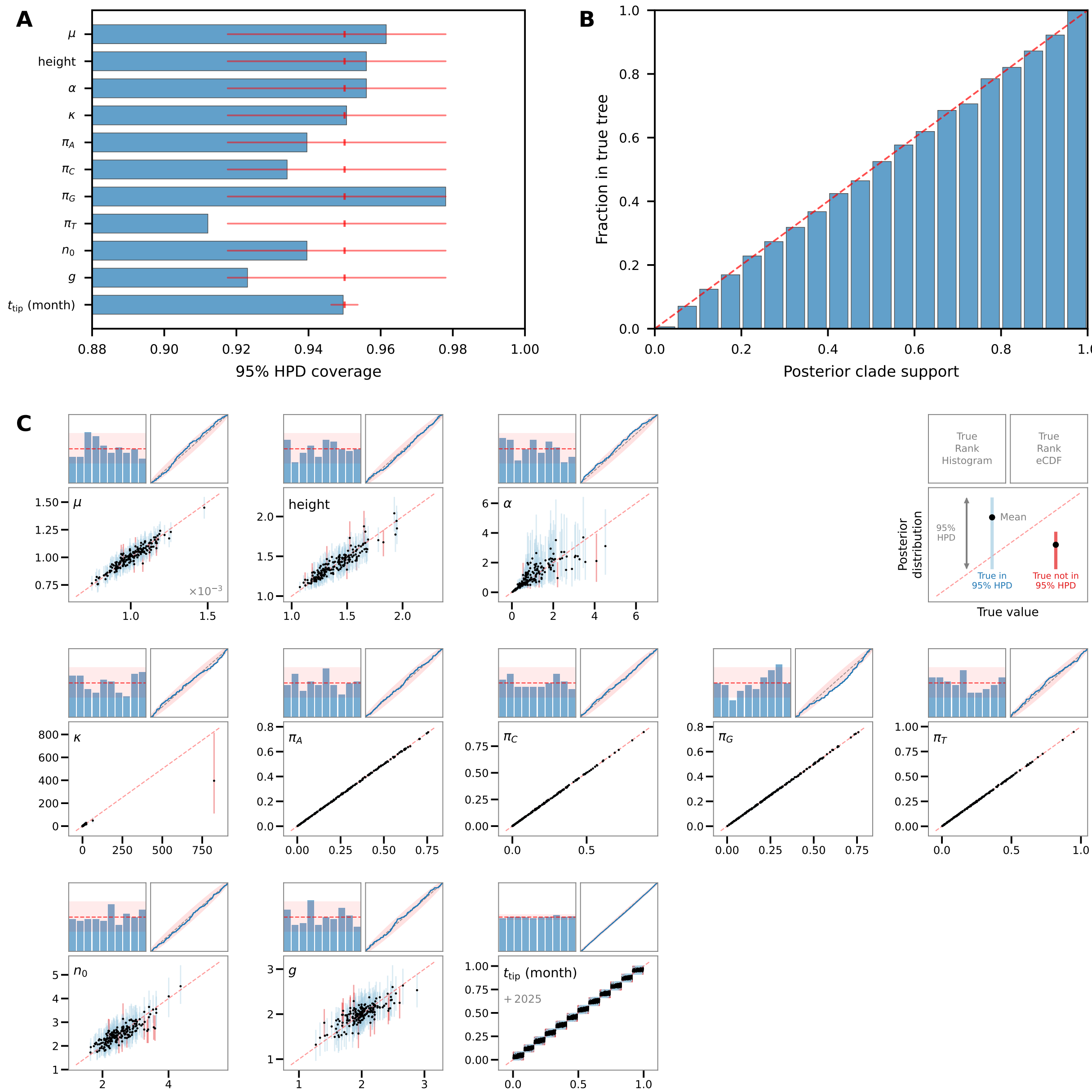

Supplementary Figure 23: WCSS Study C. Comprehensive exponential growth WCSS: parameters as in B (Fig 22), except the population model is exponential, with final population size $n_0 \sim \text{Inv-Gamma}(\mu = 2.5,\ \sigma = 0.5\,\text{years})$ and growth rate $g \sim \text{Laplace}(\mu = 2.0,\ s = 0.2\,\text{year}^{-1} | g > 0.5\,\text{year}^{-1})$. See Figure 21 for panel descriptions

confidently misplaced over the length of the run that have incorrectly high ESS estimates, and thus show up in the final WCSS figure.

Taken together, the studies in this section both provide strong evidence that our MCMC implementation is correct (*A*-*C*), but also hint that there are scenarios where sampling difficulties can cause problems (*D*). Taken together with the results in the main text, these studies justify the confident use of Delphy in scenarios typical of genomic epidemiology, but also motivate further development of more advanced MCMC moves that overcome sampling difficulties. At this time, we do not recommend using Delphy for datasets with many tips having year-long uncertainties, and as with the errant tips problem described in Section 14, we strongly recommend the standard practice of performing several independent MCMC runs to verify that no important scientific conclusion results from a misplaced tip in a single, incompletely converged run.

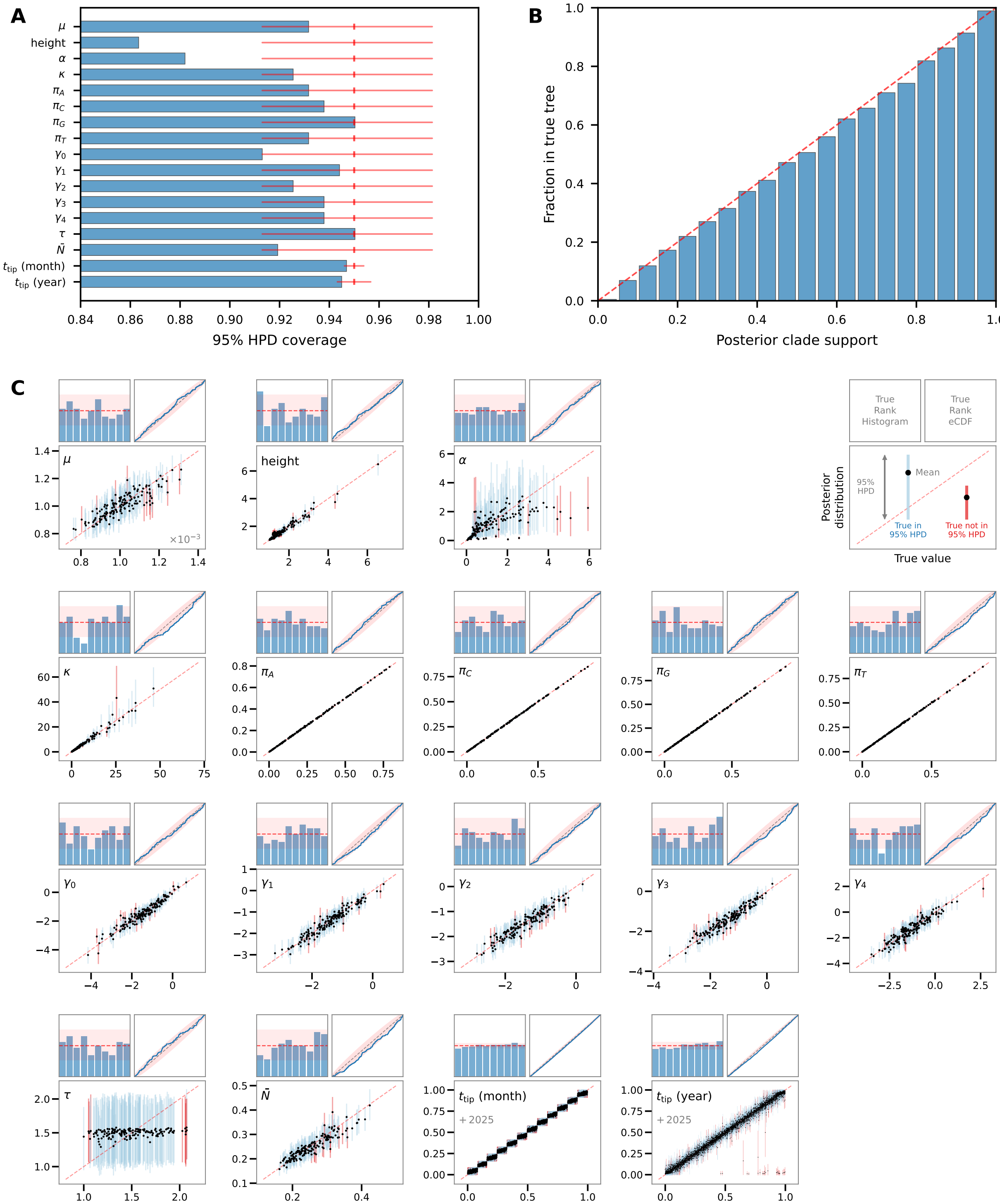

Supplementary Figure 24: WCSS Study D. Comprehensive Skygrid WCSS with 5 % year-long tip date uncertainties: the misplacement of a handful of such tips with year-long date uncertainties (lower-right corner of "t_{tip} (year)" panel) leads to overestimates of the root height, and thus to 95 % HPD coverage inconsistent with correct sampling. See Figure 21 for panel descriptions

References

[1] Guy Baele et al. “Hamiltonian Monte Carlo sampling to estimate past population dynamics using the skygrid coalescent model in a Bayesian phylogenetics framework”. In: *Wellcome Open Research* 5.53 (2020). DOI: 10.12688/wellcomeopenres.15770.1.

[2] Guy Baele et al. “HIPSTR: highest independent posterior subtree reconstruction in TreeAnnotator X”. In: *Bioinformatics* 41.10 (2025), btaf488. DOI: 10.1093/bioinformatics/btaf488.

[3] Lars Berling et al. “Accurate Bayesian phylogenetic point estimation using a tree distribution parameterized by clade probabilities”. In: *PLoS Comput Biol* 21.2 (2025), e1012789. DOI: 10.1371/journal.pcbi.1012789.

[4] R. Borndörfer, Z. Elijazyfer, and S. Schwartz. “Approximating Balanced Graph Partitions”. In: *Zuse Institute Berlin Report* 19.25 (2019).

[5] Nawaf Bou-Rabee and Jesús María Sanz-Serna. “Randomized Hamiltonian Monte Carlo”. In: *The Annals of Applied Probability* 27.4 (Aug. 2017), pp. 2159–2194. DOI: 10.1214/16-AAP1255.

[6] John W Byers et al. “Informed content delivery across adaptive overlay networks”. In: *IEEE/ACM transactions on networking* 12.5 (2004), pp. 767–780.

[7] Nicola De Maio et al. “phastSim: Efficient simulation of sequence evolution for pandemic-scale datasets”. In: *PLoS Computational Biology* 18 (4 2022), e1010056. DOI: 10.1371/journal.pcbi.1010056.

[8] Alexei J. Drummond et al. “Estimating Mutation Parameters, Population History and Genealogy Simultaneously From Temporally Spaced Sequence Data”. In: *Genetics* 161 (2002), pp. 1307–1320.

[9] Joseph Felsenstein. “Evolutionary trees from DNA sequences: A maximum likelihood approach”. In: *Journal of Molecular Evolution* 17.6 (1981), pp. 368–376. DOI: https://doi.org/10.1007/BF01734359.

[10] Jiansi Gao et al. “Biological causes and impacts of rugged tree landscapes in phylodynamic inference”. In: *PNAS* 123 (2 2026), e2510938123. DOI: 10.1073/pnas.2510938123.

[11] Mandev S. Gill et al. “Improving Bayesian Population Dynamics Inference: A Coalescent-Based Model for Multiple Loci”. In: *Molecular Biology and Evolution* 30.3 (Nov. 2012), pp. 713–724. DOI: 10.1093/molbev/mss265.

[12] W.K. Hastings. “Monte Carlo sampling methods using Markov chains and their applications”. In: *Biometrika* 57 (1970), pp. 97–109.

[13] William Howard-Snyder et al. “Densely sampled phylogenies frequently deviate from maximum parsimony in simple and local ways”. In: *arXiv* (), 2311.10913 [q–bio.PE]. DOI: https://doi.org/10.48550/arXiv.2311.10913.

[14] J.F.C. Kingman. “On the Genealogy of Large Populations”. In: *Journal of Applied Probability* 19 (1982), pp. 27–43.

[15] J.F.C. Kingman. “The Coalescent”. In: *Stochastic Processes and the Applications* 13 (1982), pp. 235–248.

[16] Alexander M. Kramer et al. “Online phylogenetics with matOptimize produces equivalent trees and is dramatically more efficient for large SARS-CoV-2 phylogenies than de novo and maximum-likelihood implementations”. In: *Systematic Biology* 72 (2023), pp. 1039–1051.

[17] Nicolas Lartillot. “Conjugate Gibbs sampling for Bayesian phylogenetic models”. In: *Journal of Computational Biology* 13.10 (2006), pp. 1701–1722. DOI: https://doi.org/10.1089/cmb.2006.13.1701.

[18] Jacob E. Lemieux et al. “Phylogenetic analysis of SARS-CoV-2 in Boston highlights the impact of superspreading events”. In: *Science* 371 (2021), eabe3261. DOI: https://doi.org/10.1126/science.abe3261.

[19] Andrew Magee et al. "How Trustworthy Is Your Tree? Bayesian Phylogenetic Effective Sample Size Through the Lens of Monte Carlo Error". In: *Bayesian Analytics* 19.2 (2024), pp. 565–593. DOI: 10.1214/22-ba1339.

[20] Nicola De Maio et al. "Maximum likelihood pandemic-scale phylogenetics". In: *Nature Genetics* 55 (2023), pp. 746–752. DOI: https://doi.org/10.1038/s41588-023-01368-0.

[21] Nicola De Maio et al. "Rate variation and recurrent sequence errors in pandemic-scale phylogenetics". In: *bioRxiv* (2024). DOI: https://doi.org/10.1101/2024.07.12.603240.

[22] Fábio K. Mendes et al. "How to Validate a Bayesian Evolutionary Model". In: *Systematic Biology* 74 (1 2025), pp. 158–175. DOI: 10.1093/sysbio/syae064.

[23] Ralph C Merkle. "A digital signature based on a conventional encryption function". In: *Conference on the theory and application of cryptographic techniques*. Springer. 1987, pp. 369–378.

[24] Ralph C Merkle. *Method of providing digital signatures*. US Patent 4,309,569. 1982.

[25] Nicholas Metropolis et al. "Equations of state calculations byfast computing machines". In: *Journal of Chemical Physics* 21 (1953), pp. 1087–1091.

[26] Vladimir N. Minin, Erik W. Bloomquist, and Mark A. Suchard. "Smooth skyride through a rough skyline: Bayesian coalescent-based inference of population dynamics". In: *Molecular Biology and Evolution* 25 (), pp. 1459–1471. DOI: https://doi.org/10.1093/molbev/msn090.

[27] Rasmus Nielsen. "Mapping Mutations on Phylogenies". In: *Systematic Biology* 51.5 (2002), pp. 729–739. DOI: https://doi.org/10.1080/10635150290102393.

[28] Rasmus Nielsen. "Mutations as Missing Data: Inferences on the Ages and Distributions of Nonsynonymous and Synonymous Mutations". In: *Genetics* 159.1 (2001), pp. 401–411. DOI: https://doi.org/10.1093/genetics/159.1.401.

[29] Áine O'Toole et al. "APOBEC3 deaminase editing in mpox virus as evidence for sustained human transmission since at least 2016". In: *Science* 382 (2023), pp. 595–600. DOI: https://doi.org/10.1126/science.adg8116.

[30] Edyth Parker et al. "Genomics reveals zoonotic and sustained human mpox spread in West Africa". In: *Nature* 643 (2025), pp. 1343–1351. DOI: https://doi.org/10.1038/s41586-025-09128-2.

[31] Louis du Plessis et al. "Establishment and lineage dynamics of the SARS-CoV-2 epidemic in the UK". In: *Science* 371 (2021), pp. 708–712.

[32] D. F. Robinson and L. R. Foulds. "Comparison of phylogenetic trees". In: *Mathematical Biosciences* 53.1 (1981), pp. 131–147. DOI: 10.1016/0025-5564(81)90043-2.

[33] Ivan Specht, Patrick Varilly, and Pardis C. Sabeti. "JUNIPER: Reconstructing Transmission Events from Next-Generation Sequencing Data at Pandemic Scale". In: *(in preparation)* (2024).

[34] Tanja Stadler. "Sampling-through-time in birth-death-trees". In: *Journal of Theoretical Biology* 267 (3 2010), pp. 396–404. DOI: 10.1016/j.jtbi.2010.09.010.

[35] M. Tuckerman, Bruce J. Berne, and G. J. Martyna. "Reversible multiple time scale molecular dynamics". In: *The Journal of Chemical Physics* 97.3 (Aug. 1992), pp. 1990–2001. DOI: 10.1063/1.463137.

[36] Yatish Turakhia et al. "Ultrafast Sample placement on Existing tRees (UShER) enables real-time phylogenetics for the SARS-CoV-2 pandemic". In: *Nature Genetics* 53.6 (2021), pp. 809–816. DOI: https://doi.org/10.1038/s41588-021-00862-7.

[37] Michael D. Vose. "A Linear Algorithm for Generating Random Numbers with a Given Distribution". In: *IEEE Transactions on Software Engineering* 17 (9 1991), pp. 972–975. DOI: doi.org/10.1109/32.92917.

[38] Alistair J. Walker. "An Efficient Method for Generating Discrete Random Variables with General Distributions". In: *ACM Transactions on Mathematical Software* 3 (3 1977), pp. 253–256. DOI: 10.1145/355744.355749.

[39] Ian J. Wilson and David J. Balding. "Genealogical Inference From Microsatellite Data". In: *Genetics* 150 (1998), pp. 499–510.

[40] Ziheng Yang. "Estimating patterns of nucleotide substitution". In: *Molecular Evolution* 39 (1994), pp. 105–111.

[41] Ziheng Yang. "Maximum Likelihood Phylogenetic Estimation from DNA Sequences with Variable Rates over Sites: Approximate Methods". In: *Journal of Molecular Evolution* 39 (1994), pp. 306–314.

[42] Cheng Ye et al. "matOptimize: a parallel tree optimization method enables online phylogenetics for SARS-CoV-2". In: *Bioinformatics* 38.15 (2022), pp. 3734–3740. DOI: `https://doi.org/10.1093/bioinformatics/btac401`.

[43] Chi Zhang, John P. Huelsenbeck, and Fredrik Ronquist. "Using Parsimony-Guided Tree Proposals to Accelerate Convergence in Bayesian Phylogenetic Inference". In: *Systematic Biology* 69.5 (2020), pp. 1016–1032. DOI: `https://doi.org/10.1093/sysbio/syaa002`.

**Notation**	**Meaning**	**Page, (Equation)**
L	Number of sites on genome	13
ℓ	A site index ($1 \le \ell \le L$)	13
`A`, `C`, `G`, `T`	A specific state at some site (`T` = `U`)	13
a, b, c, …	A variable representing a state	13
$\mathcal{T}$	An explicit mutation-annontated tree (EMAT); also all the points on this tree	13
N	Number of tips, number of input sequences	13
i, j, k, …	The index of a node on a tree ($1 \le i \le N$ for tips; $N < i < 2N$ for inner nodes). Also the branch ending at node i	13
$\varphi(i)$	The ancestor node of node i	13
t_i	The time of node i; increases forwards from past to future	13
$\perp$	A conceptual point above the root node	13
$[[i, t]]$	A particular point on the tree, located at time t on branch i	14
x, y, z	A variable representing a tree position	14
x^-, x^+	The points immediately preceding (x^-) or succeeding (x^+) the point x.	14
τ_i	The length of branch i; $\tau_i = t_i - t_{\varphi(i)}$	14
B_i	The set of all points on branch i	14
$\mathcal{M}_i$	The set of mutations $\{(i, t, a, \ell, b)\}$ on branch i; a mutation marks a change at time t of the state at site ℓ from a to b	14
$\mathcal{M}$	The set of all mutations; $\mathcal{M} = \cup_i \mathcal{M}_i$	14
$\mathcal{M}^{(\ell)}$	The set of mutations anywhere occurring at site ℓ	18
$\mathcal{M}_i^{(\ell)}$	The set of mutations on branch i occurring at site ℓ	18
M	The total number of mutations	17, (9)
$s_x^{(\ell)}$	The state at site ℓ at position x on the tree. The sequence at x is $s_x^{(1)} s_x^{(2)} \cdots s_x^{(L)}$	14
Φ_i	The set of missations $\{(i, a, \ell)\}$ at the start of branch i; a missation denotes that at all points at the start of branch i, the state at site ℓ is a, but no tips below that site are informative at site ℓ	14
Φ	The set of all missations; $\Phi = \cup_i \Phi_i$.	14
$\xi(x)$	The set of sites for which at least one tip downstream of x is informative	14
$\int_{x \in B_i} f(x)\, dx$	An integral over all points x on branch i	14, (1)
$\int_{x \in T} f(x)\, dx$	An integral over all points x on tree $\mathcal{T}$	14, (2)

Supplementary Table 4: Summary of key notation for EMATs.

Notation	Meaning	Page, (Equation)
$\boldsymbol{\theta}$	A vector of global model parameters, such as mutation rate, population curve parameters, etc.	15
$P(\mathcal{T}, \boldsymbol{\theta})$	The overall posterior distribution sampled by Delphy	15, (3)
$\pi_{\boldsymbol{\theta}}(\boldsymbol{\theta})$	A parameter prior for $\boldsymbol{\theta}$	19, 23, (30)
$\pi_{\text{anc}}(\mathcal{T}\|\boldsymbol{\theta})$	An ancestry prior on the genealogy of $\mathcal{T}$	19, 48, (56)
$\mathcal{G}(\mathcal{T}\|\boldsymbol{\theta})$	A genetic prior on the exact root sequence and mutational history encoded in $\mathcal{T}$	16, (8)
$L(\mathcal{T})$	The likelihood of the data given the entire EMAT model $\mathcal{T}$	15
$Q^{(\ell)}_{ab}$	An element of the transition rate matrix at site ℓ; for $a \neq b$, the rate at which a site with state a mutates to state b; for $a = b$, negative of the escape rate at which a site with state a mutates to anything else	16
$P^{(\ell)}_{ab}(t)$	An element of the evolution matrix at site ℓ for a trajectory of length t; the probability that a trajectory for site ℓ of length t that starts with state a ends in state b	16, 24, (6), (32), (32)
$Q^{(\ell)}_{a}$	The escape rate at which a site with state a mutates to anything else	16, (4)
$\pi^{(\ell)}_{a}$	The stationary frequency of state a at site ℓ	16, (5)
$\lambda(x)$	The total mutation rate at position x on the tree	16, (7)
$\mathcal{L}_{\text{tree}}(\mathcal{T}\|\boldsymbol{\theta})$	The traditional tree likelihood of $\mathcal{T}$ (ignoring mutations on $\mathcal{T}$), as calculated by Felsenstein pruning	17, 26, (38)
$\mathcal{G}_i(\mathcal{T}\|\boldsymbol{\theta})$	The branch genetic prior for branch i, i.e., the portion of $\mathcal{G}(\mathcal{T}\|\boldsymbol{\theta})$ arising from branch i	17, (10), (11)
$\mathcal{G}^{(\ell)}(\mathcal{T}\|\boldsymbol{\theta})$	The site genetic prior for site ℓ, i.e., the portion of $\mathcal{G}(\mathcal{T}\|\boldsymbol{\theta})$ arising from site ℓ	18, (12)
$\lambda^{(\ell)}(x)$	The contribution to the total mutation rate $\lambda(x)$ at x arising from site ℓ	18
$\mathcal{G}^{(\ell)}_i(\mathcal{T}\|\boldsymbol{\theta})$	The site-branch genetic prior for site ℓ and branch i, i.e., the portion of $\mathcal{G}(\mathcal{T}\|\boldsymbol{\theta})$ arising from site ℓ and branch i	18, (13)
μ	The mean per-site mutation rate	19, (17)
$\nu^{(\ell)}$	The site-relative rate of site ℓ	19, (17)
q_{ab}	The reduced transition rate matrix elements	19, (18)
κ	The transition-transversion ratio in the HKY evolution model	19, (18)
α	The shape of the site-rate Gamma distribution	20, (21)
$N(t)$	The population curve in a coalescent ancestry prior	20, 21, (24), (27), (28)
$k(t)$	The number of active lineages at time t	48, (56)
n_0	Effective population size times generation time at the time of latest tip (exponentially growing population)	20, (24)
g	Population growth rate (exponentially growing population)	20, (24)
M	Number of intervals (Skygrid population model)	21
$x_0 < \ldots < x_M$	Knot times (Skygrid population model)	21, (25)
$\gamma_0, \ldots, \gamma_k$	Log-population values at corresponding knot times (Skygrid population model)	21, (26)
τ, D, T_2	Different parametrizations of the population curve a priori flexibility: precision τ, diffusion constant D, timescale T_2 for factor-of-2 change (Skygrid population model)	22, (29)

Supplementary Table 5: Summary of key notation for tree and parameter distributions.
