# Supplementary material for: Scalable near-real-time Bayesian phylogenetics for outbreaks with Delphy
Source: Nature. 2026 Sep 16;658(8136):747–56. doi: 10.1038/s41586-026-11012-6 (PMC13668698; doi:10.1038/s41586-026-11012-6)
Supplement: Supplementary file 2 [file 41586_2026_11012_MOESM2_ESM.pdf]

nature portfolio

Corresponding author(s): Patrick Varilly
Pardis C. Sabeti

Last updated by author(s): Jun 1, 2026

Reporting Summary

Nature Portfolio wishes to improve the reproducibility of the work that we publish. This form provides structure for consistency and transparency in reporting. For further information on Nature Portfolio policies, see our Editorial Policies and the Editorial Policy Checklist.

Statistics

For all statistical analyses, confirm that the following items are present in the figure legend, table legend, main text, or Methods section.

n/a	Confirmed	
☐	☒	The exact sample size (*n*) for each experimental group/condition, given as a discrete number and unit of measurement
☐	☒	A statement on whether measurements were taken from distinct samples or whether the same sample was measured repeatedly
☐	☒	The statistical test(s) used AND whether they are one- or two-sided<br>*Only common tests should be described solely by name; describe more complex techniques in the Methods section.*
☐	☒	A description of all covariates tested
☐	☒	A description of any assumptions or corrections, such as tests of normality and adjustment for multiple comparisons
☐	☒	A full description of the statistical parameters including central tendency (e.g. means) or other basic estimates (e.g. regression coefficient) AND variation (e.g. standard deviation) or associated estimates of uncertainty (e.g. confidence intervals)
☒	☐	For null hypothesis testing, the test statistic (e.g. *F*, *t*, *r*) with confidence intervals, effect sizes, degrees of freedom and *P* value noted<br>*Give P values as exact values whenever suitable.*
☐	☒	For Bayesian analysis, information on the choice of priors and Markov chain Monte Carlo settings
☒	☐	For hierarchical and complex designs, identification of the appropriate level for tests and full reporting of outcomes
☒	☐	Estimates of effect sizes (e.g. Cohen's *d*, Pearson's *r*), indicating how they were calculated

Our web collection on statistics for biologists contains articles on many of the points above.

Software and code

Policy information about availability of computer code

Data collection	Data was downloaded directly from previous papers' supplementary information where available, or from GenBank directly (using the Entrez module of the Python biopython package version 1.85), or from GISAID directly (manually). The data repository accompanying the paper, at https://github.com/broadinstitute/delphy-2026-paper-data, contains complete executable scripts that thus document how to download all raw data for all analyses and figures directly from the original sources.
Data analysis	The essential software was Delphy itself, version 1.1.4 (version 1.0 was used in the sars-cov-2-gisaid-week-by-week and sims benchmars). We also used MAFFT Version 7.505 (2022/Apr/10), BEAST2 v2.7.7, BEAST X 10.5.0, BEAGLE commit 6480ad3 (Mon Sep 50 2025), Sapling Version 0.1.1 (build 2, commit a0b9da1 - for sims benchmarks) and 0.2.0 (build 7, commit 5b5dcc7 - for WCSS studies) - this is custom software developed for this paper and described in the Methods), IQ-TREE 2.3.6 and TreeTime 0.11.4. All scripts used to go from raw data to paper figures are available at https://gtihub.com/broadinstitute/delphy-2026-paper-data

For manuscripts utilizing custom algorithms or software that are central to the research but not yet described in published literature, software must be made available to editors and reviewers. We strongly encourage code deposition in a community repository (e.g. GitHub). See the Nature Portfolio guidelines for submitting code & software for further information.

Data

Policy information about availability of data

All manuscripts must include a data availability statement. This statement should provide the following information, where applicable:

- Accession codes, unique identifiers, or web links for publicly available datasets
- A description of any restrictions on data availability
- For clinical datasets or third party data, please ensure that the statement adheres to our policy

All of our data and analysis scripts are publicly available at https://github.com/broadinstitute/delphy-2026-paper-data, and the scripts link directly to the original source data where available. For runs including GISAID data, which cannot be shared publicly, we provide instructions on how anyone with access to GISAID can download and format the raw data in a way that will allow all run and analysis scripts to proceed. Our version of that data is available upon request to users who have also agreed to the GISAID Data Access Agreement.

Research involving human participants, their data, or biological material

Policy information about studies with human participants or human data. See also policy information about sex, gender (identity/presentation), and sexual orientation and race, ethnicity and racism.

Reporting on sex and gender	N/A
Reporting on race, ethnicity, or other socially relevant groupings	N/A
Population characteristics	N/A
Recruitment	N/A
Ethics oversight	N/A

Note that full information on the approval of the study protocol must also be provided in the manuscript.

Field-specific reporting

Please select the one below that is the best fit for your research. If you are not sure, read the appropriate sections before making your selection.

☒ Life sciences ☐ Behavioural & social sciences ☐ Ecological, evolutionary & environmental sciences

For a reference copy of the document with all sections, see nature.com/documents/nr-reporting-summary-flat.pdf

Life sciences study design

All studies must disclose on these points even when the disclosure is negative.

Sample size	N/A: All data analyzed was either previously reported, or is simulated
Data exclusions	Except for the SARS-CoV-2 week-by-week vignette, all data from the original studies was used where available. When the original data included GISAID data, which was not made available publicly in the original studies, we excluded it from our analyses. For the SARS-CoV-2 week-by-week vignette, clear phylogenetic outliers (often involving an abnormally large number of mutations from the root) were excluded after visual inspection of short test runs, as described in the Methods; most of these outliers have been previously reported as unreliable (Supplementary Tables 1 and 2). For the H5N1 data, we excluded non-cattle samples, non-B3.13-genotype samples, and samples marked as retracted. We also removed samples without exact collection dates as described in the paper. A handful of additional samples were also excluded by visual inspection of initial test runs (documented in our analysis script), though these were later excluded anyway owing to lack of exact dates.
Replication	Not an experimental paper, but we strove to completely automate end-to-end our data collection, analyses and reporting in the accompanying data repository (https://github.com/broadinstitute/delphy-2026-paper-data)
Randomization	N/A
Blinding	N/A

Reporting for specific materials, systems and methods

We require information from authors about some types of materials, experimental systems and methods used in many studies. Here, indicate whether each material, system or method listed is relevant to your study. If you are not sure if a list item applies to your research, read the appropriate section before selecting a response.

Materials & experimental systems

n/a	Involved in the study
☒	☐ Antibodies
☒	☐ Eukaryotic cell lines
☒	☐ Palaeontology and archaeology
☒	☐ Animals and other organisms
☒	☐ Clinical data
☒	☐ Dual use research of concern
☒	☐ Plants

Methods

n/a	Involved in the study
☒	☐ ChIP-seq
☒	☐ Flow cytometry
☒	☐ MRI-based neuroimaging

Plants

Seed stocks	*Report on the source of all seed stocks or other plant material used. If applicable, state the seed stock centre and catalogue number. If plant specimens were collected from the field, describe the collection location, date and sampling procedures.*
Novel plant genotypes	*Describe the methods by which all novel plant genotypes were produced. This includes those generated by transgenic approaches, gene editing, chemical/radiation-based mutagenesis and hybridization. For transgenic lines, describe the transformation method, the number of independent lines analyzed and the generation upon which experiments were performed. For gene-edited lines, describe the editor used, the endogenous sequence targeted for editing, the targeting guide RNA sequence (if applicable) and how the editor was applied.*
Authentication	*Describe any authentication procedures for each seed stock used or novel genotype generated. Describe any experiments used to assess the effect of a mutation and, where applicable, how potential secondary effects (e.g. second site T-DNA insertions, mosiacism, off-target gene editing) were examined.*
