# Supplementary material for: Scalable near-real-time Bayesian phylogenetics for outbreaks with Delphy
Source: Nature. 2026 Sep 16;658(8136):747–56. doi: 10.1038/s41586-026-11012-6 (PMC13668698; doi:10.1038/s41586-026-11012-6)
Supplement: Supplementary file 3 [file 41586_2026_11012_MOESM3_ESM.pdf]

nature portfolio

Peer Review File

Scalable near-real-time Bayesian phylogenetics for outbreaks with Delphy

Corresponding Author: Professor Pardis Sabeti

Any redactions in this file are there to maintain patient confidentiality, the confidentiality of unpublished data, or to remove third-party material.

This file contains all reviewer reports in order by version, followed by all author rebuttals in order by version.

Version 0:

Reviewer comments:

Referee #1

(Remarks to the Author)
Statistical phylogenetics has been practiced for 4 decades and Bayesian phylogenetics for almost 3 decades. Although there have been many incremental optimizations, software today proceeds according to the same foundations as the original papers introducing these concepts: Felsenstein pruning, and classical MCMC iteration.

Another approach is to consider phylogenetic trees with mutations directly mapped onto the trees. This can be found in USHER, MAPLE, and the work by Lartillot, Nielsen and others. When I first looked into Delphy it seemed like an incremental update, merging these ideas together.

After a closer look, Delphy has required a _lot_ to make this new direction practical rather than just a proof of concept. N-pruning / missations, the parallelizable tree prior, and mdSPR moves are non-trivial technical advances.

More interestingly, they put these technical advances together into a package that is almost unbelievably easy to use.

The supplemental material is a remarkable document. Not every new software package provides proofs that their code is doing the right thing, or explains approximations. Indeed, there have been optimizations that have come out in phylogenetic software where the source code has had to be scrutinized to understand what they are doing.

All of that said, the practical improvement that these developments bring is not yet obvious from the manuscript as detailed below.

Benchmarks

These advances give a remarkable speedup for SARS-CoV-2. That's fantastic.

The speedups for the other viruses are less clear. At first it might seem as though if we have 11 logged parameters and 10/11 of them give about a 10x speedup (e.g. Supp fig 2) then this really corresponds to a 10x speedup in, say, tree exploration.

This doesn't necessarily follow. The current manuscript uses a different mix of proposals so it is difficult to interpret the ESSs of the various parameters. The moves for the continuous parameters are so awesomely fast that it makes sense to make them more frequently, which would boost their ESS.

In fact, the fact that the likelihood lags behind the other statistics makes me suspicious that the tree exploration may not be significantly improved for the non-SARS viruses. There is now quite a lot of work on the importance of assessing topological convergence (https://academic.oup.com/ve/article/10/1/veae081/7822753, https://doi.org/10.1093/molbev/msaa295) and that continuous proxies may not capture problems. There is also now a literature on means of assessing topological

convergence (https://doi.org/10.1093/gbe/evw171, https://besjournals.onlinelibrary.wiley.com/doi/full/10.1111/2041-210X.13727, https://doi.org/10.1214/22-ba1339). Specifically, it seems possible that the tree partitioning scheme could restrict the MCMC moves to be rather small.

Do the speedups come from the large number of sequences or the dense sampling? The manuscript seems ambiguous on this point. It says "Delphy achieved ~10x speedups as expected: its advantages grow with dataset size (see Discussion)." However, in the discussion it seems that the authors point more to dense sampling: "When confronted with datasets that are locally or globally far from parsimonious, Delphy degrades gracefully by remaining exact but slowing down, rather than silently introducing arbitrary artifacts and errors." It would be easy to address this question using dataset subsampling or simulation.

I was a little surprised to see the speed comparson be to BEAST 2 rather than BEAST X. BEAST X is more widely used for viruses and my impression is that the developers have focused more on speed than BEAST 2. It was also used in the O'Toole paper. In any case it would be easy to do the comparison given the models presented here, and indeed it appears that it's already in the AWS instance they use to run the benchmarks.

Could the authors provide a little more detail on how burn-in is handled with in the ESS comparison?

Relatedly, BEAST is not parallel-friendly, and the Delphy authors have done a bunch of great work making parallel tree moves possible. On the other hand, if the benchmarks are happening on a machine with 8 vCPUs, it would seem reasonable to compare the ESS accumulation of Delphy to that of 8 BEAST processes run independently.

While we are on the topic I don't think that this sentence makes the actual speedups especially clear: "By reproducing key analyses from recent major epidemics (Ebola, Zika, SARS-CoV-2, mpox, and H5N1), we demonstrate SOTA accuracy with up to 1,000x speedups." This isn't wrong but it is imprecise: the big speedups are unique to only one of those viruses.

Overall, a pessimistic view of this paper is that it just accumulates ESS quickly in the SARS-CoV-2 case by rapidly making small tweaks to a tree that is mostly quite well resolved. The authors have an opportunity to counter that pessimistic view.

Exposition

This paper seems like an unusual fit for Nature. There are no new biological results here, but rather a new algorithm to do something one can already do but faster and more easily.

It's not obvious that the short format works to the authors advantage. For example, Figure 1B introduces the key concepts that drive Delphy, but to actually understand what the symbology means one needs to read the supplement.

I love the web interface, but I don't know if I've ever seen screenshots of a UI in a Nature paper.

Overall, the figures are nice but aren't quite up to Nature standards. They frequently use too small of fonts.

The choice of the phrase "genetic prior" took some getting used to, as this is where the main likelihood computation happens. An alternate would be "augmented likelihood", where the augmentation is the sampling of the mutation events.

Other questions

As the authors note, parallelizing the coalescent is hard because the coalescent rate depends on what is happening in the other lineages. Would a birth-death prior, e.g. https://github.com/BEAST2-Dev/bdsky, be easier?

Is it obvious that the parallel tree proposals give an ergodic Markov chain? A proof seems challenging but perhaps some discussion is merited.

The license for this project is different than most phylogenetics programs. Let's imagine I have an idea about how to improve the project and I would like to build upon the remarkable foundation here. What do I do? In a usual academic project I would fork the repository and start hacking away, but here I would hesitate a little due to the license and the patent application. Perhaps the answer is that nobody is expected to contribute, but that's rather different than projects such as BEAST.

Details

I was a bit thrown off by the description of the equation for \cal{G}(T|θ) being in terms of the jump chain. In a traditional jump-chain formulation one would have the probability of any event times the conditional probability of that event being the observed one. This would correspond to a normalized Q, which is not what's here. After thinking about it a little, the conditioning gives a 1/Q_aa term, which is then cancelled by the constant in front of the Poisson distribution. I know that the authors have a separate proof in terms of Kolmogorov, but explaining the jump chain a little more would help.

Sometimes the "root" is short for "root sequence", however the highest internal node in the tree is called the "root node". One

way to disambiguate these if the authors want is to call what they call the "root node" the UCA, for universal common ancestor. This is just an optional suggestion!

L39: It is an exact formulation modulo approximations to the coalescent, correct?
L70: It's typically "MrBayes"
L745: "à priori": there should not be any accent on this. It's latin.

MAPLE is now published in Nature Genetics at doi: 10.1038/s41588-023-01368-0.

Supplementary typo: "whichever is closets"

(Remarks on code availability)
I have tried the web interface. The authors have done extensive work to make the analysis reproducible, though I have not tried reproducing it myself.

Referee #3

(Remarks to the Author)
Varilly et al. present a new strategy for phylogenetic and photodynamic inference to study infectious disease outbreaks. Their main innovation is a set of Markov chain Monte Carlo algorithms that operate on the extended state-space of phylogeneties and detailed histories of mutations mapped on these phylogenies. As the authors noted themselves, the ideas of using this extended state-space was "in the air" for more than two decades, but none of the previous approaches managed to scale up MCMC on this state-space without taking some shortcuts (e.g., fixing a phylogeny). One always has to wonder if such breakthroughs are "real" or just being oversold as "real" by the authors, but it looks real to me, so I am excited and hopeful about these developments. As far as I can tell, the success of Varilly et al.'s approach is a combination of them parallelizing computations across different parts of the phylogenetic tree and availability of genomic data collected in densely sampled outbreaks. The authors note that sparse sampling leads to loss of efficiency of their algorithm relative to standard approaches that rely on Felsenstein's pruning algorithm for phylogenetic likelihood calculation. This reliance on dense sampling (to the best of my understanding the operational definition here is that each branch of the phylogenetic tree has a very small number of mutations mapped on it probabilistically speaking) potentially explains why previous attempts at using trees and mutational histories were not able to outperform state-of-the-art phylogenetic methods. In summary, very promising work that has real potential to transform Bayesian analyses of pathogen genomic surveillance data.

Main critique points:

- I would like the authors to be a little more careful when they compare Bayesian and frequentist methods. I am mostly talking about the intro paragraph on page 2.
a. The authors state that Bayesian phylogenetics is the gold standard. I am not sure everyone will agree with this statement. It depends on what one wants to accomplish. For dating ages of the tree nodes, there is in principle nothing wrong with maximum likelihood, equipped with bootstrap to get confidence intervals for node ages. Non-Bayesian approaches become more cumbersome when one to add a hierarchical structures, but this has also been done in LAMARC and MIGRATE software packages. These tools are not popular in viral phylodynamics, but fundamentally they are comparable to BEAST.
b. I wouldn't call phylogeography parameters or effective population size "covariates." It could be confusing since in standard regression models covariates are assumed to be fixed/conditioned on, but objects that the authors refer to are unknown parameters or latent variables.

- The authors' description of the posterior P(T,\theta | data) on page 4 is not fully rigorous. For example, it completely omits the coalescent prior. In my opinion it is cleaner to represent an EMAT as (T, M), where T is the phylogeny and M is mutational history on this phylogeny. Then the authors can write P(T, M) = P(M | T) P(T), where P(M | T) is the CTMC complete data likelihood (product of exponential densities and tail probabilities) and P(T) is the coalescent likelihood. In the Methods section, the authors have the coalescent present; they use \pi_{anc}(T | \theta) in place of my suggested P(T), where both represent the Kingsman's coalescent density. It is strange to have two terms with T | \theta in parentheses. I think separating T into (T, M) per my suggestion is cleaner. I also strongly suggest calling P(M | T) complete data likelihood instead of a "genetic prior." Although missing data and parameters are both random variables in a Bayesian setting, this is not in a frequentist setting, so it is easier to follow a more universal notation.

- I am not fully convinced by the authors explanation of the disagreement between Delphy and BEAST2 when approximating the posterior distribution of the rate heterogeneity parameter \alpha. The authors blame BEAST2 discretization of the gamma distribution, which seems plausible. Can the authors confirm this hunch by increasing the number of gamma distribution discrete bins from 4 to 8 and then to 12? If the authors' explanation is correct, one would expect that increasing the number bins in BEAST2 should bring the two methods closer to each other.

Minor critique points:

- I would add RevBayes to the list of state-of-the-art Bayesian phylogenetic tools. This software is more actively developed than MrBayes and is comparable to BEAST in functionality.

- "Whereas traditional methods effectively perform this integration analytically, Delphy performs them it statistically" -> "Whereas traditional methods effectively perform this integration analytically, Delphy folds this integration into MCMC" -- this

is how Bayesian data augmentation is usually explained, so I would stick to this point of view.

- On page 25, in the Posterior distribution section, G(T | \theta) is written as G(T, \theta) in the itemized list below the formula.

(Remarks on code availability)
I poked around GitHub and run the software demo. I haven't really done any stress testing of the software though.

Referee #4

(Remarks to the Author)
I co-reviewed this manuscript with one of the reviewers who provided the listed reports.

(Remarks on code availability)

Referee #5

(Remarks to the Author)
This paper provides a comprehensive description of a new way to do Bayesian phylogenetics and represent a tremendous amount of work, the details of which are clearly written up. The authors went to great lengths to optimise every detail of the Delphy system, which now makes it possible to perform large scale Bayesian phylogenetic analyses with little effort. I enjoyed reading about the innovations in these optimisations, such as the clever exploitation of Jukes Cantor model to simulate new mutations, the exploitation of explicit mutations to inform candidate locations for SPR moves and application of Merkle trees to obtain summary trees to name just a few. The user interface is intuitive and works well, so I am sure it will be appreciated by the non-specialists that the software is catering for. This has the potential of revolutionising the way large scale pathogenic outbreaks will be managed.

I mainly concentrated on the technical details in the supplementary information, and have not been able to find any major flaws. However, there are a few things I would like to see clarified, mainly around validation of the implementation.

Correctness of the implementation is supported by pointing out results are identical to BEAST2 output. As proof, some marginal posterior and tree figures are presented. Given quite a few optimisations and approximations are involved and there are many technical details, from experience I would expect there to be at least some bugs, so thorough testing is required. I would not consider this sufficient evidence the implementation is correct. Performing a well calibrated simulation study (WCSS) is the current threshold of proof to demonstrate it is. Tools described in Mendes et al. (2025) can be used to do such studies.

A WCSS demonstrates measurable properties are correctly estimated, but does not make claims about the trees, and since new tree operators were introduced it is desirable to demonstrate the posterior tree distribution is correct as well. pg 20 line 338 says "The Delphy- and BEAST2-inferred MCC trees for the SARS-CoV-2 dataset show excellent agreement (Fig 3A and 3B)." is hard to verify by looking at a picture on PFD.
Please use clade support and height graph to demonstrate the tree distributions agree (e.g. as described here: https://www.beast2.org/2020/04/20/comparing-tree-sets.html). A nice to have, as part of the WCSS is to see "triangle graphs" (https://github.com/rbouckaert/DeveloperManual?tab=readme-ov-file#clade-coverage) to demonstrate clade support is correctly estimated.

MCC trees are used to summarise posterior tree sets, and though they were the default for BEAST2 users for many years, it should be acknowledged they are not good representatives of posterior distributions (Berling et al, 2025). Conditional clade distributions (CCDs), in particular the CCD0 variant, allow finding a maximum a posteriori tree efficiently, but is arguably more computationally complex to create. The Merkle tree approach will elevate some of that. I suppose a workaround is exporting the tree set and run TreeAnnotator with the CCD0 topology option, but would it be feasible to implement a CCD0 summary tree or any other type of summary tree that is at least not unrepresentative as MCC trees?

One use case is to use Delphy to find a reasonably good tree, then do post processing on the summary tree that comes out of Delphy, e.g. to infer some phylodynamic process. Delphy makes it now possible to do this for large number of sequences. However, the MCC tree produced by Delphy often contains negative branch lengths due to parent clade ages being estimated younger than their child ages. This is inevitable when using the mean age of observed clades as age estimates when there are many clades and most of them observed at low frequency (which is the case for many datasets Delphy is aiming at). The common ancestor approach to age estimation (Heled & Bouckaert, 2013) evades that problem. Can this be implemented, and if not, do you have suggestions to evade this problem?

A nice new method for handling missing data is introduced, and motivated by pg 14 line 243 "However, during SPR moves on subtrees with missing data, such imputation would require global rearrangements, as for example, in an SPR on a subtree whose tips have no data at site l, while the state of that site at P and P’ differs". This is unclear to me: tips can be annotated with labels just like branches can be annotated with mutations (a minor benefit is that the same technique works for dealing with ambiguous characters). These values can be resampled occasionally, perhaps more often when involved in SPR moves, but their contribution can be integrated out by MCMC. This may be less efficient than using missations, but do not require global rearrangements with every SPR move. Would it be possible to provide a better motivation for missation handling?

The Supp info contains all technical details of interest, is well written and easy to follow when reading linearly, but it is hard to track down symbol definitions. It could benefit from a table listing all symbols used in the paper so that, for example, reading Eq (26) it can be easily checked what the definitions are of all symbols.

Minor things:

Eq(6) appears to have extra p before G on LHS of equation.

pg7 line 129ff Why call it "genetic prior" while at the same time explaining "The genetic prior functions similarly to the Felsenstein tree likelihood". Feels more natural to call it a (tree) likelihood and incorporate what is called likelihood on line 132.

pg14 line 237 "We note that mdSPR's robustness degrades when many tip dates have large uncertainties, so Delphy is currently unsuitable for analysing such datasets." Can you speculate what can be the cause of this behaviour? Is it because long branch lengths can be introduced containing many mutations perhaps?

pg 21 line 354 "Delphy achieves a 1000x higher statistical efficiency than BEAST2" v2.6, while BEAST v2.7 has been available since September 2022 and has a number of operator improvements specifically targeting mixing of tree distributions with tip dates. A fairer comparison is to v2.7 instead of v2.6.

Maybe I overlooked it, but how are site patterns treated? Equal site patterns get same mutation history or does each site gets its own? If the former, how is it verified this is correct?

pg 40 line 502: what is a "96 vCPU AWS instance"?

pg 42 line 532 "Separately, our parallelization implementation needs further optimization; for example, the 100,000-sample run used less than 25% of the machine's compute capacity at any given time." Could this be because at that size calculation is memory bound instead of CPU bound, or is it a matter of changing operator frequency, or something else?

pg 39 Figure 6: where does the geographical reconstruction come from?

Supplementary figure 17: What is the difference between closed and open circle (path 3 vs 4)?

Supp info pg 39 "even though mdSPR exposes no tunable parameters" f could be a tunable parameter?

Supp info pg 43 k_c in Eq (29) can be non-integer?

Supp info pg 17 "where T is the total branch length of the tree and M is the total number of mutations on it" add " and L is total number of sites"

Supp info pg 39 supp info "moveset"?

Supp info pg 49 "fingerprints are all essentially independent random C-bit fingerprints." Since internal node fingerprints within a tree are functions of tips below the node, this seems the opposite of independent. Can this be substantiated? Did you verify experimentally the collision numbers for tree sets produced by Delphy?

Nice to see the number of mutations for the COVID demo in the web interface converging to the number of the BEAST.

References

L Berling, J Klawitter, R Bouckaert, D Xie, A Gavryushkin, A Drummond. Accurate Bayesian phylogenetic point estimation using a tree distribution parameterized by clade probabilities PLOS Computational Biology 21.2 (2025) DOI:10.1371/journal.pcbi.1012789

Heled and Bouckaert: Looking for trees in the forest: summary tree from posterior samples. BMC Evolutionary Biology 2013 13:221.

Mendes FK, Bouckaert R, Carvalho LM, Drummond AJ. How to validate a Bayesian evolutionary model. Systematic Biology. 2024 Nov 7:syae064. DOI:10.1093/sysbio/syae064

Remco Bouckaert

(Remarks on code availability)
The code is nicely commented and well organised.

The code runs flawless in browsers and the binary for Linux worked on an x86 Ubuntu computer.

However, I ran into problems building the program from source. Installation instructions may require some improvements. Perhaps mention 'cmake' as requirement (had to install separately on OS X). Could not get it to go due to compiler error:
```
error: unknown warning option '-Werror=maybe-uninitialized'; did you mean '-Werror=uninitialized'? [-Werror,-Wunknown-warning-option]
```
Editing CMakeLists.txt
-Wno-error=maybe-uninitialized # Ignore something in abseil's raw_hash_set.hpp that I cannot fix
=>
-Wno-error=uninitialized # Ignore something in abseil's raw_hash_set.hpp that I cannot fix
resulted in
```
In file included from /Users/remco/workspace-c/delphy/core/ancestral_tree_prober.cpp:1:
In file included from /Users/remco/workspace-c/delphy/core/ancestral_tree_prober.h:4:
In file included from /Users/remco/workspace-c/delphy/core/tree_prober.h:6:
/Users/remco/workspace-c/delphy/core/estd.h:31:23: error: no type named 'range' in namespace 'std::ranges'
template<std::ranges::range R>
~~~~~~~~~~~~~~^
```

On linux Ubuntu 22.4 under UTM (i.e. using ARM not x86)
```
(delphy-venv) rrb@macbookpro:~/workspace/delphy> cmake --preset conan-debug
CMake Error: Could not read presets from /home/rrb/workspace/delphy-1.0.2: Unrecognized "version" field
```

On linux 20.4 x86:

[85%] Building CXX object core/CMakeFiles/delphy_core.dir/very_scalable_coalescent.cpp.o
In file included from /home/rbou019/workspace-c/delphy/core/phylo_tree_calc.cpp:1:
/home/rbou019/workspace-c/delphy/core/phylo_tree_calc.h:4:10: fatal error: span: No such file or directory
#include
^~~~~~
compilation terminated.

And on linux 22.4 x86 again:
```
(delphy-venv) rbou019@sc466327:~/workspace-c/delphy$ cmake --preset conan-debug
CMake Error: Could not read presets from /home/rbou019/workspace-c/delphy: Unrecognized "version" field
```

Version 1:

Reviewer comments:

Referee #1

(Remarks to the Author)
The authors have done a great job of responding to reviews and improving the paper.

Personally, I'm very happy they chose to change the license and I hope that this stimulates more interest and engagement with the community. Bravo!

(Remarks on code availability)
This is a modern project that built easily (I only had to modify Conan's settings.yml for clang 17). There is extensive test coverage, which is rare for a bioinformatics program. As far as the code structure is concerned, one could argue that the Run god class is a structural wart, but overall this is well above current standards in the field.

Referee #3

(Remarks to the Author)
Good job revising the manuscript. Implementation of Skygrid is impressive! I am happy with the current version of the manuscript and have no further requests for the authors.

(Remarks on code availability)
I ran the new demos. Core availability and user interfaces look superb.

Referee #4

(Remarks to the Author)
I co-reviewed this manuscript with one of the reviewers who provided the listed reports.

(Remarks on code availability)

Referee #5

(Remarks to the Author)
Thanks for the authors of addressing most of my questions in such great detail. Almost all my concerns are addressed by the authors. The list of symbols in the supp info is much appreciated.

Something that somewhat surprised me is that no WCSS is included in the ms, even though the authors say they considered it. The argument that it would be too computationally expensive is not convincing -- I have done WCCSs with BEAST2 using 1000 taxa trees, which if replicated with Delpy would already give substantial evidence there are no major issues with the implementation. Not having this evidence for even smaller cases than aimed for puts doubt on whether it works for larger cases.

Also, I do not understand that the trees pictured in figures sourced from different programs are presented as evidence of correctness, given these trees invariably differ, sometimes substantially. This is not surprising, given that MCC trees are inherently unstable, making it hard to get the same pair of summary trees from even the same software package when run with different random seeds. I suggested a simple alternative to visualise this, which has been ignored. Another way of measuring tree mixing is presented based on Magee et al. (2024), but not shown to measure agreement between different runs from different software packages as far as I can tell.

My experience is that even after decades of BEAST development we are still are finding bugs. Altogether, it seems to me that the authors take on a substantial risk in leaving some parts of validating their method unexplored.

Probably due to some minor configuration issue, trying to compile the code from source, I am getting this error both on M1 and Ubuntu:

```
CMake Error at CheckGit.cmake:58 (LIST):
LIST index: 1 out of range (-1, 0)
```

On Mac, this may have to do with brew not properly installing emscripten:

```
~> brew install cmake emscripten
.
.
.
==> Installing node dependency: z3
Error: An exception occurred within a child process:
FormulaUnavailableError: No available formula with the name "formula.jws.json".
```

This is the full error log for running cmake afterwards:

```
(delphy-venv) SC442557:delphy remco$ cmake --preset conan-debug
-- Using Conan toolchain: /private/tmp/delphy/build/debug/conan_toolchain.cmake
-- Conan toolchain: Defining libcxx as C++ flags: -stdlib=libc++
-- Conan toolchain: C++ Standard 17 with extensions ON
-- The C compiler identification is AppleClang 14.0.0.14000029
-- The CXX compiler identification is AppleClang 14.0.0.14000029
-- Detecting C compiler ABI info
-- Detecting C compiler ABI info - done
-- Check for working C compiler: /usr/bin/cc - skipped
-- Detecting C compile features
-- Detecting C compile features - done
-- Detecting CXX compiler ABI info
-- Detecting CXX compiler ABI info - done
-- Check for working CXX compiler: /usr/bin/c++ - skipped
-- Detecting CXX compile features
```

```
-- Detecting CXX compile features - done
-- Warning: Standard value defined in conan_toolchain.cmake to 17 has been modified to 20 by /tmp/delphy/CMakeLists.txt
CMake Error at CheckGit.cmake:58 (LIST):
LIST index: 1 out of range (-1, 0)
Call Stack (most recent call first):
CheckGit.cmake:114 (CheckGitVersion)
CMakeLists.txt:22 (CheckGitSetup)


-- Conan: Component target declared 'Microsoft.GSL::GSL'
-- Performing Test CMAKE_HAVE_LIBC_PTHREAD
-- Performing Test CMAKE_HAVE_LIBC_PTHREAD - Success
-- Found Threads: TRUE
-- Conan: Component target declared 'Eigen3::Eigen'
-- Conan: Component target declared 'Boost::headers'
-- Conan: Component target declared 'Boost::boost'
-- Conan: Target declared 'boost::boost'
-- Performing Test ABSL_INTERNAL_AT_LEAST_CXX17
-- Performing Test ABSL_INTERNAL_AT_LEAST_CXX17 - Success
-- Performing Test ABSL_INTERNAL_AT_LEAST_CXX20
-- Performing Test ABSL_INTERNAL_AT_LEAST_CXX20 - Success
CMake Deprecation Warning at /private/tmp/delphy/build/debug/_deps/googletest-src/CMakeLists.txt:4
(cmake_minimum_required):
Compatibility with CMake < 3.10 will be removed from a future version of
CMake.

Update the VERSION argument <min> value. Or, use the <min>...<max> syntax
to tell CMake that the project requires at least <min> but has been updated
to work with policies introduced by <max> or earlier.


CMake Deprecation Warning at /private/tmp/delphy/build/debug/_deps/googletest-src/googlemock/CMakeLists.txt:39
(cmake_minimum_required):
Compatibility with CMake < 3.10 will be removed from a future version of
CMake.

Update the VERSION argument <min> value. Or, use the <min>...<max> syntax
to tell CMake that the project requires at least <min> but has been updated
to work with policies introduced by <max> or earlier.


CMake Deprecation Warning at /private/tmp/delphy/build/debug/_deps/googletest-src/googletest/CMakeLists.txt:49
(cmake_minimum_required):
Compatibility with CMake < 3.10 will be removed from a future version of
CMake.

Update the VERSION argument <min> value. Or, use the <min>...<max> syntax
to tell CMake that the project requires at least <min> but has been updated
to work with policies introduced by <max> or earlier.


-- Found Python: /private/tmp/delphy/delphy-venv/bin/python3.12 (found version "3.12.3") found components: Interpreter
-- Configuring incomplete, errors occurred!


(delphy-venv) SC442557:delphy remco$ cmake --preset conan-debug
-- Using Conan toolchain: /private/tmp/delphy/build/debug/conan_toolchain.cmake
-- Conan toolchain: Defining libcxx as C++ flags: -stdlib=libc++
-- Conan toolchain: C++ Standard 17 with extensions ON
-- Warning: Standard CMAKE_CXX_STANDARD value defined in conan_toolchain.cmake to 17 has been modified to 20 by
/tmp/delphy/CMakeLists.txt
CMake Error at CheckGit.cmake:58 (LIST):
LIST index: 1 out of range (-1, 0)
Call Stack (most recent call first):
CheckGit.cmake:114 (CheckGitVersion)
CMakeLists.txt:22 (CheckGitSetup)


-- Conan: Component target declared 'Microsoft.GSL::GSL'
```

```
-- Conan: Component target declared 'Eigen3::Eigen'
-- Conan: Component target declared 'Boost::headers'
-- Conan: Component target declared 'Boost::boost'
-- Conan: Target declared 'boost::boost'
CMake Deprecation Warning at /private/tmp/delphy/build/debug/_deps/googletest-src/CMakeLists.txt:4
(cmake_minimum_required):
Compatibility with CMake < 3.10 will be removed from a future version of
CMake.

Update the VERSION argument <min> value. Or, use the <min>...<max> syntax
to tell CMake that the project requires at least <min> but has been updated
to work with policies introduced by <max> or earlier.


CMake Deprecation Warning at /private/tmp/delphy/build/debug/_deps/googletest-src/googlemock/CMakeLists.txt:39
(cmake_minimum_required):
Compatibility with CMake < 3.10 will be removed from a future version of
CMake.

Update the VERSION argument <min> value. Or, use the <min>...<max> syntax
to tell CMake that the project requires at least <min> but has been updated
to work with policies introduced by <max> or earlier.


CMake Deprecation Warning at /private/tmp/delphy/build/debug/_deps/googletest-src/googletest/CMakeLists.txt:49
(cmake_minimum_required):
Compatibility with CMake < 3.10 will be removed from a future version of
CMake.

Update the VERSION argument <min> value. Or, use the <min>...<max> syntax
to tell CMake that the project requires at least <min> but has been updated
to work with policies introduced by <max> or earlier.


-- Configuring incomplete, errors occurred!
```

(Remarks on code availability)
Looked at the code for the first round, but not for the second.

Version 2:

Reviewer comments:

Referee #3

(Remarks to the Author)
The authors' 2nd revision of the manuscript is very responsive to reviewers' critiques. Good job stress testing MCMC implementation. The Well-Calibrated Simulation Study section is convincing. Clade correlation has a some kinks, but the authors explained them plausibly.

(Remarks on code availability)
I've reviewed the code last time. Not much changed since then.

Open Access This Peer Review File is licensed under a Creative Commons Attribution 4.0 International License, which permits use, sharing, adaptation, distribution and reproduction in any medium or format, as long as you give appropriate credit to the original author(s) and the source, provide a link to the Creative Commons license, and indicate if changes were made.
In cases where reviewers are anonymous, credit should be given to 'Anonymous Referee' and the source.
The images or other third party material in this Peer Review File are included in the article's Creative Commons license, unless indicated otherwise in a credit line to the material. If material is not included in the article's Creative Commons license and your intended use is not permitted by statutory regulation or exceeds the permitted use, you will need to obtain permission directly from the copyright holder.
To view a copy of this license, visit https://creativecommons.org/licenses/by/4.0/

Summary of Major Revisions

We have substantially revised the manuscript in response to the reviewers' comments. These revisions include new methodological extensions, expanded large-scale benchmarks, and additional analyses that directly address concerns about rigor, generality, scalability, and convergence. Below we summarize the principal changes.

1. Extension to flexible population dynamics.

We have extended Delphy to support a standard flexible population model (Skygrid), enabling analysis of outbreaks that grow, plateau, and recede over multi-year timescales. This addition allows Delphy to operate in epidemiological regimes representative of mature and heterogeneous outbreaks, rather than being limited to early exponential growth. We include multiple analyses demonstrating the correctness, stability, and practical behavior of this implementation.

2. Expanded benchmarking on large, real-world outbreak datasets.

With the addition of Skygrid, we now benchmark Delphy on a broader and more decisive set of real-world datasets beyond SARS-CoV-2. These include the ~1,600-sequence Ebola dataset from the 2014–16 West Africa outbreak and the ~3,300-sequence H5N1 dataset from the ongoing U.S. cattle outbreak. Across these settings, Delphy shows close agreement with state-of-the-art Bayesian tools while achieving 2–3 orders of magnitude gains in efficiency, demonstrating that its advantages generalize across pathogens, outbreak histories, and sampling densities.

3. Updated comparisons against current state-of-the-art tools.

We have rerun all benchmarks against the latest versions of BEAST 2 and BEAST X, rather than earlier releases available during Delphy's initial development. While recent improvements in these tools narrow performance differences for smaller datasets, Delphy's scalability continues to yield substantial advantages as dataset size grows. To reflect these updated comparisons accurately, we have replaced earlier phrasing of "1,000× faster" with the more precise and conservative description of "2–3 orders of magnitude faster."

4. Explicit assessment of tree topology mixing and convergence.

To address concerns about topological convergence, we implemented a direct assessment of tree topology mixing using the frechetCorrelationESS metric (Magee et al., 2024). This analysis shows that Delphy does not suffer from the slow topological sampling raised as a potential concern. In the largest benchmark (H5N1), we observe small discontinuities in the trace for the number of mutations in some runs; these effects are not detectable by standard scalar diagnostics and may also arise in existing Bayesian phylogenetics tools, but they have negligible impact on primary quantities of interest such as population dynamics and tMRCA estimates.

5. Formal dedication of Delphy to the public domain.

We have formally dedicated Delphy's core technical innovations to the public domain and released the software under a permissive open-source MIT license. This step is intended to remove barriers to adoption, validation, and extension, and to support unrestricted use of Delphy for research, disease surveillance, outbreak analysis, and public health action.

Referee #1

Benchmarks

These advances give a remarkable speedup for SARS-CoV-2. That's fantastic.

The speedups for the other viruses are less clear. At first it might seem as though if we have 11 logged parameters and 10/11 of them give about a 10x speedup (e.g. Supp fig 2) then this really corresponds to a 10x speedup in, say, tree exploration.

This doesn't necessarily follow. The current manuscript uses a different mix of proposals so it is difficult to interpret the ESSs of the various parameters. The moves for the continuous parameters are so awesomely fast that it makes sense to make them more frequently, which would boost their ESS.

In fact, the fact that the likelihood lags behind the other statistics makes me suspicious that the tree exploration may not be significantly improved for the non-SARS viruses. There is now quite a lot of work on the importance of assessing topological convergence (https://academic.oup.com/ve/article/10/1/veae081/7822753, https://doi.org/10.1093/molbev/msaa295) and that continuous proxies may not capture problems. There is also now a literature on means of assessing topological convergence (https://doi.org/10.1093/gbe/evw171, https://besjournals.onlinelibrary.wiley.com/doi/full/10.1111/2041-210X.13727, https://doi.org/10.1214/22-ba1339). Specifically, it seems possible that the tree partitioning scheme could restrict the MCMC moves to be rather small.

We thank the reviewer for this thoughtful and technically nuanced critique of our benchmarking strategy. We agree with the core concern: improvements in scalar ESS values alone, particularly in early SARS-CoV-2 datasets, are not sufficient to establish improved exploration of phylogenetic tree space, especially when different samplers prioritize different types of MCMC moves. This observation directly shaped how we revised both the benchmarks themselves and their presentation.

A primary goal of the revision was therefore to **move beyond SARS-CoV-2 and to explicitly test Delphy's behavior on large, non-SARS outbreaks with different epidemic structures**. To that end, we expanded the benchmarks to include two substantial real-world datasets: the approximately 1,600-sequence Ebola dataset from the 2014–16 West Africa outbreak and the approximately 3,300-sequence H5N1 dataset from the ongoing U.S. cattle outbreak. These datasets were chosen specifically to assess whether Delphy's advantages persist outside the early, densely sampled SARS-CoV-2 regime and under more complex population dynamics.

Within this framework, the figures are intended to answer distinct but complementary questions:

Figure 2 is intended to establish correctness and comparability of inference. This figure addresses a prerequisite concern: whether Delphy samples the same posterior distribution as existing Bayesian tools on well-characterized datasets. The close agreement between Delphy and BEAST X in maximum-clade-credibility trees, posterior distributions of key parameters, and inferred population dynamics demonstrates that Delphy's performance gains are not achieved by altering the underlying inference or sacrificing accuracy. This figure is therefore included to establish equivalence of inference, rather than to argue about mixing behavior.

Figure 5 is intended to probe scalability and tree topology exploration in a large, non-SARS outbreak. We fully agree with the reviewer that scalar ESS values can be misleading if continuous parameters mix rapidly while tree topology lags behind. In response to this concern, we added an explicit topology-based diagnostic using the `frechetCorrelationESS` metric introduced by Magee et al. (2024). This metric directly quantifies decorrelation in tree topology and is designed to detect precisely the failure mode raised by the reviewer. Applying this measure to the Ebola and H5N1 datasets shows that Delphy's tree topology ESS scales comparably to its scalar ESS, indicating that topological exploration is not lagging behind other dimensions of the posterior.

We also report, transparently, that in the largest H5N1 runs we observe small discontinuities in the trace for the number of mutations midway through some runs. These events are not detectable by standard scalar diagnostics and may plausibly occur in other Bayesian phylogenetics tools as well. Importantly, they have negligible impact on the primary quantities of interest, including inferred population dynamics, tMRCAs, and clade structure, all of which remain stable across runs.

Finally, to ensure fair and current comparisons, **we reran all benchmarks against the latest versions of BEAST 2 and BEAST X**. While recent improvements narrow performance differences for smaller datasets, Delphy's scalability yields substantial advantages for larger, densely sampled outbreak datasets, which are the primary target of this work. We have revised the text and softened quantitative claims accordingly to reflect this dependence.

We hope this revised benchmarking strategy and clearer explanation of the figures address the reviewer's concerns and clarify how the benchmarks are intended to assess both correctness and effective exploration of tree space.

For ease of review, we have included Figure 2 and Figure 5 below.

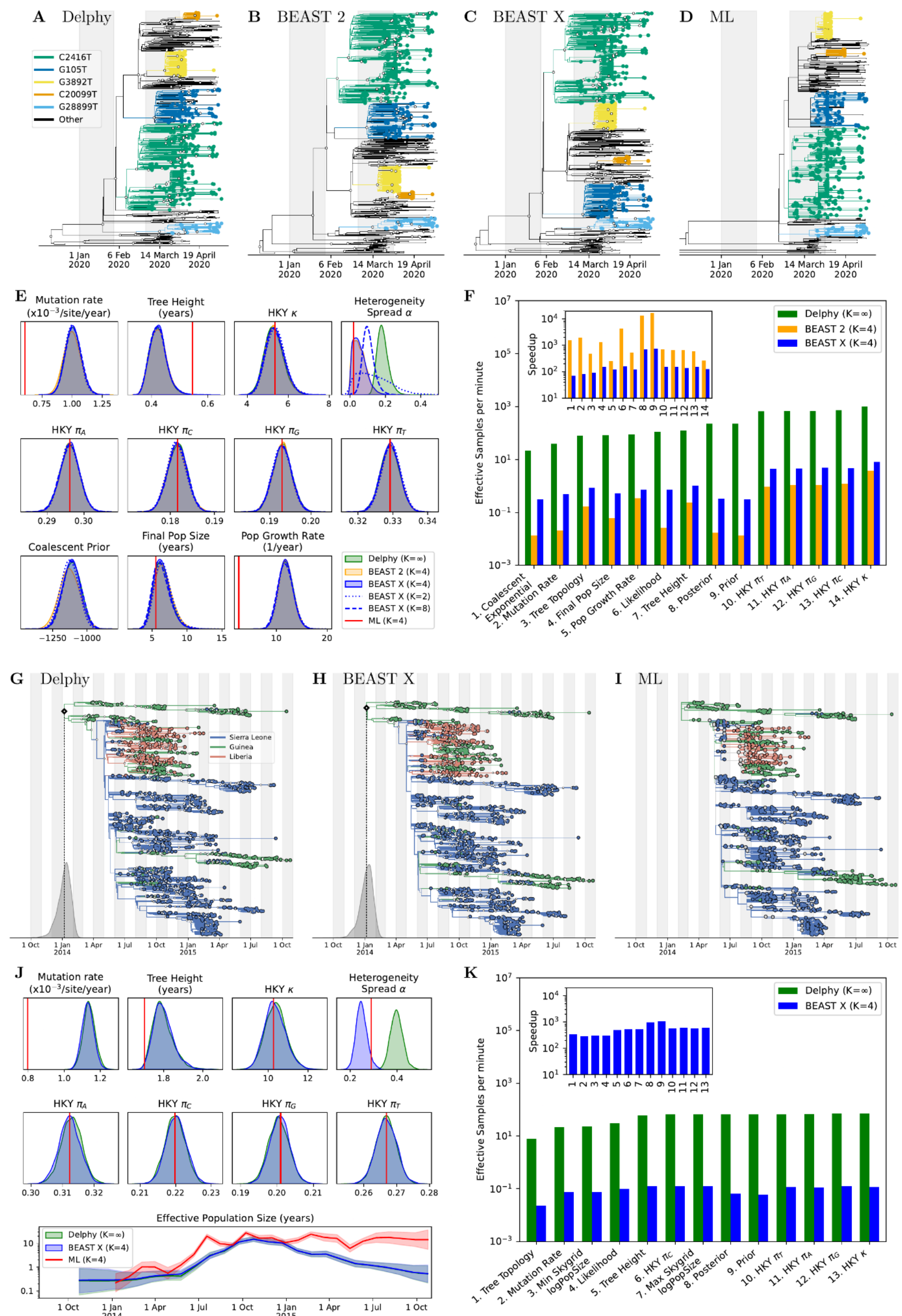

Figure 2: Delphy is consistent with existing Bayesian tools. A. Maximum-Clade-Credibility (MCC) tree for 757 SARS-CoV-2 samples from Lemieux et al. (Lemieux)[49] as produced by Delphy (results recapitulate Figure 3A in Lemieux[49]). Inner nodes with posterior support above 95% are marked with white dots; local rearrangements of low-support nodes are not highly significant and are expected even between identical finite runs. Interior labels are imputed via naïve parsimony on the MCC. **B,C.** Analog for BEAST X and BEAST 2. **D.** Maximum-likelihood timed tree, as calculated by IQ-Tree 2 and TreeTime. **E.** Distributions of key observables, as produced by Delphy (green) and BEAST 2 (orange), BEAST X (blue), and maximum-likelihood estimate (red); the discrepancy in the heterogeneity spread parameter α arises from Delphy's use of a continuous Gamma model vs. a K-category discrete approximation in the BEAST runs (Methods); the curves for K=2 (dotted line), K=4 (the standard setting, filled blue), and K=8 (dashed line) demonstrate the sensitivity of α to K and its convergence towards the Delphy result (K=∞). **F.** Runtime

efficiency of Delphy vs BEAST X and BEAST 2. **G,H.** MCC for the 1,610-sequence 2014-16 Ebola dataset from Dudas et al.[50]

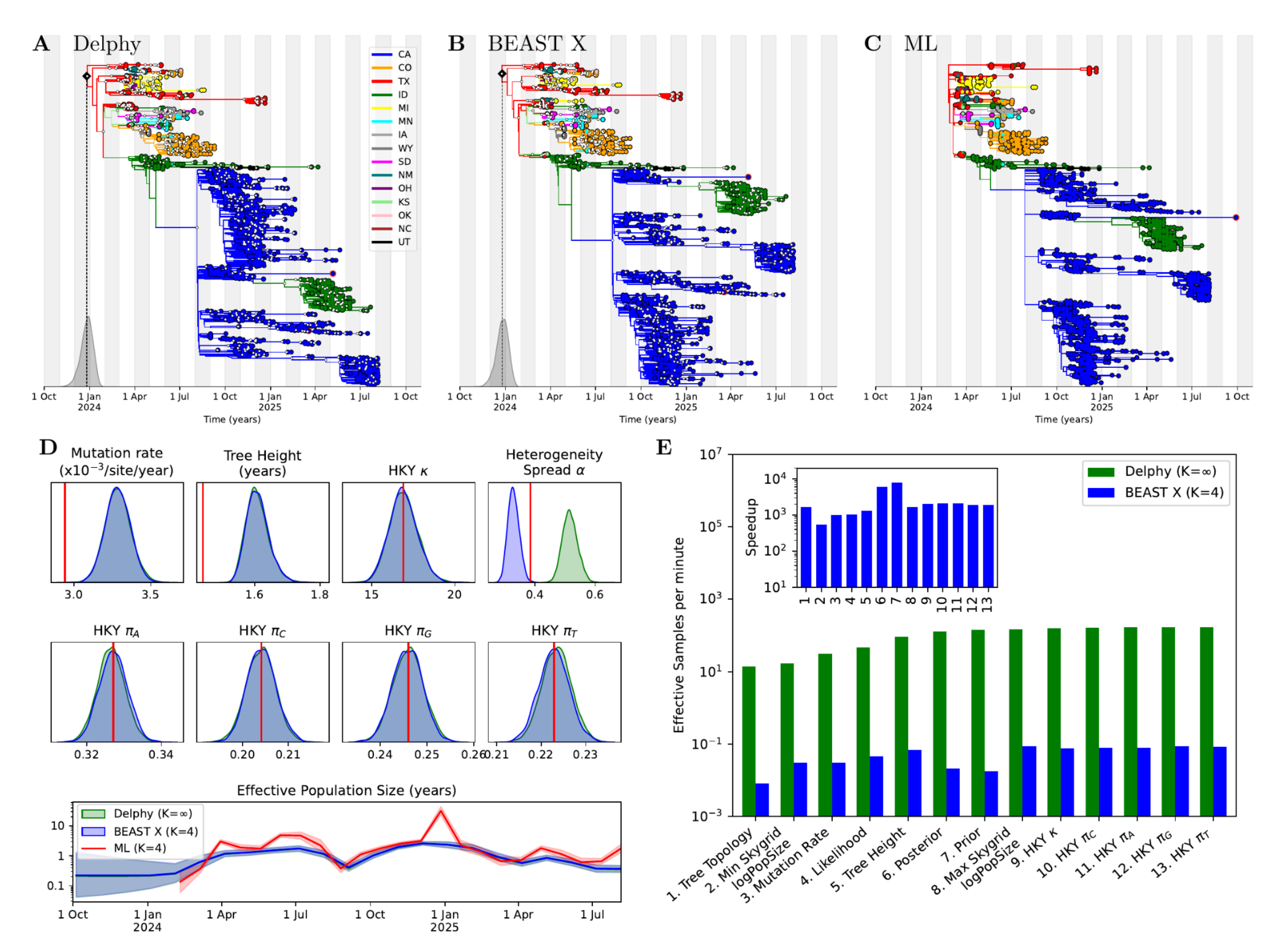

Figure 5: H5N1 influenza outbreak in U.S. cattle, 2024-2025. **A.** MCC tree for 3,339 H5N1 B3.13 sequences in cattle as produced by Delphy. Inner nodes with posterior support above 95% are marked with white dots; local rearrangements of low-support nodes are not highly significant and are expected even between identical finite runs. Phylogeographic reconstruction reflects naïve parsimony on the MCC. **B.** Analog for BEAST X. **C.** Maximum-likelihood timed tree, as calculated by IQ-Tree 2 and TreeTime. TreeTime flagged two tips as “outliers”, whose apparent and stated dates are highly inconsistent (red circles in A,B,C) **D.** Distributions of key observables. **E.** Runtime efficiency of Delphy vs BEAST X.

Regarding the source of Delphy's speedups, we agree that the original presentation did not sufficiently distinguish the roles of dense sampling and dataset size. Both effects contribute to performance, but they do so in different ways, and we have revised the Discussion to clarify this distinction.

Dense sampling is the dominant factor underlying Delphy's speedups in typical viral genomic epidemiology datasets. A pragmatic definition of dense sampling in this context is that the number of mutations on most branches does not exceed two. In this regime, the cost of Delphy's core MCMC moves scales with the number of mutations that must be considered, either because new mutation sequences are proposed on a branch, or because mutations along the path between two grafting points must be examined. When sampling is dense, most moves therefore have a small, approximately constant cost that does not grow with dataset size. By contrast, methods based on Felsenstein pruning incur a cost per move that scales with the total number of site patterns in the dataset, which typically increases with dataset size until saturation.

Dataset size contributes an additional, secondary advantage by enabling parallelism. Larger datasets provide more opportunities to partition the tree and execute independent local moves in parallel. This effect is most evident in our largest simulations, such as the 100,000-sequence runs, where Delphy is able to exploit many CPU cores effectively, even though the current implementation is not yet fully optimized for parallel efficiency.

To make this distinction concrete, we now explicitly highlight the Zika benchmark as a contrasting example. The Zika dataset (174 sequences with approximately 1,200 mutations, corresponding to roughly four mutations per branch) is the least densely sampled among our benchmarks. As expected under the above reasoning, Delphy's relative advantage is reduced in this setting: the tree topology ESS is approximately seven-fold lower in Delphy than in BEAST X for comparable runs, whereas in more densely sampled datasets the tree ESS values are similar. We have added a sentence highlighting this example in the Discussion to make this relationship explicit.

Because the scope of the paper already includes a broad set of real and simulated benchmarks, we did not further subdivide datasets or introduce additional simulations solely to isolate these effects. However, a more systematic exploration of the interplay between sampling density and dataset size would indeed be an interesting direction for future work.

> I was a little surprised to see the speed comparson be to BEAST 2 rather than BEAST X. BEAST X is more widely used for viruses and my impression is that the developers have focused more on speed than BEAST 2. It was also used in the O'Toole paper. In any case it would be easy to do the comparison given the models presented here, and indeed it appears that it's already in the AWS instance they use to run the benchmarks.

This is a fair point, and we agree that BEAST X is widely used for viral phylogenetics and is an important comparator for outbreak-focused analyses. The original comparisons to BEAST 2 reflect the historical context in which Delphy was developed. When this project began, the relevant BEAST X performance improvements were not yet available, and the SARS-CoV-2 analysis that originally motivated Delphy (Lemieux et al., 2021) was carried out using BEAST 2.

At that time, we reasoned that because the dominant computational cost in both BEAST 2 and BEAST X arises from Felsenstein pruning implemented in the shared BEAGLE library, the choice of BEAST 2 versus BEAST X

as the orchestration layer would not materially affect the comparison. We also ensured that the most recent version of BEAGLE available at the time was used in the original benchmarks.

Based on this comment, and similar feedback from Reviewer #5, we have now rerun all benchmarks against the latest versions of both tools (BEAST 2 v2.7.7 and BEAST X v10.5.0, as of September 2025). These updated comparisons materially strengthen the manuscript. They confirm that both BEAST 2 and BEAST X have made substantial performance improvements since the project began, particularly through more effective proposal mechanisms. They also show, however, that while these improvements narrow performance differences for smaller datasets, Delphy's advantages remain pronounced for larger datasets, where scalability becomes the dominant factor.

For the smallest benchmark (the 81-sequence Ebola dataset from Gire et al., 2014), BEAST X performance is now closer to Delphy's, which we view as an expected and reassuring outcome. For larger datasets, Delphy continues to achieve order-of-magnitude improvements in efficiency. We have revised the manuscript to reflect these updated comparisons, giving greater prominence to benchmarks at scales that are increasingly representative of modern genomic surveillance and softening quantitative claims accordingly.

We believe these changes improve both the fairness and the clarity of the benchmarking comparisons and better align the manuscript with current practice in viral phylogenetics.

> Could the authors provide a little more detail on how burn-in is handled with in the ESS comparison?

Regarding how burn-in is handled in the ESS calculations, we have now made the procedure explicit in both the main text and the Methods. To avoid subtle or dataset-specific choices, we adopted a simple and deliberately conservative approach: for every run, we discard the first 30% of samples as burn-in.

We verified by visual inspection that no trace exhibited sustained drift beyond this cutoff. In a small number of H5N1 runs, we observed rare and minor discontinuities in the trace for the number of mutations, which we discuss explicitly in the H5N1 section. These events do not affect the inferred population dynamics, tMRCAs, or clade structure, and they occur well within the retained portion of the chains.

For reporting efficiency, the ESS per minute metric is computed by dividing the post–burn-in ESS by the total wallclock time of the run. One could alternatively divide by only the post–burn-in wallclock time, which would increase all ESS per minute values by a uniform factor of approximately 1/0.7, without affecting any relative speedup ratios. We therefore chose the more conservative normalization.

Instrumenting runs to record the exact wallclock time corresponding to burn-in would require rerunning all benchmarks and is unlikely to yield material differences. Our goal with these benchmarks is to quantify order-of-magnitude differences in performance rather than fine-grained constant factors, and the conclusions are insensitive to this choice.

> Relatedly, BEAST is not parallel-friendly, and the Delphy authors have done a bunch of great work making parallel tree moves possible. On the other hand, if the benchmarks are happening on a machine with 8 vCPUs, it would seem reasonable to compare the ESS accumulation of Delphy to that of 8 BEAST processes run independently.

This is a reasonable suggestion, and we considered comparing Delphy's ESS accumulation to that of multiple independent BEAST runs executed in parallel. We ultimately did not include such a comparison because, for typical Bayesian phylogenetic workflows, it would not be a fair comparison for BEAST. In practice, running multiple independent BEAST chains duplicates burn-in costs without reducing time to usable inference, which would make BEAST appear slower in wallclock time rather than providing a more informative baseline.

The core issue is that burn-in constitutes a substantial fraction of total runtime for the kinds of datasets and run lengths considered here. If a single chain spends time **T** in burn-in and then requires additional time proportional to the number of effective samples collected, running **K** independent chains in parallel does not reduce this initial cost. The wallclock time to obtain a fixed number of effective samples **N** assuming an autocorrelation time of **tau** is approximately T + (tau * N / K), rather than (T + tau * N) / K. With our conservative burn-in criterion of 30%, this places an upper bound of roughly a 3.3x speedup even with arbitrarily many parallel chains.

In addition, BEAST already exploits parallelism within a single chain. Both BEAST 2 and BEAST X parallelize Felsenstein pruning over site patterns, so wallclock time is already reduced when running on multi-core machines, although typically by much less than the number of available cores. As a result, running multiple independent BEAST chains in parallel on the same hardware would offer limited additional benefit beyond what is already captured by our single-chain benchmarks.

By contrast, Delphy's parallelization operates at a different level, enabling independent local tree moves across disjoint subtrees within a single chain. We emphasize that this parallelization is currently implemented conservatively to ensure correctness rather than to fully saturate available hardware. Indeed, the present implementation does not yet exploit all available parallelism; for example, a Delphy run on the Lemieux et al. SARS-CoV-2 dataset runs only about twice as fast on an 8-vCPU machine compared to a single core. For this reason, we believe that comparing a single BEAST run and a single Delphy run on the same hardware provides a conservative and well-matched assessment of relative performance.

Finally, we note that the wallclock time of a single run is what most users experience in practice. Beyond brief exploratory analyses, typical workflows involve running one or two long chains to a target ESS and then combining them for downstream interpretation. Our benchmarking choices were therefore designed to reflect this common usage pattern, rather than idealized parallel scaling scenarios that are rarely realized in routine analyses.

> While we are on the topic I don't think that this sentence makes the actual speedups especially clear: "By reproducing key analyses from recent major epidemics (Ebola, Zika, SARS-CoV-2, mpox, and H5N1), we demonstrate SOTA accuracy with up to 1,000x speedups." This isn't wrong but it is imprecise: the big speedups are unique to only one of those viruses.
>
> Overall, a pessimistic view of this paper is that it just accumulates ESS quickly in the SARS-CoV-2 case by rapidly making small tweaks to a tree that is mostly quite well resolved. The authors have an opportunity to counter that pessimistic view.

This is a helpful observation, and we agree that the original sentence overstated the uniformity of the speedups across datasets. While the statement was not incorrect, it did not sufficiently convey how the magnitude of the speedup depends on dataset characteristics, and in particular on scale and sampling density. We have revised the language accordingly.

In the revised manuscript, we have softened the headline claim to “2–3 orders of magnitude” and clarified that the largest speedups arise in large, densely sampled outbreak datasets. This change reflects both recent performance improvements in BEAST 2 and BEAST X and the fact that Delphy’s advantages are not constant across all datasets, but instead grow with dataset size and sampling structure.

We also took the reviewer’s broader concern seriously, namely the possibility that Delphy’s performance gains might be an artifact of rapidly accumulating ESS in early SARS-CoV-2 trees that are already largely resolved. To address this directly, we expanded the benchmarks to include large, non-SARS-CoV-2 datasets with distinct epidemic structures, specifically the Ebola and H5N1 outbreaks. These datasets demonstrate that the observed speedups are not unique to SARS-CoV-2, nor to a single well-resolved tree, but persist in settings with different population dynamics and tree structures.

More generally, Delphy’s performance gains arise from the combination of dense sampling, which makes individual MCMC moves inexpensive, and scalability, which allows those gains to compound as datasets grow. As discussed in the revised manuscript, this combination unlocks increasingly large advantages as outbreak datasets expand. Given current trends in sequencing cost and global surveillance capacity, we expect such large, densely sampled datasets to become increasingly common, making this regime central rather than exceptional.

We believe these revisions clarify the scope and interpretation of the reported speedups and directly address the pessimistic reading the reviewer raises.

### Exposition

This paper seems like an unusual fit for *Nature*. There are no new biological results here, but rather a new algorithm to do something one can already do but faster and more easily.

We appreciate the reviewer’s observation and agree that this manuscript differs from a typical biological discovery paper. At the same time, we believe the advance presented here is well aligned with *Nature*’s interest in work that introduces new scientific capabilities with broad consequences for how biology is studied and applied.

Delphy enables a fundamentally different mode of Bayesian phylogenetic analysis. By making such inference fast, scalable, and straightforward to use, it moves Bayesian phylogenetics from a retrospective, specialist activity to one that can be applied routinely and in near real time, including during ongoing outbreaks. This shift substantially expands the set of analyses that are practically feasible, rather than simply accelerating existing workflows.

In current practice, Bayesian phylogenetic methods are often avoided in operational settings because of their computational cost and complexity, despite their conceptual advantages. Delphy removes these barriers, enabling uncertainty-aware evolutionary inference at scales and on timelines that were previously inaccessible. This capability materially changes how genomic epidemiology can be carried out in public health contexts.

Our intent in submitting to *Nature* was to reach a broad community of biologists, epidemiologists, and public health researchers who rely on phylogenetic insight but are unlikely to engage with highly specialized computational journals. For this audience, the primary contribution is the new analytical capability that Delphy makes available.

Accordingly, the main text focuses on the conceptual advance and its practical implications, illustrated through demonstrations of accuracy, speed, accessibility, and scalability. Comprehensive technical details, algorithmic derivations, and fully reproducible benchmarks are provided in the Supplementary Information for readers who wish to examine the methodological advances in depth.

> It's not obvious that the short format works to the authors advantage. For example, Figure 1B introduces the key concepts that drive Delphy, but to actually understand what the symbology means one needs to read the supplement.

We agree that the short format imposes constraints on how much technical detail can be conveyed in the main text, and we therefore aimed to strike a balance between conceptual exposition in the main text and full technical development in the Supplementary Information. Figure 1, particularly panel B, is not intended to substitute for a detailed mathematical exposition. This was an intentional design choice rather than an oversight, and we appreciate the opportunity to clarify the role that Figure 1 is meant to play.

Figure 1 is designed primarily as a conceptual overview that answers the question “What does Delphy do?” rather than “How is Delphy implemented in full detail?” For readers who are not deeply familiar with the mechanics of Bayesian phylogenetics, the figure emphasizes explicit mutations as the central organizing principle that enables Delphy’s improvements. In particular, it highlights how explicit mutations implicitly encode sequences along the tree, allow posterior ratios to be evaluated locally and efficiently, enable mutation-directed SPR proposals to identify plausible regrafting points even over long distances, and make it plausible to parallelize MCMC moves over disjoint subtrees.

We agree that the mathematical notation in panels B and C can appear opaque at first glance. For more technically inclined readers, the notation is intended to connect to standard intuition about continuous-time Markov processes, in which mutations correspond to jumps and the posterior evaluates the probability of a particular sequence of waiting times and jumps. For readers who wish to engage with these details, the Supplementary Information provides a complete and rigorous derivation.

Finally, for readers who already understand Delphy, Figure 1B is intended to function as a compact mnemonic and calculational aid, making it straightforward to reason about posterior ratios for arbitrary MCMC moves without repeatedly referring back to the full derivations. In this sense, the figure is meant to operate at multiple levels, with the Supplementary Information serving as the authoritative source for full technical detail.

We believe this division of labor between the main text and the Supplementary Information is appropriate for the short format and for the broad readership of *Nature*, and we have ensured that readers seeking deeper understanding are fully supported by the supplementary material.

> I love the web interface, but I don't know if I've ever seen screenshots of a UI in a *Nature* paper.

We appreciate the reviewer’s reaction and agree that screenshots of a user interface are uncommon in *Nature* articles. We nonetheless felt it was important to include the web interface because it is central to what makes Delphy practically useful, rather than a peripheral implementation detail.

A core goal of Delphy is to make Bayesian phylogenetics accessible for real-world outbreak response. Outbreaks can emerge anywhere, often in settings without specialized computational expertise or infrastructure, and local public health teams are best positioned to analyze their own data and act on it. The web interface removes nearly all technical friction, allowing users to move directly from raw sequence data to uncertainty-aware phylogenetic insight without installation, configuration, or data transfer barriers. In this sense, the interface is part of the scientific contribution, as it enables a mode of use that would otherwise be impractical.

That said, we recognize the reviewer's and editor's preferences regarding presentation. To accommodate this, we have moved the figure showing the web interface to the Supplementary Information, while retaining references in the main text to guide interested readers. This preserves the focus of the main article while still documenting an essential component of the system.

> The choice of the phrase "genetic prior" took some getting used to, as this is where the main likelihood computation happens. An alternate would be "augmented likelihood", where the augmentation is the sampling of the mutation events.

We appreciate the reviewer's reaction and agree that the term "genetic prior" is nonstandard and may take some adjustment, particularly since it occupies a role analogous to where likelihood computations appear in more traditional formulations. We have added clarifying text in the main manuscript to motivate this choice and to explain how it relates to alternative terms such as "augmented likelihood" or "complete data likelihood."

At a pragmatic level, Delphy samples from the joint posterior distribution, and the separation of that posterior into "prior" and "likelihood" components is partly a matter of choice. During early development we did, in fact, use the term "augmented likelihood." We ultimately adopted "genetic prior" because it provides a more internally consistent separation between what constitutes the generative model and what constitutes observed data in Delphy's formulation.

In Delphy, the model explicitly includes the full ancestry and mutational history represented by an EMAT, together with associated parameters. Given such a model, the likelihood of the observed data reduces to whether the implied tip sequences agree with the observations (currently a binary condition, in the absence of sequencing error). In this framing, the probability of generating a particular mutational history under an evolutionary process naturally functions as a prior over models, rather than as a likelihood of observed data conditioned on a fixed tree. This contrasts with the usual Felsenstein pruning setup, where the "model" is the phylogeny without any detailed mutational history, and the likelihood of the "data" given the "model" is indeed the natural location for integrating over implicit mutational histories.

Looking forward, the main reason we strongly prefer "genetic prior" over "augmented likelihood" is to set the stage for a natural next step in our framework: modeling selective effects. Here, ancestry and genetics become tightly coupled. For example, in the context of a birth-death model, one might make the birth rate of a lineage dependent on the presence of a particular mutation. There is no conceptual difficulty in writing the probability for a joint birth-death-sampling-mutation Continuous-Time Markov Chain (CTMC) to generate a particular EMAT. Crucially, though, that probability no longer cleanly factorizes into an "ancestry prior" and a "genetic augmented likelihood". Our choice of "genetic prior" anticipates this difficulty, and results naturally from regarding the "model" being inferred by Delphy as the complete EMAT.

Nonetheless, we recognize that terminology choices are guided in part by reader familiarity, and we have worked to explain this distinction clearly in the revised text. If the editors or reviewers feel that an alternative term would ultimately be clearer for the intended audience, we would be open to making that change.

### Other questions

As the authors note, parallelizing the coalescent is hard because the coalescent rate depends on what is happening in the other lineages. Would a birth-death prior, e.g. https://github.com/BEAST2-Dev/bdsky, be easier?

Yes, a birth–death model would indeed be much easier to parallelize, and we agree with the reviewer that it is a natural fit for Delphy's framework. We are well aware that factorizable ancestry models, such as birth–death and epidemiological models, parallelize more naturally than the coalescent, precisely because their rates do not depend globally on the full set of active lineages.

In response to this point, we now explicitly note in the Discussion that Delphy has not yet been extended to directly factorizable ancestry models, including birth–death models. For this initial paper, our goal was to demonstrate that Delphy can handle the full complexity of standard practice in viral outbreak analysis, which has historically relied on coalescent-based models. Establishing correctness, scalability, and equivalence to existing Bayesian tools within that familiar framework was therefore a priority.

That said, we view birth–death models as a particularly promising direction for future work. In addition to enabling more straightforward and efficient parallelization, they naturally support tighter coupling between ancestry and sequence evolution, for example in models incorporating selection. Extending Delphy to birth–death priors will require substantial additional development and validation, and is therefore beyond the scope of the present manuscript, but it is very much an intended direction for future work.

Is it obvious that the parallel tree proposals give an ergodic Markov chain? A proof seems challenging but perhaps some discussion is merited.

This is an important question. A full formal proof of ergodicity for the parallel proposal scheme would indeed be challenging, but we believe the conditions under which ergodicity is preserved are reasonably clear, and we have added discussion in the main text to make this explicit.

At a high level, the key observation is that Delphy's parallel proposals are equivalent to executing standard local tree moves serially in some arbitrary interleaving. As noted in the main text (Figure 1F), parallel execution does not introduce new move types or restrict the underlying proposal space; it simply attempts multiple independent local moves simultaneously. Provided that all configurations remain reachable with nonzero probability using such serial moves, the Markov chain remains ergodic.

More concretely, any pair of phylogenetic trees can be transformed into one another through a finite sequence of SPR moves. In Delphy's parallel scheme, SPR moves are restricted to operate within subtrees during a given parallel phase. The potential concern is that a required SPR move might span multiple subtrees. To address this, Delphy periodically repartitions the tree, and under this repartitioning there is a nonzero probability that any given pair of nodes will eventually be assigned to the same subtree. As a result, every SPR

move that could be proposed in a serial setting can also be proposed, with nonzero probability, in the parallel setting. This is sufficient to preserve ergodicity.

We agree with the reviewer that ergodicity alone does not guarantee good mixing. In the extreme case of partitioning a tree into very small subtrees, topological rearrangements could be severely restricted, leading to poor mixing. To avoid this failure mode, Delphy enforces a minimum subtree size (currently requiring at least 10 internal nodes per subtree), which ensures that meaningful topological moves remain available within each parallel phase. The benchmarks included in the manuscript are intended, in part, to provide empirical evidence that this heuristic does not lead to pathological mixing behavior in practice.

We believe this combination of theoretical reachability and empirical validation provides a reasonable justification for the correctness of the parallel proposal scheme at the level appropriate for this manuscript.

> The license for this project is different than most phylogenetics programs. Let's imagine I have an idea about how to improve the project and I would like to build upon the remarkable foundation here. What do I do? In a usual academic project I would fork the repository and start hacking away, but here I would hesitate a little due to the license and the patent application. Perhaps the answer is that nobody is expected to contribute, but that's rather different than projects such as BEAST.

We appreciate this question, as it speaks directly to how Delphy is intended to live within the phylogenetics community. Our goal has always been broad adoption, scrutiny, and extension of the work, and not to discourage engagement or contribution.

We should clarify that patenting is not our default posture as an academic group. Being at the Broad, we have been persuaded by a model in which an initial filing can be used to formally disclose the full technical details of the system, while preserving the option to ensure that the work ultimately remained broadly accessible to the community. Our intent was protective rather than exclusionary.

That said, through feedback from many researchers in the field, including the reviewers, it became clear that this posture created hesitation and uncertainty for exactly the kind of open-ended contribution the reviewer describes. We took that feedback seriously. As a result, we have now relicensed Delphy under a permissive MIT license and withdrawn the patent application, explicitly dedicating the disclosed material to the public domain.

Under this model, Delphy can be freely forked, modified, and extended, in the same spirit as community projects such as BEAST. We very much hope that others will build on this foundation, experiment with new models, and contribute improvements back to the ecosystem, and we would be delighted for members of the community to build on this work and “start hacking away” :)

> **### Details**
>
> I was a bit thrown off by the description of the equation for \cal{G}(T|θ) being in terms of the jump chain. In a traditional jump-chain formulation one would have the probability of any event times the conditional probability of that event being the observed one. This would correspond to a normalized Q, which is not what's here. After thinking about it a little, the conditioning gives a 1/Q_aa term, which is then cancelled by

the constant in front of the Poisson distribution. I know that the authors have a separate proof in terms of Kolmogorov, but explaining the jump chain a little more would help.

This is a helpful observation, and we agree that the original presentation of G(T | theta) in terms of the jump chain could benefit from additional explanation, particularly for readers approaching the formulation from a traditional jump-chain perspective.

In response, we have expanded Section 3.2 to include a more explicit derivation of the genetic prior G(T | theta) in terms of waiting-time distributions and the probabilities of selecting specific mutation events after each waiting period. This expanded discussion makes clear how the apparent discrepancy with a normalized jump-chain formulation is resolved, including the cancellation of the 1 / Q_aa term by the normalization constant in the Poisson process.

We believe this additional derivation complements the Kolmogorov-based proof already provided and makes the connection to standard continuous-time Markov chain intuition more transparent for readers.

Sometimes the "root" is short for "root sequence", however the highest internal node in the tree is called the "root node". One way to disambiguate these if the authors want is to call what they call the "root node" the UCA, for universal common ancestor. This is just an optional suggestion!

Much appreciated. We have revised the manuscript throughout to explicitly distinguish between the “root sequence” and the “root node,” eliminating any ambiguity in usage. We agree that this clarification improves readability, and we have updated all references to the root accordingly.

L39: It is an exact formulation modulo approximations to the coalescent, correct?

Yes, that is correct. We now make this distinction explicit in the Discussion. Delphy’s genetic component is an exact reformulation of Bayesian phylogenetics, while the approximation arises in the parallelized treatment of the coalescent prior.

This approximation has a measurable effect in the largest benchmarks, including the H5N1 dataset and the 100,000-sequence simulations, where a finer discretization of time is required to closely match BEAST results and simulation parameters, respectively. We note this behavior explicitly in the revised manuscript.

We also clarify that this approximation is not fundamental. If parallelization were disabled, an exact coalescent prior based on tree intervals could be used, yielding exact agreement with standard Bayesian phylogenetics. Likewise, if ancestry were modeled using a birth–death process rather than a coalescent, no approximation would be required, as such models are directly factorizable.

L70: It's typically "MrBayes"

Noted and fixed.

L745: "à priori": there should not be any accent on this. It's latin.

Noted and fixed.

MAPLE is now published in *Nature Genetics* at doi: 10.1038/s41588-023-01368-0.

Noted and fixed.

Supplementary typo: "whichever is closets"

Noted and fixed. We thank the reviewer for their careful reading and attention to detail.

Referee #1 (Remarks on code availability):

I have tried the web interface. The authors have done extensive work to make the analysis reproducible, though I have not tried reproducing it myself.

We appreciate the reviewer taking the time to try the web interface and for noting the emphasis on reproducibility. Ensuring that analyses can be readily inspected and reproduced was a central goal of the project, and we are grateful for the reviewer's engagement with this aspect of the work.

Referee #3

Varilly et al. present a new strategy for phylogenetic and photodynamic inference to study infectious disease outbreaks. Their main innovation is a set of Markov chain Monte Carlo algorithms that operate on the extended state-space of phylogeneties and detailed histories of mutations mapped on these phylogenies. As the authors noted themselves, the ideas of using this extended state-space was "in the air" for more than two decades, but none of the previous approaches managed to scale up MCMC on this state-space without taking some shortcuts (e.g., fixing a phylogeny). One always has to wonder if such breakthroughs are "real" or just being oversold as "real" by the authors, but it looks real to me, so I am excited and hopeful about these developments. As far as I can tell, the success of Varilly et al.'s approach is a combination of them parallelizing computations across different parts of the phylogenetic tree and availability of genomic data collected in densely sampled outbreaks. The authors note that sparse sampling leads to loss of efficiency of their algorithm relative to standard approaches that rely on Felsenstein's pruning algorithm for phylogenetic likelihood calculation. This reliance on dense sampling (to the best of my understanding the operational definition here is that each branch of the phylogenetic tree has a very small number of mutations mapped on it probabilistically speaking) potentially explains why previous attempts at using trees and mutational histories were not able to outperform state-of-the-art phylogenetic methods. In summary, very promising work that has real potential to transform Bayesian analyses of pathogen genomic surveillance data.

We are grateful for this thoughtful and generous assessment. We appreciate the reviewer's careful engagement with both the historical context of explicit-mutation approaches and the specific factors that make Delphy effective in practice. In particular, we value the reviewer's recognition that while the underlying ideas have been "in the air" for some time, the combination of scalable MCMC over extended state spaces and the characteristics of densely sampled outbreak data is what allows these ideas to be realized without the shortcuts that limited earlier work.

We also appreciate the reviewer's clear articulation of why dense sampling is central to Delphy's performance, and their framing of this dependence as an explanation for why previous attempts did not outperform state-of-the-art methods. We are encouraged by their assessment that the advance appears to be real rather than overstated, and we share their optimism that this approach has the potential to transform Bayesian analyses of pathogen genomic surveillance data.

Main critique points:

- I would like the authors to be a little more careful when they compare Bayesian and frequentist methods. I am mostly talking about the intro paragraph on page 2.

a. The authors state that Bayesian phylogenetics is the gold standard. I am not sure everyone will agree with this statement. It depends on what one wants to accomplish. For dating ages of the tree nodes, there is in principle nothing wrong with maximum likelihood, equipped with bootstrap to get confidence intervals for node ages. Non-Bayesian approaches become more cumbersome when one to add a hierarchical structures, but this has also been done in LAMARC and MIGRATE software packages. These tools are not popular in viral phylodynamics, but fundamentally they are comparable to BEAST.

We agree with the reviewer that the original wording in the Introduction overstated the case by describing Bayesian phylogenetics as a “gold standard” without sufficient nuance. We have revised this language to emphasize instead the generality of Bayesian phylogenetics and its role as a flexible scaffold for building hierarchical models, which is particularly important for applications such as outbreak reconstruction, phylodynamics, and joint inference of evolutionary and epidemiological processes.

To make this distinction concrete, we now more carefully contrast Bayesian phylogenetics with how maximum-likelihood methods are currently used in viral genomic epidemiology, namely through pipelines such as IQ-TREE 2 combined with TreeTime, which form the computational core of Nextstrain. We show that while these approaches often recover the broad structure of phylogenies efficiently, they can yield substantially different point estimates and uncertainty characterizations for key quantities such as mutation rates and times to most recent common ancestors.

Our intent is not to diminish the value of frequentist approaches, which remain powerful and appropriate for many tasks, including tree reconstruction and exploratory analysis. Rather, we aim to clarify why Bayesian formulations are particularly well suited for analyses that require coherent uncertainty quantification and the integration of multiple latent processes. We believe this revised framing more accurately reflects the strengths and limitations of the different approaches and addresses the reviewer’s concern.

I wouldn't call phylogeography parameters or effective population size "covariates." It could be confusing since in standard regression models covariates are assumed to be fixed/conditioned on, but objects that the authors refer to are unknown parameters or latent variables.

Agreed. We have revised the manuscript to avoid referring to phylogeographic parameters and effective population size as “covariates,” and now consistently describe them as model parameters or latent variables, as appropriate. This change eliminates the potential for confusion with standard regression terminology.

The authors' description of the posterior P(T,\theta | data) on page 4 is not fully rigorous. For example, it completely omits the coalescent prior. In my opinion it is cleaner to represent an EMAT as (T, M), where T is the phylogeny and M is mutational history on this phylogeny. Then the authors can write P(T, M) = P(M | T) P(T), where P(M | T) is the CTMC complete data likelihood (product of exponential densities and tail probabilities) and P(T) is the coalescent likelihood. In the Methods section, the authors have the coalescent present; they use \pi_{anc}(T | \theta) in place of my suggested P(T), where both represent the Kingsman's coalescent density. It is strange to have two terms with T | \theta in parentheses. I think separating T into (T, M) per my suggestion is cleaner. I also strongly suggest calling P(M | T) complete data likelihood instead of a "genetic prior." Although missing data and parameters are both random variables in a Bayesian setting, this is not in a frequentist setting, so it is easier to follow a more universal notation.

We agree with the reviewer that the presentation of the posterior on page 4 was not as clear as it should have been, and we appreciate the careful reading. While the ancestry prior was intended to be included implicitly via Pi(T, theta), we recognize that the original wording could be read as omitting the coalescent prior. We have revised the main text to make the factorization explicit, including a clear statement of the coalescent (ancestry) prior alongside the genetic component and the parameter priors, and we now cross-reference the corresponding Methods and Supplementary Information sections more directly.

We also agree that representing an EMAT as (T, M), with T the phylogeny and M a detailed mutational history, is a clean and familiar way to write the posterior as
P(T, M | data) proportional to P(data | T, M) * P(M | T) * P(T).

Our formulation is equivalent, but we chose to keep the mutational history bundled into the EMAT object T because Delphy treats the EMAT, including explicit mutation events, as part of the generative model being sampled. This makes it easier to maintain a consistent separation between model specification and data likelihood in an explicit-mutation framework, and it also anticipates future extensions in which ancestry and sequence evolution are more tightly coupled (for example, selection models), where the separation between P(M | T) and P(T) becomes less natural.

Finally, regarding terminology, we recognize the reviewer's point that "complete data likelihood" is familiar language in the CTMC literature. We have added explanatory text to motivate our use of the term "genetic prior" and to clarify its relationship to standard CTMC complete-data formulations. We hope these revisions make the page-4 presentation fully rigorous and easier to follow, without changing the underlying model.

> I am not fully convinced by the authors explanation of the disagreement between Delphy and BEAST2 when approximating the posterior distribution of the rate heterogeneity parameter \alpha. The authors blame BEAST2 discretization of the gamma distribution, which seems plausible. Can the authors confirm this hunch by increasing the number of gamma distribution discrete bins from 4 to 8 and then to 12? If the authors' explanation is correct, one would expect that increasing the number bins in BEAST2 should bring the two methods closer to each other.

This is a fair concern, and we agree that the explanation required explicit validation. In response to the reviewer's suggestion, we reran the BEAST analyses using different numbers of discrete Gamma rate categories to directly test whether discretization accounts for the observed discrepancy in the posterior distribution of the rate heterogeneity parameter alpha.

We performed additional BEAST runs using 2 and 8 discrete Gamma categories, in addition to the standard setting of 4, and report the results in Figure 2E. As shown in Figure 2E (reproduced below), these runs exhibit two clear effects. First, the inferred posterior distribution of alpha depends strongly on the number of discrete categories used in BEAST. Second, increasing the number of categories systematically shifts the inferred distribution toward the value obtained by Delphy, which uses a continuous Gamma model. In particular, the distribution obtained with 8 categories lies approximately halfway between the original 4-category result and the Delphy result, consistent with convergence toward the continuous limit.

Because the computational cost of likelihood calculations scales linearly with the number of Gamma categories, and these BEAST runs were already among the longest in our benchmarking suite, we did not initially extend the analysis to 12 categories. The 8-category run already demonstrates the expected convergence trend toward the continuous Gamma limit, making an additional 12-category run unlikely to change the interpretation while substantially increasing computational cost.

We believe this analysis directly supports our hypotheses that the discrepancy arises from Gamma discretization rather than from a substantive difference in inference, and that these discrepancies do not materially affect any other key inferences. We have updated the manuscript to include this figure and to clarify this point in the text. If the reviewer feels that an additional run with 12 categories would further strengthen this conclusion, we would be happy to include it.

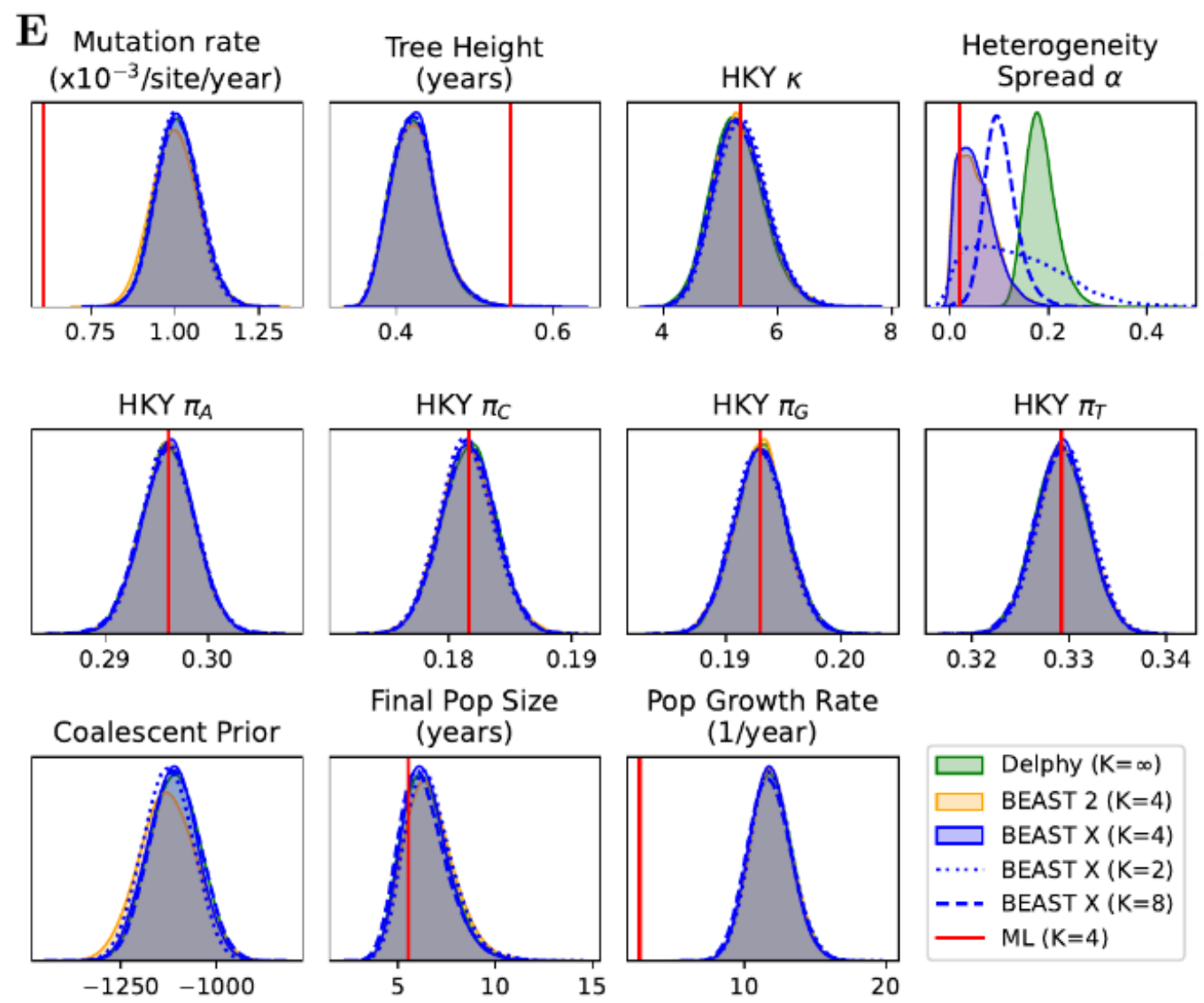

Figure 2E: Delphy is consistent with existing Bayesian tools. Distributions of key observables, as produced by Delphy (green) and BEAST 2 (orange), BEAST X (blue), and maximum-likelihood estimate (red); the discrepancy in the heterogeneity spread parameter α arises from Delphy's use of a continuous Gamma model vs. a K-category discrete approximation in the BEAST runs (Methods); the curves for K=2 (dotted line), K=4 (the standard setting, filled blue), and K=8 (dashed line) demonstrate the sensitivity of α to K and its convergence towards the Delphy result (K=∞).

Minor critique points:

- I would add RevBayes to the list of state-of-the-art Bayesian phylogenetic tools. This software is more actively developed than MrBayes and is comparable to BEAST in functionality.

Agreed. We have added RevBayes to the list of state-of-the-art Bayesian phylogenetic tools and updated the text accordingly.

- "Whereas traditional methods effectively perform this integration analytically, Delphy performs them it statistically" -> "Whereas traditional methods effectively perform this integration analytically, Delphy folds this integration into MCMC" -- this is how Bayesian data augmentation is usually explained, so I would stick to this point of view.

We agree with the reviewer's suggestion in principle. However, at the point in the manuscript where this sentence appears, MCMC has not yet been introduced explicitly as the sampling framework. For clarity and consistency with the surrounding text, we therefore use the phrasing "Delphy folds this integration into the sampling," and introduce the MCMC-specific language later, once the sampler is defined.

- On page 25, in the Posterior distribution section, G(T | \theta) is written as G(T, \theta) in the itemized list below the formula.

Agreed. The notation has been corrected from G(T, theta) to G(T | theta).

Referee #3 (Remarks on code availability):

I poked around GitHub and run the software demo. I haven't really done any stress testing of the software though.

We appreciate the reviewer taking the time to explore the GitHub repository and to run the software demo. We are glad that the interface and demonstration were accessible, and we thank the reviewer for engaging with this aspect of the work.

Referee #4

I co-reviewed this manuscript with one of the reviewers who provided the listed reports.

We appreciate the reviewer’s time and effort in co-reviewing this manuscript. We value the co-review process and are grateful for the thoughtful feedback provided.

Referee #5

This paper provides a comprehensive description of a new way to do Bayesian phylogenetics and represent a tremendous amount of work, the details of which are clearly written up. The authors went to great lengths to optimise every detail of the Delphy system, which now makes it possible to perform large scale Bayesian phylogenetic analyses with little effort. I enjoyed reading about the innovations in these optimisations, such as the clever exploitation of Jukes Cantor model to simulate new mutations, the exploitation of explicit mutations to inform candidate locations for SPR moves and application of Merkle trees to obtain summary trees to name just a few. The user interface is intuitive and works well, so I am sure it will be appreciated by the non-specialists that the software is catering for. This has the potential of revolutionising the way large scale pathogenic outbreaks will be managed.

We are very grateful for this thoughtful and encouraging assessment. We appreciate the reviewer's careful reading of both the technical details and the broader system design, and we are pleased that the combination of explicit-mutation representations, optimized MCMC moves, and efficient summarization techniques came through clearly. We are also glad that the usability of the interface resonated, as making large-scale Bayesian phylogenetic analysis accessible to non-specialists was a central motivation for this work. We appreciate the reviewer's optimism about the potential impact of this approach on pathogen genomic surveillance and outbreak response.

I mainly concentrated on the technical details in the supplementary information, and have not been able to find any major flaws. However, there are a few things I would like to see clarified, mainly around validation of the implementation.

Correctness of the implementation is supported by pointing out results are identical to BEAST2 output. As proof, some marginal posterior and tree figures are presented. Given quite a few optimisations and approximations are involved and there are many technical details, from experience I would expect there to be at least some bugs, so thorough testing is required. I would not consider this sufficient evidence the implementation is correct. Performing a well calibrated simulation study (WCSS) is the current threshold of proof to demonstrate it is. Tools described in Mendes et al. (2025) can be used to do such studies.

A WCSS demonstrates measurable properties are correctly estimated, but does not make claims about the trees, and since new tree operators were introduced it is desirable to demonstrate the posterior tree distribution is correct as well. pg 20 line 338 says "The Delphy- and BEAST2-inferred MCC trees for the SARS-CoV-2 dataset show excellent agreement (Fig 3A and 3B)." is hard to verify by looking at a picture on PFD. Please use clade support and height graph to demonstrate the tree distributions agree (e.g. as described here: https://www.beast2.org/2020/04/20/comparing-tree-sets.html). A nice to have, as part of the WCSS is to see "triangle graphs" (https://github.com/rbouckaert/DeveloperManual?tab=readme-ov-file#clade-coverage) to demonstrate clade support is correctly estimated.

We appreciate the reviewer's careful focus on validation and correctness, and we agree that this is a central concern given the number of algorithmic optimizations and approximations introduced in Delphy.

Validation in this work is supported by multiple, complementary layers of testing and benchmarking carried out over nearly four years of development. At the system level, we show close agreement with established Bayesian phylogenetic tools across a wide range of real and simulated datasets, including agreement in

posterior distributions of key parameters, inferred population dynamics, and tree summaries. In addition, we include simulation-based benchmarks with known ground-truth parameters (Figure 7), which demonstrate accurate recovery of mutation rates, population size trajectories, and other observables. The one notable deviation observed for the largest simulated dataset is traced to discretization effects in the parallelized coalescent prior and is explicitly analyzed (SI Fig. 10).

At the implementation level, a substantial portion of this development period was devoted to targeted testing and validation of the most error-prone components of the system, including SPR and mutation-directed SPR proposals, mutation-history sampling, handling of missing data, and evaluation of posterior ratios. These components are covered by comprehensive unit tests that are run automatically with each build. Over the course of development, discrepancies in these elements consistently manifested as either pathological behavior in MCMC traces (for example, abrupt jumps, stalled mixing, or inconsistent acceptance rates) or statistically significant disagreement in posterior summaries when compared against reference implementations. The absence of such signatures across the diverse benchmarks presented provides strong evidence that these components are behaving as intended for the class of viral genomic epidemiology datasets studied.

We agree with the reviewer that well-calibrated simulation studies (WCSS) and detailed clade-coverage analyses represent a high standard for validating Bayesian phylogenetic software, particularly for tree distributions. We considered including such analyses here. However, at the dataset sizes emphasized in this manuscript, existing WCSS and clade-coverage tooling is itself computationally prohibitive. For example, applying standard clade-coverage and height-graph tools to even moderate-sized tree sets proved impractical, and adapting these tools to scale using Delphy's internal representations would require substantial additional development and validation beyond the scope of the present work.

As a step toward addressing tree-level agreement, we annotate MCC trees with high-support clades and expand validation across multiple outbreak datasets, models, and sampling regimes, including non–SARS-CoV-2 outbreaks and flexible population models. Taken together, these results provide concrete, practical evidence of correctness, while acknowledging that no finite validation regime can exclude all possible implementation errors.

Our intent with this initial release is to make Delphy available as a robust and scalable tool for large-scale Bayesian phylogenetics, while encouraging careful interpretation and cross-validation where appropriate. To support this, Delphy outputs are fully compatible with BEAST 2 and BEAST X workflows, enabling direct comparison and diagnosis if unexpected results arise. We expect confidence in the approach to continue to grow through ongoing use, scrutiny, and extension by the community.

MCC trees are used to summarise posterior tree sets, and though they were the default for BEAST2 users for many years, it should be acknowledged they are not good representatives of posterior distributions (Berling et al, 2025). Conditional clade distributions (CCDs), in particular the CCD0 variant, allow finding a maximum a posteriori tree efficiently, but is arguably more computationally complex to create. The Merkle tree approach will elevate some of that. I suppose a workaround is exporting the tree set and run TreeAnnotator with the CCD0 topology option, but would it be feasible to implement a CCD0 summary tree or any other type of summary tree that is at least not unrepresentative as MCC trees?

We agree with the reviewer that maximum clade credibility (MCC) trees are an imperfect summary of posterior tree distributions, and we appreciate the thoughtful discussion of alternatives such as conditional clade

distributions (CCD0) and related approaches. We have added a reference to CCD0 trees, as well as HIPSTR, in the manuscript as examples of promising alternative summary methods that could be integrated into Delphy in the future.

For the present paper, our choice to use MCC trees was deliberate and pragmatic. We are targeting a broad audience for whom MCC trees remain the most commonly used and recognizable summary, and we are comparing Delphy against previously published datasets and analyses that themselves report MCC trees. Using the same summary method therefore allows direct visual and conceptual comparison, even while acknowledging its limitations.

We agree that alternative summaries such as CCD0 trees can provide a more faithful representation of posterior structure, but they are also more computationally demanding, particularly at the scales emphasized in this work. While Delphy's internal representations, including its use of Merkle-tree–based fingerprinting, make such approaches increasingly feasible, implementing and validating CCD-based summaries within Delphy would require additional development beyond the scope of the present manuscript.

In response to the reviewer's comment, we have added explicit guidance in the text noting that Delphy can export full posterior tree sets, enabling downstream analysis with external tools such as BEAST 2's CCD0 calculator or related software. This provides users who require alternative posterior summaries with a clear and supported workflow today.

We view improved posterior tree summarization as an important area for future work and appreciate the reviewer's suggestions in this direction.

One use case is to use Delphy to find a reasonably good tree, then do post processing on the summary tree that comes out of Delphy, e.g. to infer some phylodynamic process. Delphy makes it now possible to do this for large number of sequences. However, the MCC tree produced by Delphy often contains negative branch lengths due to parent clade ages being estimated younger than their child ages. This is inevitable when using the mean age of observed clades as age estimates when there are many clades and most of them observed at low frequency (which is the case for many datasets Delphy is aiming at). The common ancestor approach to age estimation (Heled & Bouckaert, 2013) evades that problem. Can this be implemented, and if not, do you have suggestions to evade this problem?

We agree with the reviewer's diagnosis of the issue. Using mean clade ages to construct MCC trees can indeed lead to negative branch lengths when parent clades are estimated to be younger than their descendants, particularly in large trees with many low-frequency clades.

All MCC trees shown in this paper are, in fact, computed using the common ancestor (CA) approach to age estimation described by Heled and Bouckaert (2013), which avoids this problem. We have now made this explicit in the manuscript (including a reference to Heled and Bouckaert (2013)) to prevent any ambiguity.

Delphy already supports CA-based age estimation internally. We experimented with making CA ages the default behavior in the web interface, but ultimately chose not to do so by default because many users in our target audience are more familiar with mean-age MCCs, and early user testing suggested that the characteristic appearance of CA MCCs, with multiple long branches emanating from key internal nodes, could be confusing in exploratory settings. For this reason, CA-based age estimation remains available in the web interface under a feature flag and could easily be exposed to users in the future.

For users who wish to perform downstream analyses on summary trees, including phylodynamic post-processing, we recommend using CA-based MCCs or exporting posterior tree sets for use with external tools, depending on the application. We are happy to revisit the default behavior as usage patterns evolve, but we currently aim to keep the interface as simple as possible while supporting more advanced workflows when needed.

A nice new method for handling missing data is introduced, and motivated by pg 14 line 243 "However, during SPR moves on subtrees with missing data, such imputation would require global rearrangements, as for example, in an SPR on a subtree whose tips have no data at site I, while the state of that site at P and P' differs". This is unclear to me: tips can be annotated with labels just like branches can be annotated with mutations (a minor benefit is that the same technique works for dealing with ambiguous characters). These values can be resampled occasionally, perhaps more often when involved in SPR moves, but their contribution can be integrated out by MCMC. This may be less efficient than using missations, but do not require global rearrangements with every SPR move. Would it be possible to provide a better motivation for missation handling?

We appreciate the reviewer's thoughtful suggestion and agree that, at first glance, treating missing or ambiguous tip states as latent variables to be resampled within MCMC appears to be a natural and appealing approach. In fact, this was also our initial strategy for handling missing data.

In practice, however, we found this approach to be prohibitively inefficient. The difficulty arises because, without missations, many SPR moves involving tips or subtrees with missing data require the Markov chain to pass through highly improbable intermediate states. Concretely, consider a tip with an ambiguous state (for example, A or C) at a given site. That tip can often be regrafted onto branches carrying either state with comparable posterior probability. However, transitioning between these two equally likely configurations without missations requires introducing a spurious mutation on a short branch, followed by a subsequent move that removes it. Such intermediate states are strongly penalized under realistic evolutionary rates. For SARS-CoV-2–like data, a single spurious mutation on a week-long branch reduces the posterior probability by a factor on the order of 10^4 to 10^5, leading to extremely slow mixing.

This problem becomes substantially worse when entire subtrees with missing data are involved. In that case, regrafting requires traversing a sequence of increasingly improbable intermediate configurations before reaching another high-probability state, causing mixing times to degrade by orders of magnitude.

Missations were introduced specifically to address this failure mode. By partially integrating over sites that are missing in all downstream tips, missations allow SPR moves to connect configurations with similar posterior probability directly, without forcing the chain through unlikely intermediate states, while retaining an explicit-mutation representation for all informative sites. We have added text to the main manuscript (in the paragraph beginning "Finally, Delphy introduces N-pruning…") to summarize this motivation more clearly.

We agree that missations are not the only conceivable solution, and we would welcome simpler alternatives. One idea we have explored conceptually is to retain fully specified mutational histories everywhere in the tree, but to augment SPR moves such that, whenever a move would otherwise introduce a spurious mutation at a site below a missing-data region, the mutational history of only that site would be resampled over the affected subtree. In most cases this resampling would correspond to unconstrained evolution under the underlying CTMC and could be implemented using a straightforward Gillespie algorithm. In such a scheme, missations would need only track which sites are missing in all downstream tips, not their states, simplifying some

bookkeeping, while allowing the tree to be treated as fully specified for other MCMC moves. While conceptually appealing, we have not yet found an implementation of this approach that achieves efficiency and simplicity comparable to the current missation framework, and exploring such alternatives remains an interesting direction for future work.

> The Supp info contains all technical details of interest, is well written and easy to follow when reading linearly, but it is hard to track down symbol definitions. It could benefit from a table listing all symbols used in the paper so that, for example, reading Eq (26) it can be easily checked what the definitions are of all symbols.

We agree with the reviewer that, while the Supplementary Information presents the technical details clearly when read linearly, it can be difficult to quickly locate symbol definitions when consulting individual equations. In response, we have added two supplementary tables at the end of the SI that summarize the key notation used throughout the paper.

Specifically, one table collects the core notation associated with EMATs, and a second table summarizes the symbols used to define and parameterize the main probability distributions. As part of this revision, we also numbered several previously unnumbered equations to make cross-referencing easier.

We did not attempt to create an exhaustive table of every symbol appearing in the SI, as much of the notation is local to specific derivations and is defined immediately where it is used. Instead, we focused on symbols that recur across sections and play a general role in the exposition, including those needed to interpret equations such as Eq. (26). We believe this addresses the spirit of the reviewer's suggestion while keeping the supplementary material readable.

> **Minor things:**
>
> Eq(6) appears to have extra p before G on LHS of equation.

Noted and fixed.

> pg7 line 129ff Why call it "genetic prior" while at the same time explaining "The genetic prior functions similarly to the Felsenstein tree likelihood". Feels more natural to call it a (tree) likelihood and incorporate what is called likelihood on line 132.

A more detailed discussion of this choice, and its relationship to standard likelihood-based formulations, is provided in our response to Referee #1 (page 12 above) and in the revised text on page 4. Briefly, in Delphy's formulation the mutational history is treated as part of the generative model being sampled, rather than as observed data conditioned on a fixed tree, which motivates treating this term as a prior over models rather than as a likelihood. This choice anticipates future work modeling selective effects, where ancestry and genetics are coupled and no longer neatly factorize into an "ancestry prior" and a "tree likelihood". We have added a short clarifying explanation at this point in the manuscript to make this distinction explicit.

pg14 line 237 "We note that mdSPR's robustness degrades when many tip dates have large uncertainties, so Delphy is currently unsuitable for analysing such datasets." Can you speculate what can be the cause of this behaviour? Is it because long branch lengths can be introduced containing many mutations perhaps?

This is a thoughtful question, and we agree that the observed degradation in mdSPR robustness when many tip dates have large uncertainties merits some discussion. We have added a brief speculative explanation in the manuscript, and we emphasize that this explanation is conjectural.

Our working hypothesis is that when tip date uncertainty becomes large, the additional entropy introduced by longer branches and broader ranges for mutation times grows faster than the genetic prior can constrain it. As a result, the posterior landscape becomes increasingly diffuse, and proposal mechanisms that rely on sequence similarity to guide efficient rearrangements, such as mdSPR, become less effective. Importantly, we suspect this behavior is not specific to Delphy, but reflects a more general challenge in Bayesian phylogenetics when a substantial fraction of tips have highly uncertain sampling times.

We also explored whether this behavior could be mitigated by introducing more complex proposal moves that jointly regraft subtrees and resample tip dates. In particular, we implemented and tested an extended SPR move that simultaneously proposes a new attachment point and a new tip date to avoid passing through unlikely intermediate configurations. In practice, however, this did not lead to measurable improvements in mixing or robustness, and we therefore do not believe the observed effect is primarily due to slow mixing of tip-date proposals.

While this phenomenon is interesting in its own right, a systematic investigation would require a broader study of tip-date uncertainty and its interaction with tree proposal mechanisms, which we felt was beyond the scope of the present paper. We have therefore limited ourselves to noting the effect, offering a plausible explanation, and clearly delineating the regime in which Delphy is currently best suited.

pg 21 line 354 "Delphy achieves a 1000x higher statistical efficiency than BEAST2" v2.6, while BEAST v2.7 has been available since September 2022 and has a number of operator improvements specifically targeting mixing of tree distributions with tip dates. A fairer comparison is to v2.7 instead of v2.6.

We agree with the reviewer that comparisons should be made against current versions of BEAST, particularly given the operator and mixing improvements introduced in BEAST 2.7. In response, we have updated all benchmarks to use BEAST 2 v2.7.7 and BEAST X v10.5.0, and we indeed observe the effects of these improvements, especially for smaller datasets.

These updated comparisons strengthen the manuscript in two ways. First, they confirm that recent BEAST improvements meaningfully narrow performance differences at smaller scales. Second, they show that the fundamental scaling distinction emphasized in this work remains unchanged. Specifically, methods based on Felsenstein pruning continue to incur costs that scale with the number of site patterns in the data, whereas Delphy's explicit-mutation formulation allows most operations to scale with the number of mutations affected by a move. As a result, Delphy continues to achieve substantial gains for larger, densely sampled datasets,

including the SARS-CoV-2 dataset (772 sequences), the Ebola dataset (approximately 1,600 sequences), and the H5N1 dataset (approximately 3,300 sequences).

To reflect these updated benchmarks accurately and conservatively, and in line with the revised framing in the cover letter and main text, we have softened the original claim of “1,000× speedups” to “2–3 orders of magnitude speedups.” This change both accounts for improvements in BEAST since the project began and emphasizes that the magnitude of the speedup depends on dataset size and structure. We believe this revised language more faithfully represents the results and addresses the reviewer’s concern.

> Maybe I overlooked it, but how are site patterns treated? Equal site patterns get same mutation history or does each site gets its own? If the former, how is it verified this is correct?

Nothing was overlooked here. Delphy does not explicitly use site patterns, and we have clarified this point in the manuscript. Each site is assigned its own mutational history and is treated independently in the explicit-mutation representation. While sites with identical observed patterns may plausibly have mutations on the same branches, there is no requirement that those mutations occur at the same times, and Delphy does not enforce such coupling.

This site-wise treatment is essential to maintaining correctness under continuous-time models of evolution and avoids assumptions that would otherwise need to be justified or verified. We believe this clarification addresses the reviewer’s question.

> pg 40 line 502: what is a "96 vCPU AWS instance"?

We have expanded this description to avoid jargon. The text now reads “a 96-core (vCPU) virtual machine rented from Amazon Web Services (AWS).” We also specify that this corresponds to a c7a.24xlarge instance and provide details in the Methods and in the README.md of the accompanying benchmarking repository.

> pg 42 line 532 "Separately, our parallelization implementation needs further optimization; for example, the 100,000-sample run used less than 25% of the machine’s compute capacity at any given time." Could this be because at that size calculation is memory bound instead of CPU bound, or is it a matter of changing operator frequency, or something else?

This is a helpful question. The limited utilization observed in the largest runs is not due to memory bandwidth constraints, but rather to serial portions of the current implementation and conservative choices around operator scheduling.

We can state with high confidence that the calculation is very unlikely to be memory-bound. One of the key advantages of Delphy’s explicit-mutation representation is its compactness. For densely sampled datasets, the memory footprint of an EMAT is typically under approximately 100 bytes per tip, so the subtree examined by any given partition often fits entirely within L1 cache. In addition, all core operations are strictly local. For example, an SPR move that regrafts a subtree from its original attachment point *P* to a new point *P’* only traverses the path between those two points, visiting the intervening nodes and mutations.

The dominant sources of underutilization instead arise from serial bottlenecks in the current parallelization scheme, including:

- **Tree partitioning:** trees are partitioned on a single core, and data are copied into new subtree structures in memory.
- **Tree reassembly:** modified subtrees are reassembled on a single core, again requiring copying of data back into a global tree representation.
- **Recomputation of global quantities:** after reassembly, central quantities such as the posterior probability, total branch length, and related statistics are recomputed serially from scratch, rather than incrementally and in parallel from the subtree-level changes.
- **Global MCMC moves:** several global moves that are independent in principle (for example, mutation rate updates, population curve updates, and site-rate heterogeneity updates) are currently executed serially.
- **Expensive global updates:** some global moves, notably those involving site-rate heterogeneity and population curve updates, involve operations that scale with the number of sites or parameters (for example, drawing **L** independent samples from Gamma distributions) and are currently confined to a single core.
- **Operator frequency tuning:** we have not yet performed a systematic tuning of the relative frequencies of local versus global moves, or among different global moves, to optimize parallel efficiency.

We have expanded the corresponding paragraph in the Discussion to clarify these points, while keeping the main text accessible. Overall, the current parallelization should be viewed as a proof of correctness and feasibility rather than a fully optimized implementation, and we expect substantial additional performance gains from addressing these serial bottlenecks in future work.

pg 39 Figure 6: where does the geographical reconstruction come from?

The geographic reconstruction shown in Figure 6 is obtained using a simple parsimony reconstruction on the MCC tree. We have added a sentence to the figure caption to make this explicit and clarified this point in the main text.

In the mpox vignette, where this reconstruction plays an important illustrative role, we explicitly note its simplicity and contrast it with more sophisticated phylogeographic analyses, such as those in Parker et al. While this parsimony-based approach is crude, we have found it effective at recovering broad geographic patterns in the densely sampled genomic epidemiology datasets targeted here.

We agree that more sophisticated geographic inference would be valuable and consider this an important direction for future work.

Supplementary figure 17: What is the difference between closed and open circle (path 3 vs 4)?

The distinction between the closed and open circles in Supplementary Figure 17 corresponds to two different proposal paths. The closed-circle path (path 3) applies to sites for which the state at the root is preserved, because there are informative tips on the opposite side of the root (illustrated by sites 37–40 in the example).

The open-circle path (path 4) applies to sites for which the root state must change as part of the proposal, because the only informative tips are downstream of node X (sites 35–36 in the example).

This distinction arises in the case where the set L_{I+1} is nonempty, as discussed near the end of the corresponding subsection. Because this is a subtle point and not central to the main message of the figure, we have added a brief note to the figure caption to clarify the meaning of the symbols without distracting from the overall illustration.

Supp info pg 39 "even though mdSPR exposes no tunable parameters" f could be a tunable parameter?

Agreed. The intent of the statement is that mdSPR does not *expose* tunable parameters to the user. While the parameter f exists internally, we have found that a single fixed value performs well across all datasets we have examined, and we therefore do not make it user-tunable. We have clarified this wording in the Supplementary Information to avoid confusion.

Supp info pg 43 k_c in Eq (29) can be non-integer?

Yes, k_c can be non-integer. We have added a note in the Supplementary Information to clarify this point and replaced the expression (k_c choose 2) with the equivalent form k_c (k_c - 1) / 2 to avoid any confusion. While the binomial coefficient does extend naturally to non-integer values in this case, the revised expression makes the intended interpretation explicit.

In rare cases, particularly for the cell containing the root, k_c can drop below 1, which would make k_c (k_c - 1) negative. We treat this as a minor discretization artifact that can be made arbitrarily small by increasing the time discretization resolution, and it does not affect the conclusions of the analysis.

Supp info pg 17 "where T is the total branch length of the tree and M is the total number of mutations on it" add " and L is total number of sites"

Noted and fixed.

Supp info pg 39 supp info "moveset"?

We have removed the term "moveset" for clarity.

Supp info pg 49 "fingerprints are all essentially independent random C-bit fingerprints." Since internal node fingerprints within a tree are functions of tips below the node, this seems the opposite of independent. Can this be substantiated? Did you verify experimentally the collision numbers for tree sets produced by Delphy?

The reviewer is correct that the original phrasing was imprecise. Full independence does not hold for fingerprints of internal nodes, since those fingerprints are deterministic functions of the tips beneath them. We have revised Section 11 of the Supplementary Information to be more precise.

The property required for our analysis is not full independence, but pairwise independence and uniformity of the fingerprints. Leaf fingerprints are chosen to be independent and uniformly random C-bit strings. The fingerprint of any clade is defined as the XOR of the fingerprints of its descendant leaves. Because the XOR of independent uniformly distributed bitstrings is itself uniformly distributed, the fingerprint of any clade is uniformly distributed over C-bit strings.

Moreover, fingerprints of distinct clades are pairwise independent. For two clades A and B, their fingerprints satisfy F_B = F_A XOR F_C, where C is the symmetric difference of the two clades. Since F_C is itself the XOR of independent, uniformly distributed leaf fingerprints disjoint from those contributing to F_A, it is uniformly distributed and independent of F_A. As a result, the joint distribution factorizes, and the probability that two distinct clades have identical fingerprints is exactly 2^(-C).

This level of independence is sufficient for bounding collision probabilities. The probability that any collision occurs among the set of clades observed across posterior samples can be bounded using a standard birthday-type argument that relies only on pairwise independence. We have tightened the exposition in the SI to make this explicit and to avoid overstating the independence assumptions.

Given the large fingerprint sizes used in practice, this bound implies an extremely low probability of collision, and we have not observed collisions in any of the tree sets produced by Delphy in our benchmarks.

Nice to see the number of mutations for the COVID demo in the web interface converging to the number of the BEAST.

We appreciate the reviewer noting this agreement :)

# References

L Berling, J Klawitter, R Bouckaert, D Xie, A Gavryushkin, A Drummond. Accurate Bayesian phylogenetic point estimation using a tree distribution parameterized by clade probabilities PLOS Computational Biology 21.2 (2025) DOI:10.1371/journal.pcbi.1012789

Heled and Bouckaert: Looking for trees in the forest: summary tree from posterior samples. BMC Evolutionary Biology 2013 13:221.

Mendes FK, Bouckaert R, Carvalho LM, Drummond AJ. How to validate a Bayesian evolutionary model. Systematic Biology. 2024 Nov 7:syae064. DOI:10.1093/sysbio/syae064

Remco Bouckaert

We thank the reviewer for these relevant references and have added them to the manuscript where appropriate.

Referee #5 (Remarks on code availability):

The code is nicely commented and well organised.

The code runs flawless in browsers and the binary for Linux worked on an x86 Ubuntu computer.

However, I ran into problems building the program from source. Installation instructions may require some improvements. Perhaps mention 'cmake' as requirement (had to install separately on OS X). Could not get it to go due to compiler error:
[...*explicit error messages elided*...]

We thank the reviewer for their careful testing of the code and for taking the time to report these build issues. We appreciate the positive comments on code organization and on the browser-based interface and Linux binary.

In response to the reported compilation problems, we have fixed the underlying issues and updated the installation instructions to make all dependencies explicit, including the requirement for cmake. We have tested the revised INSTALL.md on fresh AWS environments covering Ubuntu 22.04 (x86-64), Ubuntu 24.04 (x86-64), macOS 15 (Sequoia) on x86-64, and macOS 15 (Sequoia) on Apple Silicon (M1), and confirmed that the code now builds successfully in each case.

We are grateful to the reviewer for helping us improve the robustness and clarity of the installation process.

Referee #1

Remarks to the Author

The authors have done a great job of responding to reviews and improving the paper.

Personally, I'm very happy they chose to change the license and I hope that this stimulates more interest and engagement with the community. Bravo!

We thank the reviewer for the many helpful suggestions, particularly the expansion of benchmarks to additional viruses and the inclusion of tree correlation ESS. The WCSSs and clade correlation analyses requested by Reviewer #5 also revealed minor convergence difficulties in a small subset of cases, similar to those reported in Gao et al 2026 (now cited), which were not apparent in any previously reported measures, including tree correlation ESS. We highlight these effects in the revised manuscript and outline two practical approaches to address them: confirming that conclusions based on individual tips are robust across multiple runs, and avoiding tip dates with very large date uncertainties.

Remarks on code availability

This is a modern project that built easily (I only had to modify Conan's settings.yml for clang 17). There is extensive test coverage, which is rare for a bioinformatics program. As far as the code structure is concerned, one could argue that the Run god class is a structural wart, but overall this is well above current standards in the field.

We thank the reviewer for these comments. We have since added continuous integration scripts for GitHub to automatically compile binaries for Linux x86-64, Linux arm64 and macos arm64, now included with each release. In this process, we further hardened the build.

We also recognize that aspects of the current orchestration, including the Run & Subrun classes, could be improved. For this initial release, our priority was to demonstrate the performance gains enabled by the explicit mutation representation, which led us to favor a simpler overall structure. Improving modularity and flexibility of the orchestration will be a focus of future development.

Referee #3

Remarks to the Author

Good job revising the manuscript. Implementation of Skygrid is impressive! I am happy with the current version of the manuscript and have no further requests for the authors.

We thank the reviewer for the many great suggestions that helped strengthen the manuscript, particularly: a) the discussion on genetic prior vs augmented likelihood; b) the highlighting of RevBayes as an actively developed SOTA Bayesian phylogenetics tool (from which we are learning much about how to specify and orchestrate flexible models without resorting to BEAST-style XML input files); and c) the request to explicitly demonstrate how varying the number of rate categories in the discrete Gamma model for site-rate heterogeneity affects the inferred value of alpha and how this inference approaches the continuous Gamma limit.

Remarks on code availability

I ran the new demos. Core availability and user interfaces look superb.

We thank the reviewer for this very positive feedback and are pleased to hear that the core availability and user interface are working well in practice.

Referee #4

Referee #4 (Remarks to the Author):

I co-reviewed this manuscript with one of the reviewers who provided the listed reports.

We thank Referee #4 for their careful reading and contribution to the review process, and we appreciate their role in strengthening the manuscript.

Referee #5

Referee #5 (Remarks to the Author):

Thanks for the authors of addressing most of my questions in such great detail. Almost all my concerns are addressed by the authors. The list of symbols in the supp info is much appreciated.

Something that somewhat surprised me is that no WCSS is included in the ms, even though the authors say they considered it. The argument that it would be too computationally expensive is not convincing -- I have done WCCSs with BEAST2 using 1000 taxa trees, which if replicated with Delpy would already give substantial evidence there are no major issues with the implementation. Not having this evidence for even smaller cases than aimed for puts doubt on whether it works for larger cases.

We agree with the reviewer on the importance of including a WCSS in the manuscript. We have now carried out a series of comprehensive WCSSs, and the results substantially strengthen the paper by providing strong evidence that Delphy's MCMC implementation samples correctly across a broad range of model components and realistic settings. We now summarize this in the main text at the end of the section "Delphy implements Bayesian Phylogenetics using Explicit Mutation-Annotated Trees (EMATs)," with full details provided in the new Section 13 of the Supplementary Information.

To support these WCSSs, we extended Delphy's priors so they could be made proper without altering the default behavior of the software. We did this in ways that preserve conjugacy. Specifically:

1. We generalized the mutation rate prior from Uniform to Gamma (and corrected a typo in the expression for the Gibbs move for μ in SI Section 7.1).
2. For exponentially growing population models, we generalized the population size prior from 1/x to Inverse-Gamma, and the growth-rate prior from a fixed Laplace to a configurable and optionally bounded Laplace.
3. For Skygrid models, we added explicit control over the placement of the first and last knot times and introduced an Inverse-Gamma prior on the overall population level. We also corrected a small error in the derivation of one of the Skygrid moves (SI Section 7.3).

We also extended our phylogenetic simulator, Sapling, to support Skygrid population models, missing data, tip-date uncertainty, and site-rate heterogeneity. This made it possible to construct WCSS scenarios that exercise nearly all of Delphy's major features.

The main WCSS result is shown in Supplementary Figure 25. This analysis includes 200 replicate runs in a Skygrid setting with site-rate heterogeneity, missing data, and uncertain tip dates, and evaluates 16

observables using 95% HPD coverage, clade coverage ("triangle graphs"), and rank-uniformity diagnostics. These results are all consistent with correct sampling, within the expected finite-replicate variation.

We also include three complementary WCSS analyses:

- **Supplementary Figure 26** provides the corresponding result for an exponentially growing population model and supports correctness of that independent code path.
- **Supplementary Figure 24** probes Delphy outside the densely sampled regime by using a much wider mutation-rate prior while holding most other parameters fixed; this supports our claim that Delphy remains correct, though slower, in the presence of long branches with many mutations.
- **Supplementary Figure 27** extends the main WCSS setting to tips with year-long date uncertainties. This again shows broadly correct behavior, while also revealing modest tree-exploration difficulties in this extreme setting, which motivated our recommendation to avoid such large tip-date uncertainties until improved MCMC moves are developed.

The accompanying data repository, https://github.com/broadinstitute/delphy-2026-paper-data, includes scripts to run all of these WCSSs and reproduce the results. We organized each study as a simple sequence of four self-contained Python scripts that sample from the priors, orchestrate the inference runs with Delphy, analyze the outputs, and produce the final figures. For transparency, we also retained a set of exploratory WCSSs that helped build the study pipeline and the required extensions to Delphy and Sapling. Clade coverage was calculated using a tip-fingerprint analog of BEAST 2's CladeSetComparator.

Together, these analyses directly address the reviewer's concern and substantially strengthen the evidence for correctness of implementation. We are grateful to the reviewer for pressing this point, as the resulting analyses have materially improved the manuscript.

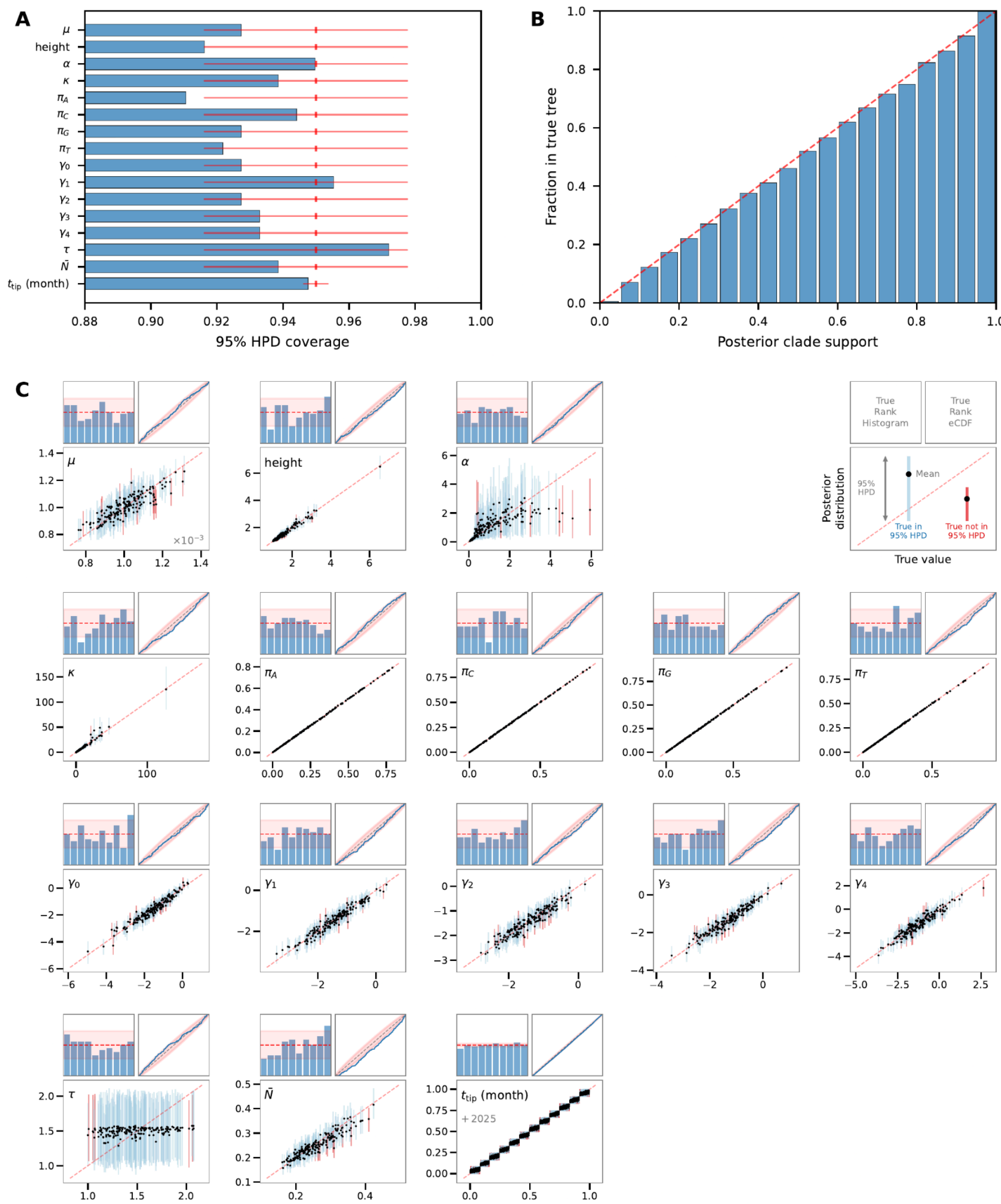

Supplementary Figure 25: Comprehensive Skygrid WCSS. Comprehensive Skygrid WCSS: mutation rate μ ~ Gamma(μ=10^{-3}, σ=10^{-4} mutations/site/year); Skygrid population model with 5 knots evenly spaced from 2025-01-01 to 2026-01-01, mean population Ñ ~ InvGamma(μ=0.25, σ=0.05 years) and precision τ ~ Gamma(μ=10^{-3}, σ=10^{-4}); site-rate heterogeneity with α ~ Expo(1.0); ~5% of bases marked as missing; and ~15% of tips having month-uncertain dates. **A.** Coverage Validation: for each inferred observable, the fraction of replicates whose true value is covered by the 95% HPD of the inferred posterior distribution (red bars = 95%, CI under correct sampling). **B.** Clade Coverage: each bin groups all clades across all posterior samples and replicates having the indicated posterior support, and bin heights are the fraction of respective true trees containing that clade (dashed diagonal = expected behaviour under correct sampling). **C.** Rank Uniformity Validation: for each observable, rank histogram shows the counts of replicates which the given true sample's rank among the associated posterior samples (red shading: per-bin 95% CI under correct sampling; *x* axis ranges from 0 to 1 and *y* axis starts at 0, with red dashed line at *N/K* with *K*=10 bins); rank eCDF shows proportion of replicates whose true sample's rank among associated posterior samples is below *p* (red shading: 95% CI under correct sampling; both axes range from 0 to 1); scatter plot shows true value (*x*) vs inferred posterior distribution (*y*) for each replicate (dot = mean, bar = 95% HPD, blue bar = true value covered by 95 HPD, red bar = true value not covered).

Also, I do not understand that the trees pictured in figures sourced from different programs are presented as evidence of correctness, given these trees invariably differ, sometimes substantially. This is not surprising, given that MCC trees are inherently unstable, making it hard to get the same pair of summary trees from even the same software package when run with different random seeds. I suggested a simple alternative to visualise this, which has been ignored. Another way of measuring tree mixing is presented based on Magee et al. (2024), but not shown to measure agreement between different runs from different software packages as far as I can tell.

My experience is that even after decades of BEAST development we are still are finding bugs. Altogether, it seems to me that the authors take on a substantial risk in leaving some parts of validating their method unexplored.

We have now implemented the reviewer's suggested clade correlation graphs using tip-fingerprinting (sources in the data repo), and used those to produce the requested detailed clade comparisons in each of the main vignettes of the paper (Figures 2 and 5, and Supplementary Figures 2–6).

We had not included these graphs previously because our initial focus was on broader agreement across MCCs, posterior distributions of key observables, and ESS-based measures of sampling efficiency, and because implementing detailed clade-level comparisons at this scale required additional tooling. However, the reviewer was entirely right that these graphs provide a much finer-grained view of posterior agreement than visual comparison of MCCs alone, and we are grateful to have been prompted to include them.

In the main text, the BEAST X versus Delphy comparisons, which mirror analogous BEAST 2 versus Delphy comparisons, show nearly complete concordance in clade support and especially clade tMRCAs. As the reviewer anticipated, however, these detailed comparisons are also more revealing than MCCs alone, and they expose a very small number of outlier clades.

Some of these differences reflect the deliberate site-rate heterogeneity model differences used in the main text (for example, compare Figure 2E and Supplementary Figure 2E). The remainder appear to reflect modest MCMC exploration difficulties in rugged tree landscapes, as described in Gao et al. 2026. In the new SI Section 12, we explore these discrepancies in detail. We find that the outliers are well explained by a handful of misplaced "errant" tips rather than by large-scale structural differences between trees. We also show that, for some datasets, similar outliers are already present in clade correlation graphs of BEAST X versus BEAST X runs, as well as in BEAST 2 versus BEAST X comparisons, indicating that these outliers are symptoms of broader sampling difficulties rather than evidence of a Delphy-specific implementation error.

We have accordingly highlighted this caveat in the discussion of Figure 2, in the H5N1 vignette, and in the broader discussion of the strengths and shortcomings of mdSPR. As in Gao et al. 2026, these sampling difficulties appear to have negligible impact on the main summary observables of interest, but they do suggest caution for conclusions that depend on the exact placement of a small number of tips. In such cases, we now recommend confirmation across multiple independent runs.

We hope that these additional clade correlation graphs satisfy the reviewer's request for much more fine-grained comparisons between the posterior distributions of Delphy and BEAST (X and 2). Overall, they provide strong evidence of the statistical indistinguishability of equally configured inferences across these tools, while also clarifying an underlying sampling difficulty, generic to current Bayesian phylogenetic inference, to which future methodological work, including our own, should be directed.

Probably due to some minor configuration issue, trying to compile the code from source, I am getting this error both on M1 and Ubuntu:

CMake Error at CheckGit.cmake:58 (LIST):
LIST index: 1 out of range (-1, 0)

On Mac, this may have to do with brew not properly installing emscripten:

~> brew install cmake emscripten
[...*explicit error messages elided*...]

We thank the reviewer for identifying this issue with our compilation instructions, and we appreciate their meticulous attention to detail. We have now hardened our compilation infrastructure to avoid the problem encountered here, which we believe arose when compiling a shallow git clone. We have also added GitHub CI actions to automatically compile CLI binaries included in each release for Linux x86-64, Linux arm64 and macos arm64. These changes ensure that the build pipeline is continuously tested in a controlled environment, and should allow most users to avoid compiling Delphy from source when they simply want to use the CLI tools.
